# Neuroanatomical and psychological considerations in temporal lobe epilepsy

**DOI:** 10.3389/fnana.2022.995286

**Published:** 2022-12-14

**Authors:** Javier DeFelipe, Jesús DeFelipe-Oroquieta, Diana Furcila, Mar Muñoz-Alegre, Fernando Maestú, Rafael G. Sola, Lidia Blázquez-Llorca, Rubén Armañanzas, Asta Kastanaskaute, Lidia Alonso-Nanclares, Kathleen S. Rockland, Jon I. Arellano

**Affiliations:** ^1^Laboratorio Cajal de Circuitos Corticales, Centro de Tecnología Biomédica, Universidad Politécnica de Madrid, Pozuelo de Alarcón, Madrid, Spain; ^2^Centro de Investigación Biomédica en Red de Enfermedades Neurodegenerativas, Madrid, Spain; ^3^Instituto Cajal, Consejo Superior de Investigaciones Científicas, Madrid, Spain; ^4^Gerencia Asistencial de Atención Primaria, Servicio Madrileño de Salud, Madrid, Spain; ^5^Facultad de Educación, Universidad Camilo José Cela, Madrid, Spain; ^6^Facultad de Educación y Psicología, Universidad Francisco de Vitoria, Madrid, Spain; ^7^Department of Experimental Psychology, Complutense University of Madrid, Madrid, Spain; ^8^Center for Cognitive and Computational Neuroscience, Complutense University of Madrid, Madrid, Spain; ^9^Cátedra UAM de “Innovación en Neurocirugía”, Facultad de Medicina, Universidad Autónoma de Madrid, Madrid, Spain; ^10^Sección Departamental de Anatomía y Embriología, Facultad de Veterinaria, Universidad Complutense de Madrid, Madrid, Spain; ^11^Institute of Data Science and Artificial Intelligence, Universidad de Navarra, Pamplona, Spain; ^12^Tecnun School of Engineering, Universidad de Navarra, Donostia-San Sebastian, Spain; ^13^Department of Anatomy & Neurobiology, Boston University School of Medicine, Boston, MA, United States; ^14^Department of Neuroscience, Yale School of Medicine, New Haven, CT, United States

**Keywords:** hippocampal connectivity, hippocampal sclerosis, epilepsy surgery, Rorschach test, Wechsler Adult Intelligence Scale, Wechsler Memory Scale, Rey-Osterrieth Complex Figure Test, projecting drawings

## Abstract

Temporal lobe epilepsy (TLE) is the most common form of focal epilepsy and is associated with a variety of structural and psychological alterations. Recently, there has been renewed interest in using brain tissue resected during epilepsy surgery, in particular ‘non-epileptic’ brain samples with normal histology that can be found alongside epileptic tissue in the same epileptic patients — with the aim being to study the normal human brain organization using a variety of methods. An important limitation is that different medical characteristics of the patients may modify the brain tissue. Thus, to better determine how ‘normal’ the resected tissue is, it is fundamental to know certain clinical, anatomical and psychological characteristics of the patients. Unfortunately, this information is frequently not fully available for the patient from which the resected tissue has been obtained — or is not fully appreciated by the neuroscientists analyzing the brain samples, who are not necessarily experts in epilepsy. In order to present the full picture of TLE in a way that would be accessible to multiple communities (e.g., basic researchers in neuroscience, neurologists, neurosurgeons and psychologists), we have reviewed 34 TLE patients, who were selected due to the availability of detailed clinical, anatomical, and psychological information for each of the patients. Our aim was to convey the full complexity of the disorder, its putative anatomical substrates, and the wide range of individual variability, with a view toward: (1) emphasizing the importance of considering critical patient information when using brain samples for basic research and (2) gaining a better understanding of normal and abnormal brain functioning. In agreement with a large number of previous reports, this study (1) reinforces the notion of substantial individual variability among epileptic patients, and (2) highlights the common but overlooked psychopathological alterations that occur even in patients who become “seizure-free” after surgery. The first point is based on pre- and post-surgical comparisons of patients with hippocampal sclerosis and patients with normal-looking hippocampus in neuropsychological evaluations. The second emerges from our extensive battery of personality and projective tests, in a two-way comparison of these two types of patients with regard to pre- and post-surgical performance.

## Introduction

Temporal lobe epilepsy (TLE) is the most common form of focal epilepsy (Box 1) and is associated with a variety of structural and cognitive alterations (reviewed in Morrell, 1989; Dinner, 1993; Morris and Estes, 1993; Bell et al., 2011; Nakahara et al., 2018; Tai et al., 2018). Numerous studies indicate that these cognitive and psychosocial alterations may be related to multiple factors such as cognitive effects of antiepileptic drugs, disruption of normal socialization, and abnormal brain functioning (for recent reviews see Helmstaedter et al., 2020; Kanner et al., 2020). Thus, the study of epilepsy represents an important link between neurology/neuroscience and psychiatry/psychology (Reynolds, 2020).

**BOX 1 Epilepsy disorders.**

The recognition of epilepsy disorders began in the second millennium BC and has evolved in parallel with the neurological sciences (e.g., Reynolds, 2020). According to the International League Against Epilepsy (ILAE) (Commission on Classification and Terminology of the International League Against Epilepsy, 1989), epileptic disorders can be categorized according to two major criteria: semiology and etiology. The first distinguishes epilepsies with generalized seizures (generalized epilepsy) from epilepsies with partial or focal seizures (localization-related, partial or focal epilepsies). The second distinguishes epilepsies in three main types according to their etiology: structural/metabolic, when there is a known lesion, malformation or metabolic disorder known to be associated with epilepsy; genetic, when the case is demonstrated or presumed to be genetic; and of unknown cause, when there is no subjacent cause (Berg et al., 2010). These terms replace the former classification of symptomatic, idiopathic and cryptogenic that are less strictly defined, but are still widely used in the literature.

Structural/metabolic epilepsies may arise shortly after an insult or disorder, or after a long latency period of up to several years (e.g., Ketz, 1974; Spencer et al., 1984a; Morrell, 1989; Dinner, 1993; Morris and Estes, 1993). It is thought that cortical circuits in the altered brain suffer a series of changes that eventually lead to epilepsy. Cognitive and psychosocial alterations are common in epileptic patients, as reflected in an updated definition by a task force of the ILAE: “Epilepsy is a disorder of the brain characterized by an enduring predisposition to generate epileptic seizures, and by the neurobiological, cognitive, psychological, and social consequences of this condition” (Fisher et al., 2005).

For unknown reasons, pharmacoresistance with regard to seizures is observed in approximately 40% of TLE patients. Of these, an estimated 10–50% might be considered good candidates for surgical treatment, although, for example in the USA, less than 1% are referred to an epilepsy center for surgical evaluation (Engel, 2018; see also Kwan et al., 2010). One of the variables commonly used to help determine which epileptic patients are likely to benefit from surgery is psychological examination, which allows the neurosurgeon to better understand the condition of the patients. The most common surgical procedure is the removal of the anterior temporal cortex (Engel and Shewmon, 1993), most of the amygdala and the anterior portion of the hippocampal formation (Penfield and Jasper, 1954; Falconer, 1971; Olivier, 1992; Fried and Spencer, 1993; Wieser et al., 1993; see Feindel et al., 2009 for a review of the history of the development of surgery for epilepsy). About 60–70% of these patients can be seizure-free with relatively few problems after surgery (e.g., McIntosh et al., 2001; Engel et al., 2003; Sola et al., 2005; Aull-Watschinger et al., 2008; Spencer and Huh, 2008; Thom et al., 2010; Ryvlin and Rheims, 2016; for recent reviews see West et al., 2019; Zhang and Kwan, 2019).

Another main objective of epilepsy surgery is to determine the anatomopathological condition of the resected tissue, which presents either a variety of pathological changes or no changes at all (Box 2). The most typical histopathological alteration observed in patients with TLE is hippocampal or Ammon’s horn sclerosis, characterized by neuronal loss and gliosis—most prominent in CA1, followed by the polymorphic layer of the dentate gyrus (DG), CA4 and CA3, while CA2 and the granule cell layer of the DG are relatively spared (Gloor, 1991; Meldrum and Bruton, 1992; Box 3). As outlined in the present report, since there is an extended anatomical hippocampal-frontal network, the existence of hippocampal damage is in line with the findings of psychological alterations that are detected with memory, personality, and emotion tests, which are more commonly administered in the context of frontal lobe disturbances. In fact, this hippocampal-frontal network has been extensively investigated in the context of psychiatric disorders (e.g., Aggleton and Brown, 1999; Godsil et al., 2013; Jin and Maren, 2015). However, histological alterations are remarkably variable between different fields of the sclerotic hippocampal formation—both within a given epileptic patient and between epileptic patients. Consequently, alterations of the hippocampal connections are also heterogeneous. Hence, the challenge is to correlate neuropathology with epileptic seizures and with the psychological features of the patients.

**BOX 2 Histopathological features: General.**

In cases with no conspicuous pathological alterations such as brain tumors or vascular malformations, standard histopathological examination of resected brain tissue reveals a range of findings that vary from severe neuronal loss to no loss at all (Meldrum and Bruton, 1992). The most typical alteration observed in patients with TLE is hippocampal or Ammon’s horn sclerosis, characterized by neuronal loss and gliosis in the hippocampus (e.g., Margerison and Corsellis, 1966; Falconer, 1974; Babb and Brown, 1987; Meldrum and Bruton, 1992; Babb and Pretorius, 1993; Wieser et al., 1993; Honavar and Meldrum, 1997; for a recent review see Blumcke et al., 2017). However, extrahippocampal pathologies in the temporal lobe are also found in up to 30% of cases with hippocampal sclerosis (“dual pathology”), focal cortical dysplasias being the most common alterations (Lévesque et al., 1991; Babb and Pretorius, 1993; Wieser et al., 1993; Cendes et al., 1995; Arai and Oda, 1997; Honavar and Meldrum, 1997; Nishio et al., 2000; Tassi et al., 2002; Salanova et al., 2004; Blümcke et al., 2011; Harroud et al., 2012).

Although the lateral temporal cortex removed from patients with TLE may show a normal appearance in standard histopathological preparations, the use of more specific immunohistochemical staining techiques may reveal alterations of cortical circuits that could be overlooked with standard histological methods. In fact, we have shown using correlative light and electron microscopic methods, that there are patches of decreased immunostaining for parvalbumin —which labels a subpopulation of powerful cortical GABAergic inhibitory interneurons including chandelier and basket cells (DeFelipe et al., 1989)—in the lateral temporal cortex of some patients. These patches may occur independently of the pathology associated with epilepsy (including hippocampal sclerosis) and represent small regions that also show abnormal synaptic circuits (DeFelipe et al., 1993; Marco et al., 1996; Marco and DeFelipe, 1997).

While tissue resected by TLE surgery comprises a relatively small portion of the temporal lobe, post-mortem analysis allows inspection of the whole brain and permits a more comprehensive understanding of the pathology associated with TLE. This whole brain type of histopathological study is not frequent and, in general, is not performed in detail or in all brain regions; but some observations can be drawn: (i) hippocampal sclerosis can be found bilaterally (Sano and Malamud, 1953; Margerison and Corsellis, 1966; Babb and Brown, 1987; Meencke et al., 1996; Thom et al., 2005, 2011); (ii) in addition to the hippocampus, neuronal loss is found in some cases in other temporal regions, including the neocortex and amygdala, as well as in extra temporal brain regions, such as the thalamus and frontal cortex (Sano and Malamud, 1953; Margerison and Corsellis, 1966). This finding is in line with quantitative MRI studies that indicate most patients with hippocampal sclerosis show widespread anatomical abnormalities (for a review see Bell et al., 2011); (iii) hippocampal sclerosis is not exclusive of TLE and severe damage of the hippocampal formation and amygdala is also observed in some individuals without seizures (Corsellis, 1957; Haymaker et al., 1958; Gloor, 1991). Hippocampal sclerosis in the elderly is not associated with seizure activity but with progressive memory loss and dementia (Dickson et al., 1994; Zarow et al., 2008).

**BOX 3 General nomenclature of the hippocampal region and main characteristics of the hippocampal sclerosis.**

In general, the term hippocampus is used in its broad sense, which includes the dentate gyrus (DG) and the hippocampus proper, or Ammon’s horn (Cornu Ammonis, CA fields). The DG is sometimes regarded as a separate structure of the hippocampus. The term hippocampal formation encompasses the DG, the hippocampus proper and the subicular complex (subiculum, presubiculum, and parasubiculum). However, other authors also consider the entorhinal cortex (EC) as part of the hippocampal formation (Insausti and Amaral, 2012). The hippocampus proper is commonly subdivided according to the nomenclature of Lorente De Nó (1934) into the CA1, CA2, CA3, and CA4 fields. However, there is some confusion regarding the nomenclature of the neurons contained inside the concavity of the DG that can be sometimes collectively referred to as “hilus of the dentate gyrus” or as CA4 (e.g., Thom, 2014). One example is in magnetic resonance imaging (MRI) studies, where the images lack enough resolution for further subdivisions and CA4 is often used to refers to “the region within the C-shaped DG” or “C4-DG” (e.g., see DeFelipe et al., 2007). However, a distinction should be made between the cells of the polymorphic layer of the dentate gyrus that include mossy cells, and the pyramidal cells of the Ammon’s horn. The first are normally labeled as hilar cells, while the latter are frequently labeled as CA4 following Lorente De Nó, 1934. Some authors, however, consider that there are no clear differences in cytoarchitecture or connectivity in humans to distinguish CA4 from CA3 pyramidal cells, and therefore do not differentiate a CA4 field in humans (Insausti and Amaral, 2012). Other authors defined the dentate hilus “as including the entire region between the blades of the DG” containing the polymorphic layer of the DG and the CA3 pyramidal neurons which would constitute the CA3 hilar neurons (subfield CA3h; Lim et al., 1997). For the purposes of the present work and unless otherwise specified, the hippocampal formation will refer to the DG, the hippocampus proper (subdivided into four fields: CA1, CA2, CA3 and CA4) and the subicular complex. “Alterations in CA4-hilus” will refer to the pathology found in the concavity of the DG, affecting both the hilus and CA4.

***Histopathological features: Hippocampal sclerosis***

Hippocampal sclerosis is characterized by gliosis and neuronal loss, most prominent in CA1, followed by the polymorphic layer of the dentate gyrus, CA4 and CA3, while CA2 and the granule cell layer of the DG are relatively spared (Gloor, 1991; Meldrum and Bruton, 1992). A common finding is the dispersion of the dentate granule cell layer, with ectopic neurons being found in the molecular layer (Houser, 1990; Houser et al., 1992) and microvascular changes (loss of microvessels and a variety of vascular alterations in the remaining blood vessels) in the sclerotic CA1 (Kastanauskaite et al., 2009). This neuronal loss and gliosis is accompanied by changes in the expression of a variety of molecules in the surviving cells, as well as axonal reorganization including both excitatory and inhibitory axons (e.g., de Lanerolle et al., 1989; Sutula et al., 1989; Babb et al., 1991; Mathern et al., 1995; Wittner et al., 2002; Arellano et al., 2004; Frotscher et al., 2006; Andrioli et al., 2007; Muñoz et al., 2007; Wittner and Maglóczky, 2017) and loss of synapses (Alonso-Nanclares et al., 2011). Historically, several types of hippocampal sclerosis have been proposed depending on the affected fields and magnitude of neuronal loss and gliosis. When neuronal loss and gliosis are present in CA1-4 and hilus fields it is named “Ammon’s horn sclerosis”, when restricted to CA4-hilus it is called “end folium sclerosis,” and when hippocampal sclerosis is accompanied by neuronal loss and gliosis in the amygdala and parahippocampal gyrus it is called “mesial temporal sclerosis” (reviewed in Thom, 2014).

We adopt the histological classification of hippocampal sclerosis into three main types (Blümcke et al., 2013; see also Thom, 2014) as follows: ILAE type 1, refers to classic hippocampal sclerosis characterized by severe neuronal loss and gliosis in both CA1 and CA4-hilus, while the rest of the hippocampal fields may present mild to severe damage; ILAE type 2, is characterized by predominant neuronal cell loss and gliosis in CA1, and ILAE type 3, by predominant neuronal cell loss and gliosis in CA4-hilus (endfolium sclerosis).

In summary, the resected brain tissue from patients with TLE presents either a variety of pathological changes or no changes. However, the fact that no changes are observed with standard histopathological methods does not mean that the brain tissue is normal. Numerous epileptogenic mechanisms and changes in neuronal circuits have been reported, including selective loss of neurons and synapses, reorganization of excitatory and inhibitory synaptic circuits, glial reactions and changes in the expression of a variety of neurotransmitters, peptides, calcium binding proteins and receptors. Whether these changes are secondary to epileptic seizures or represent a primary process which initiates seizures or, alternatively, protects against the development of seizure activity are enduring questions in the field of human epilepsy pathology (e.g., Sloviter, 1994; DeFelipe, 1999; Avoli et al., 2005; Cavazos and Cross, 2006; Ben-Ari, 2008; de Lanerolle et al., 2012; Scharfman and Brooks-Kayal, 2014; Huberfeld et al., 2015; Alexander et al., 2016; Wittner and Maglóczky, 2017).

Once the pathologist has examined selected portions of the resected tissue, the remaining tissue is also of great value for research purposes. For example, in the 1980s and 1990s, numerous studies were performed to analyze in vitro the functional characteristics of human brain tissue, with the major goal being to investigate the mechanisms underlying seizures and epileptogenesis (reviewed in Avoli et al., 2005; Köhling and Avoli, 2006). From the microanatomical point of view, this tissue is of considerable interest because it can be immediately immersed in the fixative and therefore the ultrastructure and quality of the labeling achieved using a variety of markers for histology and immunocytochemistry is comparable to that obtained in experimental animals (e.g., del Río and DeFelipe, 1994). Recently, there has been renewed interest in using this kind of tissue, in particular ‘non-epileptic’ brain samples with normal histology (removed at a distance from the epileptic focus), in order to study the normal human brain organization. A variety of methods, including microanatomical, molecular, genetic, biophysical and computational techniques have been successfully applied (e.g., Szabadics et al., 2006; Verhoog et al., 2013; Testa-Silva et al., 2014; Tian et al., 2014; Mohan et al., 2015; Varga et al., 2015; Eyal et al., 2016; Molnár et al., 2016). An important limitation of this approach is that it is not clear how different clinical characteristics of the patients may modify the brain tissue. Thus, to better determine how “normal” the resected tissue is, it is fundamental to know in detail certain clinical, anatomical, and psychological characteristics of the patients. Unfortunately, this information is frequently not fully available for the patient from which the resected tissue has been obtained—or is not fully appreciated by the neuroscientists analyzing the brain samples, who are not necessarily experts in epilepsy.

From 1991 to 2007, we have had access to the resected brain tissue from 260 patients that were operated on at the Hospital de la Princesa in Madrid. Of these patients, for the present study, we selected 34 TLE patients (most of whom were also included in DeFelipe et al., 1993; Arellano et al., 2004; Andrioli et al., 2007; Kastanauskaite et al., 2009; Alonso-Nanclares et al., 2011; Armañanzas et al., 2013), based on the detailed clinical, anatomical and psychological information available for the same patients. One of the main aims was to further examine the possible relationships between epilepsy, neuropathology and standard cognitive and personality studies considering all of the data together.

In order to present the full picture of TLE in a way that would be accessible to multiple communities (e.g., basic researchers in neuroscience, neurologists, neurosurgeons, and psychologists), we have organized this article to include clinical pathology, surgical descriptions, histopathology, and—in particular— a detailed account of pre- and postoperative psychological testing, as applied to this population of 34 TLE patients. More specifically, we investigated the histopathological characteristics of the resected tissue (both hippocampal and extrahippocampal regions) of patients with or without sclerosis, with reference to the pre- and postoperative cognitive and personality testing. Our aim was to convey the full complexity of the disorder, its putative anatomical substrates, and the wide range of individual variability, with a view toward (1) emphasizing the importance of considering critical patient information when using brain samples for basic research and (2) gaining a better understanding of normal and abnormal brain functioning.

## Patients and methods

**FIGURE 1:**
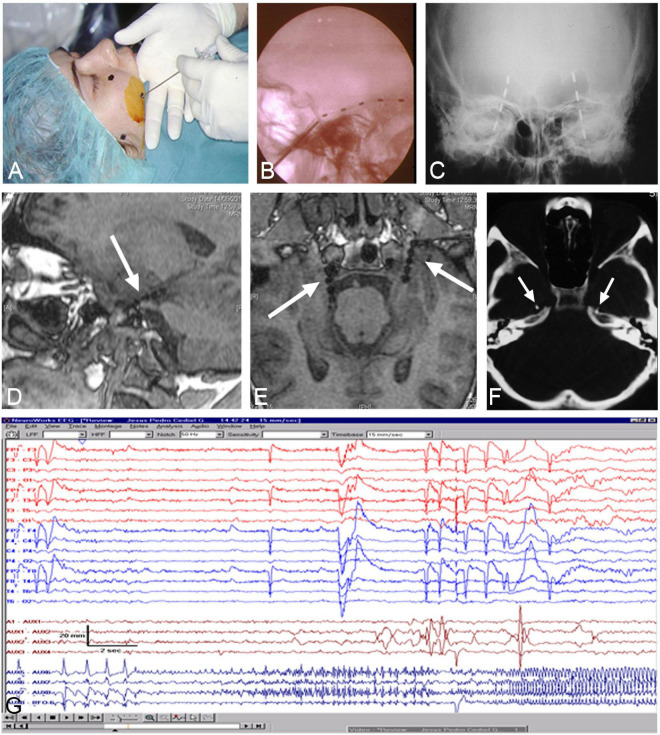
Foramen ovale electrodes (FOE). **(A)** Surgical image showing the insertion of the needle into the trigeminal (Gasser) ganglion cistern. **(B,C)** lateral and antero-posterior X-ray to assess the position of implanted FOE. **(D–F)** MRI and CT-scan to verify the position of FOE in the ambiens cistern, in contact with the uncus/parahippocampal gyrus (arrows). **(G)** Video-EEG recording revealing onset of seizures in contacts of the left FOE.

Patients were pre- and post-surgically evaluated according to the protocol used at the “Hospital de la Princesa” (Madrid, Spain), as described elsewhere (Sola, 1997; Sola et al., 2005; Pastor et al., 2010a). All patients were studied in two steps: (1) Non-invasive tests, consisting of MRI, SPECT, EEG, and neuropsychological and personality studies (Martín et al., 1992, 1994; Maestú et al., 2000) and (2) If temporal lobe epilepsy was confirmed, foramen ovale electrodes were bilaterally implanted under general anesthesia (Figures 1A–F) and subsequent Video-EEG was recorded (Figure 1G; Pastor et al., 2004, 2006, 2008a,b; Pastor and Sola, 2008; Herrera-Peco et al., 2010, 2011; Sanz-García et al., 2016).

### Surgery

**FIGURE 2:**
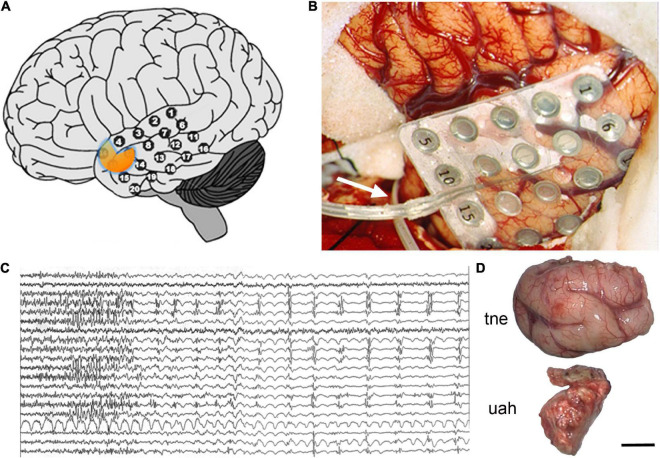
Surgical procedure. **(A,B)** 20-electrode grid over the left temporal neocortex and a strip of 8 electrodes, entering through the base of the lateral (Sylvian) sulcus [arrow in panel **(B)**], to be placed between the tentorium cerebelli and the uncus-parahippocampal gyrus. **(C)** Electrocorticographic (EcoG) recording after pharmacological stimulation with etomidate. **(D)** Image of the en-bloc resection of the temporal neocortex (tne) and the uncus-amygdala-hippocampal complex (uah). The orange area in panel **(A)** illustrates the approximate location of the amygdala and head of the hippocampus. Scale bar: 10 mm.

**FIGURE 3:**
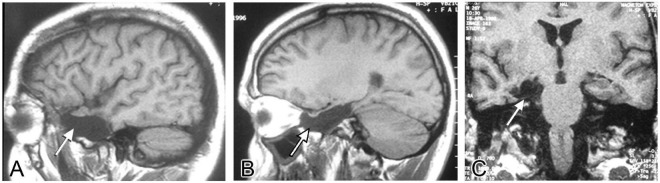
MRI control after surgery. MRI images illustrating the surgical resection of the temporal neocortex and uncus-amygdala-hippocampal complex (arrows) from the same patient. **(A,B)** Sagittal planes, **(C)** coronal plane.

If the focus of seizures was confirmed to be in the temporal lobe and only in one hemisphere, patients underwent surgery. During the procedure, the temporal cortex was exposed and a 20-electrode grid was positioned covering the anterior temporal neocortex (Figures 2A,B). Also, a 6–8 electrode strip was placed between the tentorium cerebelli and the uncus/parahippocampal gyrus (arrow in Figure 2B). Electrocorticographic recording of spontaneous activity and after pharmacological stimulation with etomidate (to induce seizure-like activity) was carried out (Ortega et al., 2008a,b; Wix et al., 2008; Pastor et al., 2010b; Vega-Zelaya et al., 2014, 2016; Figure 2C). Based on the recording results, a tailored resection of the neocortex and amygdala-hippocampal complex was performed following Spencer’s technique (Spencer et al., 1984b) in two blocks (Figure 2D): (i) a resection from the tip of the temporal lobe sparing the superior temporal gyrus, followed by (ii) removal of approximately 2/3 of the anterior portion of the hippocampus and about 95% of the amygdala. After surgery a control MRI was performed (Figure 3).

Written informed consent was obtained from all participants in accordance with the Helsinki Declaration (World Medical Association, 2013). This study and all protocols received institutional ethics approval by the ethical committee at the “Hospital de la Princesa”. From the cohort of 260 epileptic patients receiving surgical treatment at this hospital, we selected 34 temporal lobe epileptic patients (according to EEG recordings), who suffered from partial, complex and secondarily generalized seizures (Table 1).

**TABLE 1:** Summary of the clinical data of the 34 epileptic patients analyzed including prognostic pathology by MRI and surgical outcome (Engel scale).

Patient	Gender	Year of birth/date evaluation pre (age)	Side	Debut (age)	MRI features	Seizure frequency	Date evaluation post (age at surgery)	Engel scale
H1	M	1968/93(25)	R	7	Possible right mesial temporal sclerosis	Weekly	1995(27)	III
H16	F	1968/92(24)	R	14	Glioneuronal hamartoma	Daily	1992(24)	III
H21	F	1964/92(28)	L	16	Left temporal atrophy	Monthly	1993(28)	II
H27	M	1963/93(30)	R	1	Normal	Monthly	1993(30)	II
H29	M	1951/93(42)	R	4	Possible right mesial temporal sclerosis	Weekly	1994(43)	I
H31	F	1971/93(22)	R	14	Possible right mesial temporal sclerosis	Daily	1994(23)	I
H33	M	1974/93(19)	L	5	Normal	Daily	1994(20)	III
H35	F	1964/94(30)	R	7	Normal	Weekly	1994(30)	I
H36	M	1932/93(61)	L	24	Possible left mesial temporal sclerosis	Weekly	1994(62)	I
H38	M	1970/94(24)	L	16	Normal	Weekly	1995(25)	II
H40	M	1975/94(19)	R	10	Normal	Weekly	1995(20)	III
H41	F	1955/94(39)	L	12	tumor with calcification	Weekly	1995(40)	I
H44	M	1960/95(35)	L	2	Left mesial temporal sclerosis	Weekly	1995(35)	I
H48	M	1954/95(41)	L	18	Possible left mesial temporal sclerosis	Weekly	1995(41)	I
H50	M	1956/95(39)	R	17	Normal	Weekly	1996(40)	III
H57	M	1969/96(27)	R	13	Possible right mesial temporal sclerosis	Weekly	1996(27)	I
H61	F	1978/96(18)	R	7	Right mesial temporal sclerosis	Weekly	1996(18)	I
H65	F	1974/96(22)	L	14	Possible left mesial temporal sclerosis	Weekly	1996(22)	II
H67	M	1957/96(39)	R	1	Right temporal atrophy	Weekly	1996(39)	I
H69	M	1944/94(50)	L	14	Normal	Monthly	1996(52)	I
H75	M	1958/95(37)	L	13	Possible left mesial temporal sclerosis	Weekly	1997(39)	II
H80	F	1951/95(44)	L	5	Possible left mesial temporal sclerosis	Daily	1997(46)	I
H84	M	1965/96(31)	R	2	Possible right mesial temporal sclerosis	Weekly	1997(32)	I
H85	M	1968/94(26)	R	14	Bilateral parietal cortical gray-matter thinning	Weekly	1997(29)	III
H94	M	1970/96(26)	L	20	Left mesial temporal sclerosis	Weekly	1998(28)	I
H104	M	1966/98(32)	L	12	Normal	Weekly	1998(32)	I
H108	M	1948/98(50)	L	15	Left mesial temporal sclerosis	Weekly	1998(50)	III
H109	F	1976/98(22)	R	18	Diffuse atrophy of the cerebellum	Weekly	1998(22)	I
H115	F	1957/97(40)	L	2	Possible left mesial temporal sclerosis	Weekly	1998(41)	III
H123	F	1973/98(25)	L	7	Possible left mesial temporal sclerosis	Daily	1999(26)	I
H136	F	1979/99(20)	R	1	Right mesial temporal atrophy	Weekly	1999(20)	I
H138	F	1958/99(41)	L	1	Normal	Weekly	1999(41)	II
H141	M	1950/99(49)	L	15	Left mesial temporal atrophy	Monthly	1999(49)	III
H164	M	1973/96(23)	R	1	Right mesial temporal atrophy	Monthly	1999(26)	I

F, female; M, male; L, left; R, right; Engel scale for surgical outcome (Engel et al., 1993): class I seizure-free, class II rare seizures, class III worthwhile improvement. All patients except H44 and H61 were right-handed.

**FIGURE 4:**
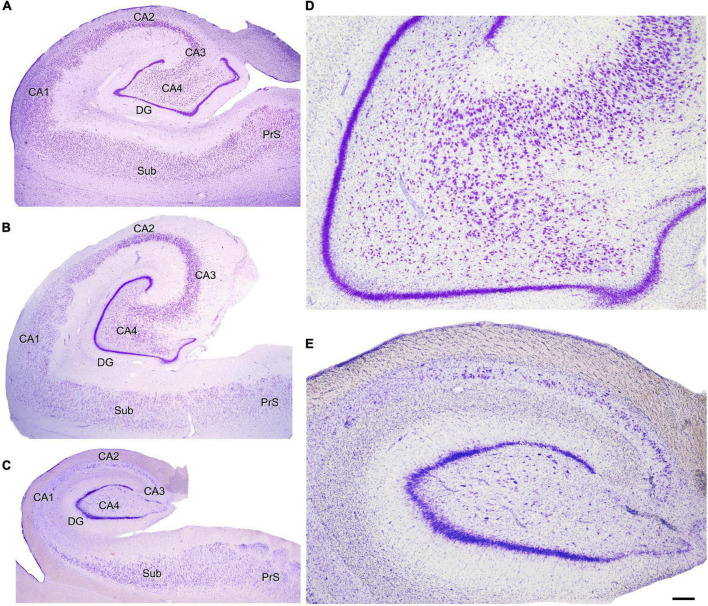
Nissl-stained hippocampal sections. **(A)** Autopsy subject (female, 42 years old). **(B)** Epileptic patient without hippocampal sclerosis or any apparent hippocampal histopathology (H65; female 22, years old). **(C)** Epileptic patient with hippocampal sclerosis (H164; male, 23 years old). Note in panel **(C)** the hippocampal atrophy and loss of neurons in all hippocampal fields with relatively preserved DG and normal-looking subicular formation. See below section “Hippocampal sclerosis.” **(D,E)** Higher magnification of panels **(B,C)**, respectively, to illustrate the normal looking histological aspect of the NoHS-patients **(D)** and the loss of neurons in the HS-patients **(E)**. Because of the atrophy of the hippocampal formation, the DG and several CA hippocampal fields can be observed in panel **(D)**, whereas in panel **(E)** only the DG and CA4 are visualized, despite both pictures having been taken at the same magnification. CA1–CA4, cornu ammonis fields; DG, dentate gyrus; Sub, subiculum; PrS, presubiculum. Scale bar shown in panel **(E)** indicates 950 μm in panels **(A–C)** and 240 μm in panels **(D,E)**. Panels **(A,B)** have been taken from Andrioli et al. (2007) and panel **(C)** from Kastanauskaite et al. (2009).

Histological analysis of the resected tissue revealed the presence of hippocampal sclerosis in 18 of them, whereas in the remaining 16 cases the hippocampal formation looked normal, not displaying histological alterations on standard histological preparations (e.g., Nissl staining) (Figure 4) or in sections immunocytochemically stained to visualize total neuronal populations (NeuN); GABAergic interneurons (GAD-1, GAT-1, PV, CB, CR, and others); or glial populations such as astroglia (GFAP) or microglia (Iba-1) (Arellano et al., 2004; Andrioli et al., 2007).

### Histopathological analysis

We classified hippocampal sclerosis based on ILAE (2013; Blümcke et al., 2013). ILAE Sclerosis type 1 refers to classic hippocampal sclerosis characterized by neuronal loss and gliosis both in CA1 and CA4-hilus, and to a variable degree in the rest of the fields. We distinguished between *Sclerosis type 1 classic*, when damage was present in CA1 with CA4 and the rest of the fields only presenting mild pathology, and *Sclerosis type 1 extensive*, when extensive loss of neurons and gliosis was found in all subfields including the DG. Cases showing an intermediate pattern of neuronal loss and gliosis will be referred to as *Sclerosis type 1 intermediate*. ILAE *Sclerosis type 2* refers to neuronal loss and gliosis predominately in CA1, and ILAE *Sclerosis type 3* exhibits predominant neuronal cell loss and gliosis in CA4-hilus (*endfolium sclerosis*).

In the pathological description, we also included the presence of alterations in the distribution of granule cells in the DG (dispersion, bi-layer pattern or patchy alterations), and the presence of corpora amylacea (spherical polyglucosan bodies with a diameter of around 10–15 μm) that are found mostly in the periventricular and subpial regions of the human brain during normal aging. Although the pathological significance of corpora amylacea is not yet clear, they are frequently described in some neurodegenerative diseases and also associated with TLE, particularly with hippocampal sclerosis (e.g., see Rohn, 2015; Riba et al., 2019, 2021).

The quantitative analysis of hippocampal histological alterations was based on Arellano et al. (2004) and included tissue from a group of epileptic patients that underwent TLE surgery, compared to tissue obtained by autopsy from three normal males (aged 23, 49, and 63 years) that died in traffic accidents (2–3 h post-mortem). Thickness of the granular layer, proliferation of mossy fibers, number of granule cells per 100 μm-wide column and neuronal densities of hippocampal subfields were quantified. As described in Arellano et al. (2004), neuronal densities were estimated using optical disectors on Nissl-stained sections by counting nucleoli (West and Gundersen, 1990). A Hitachi video camera attached to a Leitz microscope with a motorized stage were used to visualize the field on a computer screen and measure the position in the z axis. For the dentate gyrus, the number of neurons per 100 μm wide segment of the granular cell layer was assessed. The reason for estimating the number of neurons per 100 μm segment instead of neuronal density of granule cells is that most epileptic patients show granular cell dispersion that naturally will decrease the cell density, even if there is no neuronal loss. For this reason, adjacent optical dissectors (100× objective; 48 × 64 μm counting frame; 30 μm thickness) were used to count granular cell nuclei along the entire granular layer including dispersed cells. Results are shown as granule cells per 100 μm wide and 30 μm thick granular cell layer segment. Six segments were counted per patient. For the polymorphic layer of the dentate gyrus (hilus) and CA4, neuronal densities were estimated counting nucleoli in 9 optical dissectors (40× objective; 120 × 160 μm counting frame, 30 μm thickness). For fields CA3, CA2, CA1, and subiculum (this last one not reported in Arellano et al., 2004), neuronal densities were estimated by counting nucleoli in adjacent optical disectors (40× objective; 120 × 160 μm counting frame, 30 μm thickness) covering the entire stratum pyramidale from the stratum oriens border to the stratum radiatum border. Six measures were obtained per patient in CA3, CA2, and subiculum and nine measures were obtained from CA1 in each patient. Aberrant proliferation of mossy fibers was estimated by measuring the extent of the ectopic labeling of Dynorphin A in the DG molecular layer of epileptic patients. All these quantitative analyses were performed at the level of the hippocampal body (Figure 4). In addition, we used Nissl-stained sections to analyze the histopathology of resected extrahippocampal regions, including medial cortical regions adjacent to the hippocampus and lateral temporal cortex.

### Psychological assessment

The psychological parameters for each patient were evaluated through three main blocks: the brief clinical interview, the neuropsychological testing and the study of personality. The specific variables recorded in each study are summarized in Table 2 and described below.

**TABLE 2:** Set of variables selected for psychological assessments.

**BRIEF CLINICAL INTERVIEW** Aggressive behavior (AGR), Anxiety (ANX), Delusions (DEL), Depression (DEP), Disinhibition (DIS), Gambling behavior (GB), Hypochondria (HYP), Hypomanic behavior (HB), Hysteria (HYS), Impulsiveness (IMP), Manic episode (MAE), Mystical delusions (MYS), Paranoia (PAR), Psychopathic deviation (PD), Schizophreniform (SCH), Suicide attempt (SA), Suicidal Ideation (SU), Though disorder (THD)

**NEUROPSYCHOLOGICAL FEATURES** **Wechsler Adult Intelligence Scale (WAIS):** Full Scale Intellectual Quotient (FSIQ): Very Low (0–69), Low (70–79), Normal (80–119), High (120–129) Very High (>130) Digit Span (DS; digit span): Very Low (0–20), Low (21–40), Normal (41–60), High (61–79) and Very High (>80) **Wechsler Memory Scale (WMS):** Immediate logical memory (LM1): Very Low (0–20), Low (21–40), Normal (41–60), High (61–79) and Very High (>80)**** Delayed logical memory (LM2): Very Low (0–20), Low (21–40), Normal (41–60), High (61–79) and Very High (>80)**** Immediate visual memory (VM1): Very Low (0–20), Low (21–40), Normal (41–60), High (61–79) and Very High (>80)**** Delayed visual memory (VM2): Very Low (0–20), Low (21–40), Normal (41–60), High (61–79) and Very High (>80) **Rey-Osterrieth Complex Figure Test (ROCF):** ROCF Copy: Very Low (0–20), Low (21–40), Normal (41–60), High (61–79) and Very High (>80)**** ROCF Memory: Very Low (0–20), Low (21–40), Normal (41–60), High (61–79) and Very High (>80) ROCF Planning ability: Type I (good planning) and Type II (poor planning)

**PERSONALITY AND PROJECTIVE ASSESSMENT** **The Rorschach Test:** Perceptual-Thinking Index (PTI): Negative, Positive, Missing. Coping Deficit Index (CDI): Negative, Positive, Missing. Suicide Constellation (Suicide Potential: S-CON): Negative, Positive, Missing. Depression Index (DEPI): Negative, Positive, Missing. Obsessive Style Index (OBS): Negative, Positive, Missing. Hypervigilance Index (HVI): Negative, Positive, Missing. Personality Style (P. Style): Introversive (EB1), Extratensive (EB2), Ambitent (EB3). Good human response (GHR) and Poor human response (PHR): GHR > PHR, GHR < PHR, GHR = PHR. Sum of the Chromatic Responses (Sum C). Sum of the Achromatic Responses (Sum C’). Sum of Texture Responses (Sum T). Sum of Vista Responses. (Sum V). Sum of Diffuse Shading Responses (Sum Y). **The projective drawings (House-Tree-Person)** (qualitative assessment):**** Two-dimensional drawings. Distortions in body image. Simplicity. Absence of basic body parts. Presence of stranger’s elements. Transparencies. Disinhibition. Dashed lines

#### Brief clinical interview

The psychological evaluation process started with a brief clinical interview to record psychopathological symptoms (Table 2) for a preliminary diagnose that is contrasted with the team and patients’ relatives. A summary of the brief interviews before and after surgery with each patient is presented in the Supplementary material (Summary of psychological interviews).

#### Neuropsychological assessment

This assessment was primarily conducted to evaluate memory deficits that might indicate a defined and restricted unilateral origin of the seizures, a pre-requisite for successful surgical intervention in TLE. In addition, such deficits provide a detailed cognitive assessment that can be correlated with the damage observed in the resected tissue.

The neuropsychological battery includes a test of general intelligence using the Wechsler Adult Intelligence Scale (WAIS; Wechsler, 1997a, 1999), complemented with a study of attention span using the Digits Span (DS) test which is the sum of the digits in forward and backward order. To examine memory dysfunction associated with medial temporal lobe dysfunction, the individuals were assessed with the Wechsler Memory Scale (WMS; Wechsler, 1997b, 2004), specifically for logical and visual memory measurements for both immediate and delayed recalls. The output of the tests was assigned to one of five categories: very low, low, normal, high and very high (Table 2). Percentiles were reported as indicated in the manual of the WMS where scores are adjusted to a normal distribution.

The Rey-Osterrieth Complex Figure Test (ROCT; Rey, 1941, 1959; Osterrieth, 1944; Meyers and Meyers, 1995; Shin et al., 2006; Peña-Casanova et al., 2009; for a recent review see Zhang et al., 2021) was administered before and after surgery to estimate visuospatial abilities, memory, attention, planning and working memory. For this test, patients were asked to reproduce a complex figure (Supplementary Figure 1), first by copying it freehand using pencils of different colors (black, red and blue in sequential order) and then, without prior warning, to reproduce it from memory after a delay of 3 min. Final scores (Table 2) were given according to the scoring norms (Osterrieth, 1944; Rey, 1959, 2003). Patients were classified according to their overall planning ability to in the progression of the drawing and sequence of colors, placement of the figure on the paper and overall integrity of the production. Planning ability was differentiated into type I and type II. Type I (good planning) defines patients that follow the hierarchical structure of the figure (beginning with the principal organizational unit, followed by the inner and outer details, while also considering placement of the figure on the paper and the integrity of the drawing. Type II (poor planning) describes individuals lacking drawing strategy, not following the sequence and showing no consideration for the placement of the figure or the integrity of the geometrical elements) (Supplementary Figure 1).

#### Personality and projective assessment

The Exner’s Rorschach Comprehensive System was used to evaluate the cognitive, perceptive and emotional responses of patients, without interference due to language understanding or cultural variables (Meyer et al., 2002; Exner, 2001, 2003; for a recent discussion about the validity and usefulness of this test, see Ales et al. (2022) and Mihura et al. (2013). All patients were assessed to evaluate problem resolution and decision making in which emotionality and ideation are involved.

The Rorschach protocols used were those included in the Rorschach Interpretation Assistance Program (RIAP5: John E. Exner Jr., Irving B. Weiner, and PAR Staff; Psychological Assessment Resources Inc. Lutz, FL, USA). Normative data is based on a sample of 600 individuals without psychological pathologies (normal population) (Exner, 2001, 2003). The Rorschach test was performed according to the criteria described in the following variables (see Table 2):

1. Perceptual-thinking index (PTI: positive* PTI ≥ 3). This index evaluates the possible existence of perceptual and thought disorders and contains 5 variables or conditions which are related to mediational and ideational difficulties, the negative perception of human movement or the percentage of shapes that do not correspond to the inkblot sheet. The probability of the presence of perceptual and thought distortions increases as the score increases. Normative data: PTI = 5, frequency 0/600; PTI = 4, frequency 0/600; PTI = 3, frequency 1/600.
2. Coping deficit index (CDI: positive* CDI ≥ 4). This index contains 11 variables or conditions which are related to the available resources, stress tolerance and responses related to cooperation as well as aggressiveness, and responses related to affection, passive or active responses, textures, isolation and food. The probability of the presence of the disorder (social disability) increases as the score increases. Normative data: CDI = 5, frequency 2/600; CDI = 4, frequency 21/600.
3. Suicide Constellation (S-CON: positive* SCON ≥ 8). The S-CON consists of an array of 12 seemingly heterogeneous variables, each of which is reviewed against a criterion to determine if the finding is positive or negative. These variables are related to negative introspection, mixture of color and shading, egocentrism, morbid responses, cognitive effort, stimulus demands against available resources, affective lack of control, proportion of adjusted responses, white space responses, number of popular responses in the population, human responses, and number of responses given to the protocol. The probability of the presence of the disorder (risk of suicidal ideation or behavior) increases as the score increases. Normative data: S-CON positive, frequency 0/600.
4. Depression index (DEPI: positive* DEPI ≥ 5). This index includes 14 variables or conditions, each of which is tested against a criterion, ultimately yielding DEPI scores from zero to seven. These variables are related to the use of perspective, shaded color, white space, egocentrism, interest in emotional stimulation, complex responses, shading and inanimate or animal responses, affective constriction, morbid responses, intellectualization, cooperative responses and isolation. The probability of the presence of the disorder increases as the score increases. Normative data: DEPI = 7 frequency, 2/600; DEPI = 6, frequency 4/600; DEPI = 5, frequency 24/600.
5. Hypervigilance index (HVI: positive* HVI ≥ 4). This index contains eight variables or conditions which involve responses related to texture as the first condition and then cognitive effort, processing efficiency, white space, full or partial human or parahuman content responses, and clothing. The probability of the presence of the disorder (hypervigilant style) increases as the score increases. Normative data: HVI positive, frequency 18/600.
6. Obsessive Style index (OBS: positive* OBS ≥ 5). This index contains 5 variables and four conditions. These variables are concerning responses related to perceptions of small details, cognitive effort, processing efficiency, number of popular responses in the normative population and over-elaborated responses. The probability of the presence of the disorder (obsessive style) increases as the score increases. Normative data: OBS positive, frequency 8/600.
7. Personality Style (P. Style, EB) is a classification into three categories according to Exner (Exner, 2001, 2003): Introversive people (EB1) like to think things through before making decisions. They prefer to keep their emotions aside during those times and tend to delay initiating behaviors until they have had time to consider various options. Extratensive individuals (EB2) are more intuitive. They are prone to use their feelings more directly in decisions making by merging them with their thinking. Ambitents (EB3), on the other hand, do not show the consistency of either the introversive or extratensive styles in their decision making or problem solving. This classification is based on the proportion of responses featuring perception of human movement and chromatic color in a protocol with 23 responses on average. Subjects whose responses predominantly relate to human movement are classified as introversive (EB1) (for example, two people dancing, a man standing up, a person giving a hug, etc.), while subjects whose responses predominantly refer to color are classified as extratensive (EB2) (for example, intense red apples, a green butterfly, spilled blood, etc.). Ambitent (EB3) subjects are those who score roughly the same for each of the two types of responses. Personality Style (EB) contains the ratio of two variables, M:WSumC. EB1 Introversive when M > WSumC; EB2 Ambitent when M = WSumC; EB3 Extratensive when M < WSumC. The normative data are the following: EB1 Introversive, frequency 199/600; EB2 Ambitent, frequency 116/600; EB3 Extratensive, frequency 227/600. Descriptive statistic for M: Mean = 4.30, SD = 1.95, Min = 1.00, Max = 10.00, Frequency = 600, Median = 4, Mode = 3.00, Sk = 0.48, Ku = −0.55. Descriptive statistic for WSumC: Mean = 4.36, SD = 1.78, Min = 0, Max = 9.50, Frequency = 599/600, Median = 4, Mode = 3.50, Sk = 0.11, Ku = −0.54.
8. PRE-POST total index (improvement, worsening, unchanged). This refers to the changes before and after surgery that are reduced (improvement), increased (worsening) or maintained (unchanged) in the Rorschach indices.
9. The variable GHR:PHR is a ratio of two variables: Good Human Response (GHR) for individuals with effective and/or adaptive responses and Poor Human Response (PHR) for individuals with ineffective and/or non-adaptive responses. GHR < PHR is considered as a negative value. Descriptive statistics GHR: Mean = 4.93, SD = 1.78, Min = 0, Max = 10, Frequency = 598/600, Median = 5, Mode = 5, Sk = 0.36, Ku = 0.02. Descriptive statistics PHR: Mean = 1.53, SD = 1.46, Min = 0, Max = 8, Frequency = 431/600, Median = 1, Mode = 1, Sk = 1.25, Ku = 2.30.
10. The variables SumC, SumC’, SumT, SumV, SumY do not have a normal distribution. The clinical validity criteria have been made by dichotomizing the variables taking into account the clinical significance, as follows:
  - Sum of chromatic responses (SumC) is associated with emotional regulation: negative value if SumC ≤ 0. Descriptive statistics SumC: Mean = 4.36, SD = 1.78, Min = 0, Max = 9.50, Frequency = 599/600, Median = 4, Mode = 3.50, Sk = 0.11, Ku = −0.54.
  - Sum of the achromatic responses (SumC’ assesses suppression or constraint of emotion): negative value if SumC’ = 0 or SumC’ > 4. Descriptive statistics SumC’: Mean = 1.49, SD = 1.16, Min = 0, Max = 10, Frequency = 490/600, Median = 1, Mode = 1, Sk = 0.41, Ku = 5.96.
  - Sum of texture responses (SumT evaluates the needs for closeness and openness to close emotional relationships): negative value if SumT = 0 or SumT ≥ 2. Descriptive statistics SumT: Mean = 0.95, SD = 0.61, Min = 0, Max = 4, Frequency = 490/600, Median = 1, Mode = 1, Sk = 0.83, Ku = 3.33.
  - Sum of vista responses (SumV assesses introspection with negative emotions): negative value if SumV ≥ 2. Descriptive statistics Sum V: Mean = 0.28, SD = 0.61, Min = 0, Max = 5, Frequency = 124/600, Median = 0, Mode = 0, Sk = 2.71, Ku = 9.58.
  - Sum of diffuse shading responses (SumY relates to feelings that are prompted by a sense of helplessness or an inability to respond): negative value if SumY ≥ 3. Descriptive statistics Sum Y: Mean = 0.61, SD = 0.96, Min = 0, Max = 10, Frequency = 262/600, Median = 0, Mode = 0, Sk = 3.53, Ku = 23.46.

In addition, we included the House-Tree-Person (H-T-P) test, which involves motor expression using a pencil to draw these elements on paper. This is a projective technique (Buck, 1948) of great clinical interest as an indicator of cognitive and emotional dysfunctions and psychopathological traits associated with brain damage. Moreover, the H-T-P test permits the acquisition of diagnostic hypotheses and personality clues that could not be observed using other tests (DeFelipe-Oroquieta et al., 1998; DeFelipe-Oroquieta and Ortiz, 2002).

### Statistical analysis

Data analysis and statistical comparisons of psychological variables between the groups were performed using McNemar, the one-factor and Mixed-design ANOVA and Chi-square (χ^2^) tests to compare the psychological variables for pre-post comparisons. Statistical analysis and graphs were performed using the SPSS statistical package (IBM SPSS Statistics for Windows, v24.0, IBM Corp., NY, USA) and GraphPad Prism 7 statistical package (Prism, San Diego, CA, USA).

### Machine learning tools

We analyzed the patients’ histopathological and psychological data using machine learning by modeling the data as a supervised classification problem where the outcome of epilepsy surgery was the class label (patients with an Engel I versus patients with Engel II or III). The relevance of the clinical descriptors was assessed by a feature subset selection process over 10,000 random restarts (sci-kit-learn ML package). Highly ranked descriptors were discussed based on their predicted statistical influence on the surgery outcome. To this end, a wrapper feature subset selection process was coupled with a logistic regressor (L1 penalized) on a repeated hold-out schema. Features’ coefficients from the regression were filtered to find highly relevant subsets and their classification performance was estimated (Supplementary Table 5).

## Results

### Histopathological and immunocytochemical studies

When reexamining the resected brain tissue from our sample of 34 TLE patients, we focused on patients with hippocampal sclerosis (HS-patients), since patients with normal-looking hippocampus (no neuronal loss and no gliosis) (No-HS-patients) did not show any apparent structural or pathological alterations other than those indicated in Table 1.

### Histopathological findings

We did not find any type 3 sclerosis in our collection, and except for three patients classified as ILAE Sclerosis type 2, most patients (*n* = 15) were classified as ILAE Sclerosis type 1, of which *n* = 11 showed *classic* subtype, *n* = 3 showed *extensive* subtype and *n* = 1 was *intermediate* (Table 3). Detailed quantitative information for most of the patients is shown in Table 4 and Supplementary Table 1, including the thickness of the granule cell layer, extent of aberrant mossy fibers and percentages of neuronal density in all hippocampal fields and controls. As can be observed in Tables 3, 4, histological alterations were rather heterogeneous between patients. Interestingly, we found a consistent increase in neuronal density in the subiculum (except in one patient) as a consequence of the general atrophy of the hippocampal formation without significant loss of subicular neurons (Table 4). Some examples of the pathological changes found in most of the HS-patients are shown in Supplementary Figure 2.

**TABLE 3:** Histopathological findings detected in biopsy tissue from epileptic patients. Pathology includes hippocampal sclerosis, classified according to ILAE (2013; Blümcke et al., 2013) criteria; alterations in the lamination of the DG (granular cell dispersion, bi-layered, or patchy distortions) and presence of corpora amylacea deposits.

	Hippocampal formation	Extrahippocampal
**H44**	Sclerosis type 1 extensive No granule cell dispersion No corpora amylacea	No apparent alterations
**H48**	Sclerosis type 1 classic Granule cell dispersion No corpora amylacea (Supplementary Figures 2-H48-1 to 2-H48-5)	No apparent alterations
**H57**	Sclerosis type 1 classic Granule cell dispersion No corpora amylacea	No apparent alterations
**H61**	Sclerosis type 2 GC dispersion and multiple clusters of ectopic GC in the molecular layer of the DG. No corpora amylacea (Supplementary Figures 2-H61-1 to 2-H61-4)	No apparent alterations
**H67**	Sclerosis type 1 classic Granule cell dispersion Abundant corpora amylacea in CA4/hilus	Lateral temporal cortex: Focal neuronal loss in layers II (mainly) and III, and abundant deposits of corpora amylacea mostly in layers I and II. Both changes coincide in some regions (Supplementary Figures 2-H67-1 to 2-H67-2)
H75	Sclerosis type 1 classic Granule cell dispersion with regions showing bi-laminar pattern No corpora amylacea (Supplementary Figure 2-H75-1)	No apparent alterations
**H80**	Sclerosis type 1 classic No granule cell dispersion No corpora amylacea (Supplementary Figures 2-H80-1 to 2-H80-6)	No apparent alterations
**H84**	Sclerosis type 1 classic GC dispersion and multiple clusters of ectopic GC cells in the molecular layer of the DG. No corpora amylacea (Supplementary Figures 2-H84-1 to 2-H84-3)	Lateral temporal cortex: Extensive neuronal loss mainly in the bottom of layer II, and abundant corpora amylacea in layers I-III. Both changes coincide in the same regions
**H94**	Sclerosis type 1 classic Granule cell dispersion with alternate regions showing a bi-laminar pattern No corpora amylacea (Supplementary Figures 2-H94-1 to 2-H94-5)	No apparent alterations
**H104**	Sclerosis type 1 classic Granule cell dispersion No corpora amylacea (Supplementary Figures 2-H104-1 to 2-H104-4)	Lateral temporal cortex: Focal neuronal loss in layers II (mainly) and III (Supplementary Figure 2-H104-5)
H108	Sclerosis type 1 intermediate Granule cell dispersion Abundant deposits of corpora amylacea mostly in the hilus, molecular layer of the subiculum and CA1 adjacent to subiculum, as well as in the white matter of the parahippocampal cortex (Supplementary Figures 2-H108-1 to 2-H108-2)	Lateral temporal cortex: Corpora amylacea in some regions of the white matter
**H109**	Sclerosis type 2 Granule cell dispersion No corpora amylacea (Supplementary Figures 2-H109-1 to 2-H109-4)	No apparent alterations
H115	Sclerosis type 1 extensive Granule cell dispersion No corpora amylacea (Supplementary Figure 2-H115-1)	No apparent alterations
**H123**	Sclerosis type 2 Granule cell dispersion Microvascular alteration between the subiculum and presubiculum No corpora amylacea (Supplementary Figures 2-H123-1 to 2-H123-8)	Lateral temporal cortex: Focal neuronal loss in layers II (mainly) and III
**H136**	Sclerosis type 1 classic Granule cell dispersion Microvascular alteration in the entorhinal cortex and white matter No corpora amylacea (Supplementary Figures 2-H136-1 to 2-H136-13)	No apparent alterations
H138	Sclerosis type 1 classic Granule cell dispersion No corpora amylacea (Supplementary Figures 2-H138-1 to 2-H138-3)	No apparent alterations
H141	Sclerosis type 1 extensive (except granule cell layer) Granule cell dispersion No corpora amylacea	NA
**H164**	Sclerosis type 1 classic Granule cell dispersion with extensive regions showing bi-laminar pattern No corpora amylacea (Supplementary Figure 2-H164-1)	No apparent alterations

Unless otherwise specified, the analysis was performed at the level of the hippocampal body (Figure 4). Histopathological analysis of extrahippocampal regions based on Nissl-staining and NeuN immunolabeling is also provided. NA indicates data not available. Code of patients in bold indicates seizure-free after surgery. See also Table 4.

**TABLE 4:** Quantitative analysis of hippocampal histological alterations in a group of epileptic patients that underwent TLE surgery compared to normal hippocampi obtained by autopsy.

	Thickness (μm)		Neurons/column	Neuronal densities					
Patient	(mean ± SEM)		(mean ± SEM)	(neurons/mm^3^; mean ± SEM)					
	GCL	Aberrant mossy fibers	GCL	Hilus	CA4	CA3	CA2	CA1	Sub
Control	90 ± 3	0	84.5 ± 5.5	4022 ± 438	6991 ± 629	14689 ± 1173	17453 ± 1105	12414 ± 1020	8609 ± 442
**H41**	105 ± 8	60 ± 9	84.1 ± 8.9	2788 ± 372	8899 ± 529	19009 ± 1899	15465 ± 983	10812 ± 974	9488 ± 915
**H44**	117 ± 14	113 ± 11	26.8 ± 6.1***	677 ± 216***	2279 ± 538***	7312 ± 1693**#	16760 ± 1637	6971 ± 1887*#	9588 ± 671
**H48**	174 ± 15***	173 ± 11	46.6 ± 13.1*	2585 ± 698	4814 ± 1497	11573 ± 622	13789 ± 1397	4873 ± 1675***	10988 ± 824
**H57**	260 ± 19***	306 ± 37	66.7 ± 8.9	515 ± 176***	1196 ± 585***	5156 ± 954***	9591 ± 643***	4290 ± 1191***	9906 ± 569
**H61**	282 ± 39***	310 ± 67	49.7 ± 13.2	4578 ± 576	8233 ± 915	14568 ± 2119	18041 ± 1087	4648 ± 1346***	11158 ± 348
H65	108 ± 12	0	63.8 ± 10.5	3715 ± 399	6691 ± 523	15921 ± 1940	21329 ± 891	11386 ± 2136	8571 ± 665
H75	351 ± 14***	388 ± 51	83.9 ± 20.5	1440 ± 421***	2685 ± 836***	6764 ± 1062***	8415 ± 1085***	3906 ± 1310***	15086 ± 1555***
**H80**	111 ± 15	0	107.6 ± 10.5	4134 ± 704	7074 ± 635	15794 ± 3112	16845 ± 1702	4288 ± 1244***	11825 ± 510***
**H84**	335 ± 20***	198 ± 37	23.7 ± 3.9***	411 ± 163***	986 ± 381***	–	2405 ± 284***	2513 ± 726***	8798 ± 564
H85	93 ± 6	0	83.9 ± 4.8	5626 ± 461	11587 ± 1879***	16284 ± 2072	–	11924 ± 1128	9359 ± 451
**H94**	299 ± 13***	271 ± 32	75.8 ± 9.8	1495 ± 361***	1657 ± 491***	10775 ± 2822	17559 ± 1207	3479 ± 679***	8943 ± 396
**H104**	318 ± 19***	268 ± 20	56.5 ± 14	1166 ± 452***	2205 ± 362***	–	9522 ± 1149***	4145 ± 1170***	9562 ± 457
H108	337 ± 34***	198 ± 24	40.4 ± 7.4***	513 ± 176***	972 ± 233***	4027 ± 721***	7234 ± 999***	4051 ± 851***	10881 ± 396
**H109**	213 ± 23***	268 ± 27	73.2 ± 7.8	3781 ± 583	5715 ± 818	15491 ± 1474	16887 ± 2147	5651 ± 932***	10490 ± 931
H115	278 ± 18***	198 ± 26	37.8 ± 3.9***	890 ± 275***	1920 ± 702***	6281 ± 1776***	11577 ± 673**#	2866 ± 1065***	7989 ± 932
**H123**	222 ± 16***	225 ± 45	83.6 ± 1.9	1677 ± 501**	5577 ± 1237	8982 ± 1445	13764 ± 1142	4648 ± 1431***	9501 ± 756
**H136**	316 ± 15***	268 ± 37	73.2 ± 15.5	822 ± 455***	3764 ± 845*	4414 ± 416***	7905 ± 912***	3804 ± 1177***	10784 ± 674
H138	233 ± 14***	225 ± 50	68.5 ± 10	1873 ± 579*	1891 ± 982***	5886 ± 1239***	12517 ± 975*	4776 ± 1736***	13140 ± 1074***

For details of the methodology, see the histopathological analysis section. NA indicates data not available. Statistically significant differences with control values were calculated with ANOVA (one-tail) and Dunnet-C *post-hoc* comparisons: *p < 0.05; **p < 0.01; ***p < 0.005. Note that values in CA4 of H85 and in the subiculum of H80, H84, and H138 are significantly higher than in control. ^#^Indicates values and/or significance levels that have been updated from Arellano et al. (2004). Code of patients in bold indicates seizure-free after surgery. CA1–CA4, cornu ammonis fields; GCL, granular cell layer; Sub, subiculum.

### GABAergic circuits are altered in temporal lobe epilepsy

A major GABAergic input to the soma (and proximal dendrites) and to the axon initial segment of principal cells originates from large basket cells and chandelier cells, respectively. These GABAergic subpopulations have been proposed to be critically involved in epilepsy (reviewed in DeFelipe, 1999). Thus, unless otherwise specified, in this section we will focus mainly on axon terminals innervating the soma and proximal dendrites (basket formations) and, in particular, on the axon terminals of chandelier cells (Ch-terminals) that innervate the axon initial segment. In the normal hippocampal formation, GABA transporter 1 (GAT-1)- and parvalbumin (PV)-immunoreactive (-ir) Ch-terminals are identified in the granular layer of the dentate gyrus, in the stratum pyramidale of the CA fields, and in the pyramidal layer of the subicular complex.

Data on the distribution and the neurochemical characteristics of Ch-terminals and basket formations in the DG and CA fields were available from Arellano et al. (2004) for 14 patients. These terminals were labeled using immunocytochemistry for GAT-1 and for the calcium binding proteins PV and calbindin D-28k (CB). As described in Arellano et al. (2004), two subtypes of Ch-terminals were observed with regard to their size and the density of terminals that were named simple and complex. Simple Ch-terminals were made up of one or two rows of labeled boutons and were the standard type found in controls. In contrast, some epileptic patients exhibited complex (or hypertrophic) Ch-terminals, consisting of tight cylinder-like structures made up of multiple rows of boutons. The distribution and neurochemical characteristics of the Ch-terminals and basket formations are shown in Supplementary Tables 2–4.

These observations reveal a conspicuous heterogeneity in the alterations of inhibitory circuits both between patients and within the same patient. The GABAergic alterations are apparently not associated with any particular cytoarchitectonic characteristic. Within the regions of severe neuronal loss, and despite the presence of some parvalbumin-immunoreactive cell bodies and some axonal processes, parvalbumin-immunoreactive Ch-terminals and basket formations were rarely found; but parvalbumin-immunoreactive dense Ch-terminals and/or basket formations were commonly found associated with some surviving neurons at the border of regions adjacent to the areas of neuronal loss.

### Relationship between psychological, and histopathological data

#### Brief clinical interview and ratios

A significantly higher proportion of HS-patients exhibited psychopathological alterations before surgery (χ^2^ = 10.485, *p* = 0.001): HS-patients 94.4% (17/18); NoHS-patients 43.8% (7/16). After surgery, the proportion of NoHS-patients with psychopathological alterations increased (McNemar, *p* = 0.016) and no statistically significant differences were found between HS-patients and NoHS-patients (χ^2^ = 0.179, *p* = 0.672): HS-patients 92.3% (12/13); NoHS-patients 87.5% (14/16). (Table 5). Hyper-religiosity, a very rare symptom also described as “mysticism delusions” (Chan et al., 2009) was found in one patient (H84) with hippocampal sclerosis in the right temporal lobe.

**TABLE 5:** Summary of the brief clinical interview, which revealed psychopathological symptoms.

Patient	Symptoms PRE	Psychopathological condition	Symptoms POST	Psychopathological condition
***NoHS-patients***				
H1	SCH	Yes	SCH	Yes
H16	NSA	No	DEP	Yes
H21	NSA	No	HB, ANX	Yes
H27	NSA	No	DEP, AB	Yes
**H29**	DEP, SCH	Yes	DEP	Yes
**H31**	SA, DEP, PAR, IMP	Yes	DEP	Yes
H33	DEP	Yes	DEP, IMP	Yes
**H35**	NSA	No	NSA	No
**H36**	NSA	No	NSA	No
H38	NSA	No	IMP	Yes
H40	DEP, IMP	Yes	DEP	Yes
**H41**	NSA	No	HB	Yes
H50	SCH	Yes	SCH	Yes
H65	NSA	No	DIS	Yes
**H69**	PAR, DEP	Yes	DEP	Yes
H85	NSA	No	DEP	Yes
		7/16 (43.8%)		14/16 (87.5%)
***HS-patients***				
**H44**	AB, PAR	Yes	PAR	Yes
**H48**	DEP	Yes	DIS, IMP	Yes
**H57**	SCH, AB, SU, DEP, GB, DIS	Yes	SCH, DEP, GB	Yes
**H61**	SA, DEP	Yes	NA	NA
**H67**	PD, HYP	Yes	NSA	No
H75	MAE, THD	Yes	MAE, IMP, THD	Yes
**H80**	ANX, DEP	No	NA	NA
**H84**	MYS	Yes	MYS	Yes
**H94**	HIP, HYS, DEP	Yes	DEP	Yes
**H104**	ANX, PAR, SCH, DEP	Yes	NA	NA
H108	DEP, SCH, IMP	Yes	SCH	Yes
**H109**	SA, SCH	Yes	SCH, DEP	Yes
H115	SU, DEP	Yes	DEP	Yes
**H123**	PAR, SCH, IMP	Yes	DEP, PER	Yes
**H136**	PAR, IMP, DEP	Yes	DEP, IMP	Yes
H138	DEP	Yes	NA	NA
H141	DEP	Yes	DEP	Yes
**H164**	ANX	Yes	NA	NA
		17/18 (94.4%)		12/13 (92.3%)

NSA, no severe alterations; NA, not available; SCH, Schizophreniform; AGR, aggressive behavior; DEP, depression; ANX, anxiety; DIS, disinhibition; HYS, hysteria; SA, suicide attempt; SU, Suicidal ideation; PAR, paranoia; PD, psychopathic deviation; HYP, hypochondria; MAE, manic episode; HB, hypomanic behavior; THD, though disorder; MYS, mystical delusions; DEL, delusions; IMP, impulsiveness; GB, gambling behavior. Code of patients in bold indicates seizure-free after surgery.

#### Neuropsychological assessment

The evaluation of the WAIS showed no differences between HS- and NoHS-patients patients. Both groups had similar IQ but HS-patients with showed statistically significant changes, by a *t*-test analysis, in digit backwards (which measures working memory), between the pre and the post-surgery conditions (*F* = 4,57; *p* < 0.05) while the NoHS-patients did not show differences in their pre- and post-surgical performance. Improvement of working memory functions in HS-patients might result from relief of the epileptic activity; but the post-surgical results were rather variable since some patients improved and others worsened in terms of IQ (Table 6).

**TABLE 6:** Summary of neuropsychological data of NoHS-patients and HS-patients, before and after surgery.

		Before surgery											After surgery										
		IQ Test				ROCF Test			WM Scale				IQ Test				ROCF Test			WM Scale			
	Patient	IQ	DS	DF	DB	Copy	Memory	Planning ability	LM1	LM2	VM1	VM2	IQ	DS	DF	DB	Copy	Memory	Planning ability	LM1	LM2	VM1	VM2
***NoHS-patients***	H1	90	50	6	4	90	10	I	16	18	NA	NA	57	25	4	3	75	10	I	3	8	NA	NA
	H16	NA	NA	NA	NA	90	10	II	NA	NA	NA	NA	NA	NA	NA	NA	75	20	I	NA	NA	NA	NA
	H21	90	84	6	5	99	10	I	58	67	NA	NA	NA	84	5	6	90	1	I	13	14	NA	NA
	H27	NA	16	4	3	99	75	I	31	67	NA	NA	NA	16	4	3	99	80	I	24	41	NA	NA
	**H29**	NA	NA	NA	NA	99	30	I	NA	NA	NA	NA	NA	NA	NA	NA	99	80	I	NA	NA	NA	NA
	**H31**	104	63	6	4	99	40	I	73	57	NA	NA	NA	85	NA	NA	99	10	I	NA	NA	NA	NA
	H33	98	NA	NA	NA	99	90	I	NA	NA	NA	NA	NA	NA	NA	NA	99	70	I	NA	NA	NA	NA
	**H35**	94	84	6	5	10	1	II	3	24	NA	NA	99	91	NA	NA	20	1	II	31	31	NA	NA
	**H36**	NA	63	6	4	75	1	I	NA	0	NA	NA	NA	NA	NA	NA	70	1	I	NA	NA	NA	NA
	H38	115	95	6	7	90	10	I	88	88	NA	NA	134	98	6	6	99	30	I	17	40	NA	NA
	H40	110	63	5	5	99	99	I	NA	NA	NA	NA	116	63	6	4	99	80	I	84	83	NA	NA
	**H41**	81	16	5	2	90	1	I	18	24	68	12	89	84	7	4	40	1	I	18	27	68	81
	H50	110	63	6	4	90	4	I	26	24	98	96	108	84	6	5	99	25	I	64	81	18	12
	H65	110	63	7	3	99	90	I	76	27	96	99	101	84	7	4	99	70	I	22	16	70	80
	**H69**	87	16	4	3	75	10	II	1	2	NA	NA	90	25	5	3	30	1	II	6	14	NA	NA
	H85	112	95	6	7	99	90	I	85	73	NA	NA	90	63	4	6	99	90	I	90	73	90	87
***HS-patients***	**H44**	68	5	4	2	20	1	I	1	1	NA	NA	NA	NA	NA	NA	1	1	II	NA	NA	NA	NA
	**H48**	123	91	6	6	99	99	I	49	30	NA	NA	NA	91	7	5	99	90	II	43	66	NA	NA
	**H57**	110	6	6	5	99	30	I	41	41	36	31	116	50	5	4	99	30	I	8	22	31	34
	**H61**	100	63	5	5	99	60	I	15	10	NA	NA	109	63	5	5	NA	NA	NA	7	7	NA	NA
	**H67**	86	64	NA	NA	40	25	II	16	6	17	6	NA	NA	NA	NA	25	40	I	10	17	NA	NA
	H75	87	50	5	4	99	90	I	16	5	12	6	83	50	4	5	70	10	I	10	17	5	6
	**H80**	100	26	NA	NA	70	80	I	19	18	33	33	102	50	NA	NA	NA	NA	NA	19	9	35	21
	H84	97	50	5	4	99	80	II	8	21	15	14	95	50	6	3	99	99	I	21	27	19	17
	**H94**	NA	50	5	4	99	50	I	21	14	37	29	105	50	5	4	99	20	I	16	10	NA	NA
	**H104**	114	84	7	4	99	99	I	47	61	NA	NA	118	91	7	5	NA	NA	NA	10	8	NA	NA
	H108	99	NA	NA	NA	99	10	I	NA	NA	NA	NA	100	NA	NA	NA	99	10	I	NA	NA	NA	NA
	**H109**	81	25	4	4	99	20	I	NA	NA	NA	NA	88	16	4	3	40	10	II	NA	NA	NA	NA
	H115	96	50	5	4	99	1	I	25	24	NA	NA	88	63	6	4	50	1	II	24	22	59	77
	**H123**	89	98	7	7	99	10	I	17	6	36	20	108	91	6	6	99	10	I	21	16	37	37
	**H136**	92	25	4	4	99	30	I	12	23	NA	NA	NA	25	4	4	99	20	I	9	17	NA	NA
	H138	98	5	4	2	99	2	I	24	13	35	26	77	NA	NA	NA	NA	NA	NA	NA	NA	NA	NA
	H141	75	5	4	2	10	10	II	15	9	26	17	86	NA	NA	NA	70	10	II	17	NA	27	NA
	**H164**	102	84	6	5	99	10	I	25	20	37	24	106	NA	NA	NA	NA	NA	NA	41	50	9	6

Wechsler Adult Intelligence Scale (WAIS): including Full Scale Intellectual Quotient (FSIQ) score and percentiles of Digits Span test (DS; digit backward test); Rey-Osterrieth Complex Figure (ROCF) test: Test values expressed in percentiles and planning ability as Type I (good planning) and Type II (poor planning); WM Scale: Wechsler Memory Scale showing percentiles of Logical Memory (LM1, immediate logical memory; L2, delayed logical memory) and Visual Memory (VM1, immediate visual memory; VM2, delayed visual memory). NA, data not available. VL (Very Low, 0–20), L (Low, 21–40), N (Normal, 41–60), H (High, 61–79) and VH (Very High, >80). In the case of IQ VL (Very Low, 0–69), L (Low, 70–89), N (Normal, 90–119), H (High, 120–129), and VH (Very High, >130). Code of patients in bold indicates seizure-free after surgery.

**FIGURE 5:**
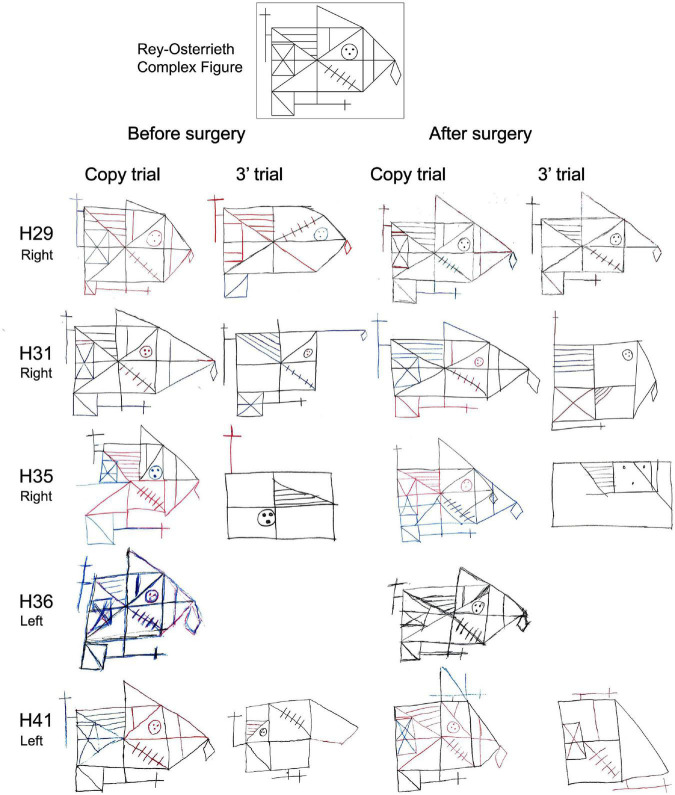
Before and after surgery ROCF drawings (copy and after 3′ trials) from NoHS-patients that became seizure-free after surgery. All patients were right-handed. Patient H36 did not made the 3′ trial because, he said, “*I don’t remember*”. The size of the original drawings has not been considered.

**FIGURE 6:**
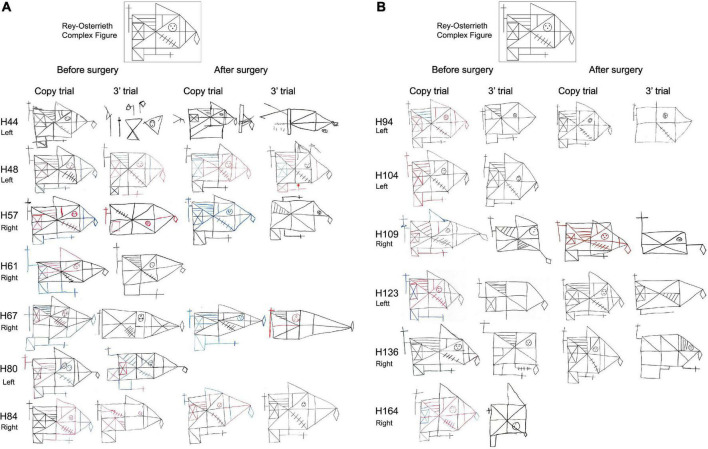
**(A)** Before and after surgery ROCF drawings (copy and after 3 min trials) from HS-patients that became seizure-free after surgery. All patients except H44 y and H61 were right-handed. Patient H61 was not evaluated after surgery. Patient H80 did not perform the tests after surgery. The size of the original drawings has not been considered. **(B)** Before and after surgery ROCF drawings (copy and after 3 min trials) from HS-patients that became seizure-free after surgery. All patients were right-handed. Patient H104 and H164 were not evaluated after surgery. The size of the original drawings has not been considered.

Regarding the evaluation of the Rey-Osterreith complex figure (ROCF), there were no significant differences between HS-patients and NoHS-patients (copy F = 0.629, *p* = 0.435; memory F = 0.001, *p* = 0.974) when comparing either pre- or post-surgery (copy F = 3.638, *p* = 0.067; memory F = 2.914, *p* = 0.099). For the direct copy scores, most of the epileptic patients showed high or very high values before surgery (15/16 NoHS-patients; 15/18 HS-patients) and low or very low values in the copy from memory (11/16 NoHS-patients; 11/18 HS-patients) (Table 6). After surgery, some patients did not show changes in the direct copy test (10/16 NoHS-patients; 7/13 HS-patients), some worsened (5/16 NoHS-patients; 5/13 HS-patients) with very few improving (1/16 NoHS-patients; 1/13 HS-patients). In the memory test, some patients also did not show changes after surgery (low values) (5/16 NoHS-patients; 6/13 HS-patients), some worsened (7/16 NoHS-patients; 5/13 HS-patients) with a few improving (4/16 NoHS-patients; 2/13 HS-patients). Concerning the planning ability of the drawing (type I and type II), no significant differences were observed between HS-patients and NoHS-patients (*F* = 1.420, *p* = 0.244), when comparing either pre- or post-surgery (*F* = 0.244, *p* = 0.626), although type II (poor planning) was more common in the HS-patients after surgery (2/16 NoHS-patients; 5/13 HS-patients). Examples of the ROCF test from patients that became seizure-free after surgery are shown in Figures 5, 6A,B, while examples from all patients can be found in the (Supplementary Figures 3, 4 for NoHS-patients and HS-patients, respectively).

As can be seen in Figures 5, 6A,B (see also Supplementary Figures 3, 4), some NoHS-patients and HS-patients showed large distortions and large clinical or idiosyncratic casuistic differences before and/or after surgery in the memory copy after 3 min trial but not in the copy trial. For example, patient H36 did not remember the figure before or after surgery (Figure 5), and patient H44 (Figure 6A) showed severe distortions in the delayed figure condition after surgery.

Both HS-patients and NoHS-patients showed impairment in their declarative memory scores. Significant differences were found in episodic memory between NoHS-patients and HS-patients patients, with the latter being worse in the WMS (logical and visual memory; *p* < 0.0002; Table 6). However, no differences were observed before and after surgery.

#### Personality and projective assessment

Regarding the information obtained from the Rorschach test, there were no significant differences between NoHS-patients and HS-patients when comparing pre- and post-surgery for some of the variables analyzed (CDI, SCON, DEPI, EB, SumC, SumY and SumT; Mixed-design ANOVA, all comparisons *p* > 0.05) (Table 7). However, some significant differences were found for some of the variables analyzed as follows. Before surgery, NoHS-patients displayed a higher SumC’ than sclerotic individuals (*p* = 0.047).^1^ After surgery, NoHS-patients showed a significant decrease in the PTI (*p* = 0.025)^2^ and in the PHR > GHR (F = 7.066, p = 0.013).^3^ After surgery, both NoHS-patients and HS-patients showed a significant increase in the OBS (*F* = 31.243, *p* = 0.000)^4^ and in the HVI (p = 0.000),^5^ but a significant decrease in the SumV (*F* = 10.44, *p* = 0.003)^6^. Furthermore, as shown in Table 7, there were changes in the Personality Style after surgery in some NoHS-patients and HS-patients (6/16 and 5/13, respectively). In addition, in the NoHS-patients group. Rorschach test was improved after surgery in 5 patients, worsened in 7 and remained unchanged in 4. In the case of HS-patients, this test was improved after surgery in 8 patients, worsened in 3 and unchanged in 2.

**TABLE 7:** Summary of Rorschach data of NoHS-patients and HS-patients, before and after surgery.

	Patient	Before surgery							After surgery							
		PTI	CDI	SCON	DEPI	HVI	OBS	EB	PTI	CDI	SCON	DEPI	HVI	OBS	EB	PRE-POST
***NoHS-patients***	H1	0	3	4	5*	0	0	EB1	2	3	4	2	1	0	EB1	Improvement
	H16	1	5*	5	4	0	0	EB2	0	5*	7	5*	2	1	EB2	Worsens
	H21	1	5*	8*	6*	0	0	EB3	2	4*	9*	5*	1	0	EB3	Unchanged
	H27	2	4*	9*	7*	0	0	EB2	0	3	5	6*	1	1	EB2	Improvement
	**H29**	4*	1	7	5*	0	0	EB2	2	3	9*	7*	4	1	EB3*	Worsens
	**H31**	0	2	4	4	0	0	EB3	0	3	7	5*	4	3	EB2*	Worsens
	H33	1	4*	6	5*	0	0	EB2	0	4*	6	5*	2	1	EB2	Unchanged
	**H35**	2	3	5	4	0	0	EB2	2	4*	5	3	2	0	EB2	Worsens
	**H36**	1	3	3	3	0	0	EB1	0	4*	4	2	2	1	EB2*	Worsens
	H38	0	4*	7	5*	0	0	EB3	0	5*	5	5*	2	1	EB2*	Unchanged
	H40	2	4*	8*	5*	0	0	EB2	0	4*	7	5*	4	1	EB1*	Improvement
	**H41**	1	3	4	3	0	0	EB2	2	2	3	4	2	1	EB2	Unchanged
	H50	4*	4*	6	5*	0	0	EB1	3*	4*	8*	5*	3	3	EB1	Worsens
	H65	3*	2	6	6*	1	0	EB1	0	1	4	4	2	0	EB2*	Improvement
	**H69**	4*	4*	7	4	0	0	EB2	0	4*	5	3	3	1	EB2	Improvement
	H85	2	4*	6	4	0	0	EB2	2	5*	5	5*	1	1	EB2	Worsens
***HS-patients***	**H44**	1	5*	5	3	0	0	EB2	2	4*	5	3	2	0	EB2	Unchanged
	**H48**	2	1	6	5*	0	0	EB2	1	3	3	4	4	2	EB1*	Improvement
	**H57**	0	3	5	5*	0	0	EB2	0	5*	5	3	3	1	EB2	Worsens
	**H61**	1	4*	4	4	0	0	EB1	NA	NA	NA	NA	NA	NA	NA	NA
	**H67**	1	4*	4	4	0	0	EB2	0	2	3	1	1	2	EB2	Improvement
	H75	1	1	4	3	0	0	EB1	1	2	3	3	3	1	EB1	Unchanged
	**H80**	0	4*	4	3	0	0	EB2	NA	NA	NA	NA	NA	NA	NA	NA
	**H84**	3*	5*	6	5*	0	0	EB3	0	4*	5	5*	3	1	EB2*	Improvement
	**H94**	0	4*	6	6*	0	0	EB2	0	3	6	5*	4	1	EB3*	Improvement
	**H104**	0	3	3	4	0	0	EB1	NA	NA	NA	NA	NA	NA	NA	NA
	H108	3*	3	7	5*	0	0	EB3	2	4*	4	4	1	0	EB2*	Improvement
	**H109**	3*	4*	5	4	0	0	EB2	2	4*	6	5*	2	0	EB2	Improvement
	H115	0	5*	7	6*	0	0	EB3	0	3	5	2	1	0	EB3	Improvement
	**H123**	1	3	4	4	0	0	EB1	1	3	7	4	5*	3	EB1	Worsens
	**H136**	0	2	4	5*	1	0	EB1	0	3	8*	6*	2	0	EB2*	Worsens
	H138	0	5*	5	5*	0	0	EB2	NA	NA	NA	NA	NA	NA	NA	NA
	H141	0	4*	7	5*	0	0	EB2	1	3	5	5*	1	1	EB3*	Improvement
	H164	2	4*	6	4	0	0	EB2	NA	NA	NA	NA	NA	NA	NA	NA

	**Patient**		**Before surgery**							**After surgery**						
			**GHR:PHR**	**SUMC**	**SUMC‘**	**SUMT**	**SUMV**			**SUMY**	**GHR:PHR**	**SUMC**	**SUMC‘**	**SUMT**	**SUMV**	**SUMY**

***NoHS-patients***	H1		2:3*	0*	3	1	1			0	1:3*	0*	2	0*	0	0
	H16		0:4*	2.5	4*	2*	0			1	1:3*	2.5	3	2*	0	1
	H21		0:0	4.5	5*	1	1			0	0:0	4	5*	0*	0	1
	H27		0:6*	3	15*	1	4*			9*	3:3	4.5	4*	1	1	2
	**H29**		3:9*	7.5	2	1	0			3*	2:7*	7.5	5*	1	1	4*
	**H31**		5:4	5	3	0*	0			1	6:2	4.5	5*	1	1	3*
	H33		1:4*	1.5	3	1	0			2	2:1	1.0	3	0*	0	1
	**H35**		2:3*	3	3	0*	0			0	0:0	0*	1	0*	0	0
	**H36**		2:5*	0*	2	0*	0			0	0:0	1.5	0*	0*	0	0
	H38		2:4*	6	7*	1	2*			1	3:2	4	5*	0*	2*	2
	H40		1:5*	4	3	0*	2*			3*	3:9*	2	4*	0*	0	0
	**H41**		0:7*	3.5	7*	0*	0			4*	0:6*	4	2	3*	1	0
	H50		4:14*	2	7*	1	2*			0	0:5*	1.5	1	1	1	0
	H65		4:5*	3	3	0*	2*			1	4:2	4.5	5*	0*	0	2
	**H69**		2:6*	2.5	4*	1	3*			0	2:5*	0.5*	1	0*	0	1
	H85		1:2*	3	1	1	0			0	1:1	1.5	0*	1	0	0
***HS-patients***	**H44**		0:0	0*	0*	0*	0			0	0:0	0.5*	1	0*	0	0
	**H48**		1:4*	3.5	3	1	1			0	4:4	1	2	0*	0	2
	**H57**		2:0	3	0*	0*	3*			0	2:3*	1.5	0*	0*	0	1
	**H61**		4:8*	0.5*	2	1	0			2	NA	NA	NA	NA	NA	NA
	**H67**		1:1	0.5*	1	0*	1			0	2:1	0.5*	0*	0*	0	0
	H75		3:0	1	0*	0*	0			0	7:2	0.5*	1	0*	0	0
	**H80**		2:2	1.5	2	1	1			2	NA	NA	NA	NA	NA	NA
	**H84**		0:3*	3	1	2*	1			1	4:2	2	4*	2*	0	1
	**H94**		2:0	2.5	3	0*	0			3*	2:2	4	3	3*	0	3*
	**H104**		7:8*	0*	2	0*	1			5*	NA	NA	NA	NA	NA	NA
	H108		0:1*	5.5	1	0*	2*			0	1:2*	1.5	1	1	0	0
	**H109**		0:0	1	1	0*	0			0	0:2*	1	2	0*	0	0
	H115		1:0	4.5	3	0*	1			0	1:0	5.5	0*	0*	0	0
	**H123**		4:3	1.5	2	0*	1			1	4:8*	2	1	0*	1	0
	**H136**		4:3	0*	0*	0*	2*			1	2:2	1.5	1	1	1	0
	H138		1:0	0.5*	6*	1	0			0	NA	NA	NA	NA	NA	NA
	H141		0:0	1	1	1	0			2	0:1*	5.5	2	1	0	0
	**H164**		1:1	0.5*	2	0*	0			0	NA	NA	NA	NA	NA	NA

Normative data is based on a sample of 600 individuals without psychological pathologies (normal population) (Exner, 2001, 2003). Negative values from the psychological point of view are indicated with an asterisk (*). Code of patients in bold indicates seizure-free after surgery. NA, data not available.

**FIGURE 7:**
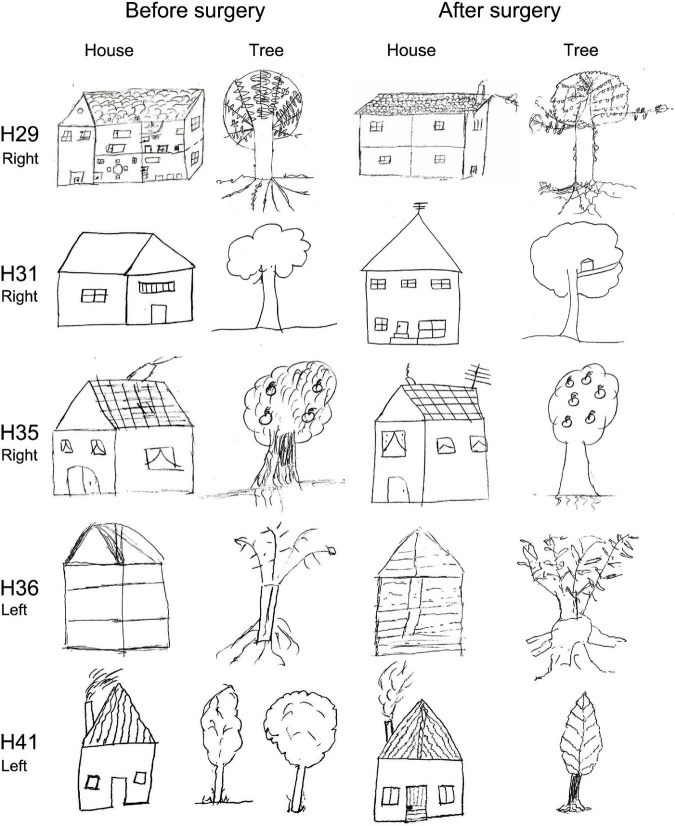
House and tree drawings of the H-T-P test (before and after surgery) performed by NoHS-patients that became seizure-free after surgery. All patients were right-handed. Note transparencies (H29; house pre surgery and H29, H35, and H36 tree before and after surgery), perspective changes (H29 house after surgery) and paranoid elements (H31, H35 house after surgery). The size of the original drawings was not considered.

**FIGURE 8:**
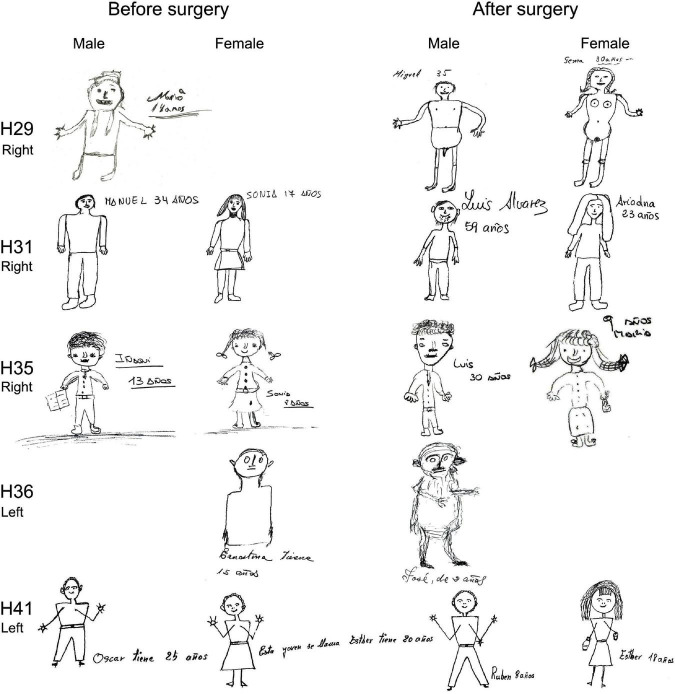
Person (male, female) drawings of the H-T-P test (before and after surgery) performed by NoHS-patients that became seizure-free after surgery. All patients were right-handed. Patients were asked to draw both genders. Patient H29 only performed one drawing; when asked for the other gender, he changed the name (Mario to Maria). After surgery this patient showed disinhibition (nude male and female). H36 had difficulty to draw and only did one. All patients had difficulties to perform the drawings except H31. The size of the original drawings was not considered.

**FIGURE 9:**
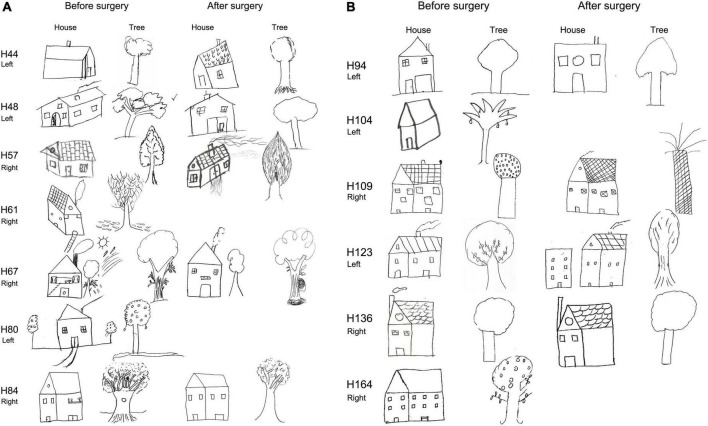
**(A)** House and tree drawings of the H-T-P test (before and after surgery) performed by HS-patients that became seizure-free patients after surgery. All patients except H44 and H61 were right-handed. Patients H61 and H80 were not evaluated after surgery. Note rotations (H44, house after surgery) or strange elements (H67; house before surgery; tree before and after surgery) or transparencies (H44 tree after surgery; H84 tree before surgery). The size of the original drawings was not considered. **(B)** House and tree drawings of the H-T-P test (before and after surgery) performed by HS-patients that became seizure-free after surgery. All patients were right-handed. Note bi-dimensionality in most patients when drawing the house. Patients H104 and H164 were not evaluated after surgery. The size of the original drawings was not considered.

**FIGURE 10:**
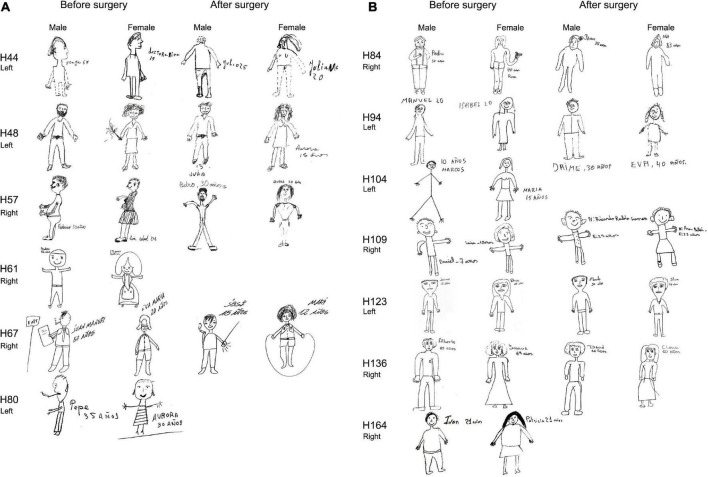
**(A)** Person (male, female) drawings of the H-T-P test (before and after surgery) performed by HS-patients rendered seizure-free after surgery. Patients were asked to draw both genders. All patients except H44 and H61 were right-handed. Patients H61 and H80 were not evaluated after surgery. Note dashed lines (H44, male before surgery and female after surgery) and transparencies (H44, female after surgery; H57, female before surgery). The size of the original drawings was not considered. **(B)** Person (male, female) drawings of the H-T-P test (before and after surgery) performed by HS-patients rendered seizure-free after surgery. Patients were asked to draw both genders. All patients were right-handed. Patients H104 and H164 were not evaluated after surgery. Note simplicity but not significant changes after surgery. The size of the original drawings was not considered.

Finally, although the analysis of the house-tree-person (H-T-P) drawings is based on clinical qualitative interpretation (Table 2), the majority of patients had difficulties with the drawings, both before and after surgery. Examples of H-T-P test from patients that became seizure-free after surgery are shown in Figures 7–10, while examples from all patients can be found in the (Supplementary Figures 3, 4 for NoHS-patients and HS-patients, respectively). As can be appreciated in Figures 7–10, the patients’ drawings showed a variety of features such as two-dimensional figures, distortion of the body image, simplicity or primitivism, absence of basic body parts, presence of strange elements (dashed lines), transparencies, disinhibition or rotations in space which are usually observed in patients with cognitive dysfunction associated to brain damage (DeFelipe-Oroquieta et al., 1998). Note that these alterations are even more pronounced after surgical intervention.

### Machine learning data analysis

**FIGURE 11:**
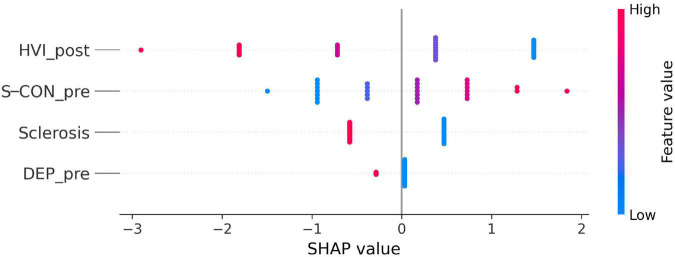
SHAP influence coefficients computed out of a logistic regressor to predict surgical outcome including only the four highest relevant features (see Supplementary Table 5): Hypervigilance Index (HVI) after surgery, Suicide Constellation (S-CON) before surgery, presence of hippocampal sclerosis (Sclerosis), and Depression (DEP_pre) from the clinical interview before surgery. Each dot represents one patient. Negative coefficient correlates with the negative class (Engel I), whereas features with a positive coefficient correlates with the positive class (Engel II or III). This model’s estimated performance is of AUC (Area Under the ROC curve) = 0.8891 (accuracy at 0.85), sensitivity = 0.8653, and specificity = 0.8344.

We created a supervised classification dataset that used the Engel scale for surgical outcome as the classification class (Engel et al., 1993). Patients with an Engel I were encoded as the negative class, or class 0, and patients with Engel II or III were grouped together as the positive class, or class 1. All other clinical information including histopathology and psychological data was translated into 56 features for a total of 29 patients for whom we had enough information to perform this analysis. Features, average coefficients, and SHAP (SHapley Additive exPlanations)^7^ values for the highest ranked are included in the (Supplementary Table 5). In Figure 11, we sorted the features by the sum of their SHAP value magnitudes for all 29 patients. This figure presents the distribution of the influence each feature has on the model output, in this case the surgery output reflected by the Engel scale. The color represents the feature value (red high, blue low). This reveals, for example, that a high value for Hypervigilance Index (HVI) after surgery strongly influences the model towards an Engel I. Conversely, a high value for Suicide Constellation (S-CON) before surgery positively influences the model to an Engel II or III outcome.

## Discussion

In agreement with a large number of previous reports, this study (1) reinforces the notion that there is substantial individual variability among epileptic patients at the histopathological and psychological levels and (2) highlights the common but overlooked psychopathological alterations that occur even in patients who become “seizure-free” after surgery. The first point is based on detailed histopathological analysis of surgical samples revealing normal-looking hippocampus in some cases and different types and degrees of hippocampal sclerosis in others, combined with pre- and post-surgical psychological evaluations. The second emerges from our extensive battery of personality and projective assessments, in a two-way comparison of HS-patients and NoHS-patients with regard to pre- and post-surgical performance.

Importantly, these correlative studies, however, are based on statistical differences between group means, which may overlook individual aspects that could help understand the relationship between neuroanatomical and psychological features. For example, from what we know of hippocampal connectivity and its role in memory, it is difficult to understand, when considering patients individually, why before surgery HS-patients and NoHS-patients may have similar psychological presentations (either normal or impaired), and why some patients improve while others do not change or worsen after surgery. Further discussion of these questions is organized below in seven sections, the first being a brief overview of TLE, surgery, and psychology. This is followed by a summary and interpretation of our results, specifically as concerns predictive value for surgical outcomes, including by the relatively novel machine learning approaches. Three further sections address improvement in cognitive functions after epilepsy surgery, interindividual variability, and problems in comparing across patients. In addition, we briefly review the entorhinal-hippocampal connections to emphasize the difficulties in interpreting psychological changes based on connectivity (and see Boxes 4, 5); as well as the hippocampal-frontal connections, hippocampal-frontal connections, especially in relation to the psychological results reported here.

**BOX 4 General connectivity of the hippocampal region.**

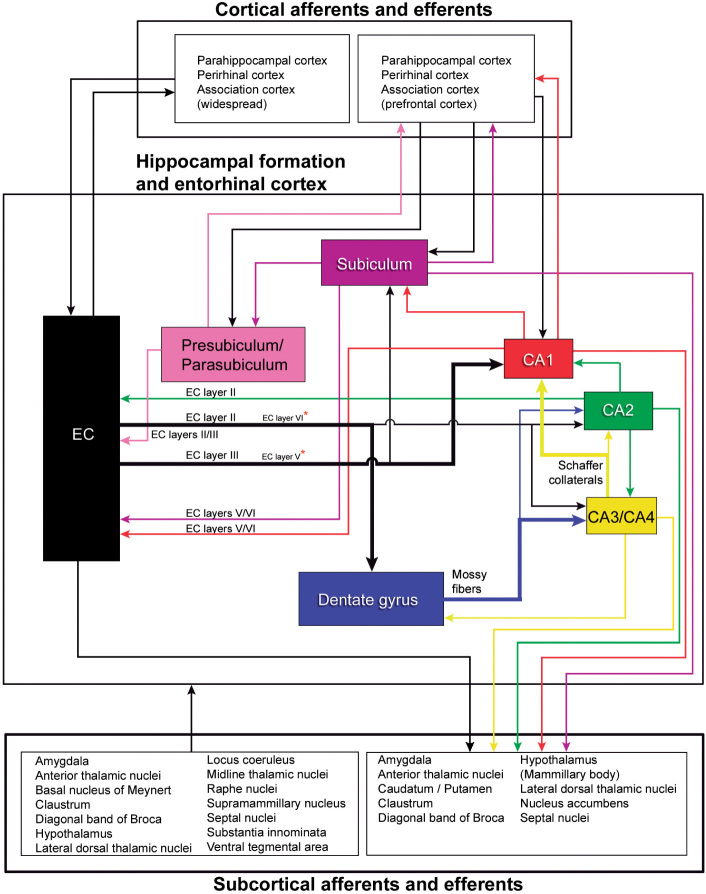

This scheme is mostly based on studies performed in rats, cats, monkeys and —most recently— in mice: Amaral and Cowan (1980), Mesulam et al. (1983), Amaral and Witter (1989), Witter and Amaral (1991), Gloor (1997), Witter et al. (2000), Kondo et al. (2009), Insausti and Amaral (2012), Rowland et al. (2013), Dudek et al. (2016), Liu et al. (2018), Marks et al. (2021), Witter and Amaral (2021). As previously discussed (e.g., Insausti and Amaral, 2012), although similar connectivity appears to exist in all species studied, there are also species-specific variations. Thus, we can only assume that the general pattern of connections of the human hippocampal formation is similar to that described in experimental animals, especially nonhuman primates, but we cannot rule out the possibility of marked differences. For example, in the monkey, only the most rostral part of the hippocampus is connected by commissural fibers from the subiculum to the contralateral entorhinal cortex (Amaral et al., 1984; Rockland and Van Hoesen, 1999; Insausti and Amaral, 2012). In humans, commissural connections seem to be even more reduced (Insausti and Amaral, 2012). Recently, Ben-Simon et al. (2022) reported that excitatory neurons in layer 6b of the mouse EC project to all sub-regions of the hippocampal formation and receive input from the CA1, thalamus and claustrum. These connections are not included in this schematic figure. Asterisks, minor projections.

**BOX 5 Main direct connections between CA1 and other brain regions in primates. Photomicrograph of a Nissl-stained coronal brain section from the human CA1 to illustrate the hippocampal layers.**

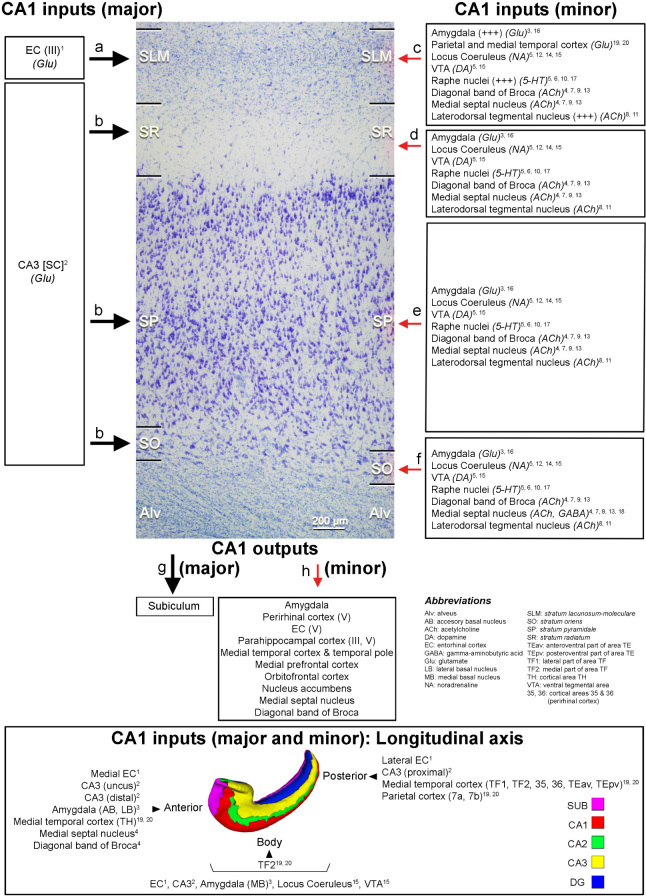

Major and minor projections have been represented with large and small arrows, respectively. Modified from Montero-Crespo et al. (2020). The 3D reconstruction of the hippocampus at the bottom of the diagram has been modified from Adler et al. (2018). Alv: alveus; SLM: stratum lacunosum-moleculare; SO: stratum oriens; SP: stratum pyramidale, SR: stratum radiatum. References: ^1^ Witter and Amaral, 1991; ^2^ Kondo et al., 2009; ^3^ Aggleton, 1986; ^4^ Alonso and Amaral, 1995; ^5^ Amaral and Cowan, 1980; ^6^ Barone et al., 1994; ^7^ De Lacalle et al., 1994; ^8^ Del Fiacco et al., 1987; ^9^ Green and Mesulam, 1988; ^10^ Ihara et al., 1988; ^11^ Iritani et al., 1989; ^12^ Klimek et al., 1999; ^13^ Mesulam et al., 1983; ^14^ Powers et al., 1988; ^15^ Samson et al., 1990; ^16^ Wang and Barbas, 2018; ^17^ Wilson and Molliver, 1991; ^18^ Gulyás et al., 1991; ^19^ Rockland and Van Hoesen, 1999; ^20^ Yukie, 2000. In rodents, it has been described a connection between layer II of the EC and the stratum lacunosum (SL) in CA1 (reviewed in Marks et al., 2021). Monosynaptic long-range GABAergic projections from frontal cortex to CA1 have been described in the mouse (Malik et al., 2022).

### Brief background on temporal lobe epilepsy, surgery, and psychology

There is a very extensive bibliography dealing with TLE, surgery, and psychology. What follows is not intended to be complete, and the unavoidable limitations in the selection of data and their interpretation reveal, in many cases, the personal views and interests of the authors.

The psychological studies performed by Scoville and Milner (1957) in patients with schizophrenia or epilepsy submitted to bilateral medial temporal lobe resection (including the well-known case patient H. M.) are of great interest not only from the surgical point of view but also for better understanding memory and cognitive changes that occur after surgery. In turn, these studies helped to provide important clues of how these parts of the brain are involved in memory and cognition (Squire, 2009b). The surgical procedure consisted in either a limited removal of the uncus and amygdala (uncotomy) or a larger resection which, in general, also included the anterior two thirds of the hippocampus and the parahippocampal gyrus and, in some cases, the temporal pole (the anterior end of the temporal lobe). When the hippocampus and parahippocampal gyrus were removed bilaterally, Scoville and Milner found a persistent impairment of recent memory, which seemed to be more pronounced depending on the extent of hippocampal removal. Memory loss included both anterograde and some retrograde amnesia, but left early memories and motor skills intact, and no deterioration in personality or general intelligence was observed. Milner and Penfield (1955) reported lack of permanent memory defects after unilateral removal of hippocampal and parahippocampal tissue in more than 90 cases of temporal lobe epilepsy. However, they also discussed two cases of unilateral partial temporal lobe resections in the dominant hemisphere (left) which resulted in an unusual severe loss of recent memory. After surgery, the general intelligence of these two patients was unimpaired and they continued with their jobs, but memory of daily events was very seriously damaged. That is, the surgical outcome was similar to those patients that had bilateral medial temporal lobe resections. Milner and Penfield concluded that in these two cases there was a severe lesion in the hippocampal region in the opposite hemisphere (right) that went unnoticed preoperatively. Thus, the unilateral removal of the hippocampal region in these two patients resulted in effect in loss of bilateral hippocampal function leading to impaired memory.

In general, deficient verbal memory is a common finding in left TLE patients indicating the involvement of the left hippocampus. In contrast, correlation between low visual memory scores and right hippocampal neuron loss or right temporal lobe surgery is not consistent between studies (e.g., Rausch and Babb, 1993; Wyler et al., 1995; Jones-Gotman et al., 1997; Shin et al., 2009). A particularly relevant study in this regard is the research performed by Jones-Gotman et al. (1997). These authors examined the general differential psychological outcome after the resection of the language-dominant left temporal lobe or the right temporal lobe, and compared the outcomes with different surgical procedures. They distinguished three groups of patients from different institutions that had undergone different types of surgical procedures: (1) anterior temporal-lobe resection including amygdala and the anterior part of the hippocampus; (2) temporal neocorticectomy, removing the neocortex and leaving intact the amygdala and hippocampus; and (3) selective resection of the medial temporal region, leaving intact the temporal neocortex. This comparison represents a good opportunity to study the involvement of the amygdala, hippocampus and temporal neocortex in learning and memory for verbal and visuospatial material. These authors found that learning and retention of words were impaired regardless of the structures removed from the left temporal lobe. However, they were unimpaired in patients submitted to right temporal resections. Learning for abstract designs was impaired in the right temporal lobe resections, but some less accentuated deficits were also observed on some trials in left-resection groups. A possible explanation given by Jones-Gotman et al. of these unexpected results is that the resection of either the hippocampal region or the temporal neocortex produces a disconnection of a circuit that is critical for the normal storage and retrieval of information. As shown in Boxes 4, 5, this conclusion is supported by the complex network of connections involved in the dialogue of the hippocampal formation with neocortical areas.

### Interpretation of our results

The main findings are six-fold when comparing TLE patients with or without hippocampal sclerosis (HS-patients and NoHS-patients, respectively). First, a significantly higher percentage of psychopathological alterations appears in HS-patients before surgery (HS-patients 94.4%; NoHS-patients 43.8%). After surgery, no statistically significant differences were found between groups but the proportion of NoHS-patients with psychopathological alterations increased. Second, the evaluation of the WAIS showed no differences between HS-patients and NoHS-patients; both groups had similar FSIQ and Digit Span values, except HS-patients that showed a significant lower performance on digit backward test. After surgery, the results were rather variable since some patients improved and others worsened in both the IQ and in Digit Span values or they showed changes in only one of these two tests. Third, the evaluation of the ROCF showed no significant differences between HS-patients and NoHS-patients when comparing either pre- or post-surgery. Most of the epileptic patients displayed high or very high values in the direct copy scores, and low or very low in the memory scores. Differences were not found to be statistically significant when comparing pre- and post-surgical conditions, although type II (poor planning) was more common in the HS-patients after surgery. From a clinical point of view, some HS-patients and NoHS-patients showed large drawing distortion and clinical or idiosyncratic casuistic differences before and/or after surgery. Fourth, although all patients showed deficits in episodic memory, neuropsychological testing revealed significant differences between NoHS-patients and HS-patients, the latter being worse in the logical and visual memory test (WMS). However, no differences were observed when comparing before and after surgery conditions. Fifth, the Rorschach test revealed significant differences between HS-patients and NoHS-patients with regard to some of the 10 variables analyzed. Before surgery, NoHS-patients displayed a higher suppression and constraint of emotion (SumC’). In all patients, the surgery intervention produced certain psychological changes: an increase in obsessive (OBS) and hypervigilant style (HVI) and a reduction in negative introspection (SumV). After surgery, NoHS-patients showed a significant decrease in the perceptual and thought disorder (PTI), as well as in the negative, ineffective and/or non-adaptive responses related to human relationships (PHR > GHR). Changes in personality style were observed after surgery in some HS-patients and NoHS-patients, with some of these changes being positive while others were negative. Sixth, the House-Tree-Person projective analysis revealed that most patients (both HS- and NoHS patients) showed affective, cognitive and motor difficulties when completing the drawings, both before and after surgery.

Despite the limited number of patients examined, the multidisciplinary combination of pathological condition (sclerotic condition), clinical interview, psychological test and machine learning tools together provide an important improvement in the criteria for selecting candidates for epilepsy surgery and to predict the outcome after surgery. We found machine learning approaches a significant aid in predicting the outcome of epilepsy surgery. Based on supervised classification data mining (as discussed in Armañanzas et al., 2013) machine learning protocols can easily take into account clinical variables as well as pathological and psychological evaluations. In the present study, the SHAP influence coefficients (see Figure 11) indicate that Hypervigilance Index (HVI) after surgery, Suicide Constellation (S-CON) before surgery, Detection of sclerotic tissue (Sclerosis), and Depression (DEP) from the clinical interview before surgery were the more relevant features to predict the outcome. We found that a high value for HVI after surgery as well as the presence of hippocampal sclerosis (Sclerosis) strongly influences the model towards an Engel I. DEPI item also influenced the model towards an Engel I, but with less impact than HVI and Sclerosis indexes. Conversely, a high value for S-CON before surgery deviated the model to an Engel II or III outcome.

### Improvement in cognitive functions after epilepsy surgery

The improvement of certain cognitive functions after surgical excision of epileptogenic regions in some patients is well-known since the early epilepsy surgeries. This is most probably explained by the dense inter-connectivity of the structures involved that allows the spread of epileptogenic activity to otherwise normal functioning areas, producing a general depression of function. This explanation was already drafted in the early 19th century theories about remote lesion effects and recovery of function. For example, regarding epileptic patients, Hughlings Jackson wrote in 1884: “destruction of a small part of the highest centers produces very little effects, whilst sudden and excessive discharges of such small part produces, although indirectly, an enormous effect…. It is notorious that a small part of the brain—a small part of the highest centers— may be destroyed without any striking symptoms; compensation is practically perfect.” (Jackson, 1884). Later, at the beginning of the 20th century, Constantin von Monakow developed a theory he called diaschisis to describe that injury in one brain area may induce significant decrease in activity in functionally connected brain areas, and how if the disruption eventually disappear, give rise to some recovery of function (Finger et al., 2004). In 1914, Monakow explained this theory as follows: “…an ‘interruption of function’ appearing in most cases quite suddenly… and concerning widely ramified fields of function, which originates from a local lesion but has its points of impact not in the whole cortex (corona radiata, etc.) like apoplectic shock but only at points where the fibers coming from the injured area enter into primarily intact grey matter of the whole central nervous system” (text taken from Finger et al., 2004). Similarly, Wilder Penfield proposed in the 1940s that these improvement of cognitive functions after surgery might be explained by a reduction or elimination of the propagation of the epileptiform discharges from the epileptogenic region or “nociferous cortex” as named by Penfield, to normal regions (see Hebb, 1977; Hermann and Seidenberg, 1995 and below sections).

Neuropsychological studies performed by some authors of the present work have shown improvement of intellectual functions after surgery in the medial temporal lobe (Martín et al., 2002). In the present study we report a similar tendency but with higher variability. The working memory differences in HS-patients found in this study before and after surgery could be indicating that the relief from epileptogenic activity induces an improvement of distant brain circuits, where the spread of the epileptogenic activity (hippocampal-prefrontal circuits) was inducing malfunctioning previously. It is important to indicate that there is a relationship between the performance on IQ test and executive functions tests in epileptic patients (Helmstaedter et al., 2019). As shown below, Hermann and Seidenberg explored the interesting example of the poor preoperative performance of the Wisconsin Card Sorting Test (WCST) that occurs in some patients with TLE. This test measures abstraction ability and cognitive flexibility that is the ability to shift from one mode of solution to another on a sorting task. Milner (1963) reported greater deficits in patients with frontal injuries than in patients with posterior brain damage and since then this test is widely used to assess possible deficits in prefrontal or executive function in humans. Hermann et al. (1988) observed in some TLE patients a poor performance on the WCST that improved (or at least did not worsen) after surgery, indicating that this was not due to temporal lobe dysfunction. Instead, they proposed that the poor preoperative WCST was probably due to the propagation of “neural noise” from the temporal lobe/hippocampal epileptic focus “via pathways which link the anterior temporal lobe and hippocampus with frontal areas.” Other researchers confirmed later the poor performance of the WCST in TLE patients (see Hermann and Seidenberg, 1995). However, Corcoran and Upton (1993) proposed that the hippocampus is directly involved in some of the executive functions that are measured with the WCST^8^. Finally, Hermann and Seidenberg (1995) compared the nociferous cortex hypothesis with the hippocampal hypothesis to explain poor WCST in TLE patients^9^ and concluded that impaired preoperative WCST performance that is seen in some TLE patients is not directly caused by hippocampal damage, but by the functional disruption of extratemporal regions, supporting the nociferous cortex hypothesis.

### Interindividual variability: A major characteristic of the human normal and epileptic brain

There are multiple sources of interindividual variability in normal brains, and even more so in those with pathological conditions. In this regard, we should first consider that there is high interindividual variability between epileptic patients not only due to obvious factors such as differences in medical treatment, onset of the disease and pathological substrates, but also because the normal human brain show large interindividual variability in many of its structural and functional attributes. For example, there is pronounced inter-subject variability with respect to brain size, shape and cytoarchitecture (e.g., Amunts et al., 1999, 2005; Uylings et al., 2005). At the microanatomical level, there are variations in the structure of cortical neurons (e.g., Jacobs et al., 1993, 1997; Benavides-Piccione et al., 2021) and in the number and density of cortical synapses (e.g., Alonso-Nanclares et al., 2008; Montero-Crespo et al., 2020; Cano-Astorga et al., 2021; Domínguez-Álvaro et al., 2021). Some of these variations have been attributed to differences in age, sex or education. There are also variations in terms of functional connectivity which has been associated to certain anatomical features such as cortical folding, brain size, amount of gray or white matter and size of different brain regions, as well as with behavioral and demographic variations and other factors (e.g., Mueller et al., 2013; Caspers et al., 2014; Demirtaş et al., 2019; Llera et al., 2019; Bethlehem et al., 2022; Marek et al., 2022). Second, “hippocampal sclerosis” is not a homogeneous entity. This pathology encompasses multiple patterns of neuron loss and gliosis as well as a variety of changes in the expression of numerous molecules in the surviving cells and axonal reorganization including both excitatory and inhibitory axons (see section “GABAergic circuits are altered in temporal lobe epilepsy” and Box 3). Thus, hippocampal sclerosis is associated with a variety of functional alterations, which contributes to interindividual variability. As we will further discuss in the next section, these features made challenging the interpretation of the neuropsychological effects based on surgical procedures and pathological findings.

### Comparing across patients is difficult

There are numerous examples in the literature that report either similar or different conclusions regarding the impact of surgery in the neuropsychology of the TLE patients and the presence or not of hippocampal sclerosis. The differences between studies may be explained by multiple factors, including different clinical characteristics of the patients, extent of brain tissue surgically removed and types and protocols of neuropsychological measures. From the structural point of view, it seems obvious that the neuropsychological differences found between TLE patients may be related to their different pathological conditions. Namely, the type of pathology (for example, tumors, cortical atrophy, hippocampal sclerosis) and whether it is bilateral or unilateral and whether more than one pathology exist. Based on hippocampal connectivity (Boxes 4, 5), it seems obvious that even in TLE cases with unilateral hippocampal sclerosis, differences in neuronal loss in one or various hippocampal regions (DG, CA1-CA3 fields and the subicular complex) between TLE patients might have important functional consequences. The multiplicity of pathways involved could reflect the multiplicity of cognitive functions. It also indicates the various direct and indirect pathways that might contribute to these functions, and thus be differentially affected in pathological conditions, which *a priori* seems to be more directly relevant when comparing NoHS-patients with HS-patients. For example, the entorhinal-hippocampal system together with the perirhinal cortex and parahippocampal cortex represent key brain circuits involved in the process of learning and memory (e.g., Squire and Zola-Morgan, 1991; Milner et al., 1998; Brown and Aggleton, 2001; Amaral and Lavenex, 2006; Squire, 2009a; van Strien et al., 2009; Strange et al., 2014; Squire and Dede, 2015; Kitamura, 2017; Nilssen et al., 2019; Marks et al., 2021; Witter and Amaral, 2021). These brain regions have been proposed to be differentially involved in multiple memory systems (for reviews, see Tulving and Schacter, 1990; Milner et al., 1998; Tulving and Markowitsch, 1998; Squire, 2009a; Squire and Dede, 2015). Since TLE patients may have different pathological characteristics, this would explain the wide range of differences between patients with regard to memory attributes^10^.

Another critical aspect regarding hippocampal connectivity, which is often overlooked in studies of TLE, is concerning the connections of the hippocampus with the frontal cortex. It is important to point out the strong direct projections of CA1 to orbito- and medial prefrontal cortex (in macaque: Barbas and Blatt, 1995; Cavada et al., 2000; Ichinohe and Rockland, 2005; Zhong et al., 2006; review: Sigurdsson and Duvarci, 2015). Since neuronal loss in CA1 is the most typical pathological condition in patients with hippocampal sclerosis, all these patients might show deficits in frontal lobe functions.

Connections of the hippocampus with the frontal cortex might be particularly important regarding the digit backwards, H-T-P and ROCF tests. It is pertinent to emphasize the major differences between both tests. In the H-T-P test, the patient is asked to draw a full-body person and write a name and age. Thereafter, they were asked to do the same for a person of the opposite sex (Figures 8, 10). Thus, the patient is projecting a distorted mental image of the human body. However, in the ROCF test, the patient is asked to copy the figure (for the evaluation of visuospatial constructional ability) and three minutes later (in the present study) they are asked to draw what they remembered (for the evaluation of nonverbal visuospatial memory) (Shin et al., 2006; Figures 5, 6A,B). Consequently, patients with lesions in the hippocampal formation in the right temporal lobe are expected to do worse on the visuospatial memory test than patients with lesions on the left hippocampal formation. Furthermore, as pointed out by Shin et al. (2006), copying the ROCF card is a very complex cognitive task “which involves the ability to organize the figure into a meaningful perceptual unit. Therefore, the ROCF is considered to be a useful tool for the evaluation of frontal lobe function, which is required for strategic planning and organizing.” This is in line with the well know functional interplay between the hippocampus and the prefrontal cortex (for reviews see Milner et al., 1985; Buckner et al., 1999; Laroche et al., 2000; Shu et al., 2003; Sigurdsson and Duvarci, 2015).

Finally, it is also important to consider the amygdala in the interpretation of psychological assessment. The necessary surgical removal of the amygdala in several of the surgical protocols is likely to have an impact on the cognitive performance of the epileptic patients. Among the most significant cognitive functions associated with the amygdala are those linked to the strong interactions with the prefrontal cortex. Particularly relevant to the present results, the amygdala has been implemented in memory-related functions concerned with the emotional significance of external and visceral stimuli (for recent reviews; Palomero-Gallagher and Amunts, 2022; see Dixon and Dweck, 2021). These facts should also be taken into consideration when evaluating the post-surgical psychopathological aspects of these epileptic patients.

## Conclusion

When studying the human brain, the significance of “n” is practically always 1 unless thousands of brains in different conditions are examined. In the present study, it is obvious that our conclusions cannot be generalized to the whole population of TLE patients. However, there are remarkable findings that may help to better understand possible correlations between connectivity and psychology. For example, there are no differences in Full Scale IQ between NoHS-patients and HS-patients using the WAIS test. Thus, alterations of the hippocampal circuitry seem not to affect Full Scale IQ, which is a common neuropsychological finding in studies of TLE. Motivated by the prominent interindividual variability in the TLE population, we were looking for individual characteristics that could provide clues to better evaluate the possible relationship between anatomical connections/structural changes and neuropsychological features.

Finally, we stress that there are patients with serious psychopathological alterations that are not revealed in the standard tests used in neuropsychology, which evaluate only certain cognitive aspects. In some cases, patients are presented with a set of phrases or questions and have to answer “true or false”, or “are you okay?” To this later question, some patients respond “yes,” when in fact they have serious psychological problems with a lack of introspection. For these reasons, we included clinical interview as well as psychological, Rorschach and H-T-P tests to better evaluate possible psychopathological alterations. Along this line, a remarkable finding is that there are changes in the Personality Style after surgery in some NoHS-patients and HS-patients. These changes are very uncommon in the general population because personality style is a highly stable characteristic that is constructed over time and that shapes the structure of the personality. In other words, we should keep in mind that after surgery, patients may be seizure-free or have rare seizures, but the clinical psychological outcome may not be favorable. However, in general, there is a notable lack of psychiatric and psychological follow-up, despite the importance of psychological support for these patients. Unlike previous studies on TLE patients, the present work has included the analysis of emotional and personality aspects, with the hope of providing a better understanding of epilepsy and, consequently, more appropriate clinical care for epileptic patients.

## Data availability statement

The original contributions presented in this study are included in the article/Supplementary material, further inquiries can be directed to the corresponding author.

## Author contributions

JD designed the study, prepared the illustrations for the manuscript, and wrote the manuscript. AK, DF, LA-N, LB-L, and JIA performed histology. JIA and JD analyzed histology. LB-L and JD produced the schematic representations of anatomical connections. RGS performed surgeries on human patients. JD-O, MM-A, and FM performed the psychological studies. RA carried out the machine learning data analysis. JD-O, KSR, and JIA contributed to the final version of the manuscript with input from all authors. All authors contributed to the article and approved the submitted version.

## Footnotes

1 *F* = 5.154, *p* = 0.047; mean ± SD = 4.429 ± 0.606 for NoHS-patients and 2 ± 0.717 for HS-patients.

2 *F* = 5.605, *p* = 0.025; mean ± SD = 1.750 ± 0.329 before surgery and 0.938 ± 0.251 after surgery.

3 *F* = 7.066, *p* = 0.013; mean ± SD = 0.875 ± 0.097 before surgery and 0.500 ± 0.129 after surgery.

4 F = 31.243, p = 0.000; mean ± SD = 0 ± 0 before surgery and 1 ± 0.230 after surgery for NoHS-patients; mean ± SD = 0 ± 0 before surgery and 0.930 ± 0.256 after surgery for NoHS-patients.

5 *F* = 99.11, *p* = 0.000; mean ± SD = 0.062 ± 0.067 before surgery and 2.25 ± 0.274 after surgery for NoHS-patients; mean ± SD = 0.083 ± 0.077 before surgery and 2.25 ± 0.316 after surgery for HS-patients.

6 *F* = 10.44, *p* = 0.003; mean ± SD = 1.063 ± 0.288 before surgery and 0.438 ± 0.133 after surgery for NoHS-patients; mean ± SD = 0.923 ± 0.320 before surgery and 0.154 ± 0.147 after surgery for HS-patients.

7 SHAP is a theoretic approach based on the classic Shapley values from game theory to explain the output of any machine learning model (Lundberg and Lee, 2017).

8 Corcoran and Upton (1993) reached this conclusion by comparing the performance on a modified WCST in three groups of epileptic patients. Group I consisted of 16 patients with medial temporal lobe dysfunction due to hippocampal sclerosis detected with MRI in the left side (*n* = 8) or in the right side (*n* = 8). Group II included 13 patients with lateral temporal lobe dysfunction with no evidence of hippocampal sclerosis on MRI and showing focal cortical dysplasia (4 patients), temporal cyst (*n* = 2), hematoma (*n* = 1) or temporal atrophy (*n* = 6). Group III, consisted of 18 patients with frontal lobe dysfunction showing focal cortical dysplasia (7 patients), cerebral abscess (*n* = 2), hematomas (*n* = 2), gliomas (*n* = 3), and surgery (*n* = 4). Corcoran and Upton reported a poorer performance in the group of subjects with hippocampal sclerosis and concluded that the hippocampus play an important role in the WCST.

9 Hermann and Seidenberg (1995) studied 74 patients with dominant left hemisphere for speech submitted to anterior temporal lobe surgery of the left (dominant) (*n* = 47) or right (*n* = 27) hemisphere. After surgery, patients were classified based in hippocampal pathology in four groups: Group I (*n* = 18): patients with no or mild hippocampal sclerosis in the left temporal lobe. Group II (*n* = 29): patients with moderate or marked hippocampal sclerosis in the left temporal lobe. Group III (*n* = 11): patients with no or mild hippocampal sclerosis in the right temporal lobe. Group IV (n = 16): patients with moderate or marked hippocampal sclerosis in the right temporal lobe. Their main findings are as follows: (1) The four groups performed similarly on the WCST prior to surgery regardless of the presence or absence of hippocampal sclerosis. (2) After surgery, there was no significant differences in the performance of WCST between groups, and in general, a slight improvement in the performance was observed. (3) Comparison of WCST performance before and after surgery between patients with impaired WCST prior to surgery showed no significant differences between the seizure free (*n* = 19) and not seizure free (*n* = 18) groups in age, education, Full Scale IQ, or preoperative WCST performance. Furthermore, they also examined 40 additional TLE patients who completed the same neuropsychological tests but did not undergo surgery (control TLE patients). They found a significant relationship between change in WCST performance and surgical outcome: 11 of 19 (58%) seizure-free patients changed to unimpaired WCST performance postoperatively compared to 3 of 18 (17%) not seizure-free patients and 4 of 14 (29%) of the epilepsy control patients.

10 For example, regarding the formation of declarative memory—which include semantic memory (facts about the world in the broadest sense) and episodic memory (personally experienced events)—, the perirhinal cortex and parahippocampal cortex would contribute to the encoding of objects and scenes, respectively, whereas the hippocampus would contribute by generating associations between the perirhinal and parahippocampal cortex (Squire and Dede, 2015). In general, the left hippocampal formation is more involved in episodic or biographical memory, whereas the right hippocampal formation is more involved in spatial memory processing (e.g., Smith and Milner, 1981, 1989; Spiers et al., 2001; Insausti and Amaral, 2012).
