# Supplementary material for: Neuroanatomical and psychological considerations in temporal lobe epilepsy
Source: Front Neuroanat. 2022 Dec 14;16:995286. doi: 10.3389/fnana.2022.995286 (PMC9794593; doi:10.3389/fnana.2022.995286)
Supplement: Supplementary file 1 [file Data_Sheet_1.zip › Supplementary material/SUMMARY OF PSYCHOLOGICAL INTERVIEWS.pdf]

SUMMARY OF PSYCHOLOGICAL INTERVIEWS

Patient H1, 25-year-old-male
Patient H16, 24-year-old-female
Patient H21, 28-year-old-female
Patient H27, 30-year-old-male
Patient H29, 42-year-old-male
Patient H31, 22-year-old-female
Patient H33, 19-year-old-male
Patient H35, 30-year-old-female
Patient H36, 61-year-old-male
Patient H38, 24-year-old-male
Patient H40, 19-year-old-male
Patient H41, 39-year-old-female
Patient H44, 35-year-old-male
Patient H48, 41-year-old-male
Patient H50, 39-year-old-male
Patient H57, 27-year-old-male
Patient H61, 18-year-old-female
Patient H65, 22-year-old-female
Patient H67, 39-year-old-male
Patient H69, 50-year-old-male
Patient H75, 37-year-old-male
Patient H80, 44-year-old-female
Patient H84, 31-year-old-male
Patient H85, 26-year-old-male
Patient H94, 26-year-old-male
Patient H104, 32-year-old-male
Patient H108, 50-year-old-male
Patient H109, 22-year-old-female
Patient H115, 40-year-old-female
Patient H123, 25-year-old-female
Patient H136, 20-year-old-female
Patient H138, 41-year-old-femal**e**
Patient H141, 49-year-old-male
Patient H164, 23-year-old-male

Patient H1, 25-year-old-male

Presurgical observations

Education: Spanish 2nd grade of Secondary School (equivalent to US 8th grade).

The patient experiences ‘strange feelings’ in his stomach and head in the phase prodromal to seizures. During his childhood, there was a period in which his father was aggressive due to alcohol problems, but both father and son appear to have overcome this. He (the patient) feels marginalized by the epilepsy. He reports schizophreniform (SCH) disease symptoms.

Postsurgical observations

The patient reports being worse after the operation and that seizures are a little more frequent. He says that he sometimes has ‘a slightly worse temperament’ and he takes quite a lot of medication. He has had 7 seizures over the course of the month so far, with no fewer than 4–5 per month. His medication has been changed again. He has hand tremors and muscle spasms, especially on his right side. The patient gets out of his chair to explain things and shows like psychotic behavior (schizophreniform, SCH).

See Table 8 *Summary of the brief clinical interview*

Patient H16, 24-year-old-female

Presurgical observations

Education: Spanish 2nd grade of Secondary School (equivalent to US 8th grade).

No severe behavioral alterations are observed (NSA). She shows difficulties regarding social relationships and lack of social skills with a defensive behavior, indifference and demotivation, likely associated with her experience living with the disease.

Postsurgical observations

The patient feels very happy due to the lack of epileptic seizures; despite this, she shows the same difficulties with regard to social skills and depressive symptoms (DEP).

-See Table 8 *Summary of the brief clinical interview*

Patient H21, 28-year-old-female

Presurgical observations

Education: Spanish 4th grade of Secondary School (equivalent to US 10th grade).

Although the patient does not complain of psychopathological alterations during the interview (NSA), she does show lacking social skills and depressive features. Both observations are confirmed by Rorschach test, which displays a high score on the suicide indicator The patient says: “*Epilepsy started with my period. I saw flickering colors, always in my right eye […], I suddenly lose consciousness, hyperventilate, move my hands… there are times when I control it if I can. I notice that [a seizure] is coming and I feel a bit afraid, as if I am going to see the images… colored lights, a very bright light and it’s like it’s flickering. There is loss of consciousness and it’s like getting very nervous. Previously, [it happened] many more times a day, now [it happens] before and after my period and during ovulation. I don’t usually have it during my period.*”

Postsurgical observations

The patient is happy with the surgery, despite that there has only been a reduction in the number of seizures (she only has 1 or 2 per month). She still shows lacking social skills, depressive features and risk of suicide (in the Rorschach test). Increased activity is observed (hypomanic behavior HB and anxiety ANX).

-See Table 8 *Summary of the brief clinical interview*

Patient H27, 30-year-old-male

Presurgical observations

Education: Spanish 2nd grade of Secondary School (equivalent to US 8th grade).

The patient does not complain of psychopathological alterations (NSA), but —during the interview— interpersonal relationship difficulties and depressive features are observed and the clinical observations are confirmed by Rorschach test, in which the risk of suicide indicator is also apparent.

Postsurgical observations

The patient is happy due to the reduction in the number of epileptic seizures. He reports having significant aggressive (AB) and depressive symptoms (DEP) following surgery, but says that he has overcome these symptoms. Depressive features are observed.

-See Table 8 *Summary of the brief clinical interview*

Patient H29, 42-year-old-male

Presurgical observations

Education: Spanish 2nd grade Secondary School (equivalent to US 8th grade).

Depressive symptoms (DEP) are observed, likely linked to the experience of living with the epileptic disease and its limitations. The patient shows psychotic traits (SCH) and complains about his cognitive functions.

Postsurgical observations

The patient is happy due to the significant reduction in the number of seizures. He still has depressive features and the suicide indicator is apparent in the Rorschach test. He refers to having overcome severe postsurgical depression (DEP). He complains of perceived changes in his psychology following the operation, saying, *"[Surgery] has affected my personality, my 'way of being', with a lot of nervousness, fears, I'm not getting a lot done, I get irritated, feelings are stronger; laughter and crying are, like, more intense now. I didn't cry watching a movie before*."

-See Table 8 *Summary of the brief clinical interview*

Patient H31, 22-year-old-female

Presurgical observations

Education: Spanish 2nd grade of Secondary School (equivalent to US 8th grade) and Vocational Training in Administrative Management.

The patient complains of her memory. She is very affected by the experience of living with epileptic disease and shows an inferiority complex. She relates that in the case of one seizure she was insulted by being called a drug-addict. She has attempted suicide twice (SA), the first time after being sexually abused. She reports that menstruation increases the number of seizures. She shows depressive symptoms (DEP), impulsive reactions (IMP) and paranoid traits (PAR).

Postsurgical observations

The patient reports that she is better than before now, despite memory problems and the continuing seizures (although they have reduced notably in frequency. She has divorced. She complains about difficulties finding a job, and about the consequences of living with the disease. She shows depression (DEP).

-See Table 8 *Summary of the brief clinical interview*

Patient H33, 19-year-old-male

Presurgical observations

Education: Spanish 6th grade of Primary School (equivalent to US 6th grade).

The patient complains about the problems associated with living with the disease, with regard to both cognitive functions and psychosocial aspects. He shows difficulties with social skills and depression (DEP).

Postsurgical observations

The patient is happy with the outcome of the surgery; the duration of the seizures has been reduced, although their frequency remains the same. He still has difficulties with social skills and depression (DEP), despite feeling a slight improvement in mood. He is a person who tends to act (impulsively, IMP) rather than thinking.

-See Table 8 *Summary of the brief clinical interview*

Patient H35, 30-year-old-female

Presurgical observations

Education: Spanish 2nd grade of Secondary School (equivalent to US 8th grade) and Vocational Training in Childcare.

The patient does not complain of psychopathological alterations (NSA). The patient does not work. She has a friend and a partner. She reports experiencing strange phenomena with the seizures, saying: "*I imagined that I had four feet, I come up with such strange things. My disease makes me 'withdraw inside myself'* ".

Postsurgical observations

The patient is happy with the outcome of the surgery and shows contralateral neuropsychological improvement (NSA). She has difficulties in interpersonal relationships due to lacking social skills. She says, "*I feel more fragile, much more sensitive, I start crying for the smallest of things, I have inner peace, I am more relaxed".*

-See Table 8 *Summary of the brief clinical interview*

Patient H36, 61-year-old-male

Presurgical observations

Education: Spanish 2nd grade of Secondary School (equivalent to US 8th grade) and Vocational Training in Administrative Management.

The patient complains about the negative consequences in his life caused by him suffering from epilepsy, regarding cognitive functions as well as socio-economical and psychological aspects. He does not complain of psychopathological symptoms (NSA).

Postsurgical observations

The patient is happy with the operation, since it has reduced the duration of the seizures, emotions are more intense, he is happier and more cheerful — and more self-confident than before surgery, but still has memory problems and lacking social skills (NSA).

-See Table 8 *Summary of the brief clinical interview*

Patient H38, 24-year-old-male

Presurgical observations

Education: completed Spanish Secondary School (equivalent to US 12th grade).

The patient does not complain of psychopathological symptoms (NSA), except for lacking social skills, and depression associated with his socio-laboral and psychological situation caused by the disease. He reports having been raped when he was 8 years old.

Postsurgical observations

The patient is happy with the outcome of the surgery. The frequency of the seizures has notably reduced. Family members complain of his impulsive nature (IMP). He still displays lacking social skillslacking social skills and depression.

-See Table 8 *Summary of the brief clinical interview*

Patient H40, 19-year-old-male

Presurgical observations

Education: 1st year of the Law School (Spanish University).

During the interview, lacking social skills and depression (DEP) are observed and the suicide score is positive in the Rorschach test. Impulsive traits (IMP) concurrent with the epileptic seizures are observed.

Postsurgical observations

The patient still displays lacking social skills and depression (DEP), but the suicide score is significantly reduced. He is happy with the outcome of the surgery, but still has seizures. Seizure onset occurs alongside hearing loss. He is currently in his 3rd year of Law School.

-See Table 8 *Summary of the brief clinical interview*

Patient H41, 39-year-old-female

Presurgical observations

Education: Spanish 6th grade of Primary School (equivalent to US 6th grade).

The patient does not report psychopathological symptoms (NSA), but lacking social skills is observed. Her medical records make reference to her alcoholic father.

Postsurgical observations

The patient is very happy with the outcome of the surgery, despite her suffering post-surgical hypomanic behavior (HB). She no longer has seizures. She perceives improvement in her cognitive functions and Rorschach test, notable difficulties putting together a specific answer or “mental engram” are observed, and she shows a tendency to be distractible.

-See Table 8 *Summary of the brief clinical interview*

Patient H44, 35-year-old-male

Presurgical observations

Education: Spanish 5th grade of Primary School (equivalent to US 5th grade).

The patient works in the city hall. He presents severe paranoid symptoms (PAR) and lacking social skills. He has a leg amputation due to an accident caused by an epileptic seizure at work (with Renfe, which is a state-owned railway company in Spain). His family reports that he is violent (AB) and that he has had difficulties with the Civil Guard (law enforcement agency in Spain), being tied up on several occasions for outbursts of violence (throwing dangerous objects, kicking someone who disagreed him in the head, etc.). He refuses to cooperate with some of the psychological tests.

Postsurgical observations

He does not have seizures and says that he feels better than before. His mother reports that he had not slept and had been standing up all night long. He refuses to cooperate with some of the psychological tests. He still presents lacking social skills and latent paranoid disorder (PAR) together with other cognitive deficits.

-See Table 8 *Summary of the brief clinical interview*

Patient H48, 41-year-old-male

Presurgical observations

Education: Graduated in Pharmacy, Spanish University (equivalent to Bachelor and Master of Pharmacy).

The patient is married, has a child, and works as a civil servant. He had polio, a problem with alcoholism, which he overcame, and a terrible childhood. He presents depression (DEP) and a complex personality structure associated with childhood experiences.

Postsurgical observations

The patient is very happy with the surgery. He says he feels freer, with a more spirited nature, but also he has some much stronger impulses (IMP) and a degree of behavioral disinhibition (DIS). He does not present depressive features.

-See Table 8 *Summary of the brief clinical interview*

Patient H50, 39-year-old-male

Presurgical observations

Education: Graduated in Business Administration (Spanish University).

The patient reports feelings of *deja vu* and schizophreniform-type (SCH) psychotic ideation with intrusive thoughts —violent and incestuous sexual thoughts—. Instead of stating suicidal ideation, he refers to "*thoughts of murdering myself*" and perceptions of unusual changes in the size of his genitals. He is being treated with Risperdal (antipsychotic drug).

Postsurgical observations

The reduction in the frequency of the seizures makes him feel livelier, despite the break-up with his partner. He complains about money problems, anomia and memory difficulties, but he has a job. He still has difficulties regarding interpersonal relationships and continues to have negative thoughts (SCH), but controls them better. He reports not feeling himself.

-See Table 8 *Summary of the brief clinical interview*

Patient H57, 27-year-old-male

Presurgical observations

Education: Spanish Secondary School (equivalent to US 11th grade).

The patient shows schizophreniform symptoms (paranoid type, SCH) and aggressiveness (AB). He verbalizes suicidal —but unstructured— ideation (SU), depression (DEP), tendency toward gambling (GB) and behavioral disinhibition (DIS).

Postsurgical observations

The patient is happy with the surgery outcome, he feels livelier, although his lacking social skills is heightened. He continues with latent schizophreniform traits (SCH) and maintains thought disorders. He complains of having suffered from severe post-surgical depression (DEP), but is feeling better. His mother comments some family issues associated with her son's gambling (GB) (his mother is in psychiatric treatment).

-See Table 8 *Summary of the brief clinical interview*

Patient H61, 18-year-old-female

Presurgical observations

Education: Spanish 2nd grade of Secondary School (equivalent to US 8th grade) and Vocational Training in Childcare.

The patient reports having had a tough childhood with bullying at school saying, "*my nickname was 'the crazy one'* ", and she refers to having attempted suicide (SA) twice. She currently has depression (DEP) and lacking social skills, despite mentioning that she has a normal life and has friends.

Postsurgical observations

There was no postsurgical evaluation (NA).

-See Table 8 *Summary of the brief clinical interview*

Patient H65, 22-year-old-female

Presurgical observations

Education: Spanish 2nd grade of Secondary School (equivalent to US 8th grade).

The patient does not complain of psychopathological alterations (NSA), except for the effects of the epileptic seizures on her life in general, both at the emotional and cognitive level. She works as a sales assistant in a family bakery, and she has friends and a partner. The Rorschach test shows cognitive and affective perceptual distortions, as well as depressive features.

Postsurgical observations

The patient is happy with the outcome of the surgery, but complains about cognitive deficits and their effects on her life. During the interview, difficulties regarding self-control (DIS) are observed.

-See Table 8 *Summary of the brief clinical interview*

Patient H67, 39-year-old-male

Presurgical observations

Education: Spanish 6th grade of Primary School (equivalent to US 6th grade).

The patient reports having little contact with people, he has no friends or partner, and shows lacking social skills. He spends most of his time at home, doing no activities. He shows psychopathic traits (PD) and a tendency towards hypochondria (HYP).

Postsurgical observations

The patient is happy with the outcome of the surgery but it is difficult to specify the pre-/post-surgery changes. Apart from not having seizures, he is still the same person and is leading the same life as before (NSA).

-See Table 8 *Summary of the brief clinical interview*

Patient H69, 50-year-old-male

Presurgical observations

Education: Spanish 2nd grade of Secondary School (equivalent to US 8th grade).

The patient complains of cognitive problems (slowing of the thinking process, anomies, memory issues, difficulty in maintaining a conversation) that hinder his daily life — and problems associated with living with the disease.

He is married and has three children. He had an unstructured family with an alcoholic father and he was interned as a catholic seminarian, which was a very bad experience for him. He reports not having a normal life and observes changes in his personality. He presents features of hypervigilance (PAR) and lacking social skills as well as depressive traits (DEP), although these were not significant in the suicide constellation score (Rorschach).

Postsurgical observations

He has had no more seizures, but the positive outcome is eclipsed by difficulties in interpersonal relationships and lacking social skills, as well as other problems with regard to emotion and personality (DEP).

-See Table 8 *Summary of the brief clinical interview*

Patient H75, 37-year-old-male

Presurgical observations

Education: Spanish 6th grade of Primary School (equivalent to US 6th grade).

The patient complains of cognitive dysfunctions related to epilepsy, but he does not report severe symptoms. However, maniform type behavior (MAE) and thought disorders (THD) are observed. He describes himself as an introvert and shows depressive features.

Postsurgical observations

He is happy with the outcome of the surgery. His father says that he had to be admitted to hospital after surgery because of a manic outburst (MAE). Difficulties with regard to controlling impulses (IMP) are observed and thought disorders (THD) persist.

-See Table 8 *Summary of the brief clinical interview*

Patient H80, 44-year-old-female

Presurgical observations

Education: Spanish 2nd grade of Secondary School (equivalent to US 8th grade).

Alterations are not apparent, except for anxious-depressive (ANX, DEP) features associated with living with the disease. She displays verbal memory deficits and lacking social skills. She is married and has two children.

Postsurgical observations

There was no postsurgical evaluation (NA).

-See Table 8 *Summary of the brief clinical interview*

Patient H84, 31-year-old-male

Presurgical observations

Education: Spanish 5th grade of Primary School (equivalent to US 5th grade).

The patient is accompanied by his mother, and he says that he feels "*soft auras in the center of the forehead on the inside, going outwards*". He has mystical delusions (MYS). In the medical report, it is stated that he had auditory and kinaesthetic hallucinations, and he attempted suicide by abdominal stabbing. In a delusional state, the patient stabbed himself twice in quick succession with a knife (to a depth of 10 cm for each stab wound) when he was 25–26 years old; one in the abdomen and the other in his chest, close to the heart. He reports, "*a strong emotional state, caused by belief in God, it is like two embers, one in the belly and the other in the heart, the best experience I have had...I stabbed myself twice, due to belief in God and the resurrection, I was not and I am not afraid of death"*. "*It is beautiful, it is beautiful seeing that, that feeling of happiness...it is a state through believing in God that fills you with happiness"*. He denies that his mother is really his mother; he considers her an impostor.

Postsurgical observations

The patient does not have epileptic seizures but he still shows psychotic phenomena (MYS). After surgery, he spends hours at home staring —with pleasure— at a picture of his hometown church. He says he would like to be alone and to feel "*that, so beautiful*". He says that before he was more spirited and now he feels more subdued. His mother says that he makes strange gestures and dances in the street. During the interview, the patient argues with his mother saying that she is not really his mother.

-See Table 8 *Summary of the brief clinical interview*

Patient H85, 26-year-old-male

Presurgical observations

Education: Spanish 2nd grade of Secondary School (equivalent to US 8th grade).

The patient reports having a normal family environment and he does not complain about the effects of epilepsy on his life. He has lacking social skills despite having many friends and a girlfriend (NSA).

Postsurgical observations

He no longer has seizures and is happy with the surgery, despite feeling a loss of strength in his left arm and leg. He will get married next year and is looking for a job. He shows depressive features (DEPI) and lacking social skills persists.

-See Table 8 *Summary of the brief clinical interview*

Patient H94, 26-year-old-male

Presurgical observations

Education: Spanish 2nd grade of Secondary School (equivalent to US 8th grade).

The patient reports having a constant whistle in his ears (more on the left) that causes insomnia. He describes a troubled childhood linked to his father's alcoholism. He works making chairs and he refers to having broken up with his partner. Hypochondriac (HYP), hysterical (HYS) and depressive (DEP) components appear, as does lacking social skills.

Postsurgical observations

The patient still hears the whistle but it is less intense. He is happy with the surgery and feels calmer and less aggressive than before. He shows depressive features (DEP).

-See Table 8 *Summary of the brief clinical interview*

Patient H104, 32-year-old-male

Presurgical observations

Education: Spanish 2nd grade of Secondary School (equivalent to US 8th grade) and Auto-mechanic Vocational Training.

The patient talks about family problems, describing that his mother treated him very poorly. He lives alone, is single and is an athlete who likes extreme sports. He reports not having friends and being always alone. He tells how sometimes he forgets his own name and does not remember the names of his students (he teaches skiing). He presents anxiety (ANX), as well as paranoid-type (PAR) schizophreniform (SCH) and depressive features (DEP).

Postsurgical observations

There was no postsurgical evaluation (NA).

-See Table 8 *Summary of the brief clinical interview*

Patient H108, 50-year-old-male

Presurgical observations

Education: Spanish 2nd grade of Secondary School (equivalent to US 8th grade) and Auto-mechanic Vocational Training.

The patient reports having a significant loss of hearing in his left hear. He is married and has two children. He works as a mechanic, but is afraid of losing his job. He presents depressive (DEP) and schizophreniform symptoms (SCH) with fear of harming his wife and children, and he fears madness and losing control (IMP).

Postsurgical observations

The patient is happy with the outcome of the surgical resection. He has fewer seizures now, and they are different. He says he breaks into tears and he cannot control himself.

He complains about cognitive dysfunctions. He has fearful obsessions of jumping out the window and he has been given permanent sick leave. His wife says that he does strange things, such as looking under the bed or repetitive movements with a window (SCH).

-See Table 8 *Summary of the brief clinical interview*

Patient H109, 22-year-old-female

Presurgical observations

Education: Spanish 6th grade of Primary School (equivalent to US 6th grade).

The patient is a girl with very few intellectual and emotional resources (her mother is muddled and difficult to deal with). The patient attempted suicide (SA) at least once. According to her mother, "*she feels and behaves like an 8-year-old girl. She does not pay any attention to her parents and she fights with her brother, etc*." She has no partner or friends and works at a recycling center. She shows schizophreniform (SCH) symptoms with significant difficulties in relationships and lacking social skills.

Postsurgical observations

The patient has not had any seizures since surgery. She complains of having suffered from post-surgical depression (DEP) with strange behaviors (SCH). The mother reports a worsening of behavior "*she said that she was a lesbian, and she wanted to jump out the window. But she is less aggressive*." She still shows childish behavior (throwing herself to the ground and kicking her feet out in frustration), as well as schizophreniform and depressive features and lacking social skills.

-See Table 8 *Summary of the brief clinical interview*

Patient H115, 40-year-old-female

Presurgical observations

Education: Spanish 2nd grade of Secondary School (equivalent to US 8th grade) and Auto-mechanic Vocational Training.

The patient is married and has two children. She has no job. During the interview major depression (DEP) emerges, with ideas of cutting her veins and jumping onto the subway and this type of ideation (SU), but they appear to be endogenous characteristics. A major depression is observed. Somatizations of anguish appear as headaches, stomach pain, etc., which are accentuated in moments of tension, as an expression of her anguish. She presents lacking social skills.

Postsurgical observations

The patient is seizure free after surgery but the post-surgical situation was very hard for her, she felt incapable of managing the household (loss of libido), she did not want to leave the hospital, she was afraid to go outside... "*like being born again*". She also spoke of the anguish that she felt during surgery, when she was asleep externally but awake in reality, she could listen to everything but could not speak. She also says that she saw that she got into a tunnel with light. Her husband describes her as more aggressive, sensitive and irritable. She refers to sensations of strangeness and sunlight with a different color. It looks like a "white depression" (DEP), in the sense that it is not reflected in the tests.

-See Table 8 *Summary of the brief clinical interview*

Patient H123, 25-year-old-female

Presurgical observations

Education: Spanish 6th grade of Primary School (equivalent to US 6th grade).

The patient lives with her parents and has no job. She has paranoid (PAR) and delusional schizophreniform symptoms (SCH) —she talks about being a princess and about planting bombs— and she shows impulsiveness (IMP).

Postsurgical observations

She feels very happy with the surgery, she is seizure free. She is a student now. Her parents are very pleased with the observed changes in their daughter, she fights less with her brother, and she is more cheerful. Her psychiatrist has discharged her. She says that she does not have the strange thoughts that she had before, and that "*it is like being born again*". Despite her positive attitude and general improvement, depressive (DEP) and hypervigilance traits (PAR) are observed (latent traits).

-See Table 8 *Summary of the brief clinical interview*

Patient H136, 20-year-old-female

Presurgical observations

Education: Spanish 1st grade of Secondary School (equivalent to US 7th grade).

The patient reports having had a normal childhood, without problems, except for the effect of epileptic seizures in her life. She has friends and lives with her parents and sister. She does not work. She talks about her sudden mood swings and sudden outbursts of anger. Depressive features (DEP), impulsivity (IMP), and paranoid features appear (PAR).

Postsurgical observations

After surgery, the patient does not have epileptic seizures, and she speaks of the changes after the resection: she goes outside more often and no longer feels so much fear. Her family confirms that she feels better, but is still sensitive, depressed (DEP) and impulsive (IMP). She does not mention suicidal ideation, but she shows high scores in the Rorschach test (latent traits).

-See Table 8 *Summary of the brief clinical interview*

Patient H138, 41-year-old-female

Presurgical observations

Education: Spanish 2nd grade of Secondary School (equivalent to US 8th grade).

The patient lives alone (single). She has friends and works as a seamstress, but she has been on sick leave for a year due to ulcerative colitis and epilepsy. She complains of cognitive deficits. She presents depressive symptoms (DEP) and lacking social skills.

Postsurgical observations

There was no postsurgical evaluation (NA).

-See Table 8 *Summary of the brief clinical interview*

Patient H141, 49-year-old-male

Presurgical observations

Education: Spanish 3rd grade of Secondary School (equivalent to US 9th grade).

The patient is married and does not have children. He has no social life. He currently does not work but has worked as a movie operator. Depressive features (DEP) and lacking social skills appear. He has significant cognitive dysfunctions and bradypsychia, making evaluation difficult.

Postsurgical observations

He felt very well after surgery, but 3 months later seizures returned, and he has relapsed with depressive symptoms (DEP). His wife reports finding him much more sensitive, especially in the periods close to the seizures.

-See Table 8 *Summary of the brief clinical interview*

Patient H164, 23-year-old-male

Presurgical observations

Education: Spanish 2nd grade of Secondary School (equivalent to US 8th grade) and Vocational Training in Carpentry.

The patient works as a carpenter with his father. He reports having many friends but presents lacking social skills. The evaluation of this patient is difficult due to the cognitive dysfunctions that he displays. He describes himself as being very nervous (ANX) and says that he cries in private.

Postsurgical observations

There was no postsurgical evaluation (NA).

See Table 8 *Summary of the brief clinical interview*
