# Supplementary material for: Neuroanatomical and psychological considerations in temporal lobe epilepsy
Source: Front Neuroanat. 2022 Dec 14;16:995286. doi: 10.3389/fnana.2022.995286 (PMC9794593; doi:10.3389/fnana.2022.995286)
Supplement: Supplementary file 1 [file Data_Sheet_1.zip › Supplementary material/Supplementary Figure 1.pdf]

Supplementary Figure 1. Example of scoring using the Rey-Osterrieth Complex Figure Test (ROCF)

This figure test comprises 44 single segments, 3 points and one circle with a hierarchical structure of the figure, with the principal organizational unit being a large rectangle with two diagonal crosses. The inner details (small rectangle, circle with three points, parallel lines, etc.) together with the outer details (triangles, square, crosses, diamond and lines) represent the secondary elements of the figure. Patients were also classified according to their overall planning ability: Type I (good planning) and Type II (poor planning), according to the progression of the drawing as indicated by the colored lines or according to the annotations of the examiner in cases where the patient used only the black pencil. An example of the scoring of patient H123 (free of seizures after surgery) using the ROCF is shown below (see the main text for further details).

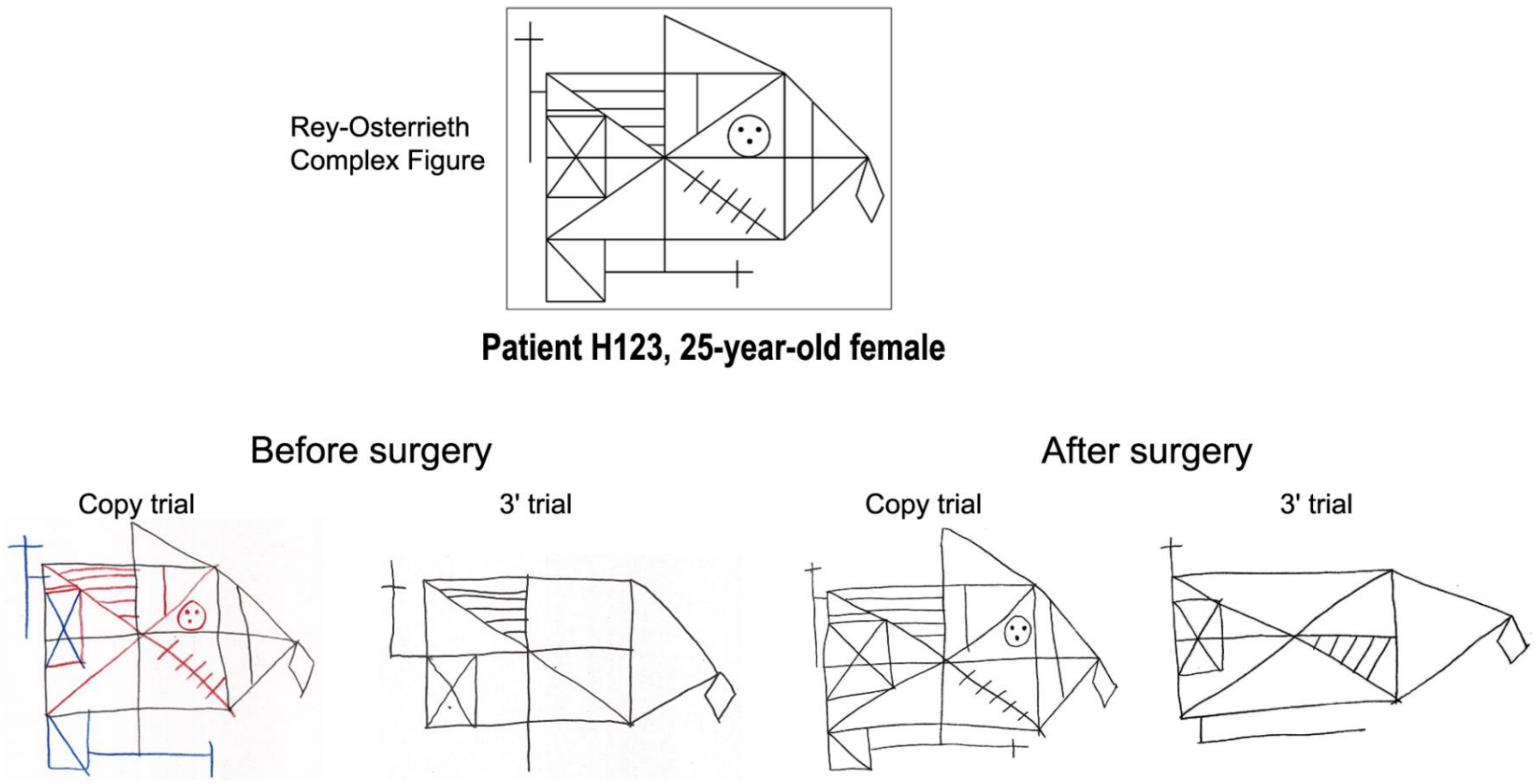

Test scoring criteria		Scores
**Precision**	**Placement on the paper and integrity**	
good	good	2
good	poor	1
poor	good	1
poor, recognizable	poor	0.5
poor, unrecognizable	poor	0

Elements	Copy scores pre	Memory scores pre	Copy scores post	Memory scores post
Cross in the upper left	2	1	2	2
Large rectangle	2	2	2	2
Diagonal cross	2	0.5	2	2
Horizontal line of the large rectangle	2	2	2	2
Vertical line of the large rectangle	2	2	2	0
Small rectangle within the large rectangle	1	1	1	2
Short line above the small rectangle	2	0	2	0
Parallel lines in the upper left triangle	2	2	2	0.5

Triangle on top right of the large rectangle	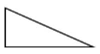	2	0	2	0
Vertical line in the upper right quadrant of large rectangle		2	0	2	0
Circle with three dots	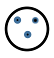	2	0	2	0
Small parallel lines in the lower right quadrant of large rectangle	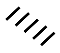	2	0	2	0
Triangle on the right of the large rectangle	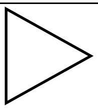	2	2	2	2
Diamond	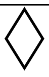	2	2	2	2
Vertical line within the triangle on the right of the large rectangle		2	0	2	0
Horizontal line within the triangle on the right of the large rectangle		2	0	2	0
Cross at the bottom	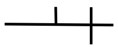	0.5	0	2	0
Square at the bottom with a diagonal line	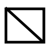	2	0	2	0
**Direct total raw score**		33.5	14.5	35	13.5
**Percentile score***		75	10	90	10

*Based on the data from the Spanish population older than 15 years old (Rey, 1959, 2003)
Furthermore, this patient is classified as Type I according to the overall planning ability (good planning)
