# Supplementary material for: Neuroanatomical and psychological considerations in temporal lobe epilepsy
Source: Front Neuroanat. 2022 Dec 14;16:995286. doi: 10.3389/fnana.2022.995286 (PMC9794593; doi:10.3389/fnana.2022.995286)
Supplement: Supplementary file 1 [file Data_Sheet_1.zip › Supplementary material/Supplementary Figures 2/Supplementary Figures 2.pdf]

Supplementary Figures 2

Examples of histopathological changes in the hippocampal formation and extrahippocampal regions (perihippocampal cortex and lateral temporal cortex) of the resected brain tissue from epileptic patients. The code of patients are included in each figure (supplementary Figures 2-H48, 2-H61, 2-H67, 2-H75, 2-H80, 2-H84, 2-H94, 2-H104, 2-H108, 2-H109, 2-H115, 2-H123, 2-H136, 2-H138 and 2-H164). Hippocampal tissue from non-sclerotic patients showed normal-looking appearance (Fig. 4) and therefore only sclerotic patients are illustrated here. Analysis included Nissl staining, immunohistochemistry against NeuN (neurons); Parvalbumin (PV, a subpopulation of GABAergic interneurons); Calbindin (CalB, labels granule cells and a subpopulation of GABAergic interneurons); anti-HLA-DR antibody (LN3) to visualize microglia cells; and anti-collagen Type IV to label the microvascular network. All the figures in this section have been either published (as indicated in the legends of the figures) or taken from brain tissue that was used in previous articles from our laboratory (Arellano et al., 2004; Andrioli et al., 2007; Kastanauskaite et al., 2009). Additional technical details can be found in these publications. Other immunocytochemical studies using additional primary antibodies that were used for quantitative or qualitative analysis included in Table 3 and supplementary tables 2-4 are not shown.

To help visualizing the changes in PV- and CalB-immunostaining in the hippocampal formation of epileptic patients, the pattern of immunostaining in the hippocampal formation of an autopsy control case with 3 h postmortem (M10: female, 63 years old) is illustrated in supplementary Figures 2-Control.

Bibliography

Andrioli, A., Alonso-Nanclares, L., Arellano, J.I., DeFelipe, J. 2007. Quantitative analysis of parvalbumin-immunoreactive cells in the human epileptic hippocampus. Neuroscience 149:131–143.

Arellano, J. I., Muñoz, A., Ballesteros-Yáñez, I., Sola, R. G., DeFelipe, J. 2004. Histopathology and reorganization of chandelier cells in the human epileptic sclerotic hippocampus. Brain 127:45–64.

Hendrickx, D. A. E., van Eden, C. G., Schuurman, K. G., Hamann, J., Huitinga, I. 2017. Staining of HLA-DR, Iba1 and CD68 in human microglia reveals partially overlapping expression depending on cellular morphology and pathology. J Neuroimmunol 15: 309: 12-22.

Kastanauskaite, A., Alonso-Nanclares, L., Blazquez-Llorca, L., Pastor, J., Sola, R. G., DeFelipe, J. 2009. Alterations of the microvascular network in sclerotic hippocampi from patients with epilepsy. J Neuropathol Exp Neurol 68: 939–950.
