# Supplementary material for: Neuroanatomical and psychological considerations in temporal lobe epilepsy
Source: Front Neuroanat. 2022 Dec 14;16:995286. doi: 10.3389/fnana.2022.995286 (PMC9794593; doi:10.3389/fnana.2022.995286)
Supplement: Supplementary file 1 [file Data_Sheet_1.zip › Supplementary material/Supplementary Figures 2/Supplementary Figures 2-Control.pdf]

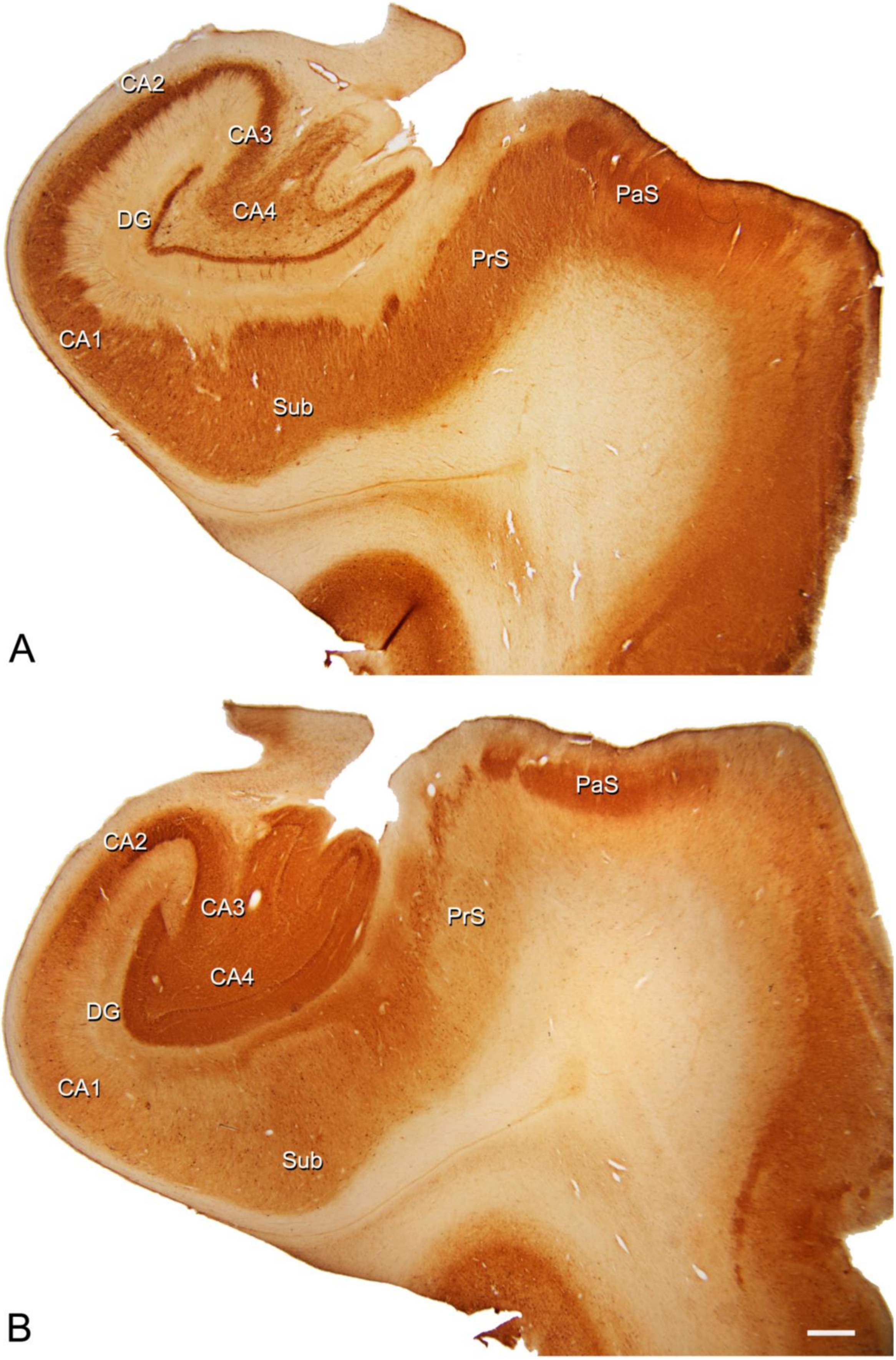

Figure 2-Control-1. **Normal human hippocampus immunostained for PV- and CalB**. Photomicrographs showing the patterns of PV (**A**) and CalB (**B**) immunostaining in adjacent hippocampal sections. Scale bar shown in (**B**) indicates 730 µm in (**A**) and (**B**). CA1-CA4: Cornu ammonis fields; DG: dentate gyrus; Sub: subiculum; PrS: presubiculum; PaS: parasubiculum.

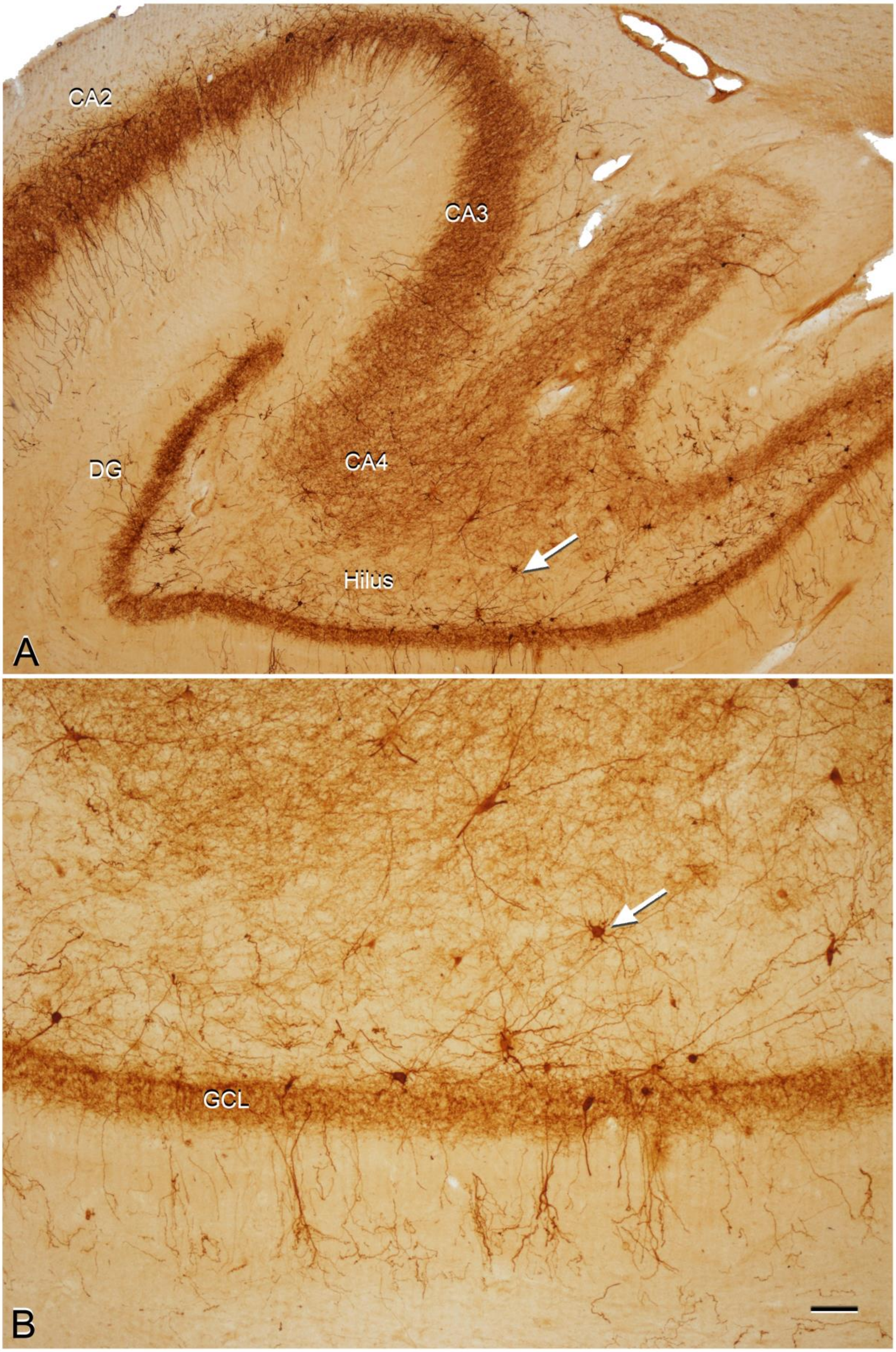

Figure 2-Control-2. **PV-immunostaining of control hippocampus**
(**A**) Higher magnification of Figure 2-Control-2A. (**B**) Higher magnification of (**A**). Arrows indicate a PV-immunoreactive (ir) neuron in the hilus of the dentate gyrus in both panels as reference. Note the relatively few PV-ir cells in the granule cell layer (GCL) of the dentate gyrus. Scale bar in (**B**) indicates 230 µm in (**A**) and 90 µm in (**B**). CA1, CA4: Cornu ammonis fields; DG: dentate gyrus.

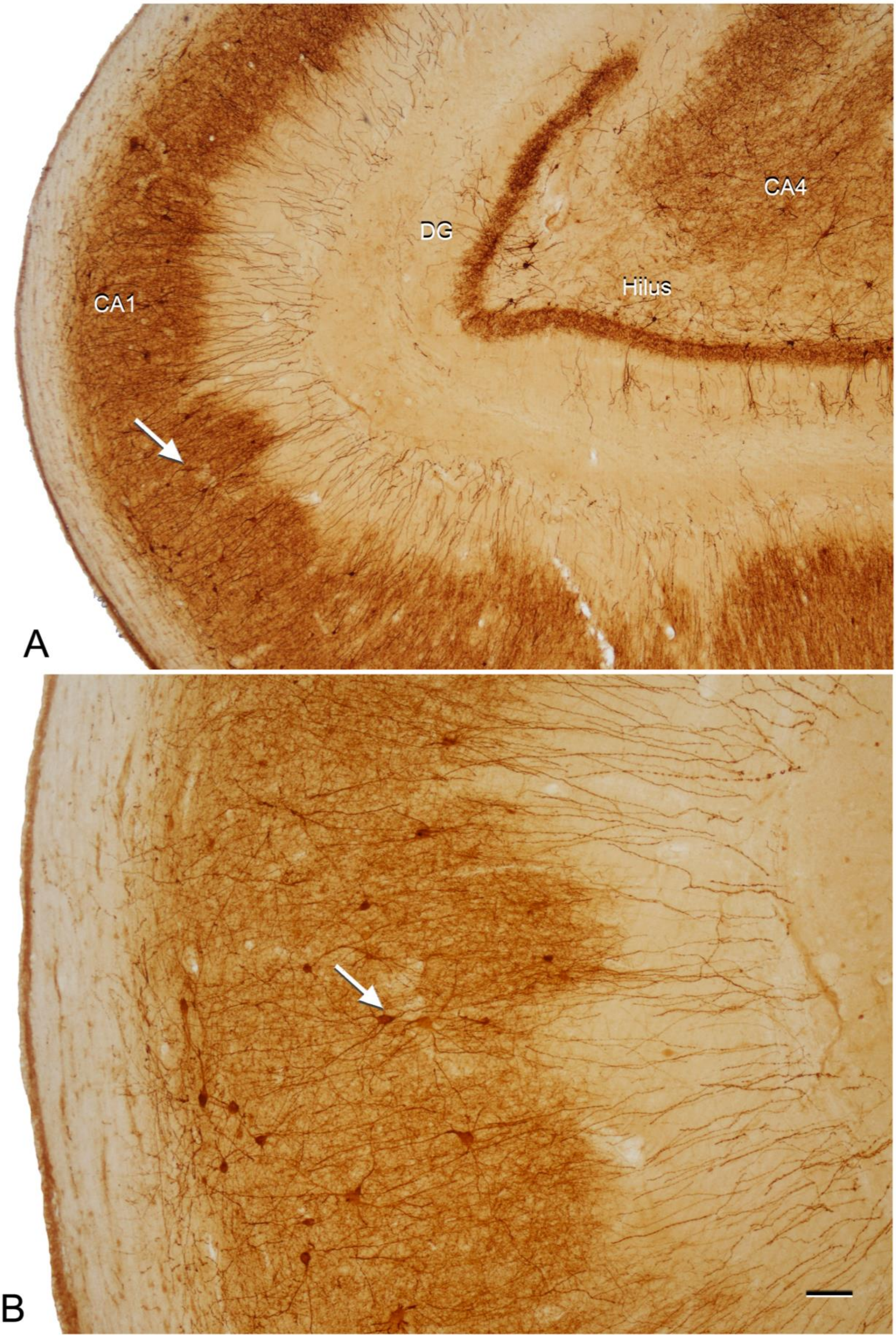

Figure 2-Control-3. **PV-immunostaining of control hippocampus**
(**A**) Higher magnification of Figure 2-Control-2A. (**B**) Higher magnification of (**A**). Arrows indicate the same neuron in CA1 in both panels. Scale bar shown in (**B**) indicates 230 µm in (**A**) and 90 µm in (**B**). The hilus of the dentate gyrus is indicated in (**A**). CA1, CA4: Cornu ammonis fields; DG: dentate gyrus.

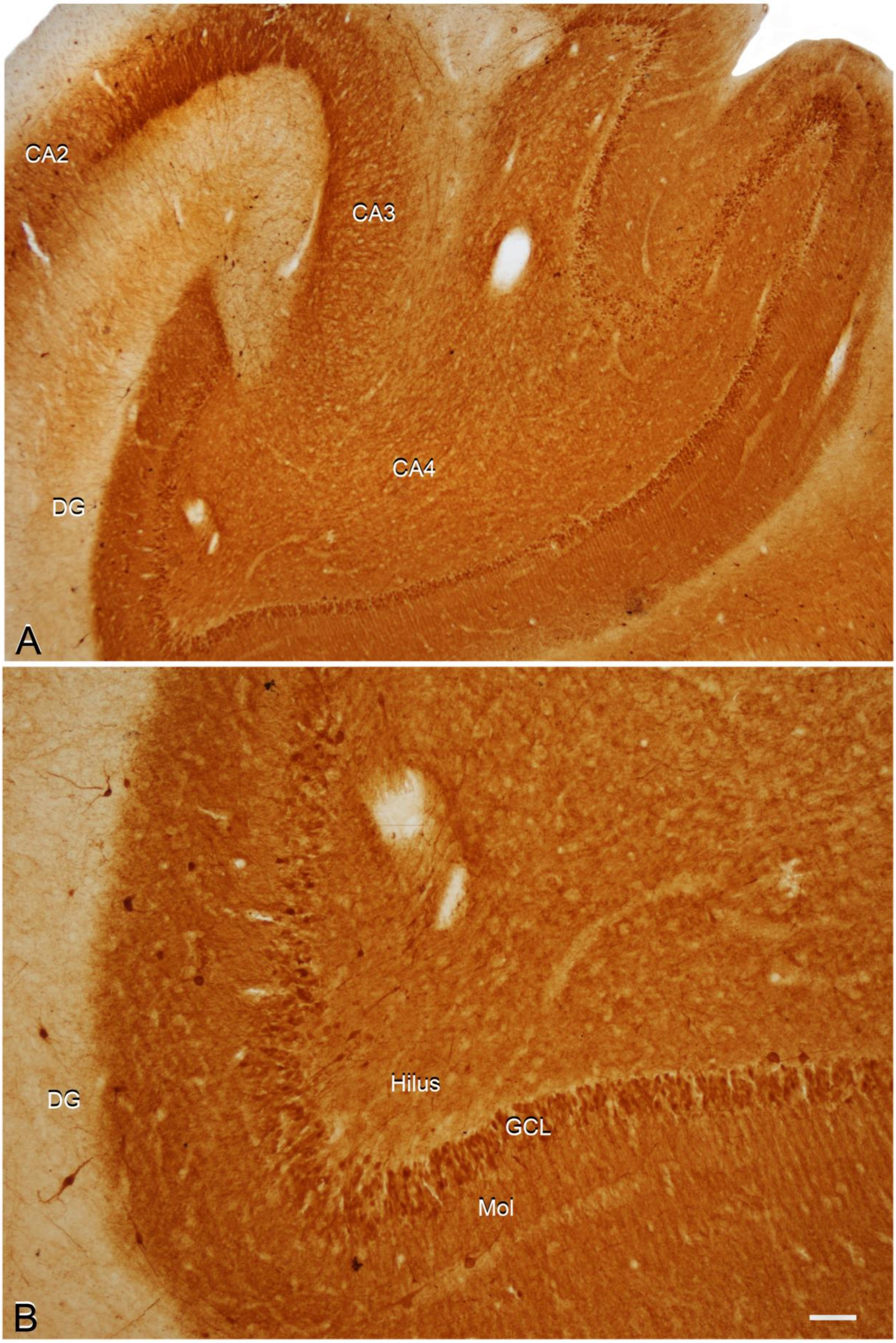

Figure 2-Control-4. **CalB-immunostaining of control hippocampus.**
(**A**) Higher magnification of Figure 2-Control-1B. (**B**) Higher magnification of (**A**). Large arrows indicate the same neuron in CA1 in both panels. Note the dense brownish diffuse staining in the neuropil of CA4, hilus and the molecular layer (Mol) of the dentate gyrus. The vast majority of neurons in the granule cell layer (GCL) are CalB-immunostained. Scale bar shown in (**B**) indicates 230 μm in (**A**) and 80 μm in (**B**). CA1-CA4: Cornu ammonis fields; DG: dentate gyrus.

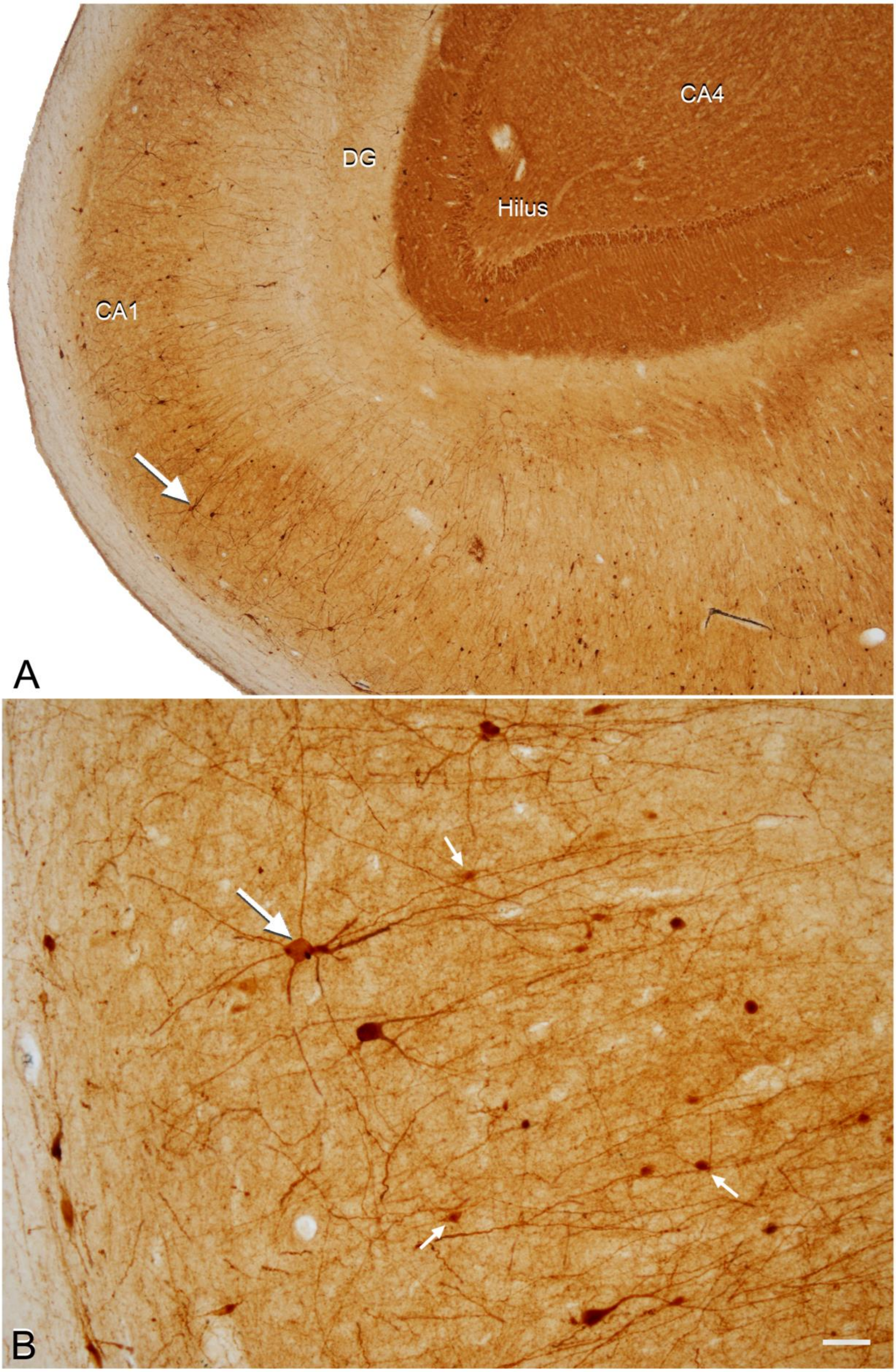

Figure 2-Control-5. **CalB-immunostaining of control hippocampus.**
(**A**) Higher magnification of Figure 2-Control-1B. (**B**) Higher magnification of (**A**). Large arrows indicate the same neuron in CA1 in both panels. Small arrows indicate some small CalB-immunoreactive neurons. Scale bar shown in (**B**) indicates 220 μm in (**A**) and 45 μm in (**B**). CA1, CA4: Cornu ammonis fields; DG: dentate gyrus.
