# Supplementary material for: Neuroanatomical and psychological considerations in temporal lobe epilepsy
Source: Front Neuroanat. 2022 Dec 14;16:995286. doi: 10.3389/fnana.2022.995286 (PMC9794593; doi:10.3389/fnana.2022.995286)
Supplement: Supplementary file 1 [file Data_Sheet_1.zip › Supplementary material/Supplementary Figures 2/Supplementary Figures 2-H104.pdf]

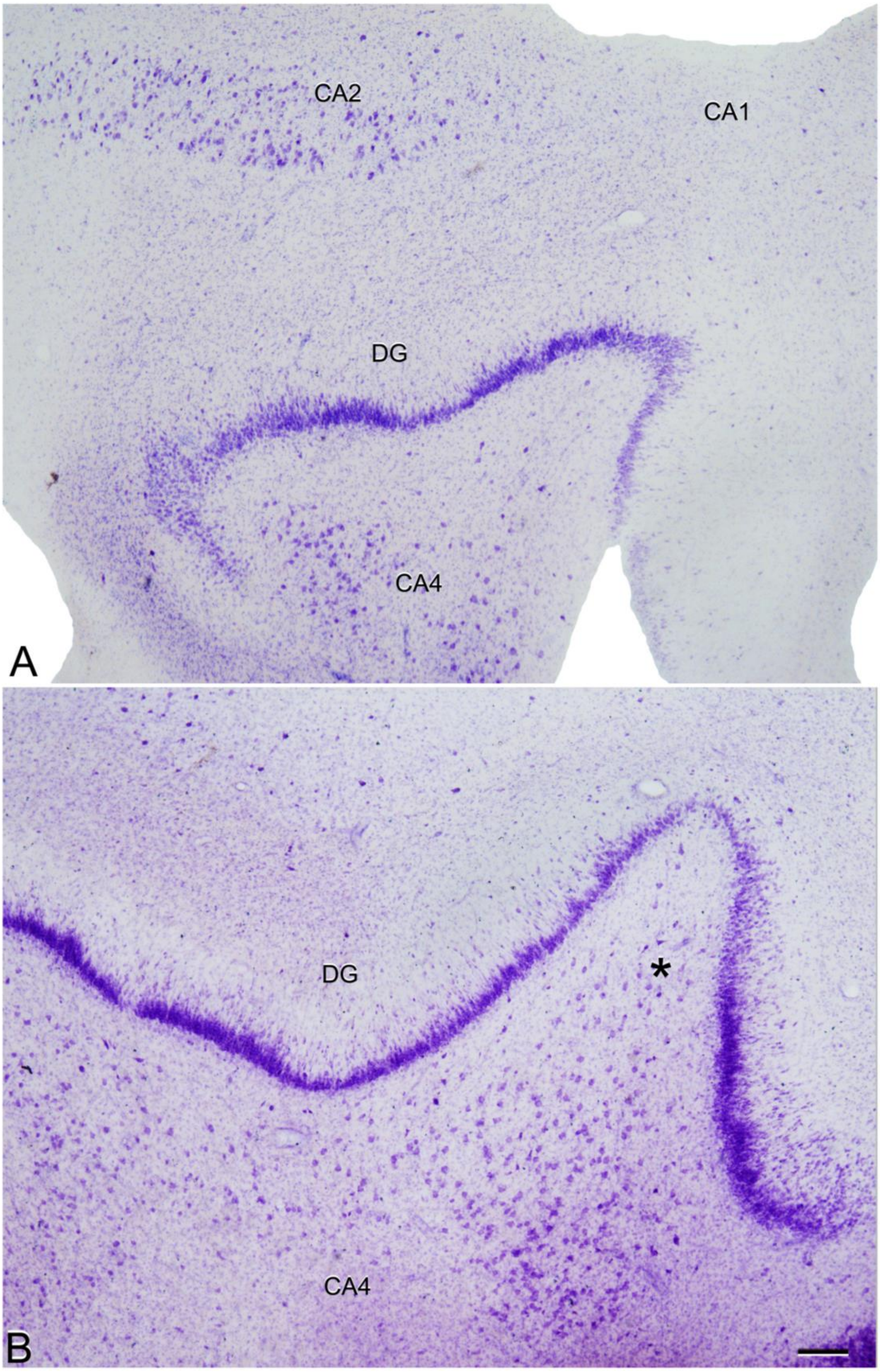

Figure 2-H104-1. **Photomicrographs of Nissl-stained sections**.
(**A**, **B**) Photomicrographs of two adjacent Nissl-stained sections showing at a rostral level the hippocampal formation. Note the extensive loss of neurons in CA1 and CA4 fields as well as the dispersion of the granule cell layer of the DG. The region indicated with an asterisk in (**B**) is also shown in Figure 2-H104-3. At more posterior levels, the hippocampal formation displayed a sclerosis type 1 classic (see Table 3), with less severe neuronal loss. Scale bar shown in (**B**) indicates 230 μm in (**A**) and (**B**). CA1-CA4: Cornu ammonis fields; DG: dentate gyrus.

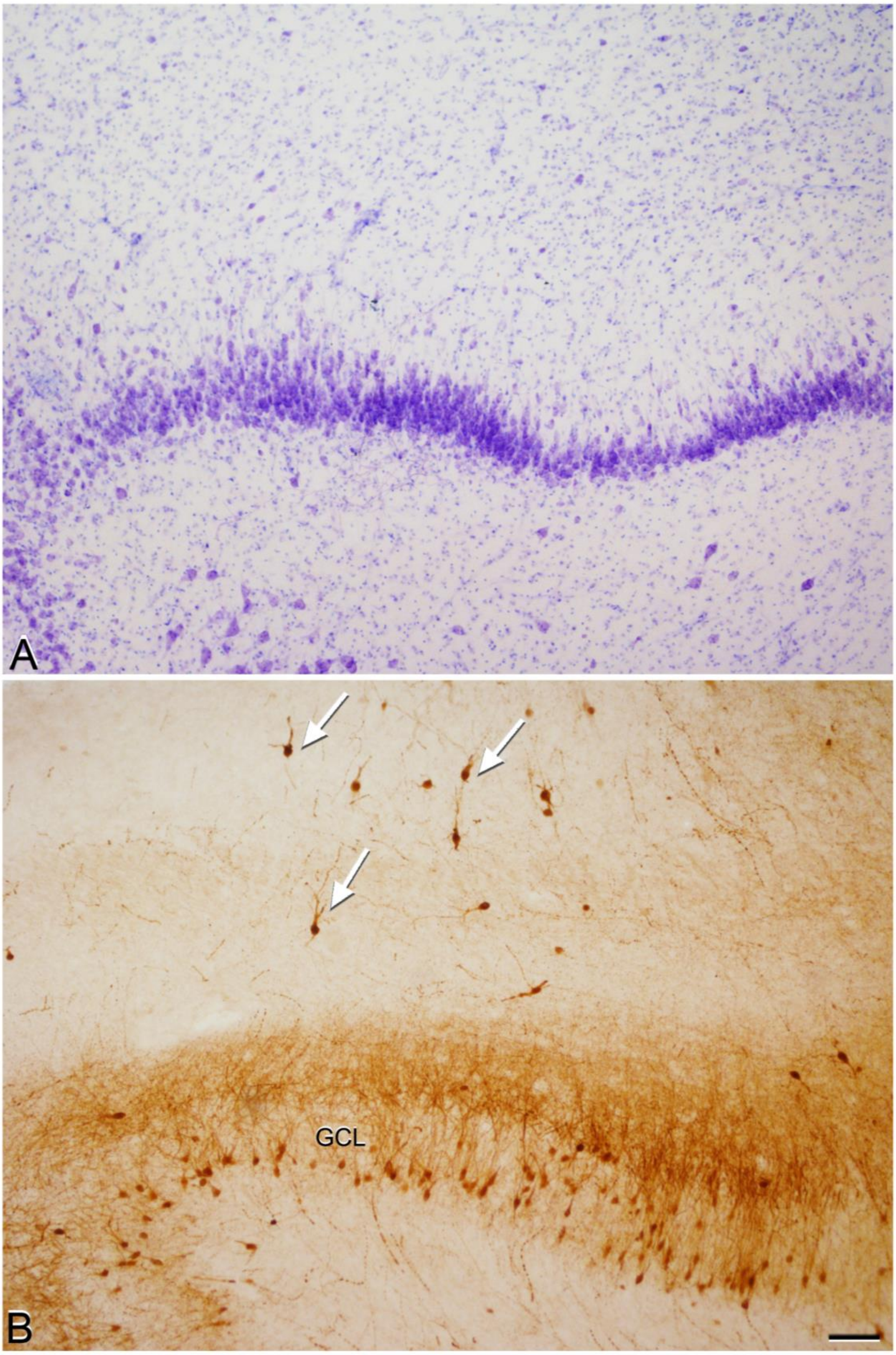

Figure 2-H104-2. **Photomicrographs of Nissl-stained and CalB-immunostained sections**

(**A**) Higher magnification of Figure 2-H104-1A. (**B**) Photomicrograph from a section adjacent to (**A**) immunostained for CalB. Note that numerous CalB-immunostained neurons are present in the granule cell layer (GCL) of the dentate gyrus and the presence of intensely labeled CalB immunostained interneurons (arrows) in a region which in the Nissl stained section display very few surviving neurons. Scale bar shown in (**B**) indicates 100 μm in (**A**) and (**B**).

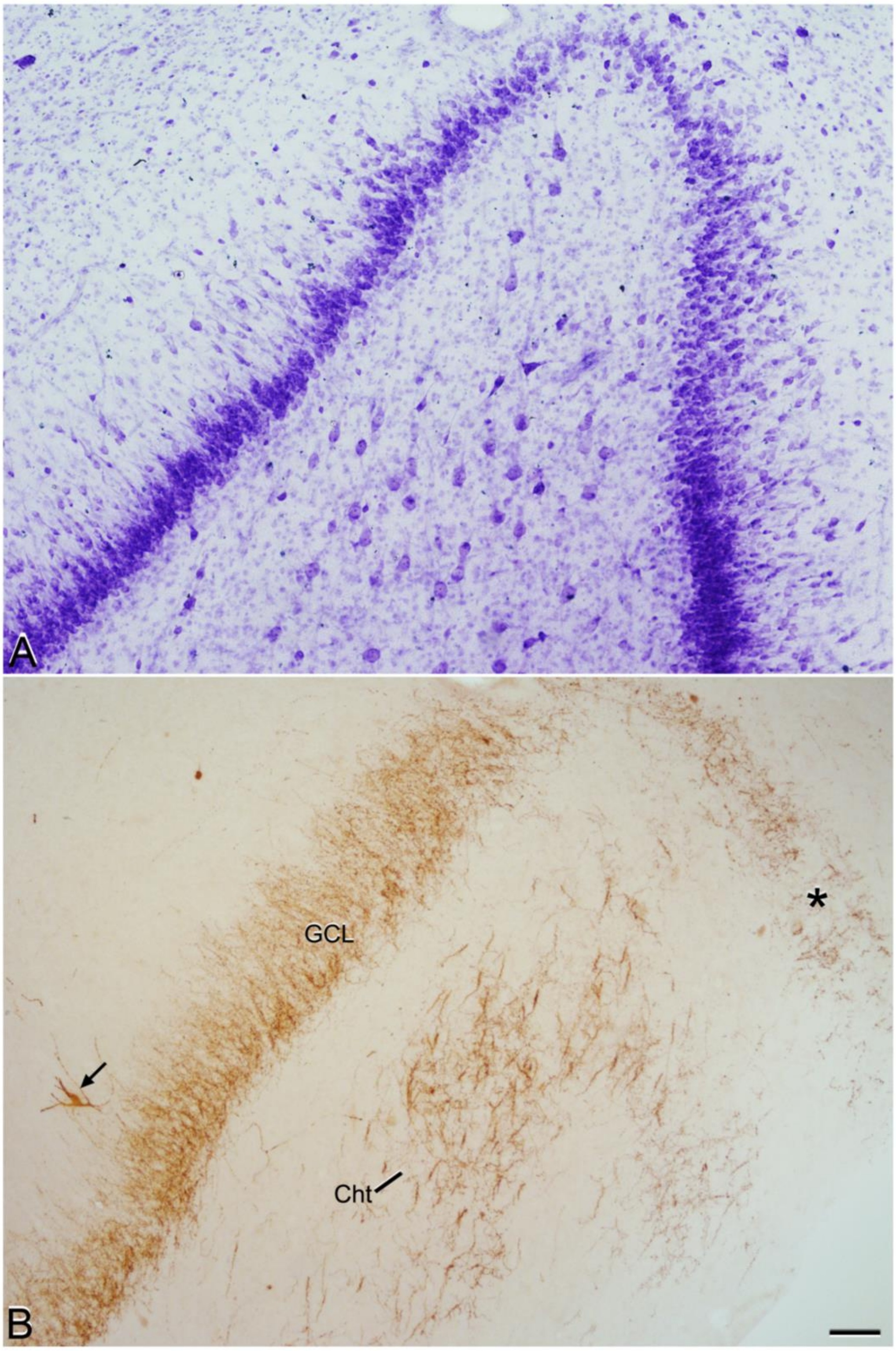

Figure 2-H104-3. **Photomicrographs of Nissl-stained and PV-immunostained sections**.

(**A**) Higher magnification of the region indicated with an asterisk in Figure 2-H104-1B. (**B**), Photomicrograph from a section adjacent to (**A**) immunostained for PV. Note that the intensity of the PV immunostaining of the neuropil in the granule cell layer (GCL) of the dentate gyrus is high in some regions and low in other regions (asterisk), and the presence of PV immunostained chandelier-terminals (Cht) in the CA4 field. These Cht are also shown at a higher magnification in Figure 2-H104-4B. Arrow indicates a PV-immunostained neuron also shown in panel (**A**) of Figure 2 H104-4A. Scale bar shown in (**B**) indicates 100 μm in (**A**) and (**B**).

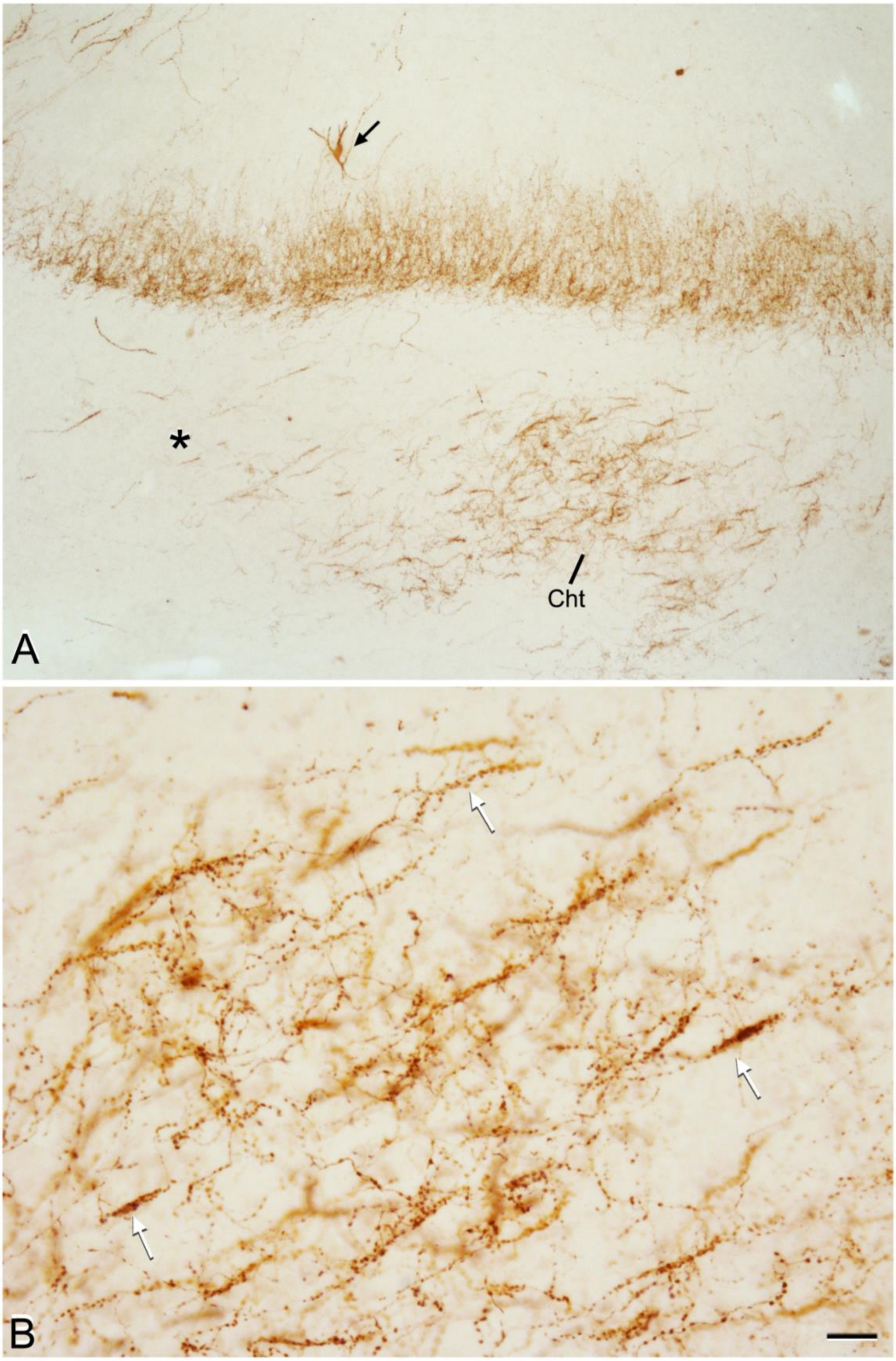

Figure 2-H104-4. **Photomicrographs of PV-immunostained sections**.

(**A**) Photomicrograph from another microscopic field of the same PV-immunostained as that shown in Figure 2-H104-3B (black arrow indicates the same PV-immunostained neuron in both figures) to illustrate the selective presence of PV immunostained chandelier-terminals (Cht) in a microzone of the CA4 field. Asterisk indicates the lack of PV-immunostaining. (**B**) Higher magnification of (**A**) to show PV immunostaining Cht (arrows). Scale bar shown in (**B**) indicates 100 μm in (**A)** and 25 μm in (**B**).

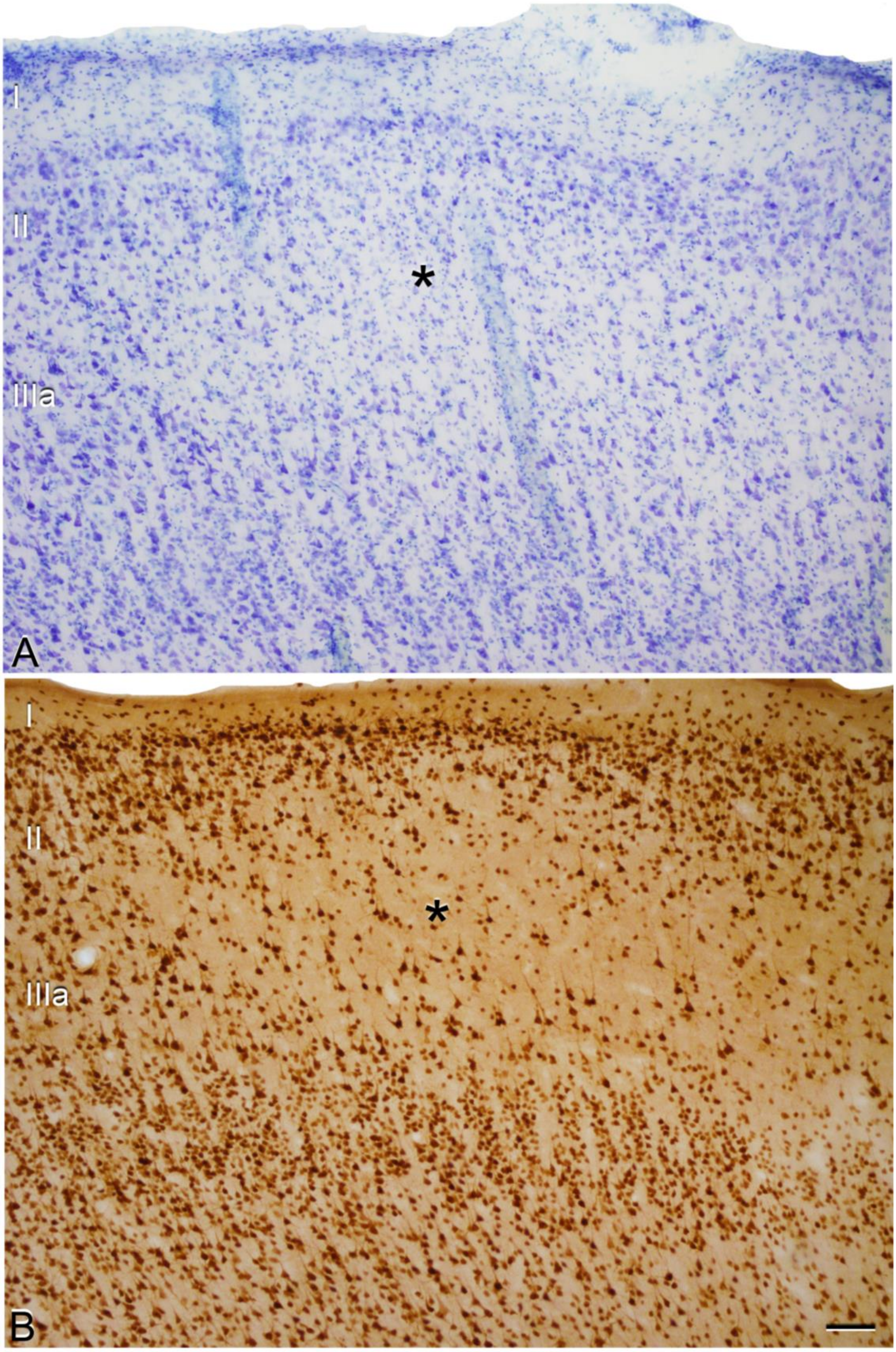

Figure 2-H104-5. **Photomicrographs of Nissl-stained and NeuN-immunostained sections**

(**A**, **B**) Photomicrographs of two adjacent sections, one stained for Nissl (**A**) and the other for immunocytochemically stained for NeuN, showing extensive neuronal loss (asterisks) mostly in layers II and upper layer IIIa of the lateral temporal cortex (area 21 of Brodmann). Scale bar shown in (**B**) indicates 100 μm in (**A**) and (**B**).
