# Supplementary material for: Neuroanatomical and psychological considerations in temporal lobe epilepsy
Source: Front Neuroanat. 2022 Dec 14;16:995286. doi: 10.3389/fnana.2022.995286 (PMC9794593; doi:10.3389/fnana.2022.995286)
Supplement: Supplementary file 1 [file Data_Sheet_1.zip › Supplementary material/Supplementary Figures 2/Supplementary Figures 2-H108.pdf]

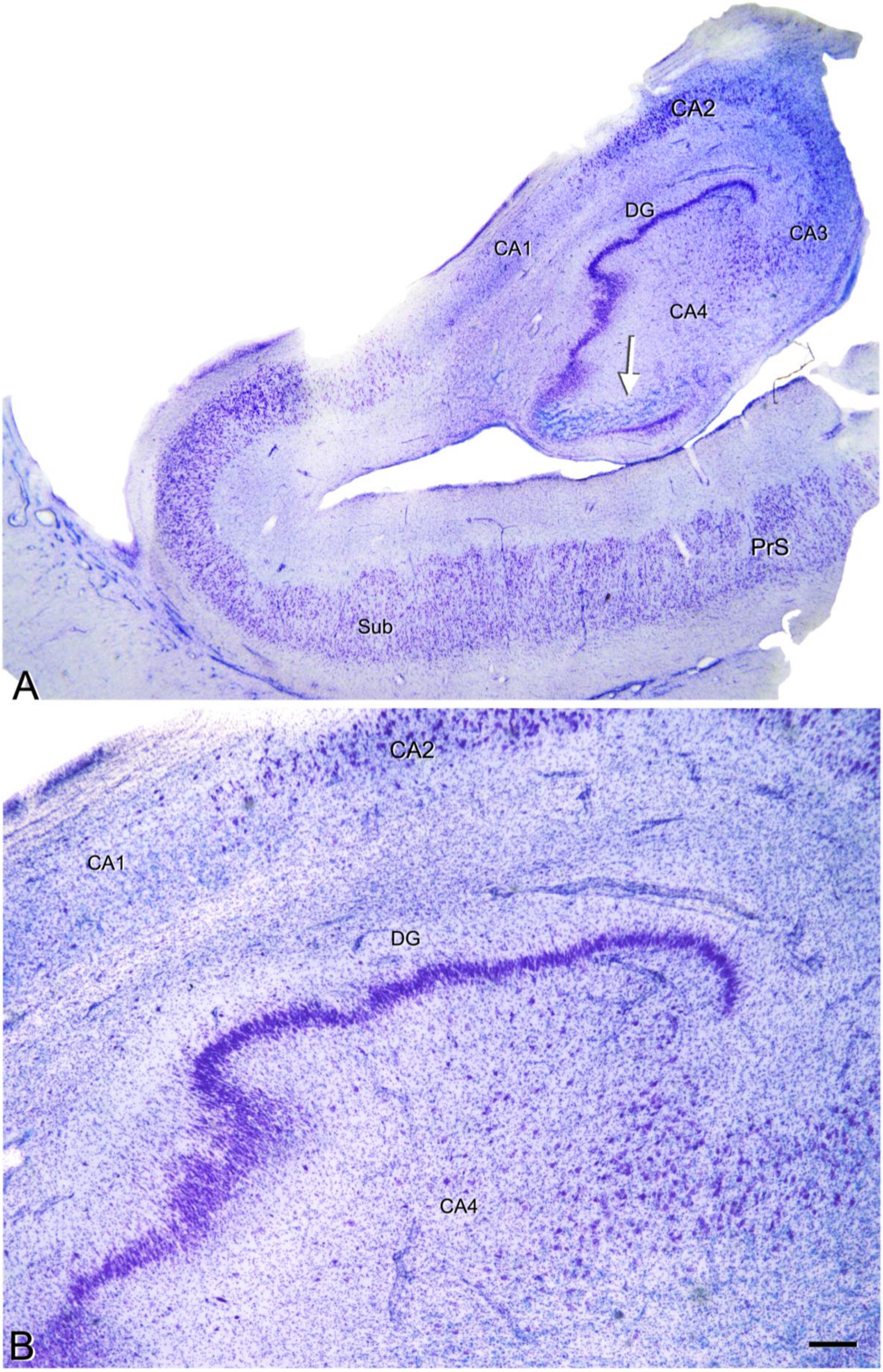

Figure 2-H108-1. **Photomicrographs of a Nissl-stained section**.
(**A**, **B**) Photomicrographs of the hippocampal formation at low (**A**) and high magnification at the border of the CA1/CA2 (B). Note the extensive loss of neurons in CA1 and CA4. Arrow in (**A**) indicate a deposit of corpora amylacea also shown at a higher magnification in Figure 2-H108-2A. Scale bar shown in (**B**) indicates 710 µm in (**A**) and 240 µm in (**B**). CA1-CA4: Cornu ammonis fields; DG: dentate gyrus; Sub: subiculum. PrS: presubiculum.

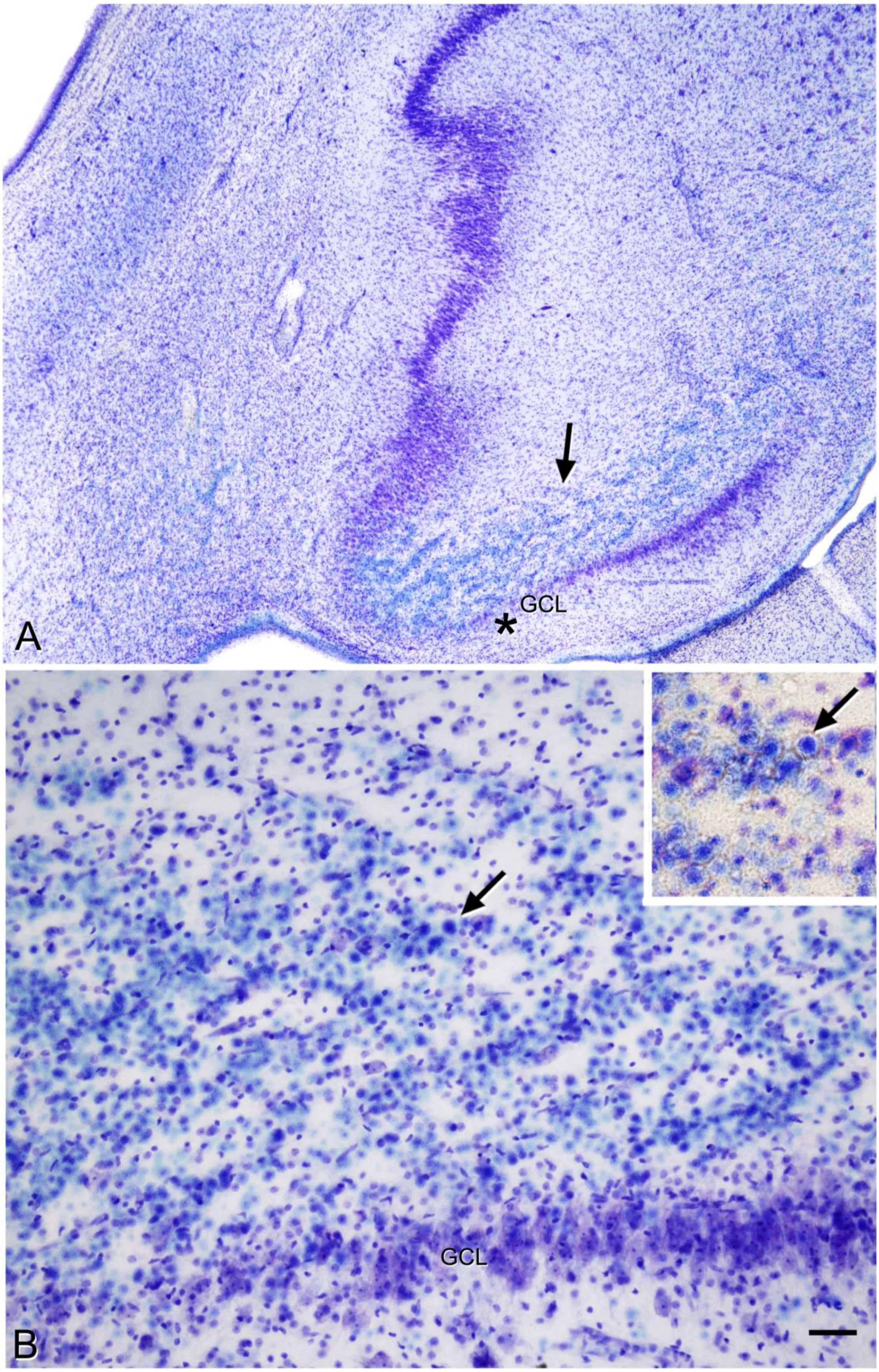

Figure 2-H108-2. **Photomicrographs of a Nissl-stained section**.

(**A**) Higher magnification of the region indicated with an arrow in Figure 2-H108-1A, showing a deposit of corpora amylacea. Note the loss of neurons in the granule cell layer (GCL) of the dentate gyrus (asterisk). (**B**) Higher magnification of (**A**) to show how numerous are the deposits of corpora amylacea. Arrow indicate some corpora amylacea also shown in the inset at a higher magnification and lowering the condenser of the microscope. Scale bar shown in (**B**) indicates 240 μm in (**A**), 40 μm in (**B**) and 30 μm in the inset.
