# Supplementary material for: Neuroanatomical and psychological considerations in temporal lobe epilepsy
Source: Front Neuroanat. 2022 Dec 14;16:995286. doi: 10.3389/fnana.2022.995286 (PMC9794593; doi:10.3389/fnana.2022.995286)
Supplement: Supplementary file 1 [file Data_Sheet_1.zip › Supplementary material/Supplementary Figures 2/Supplementary Figures 2-H109.pdf]

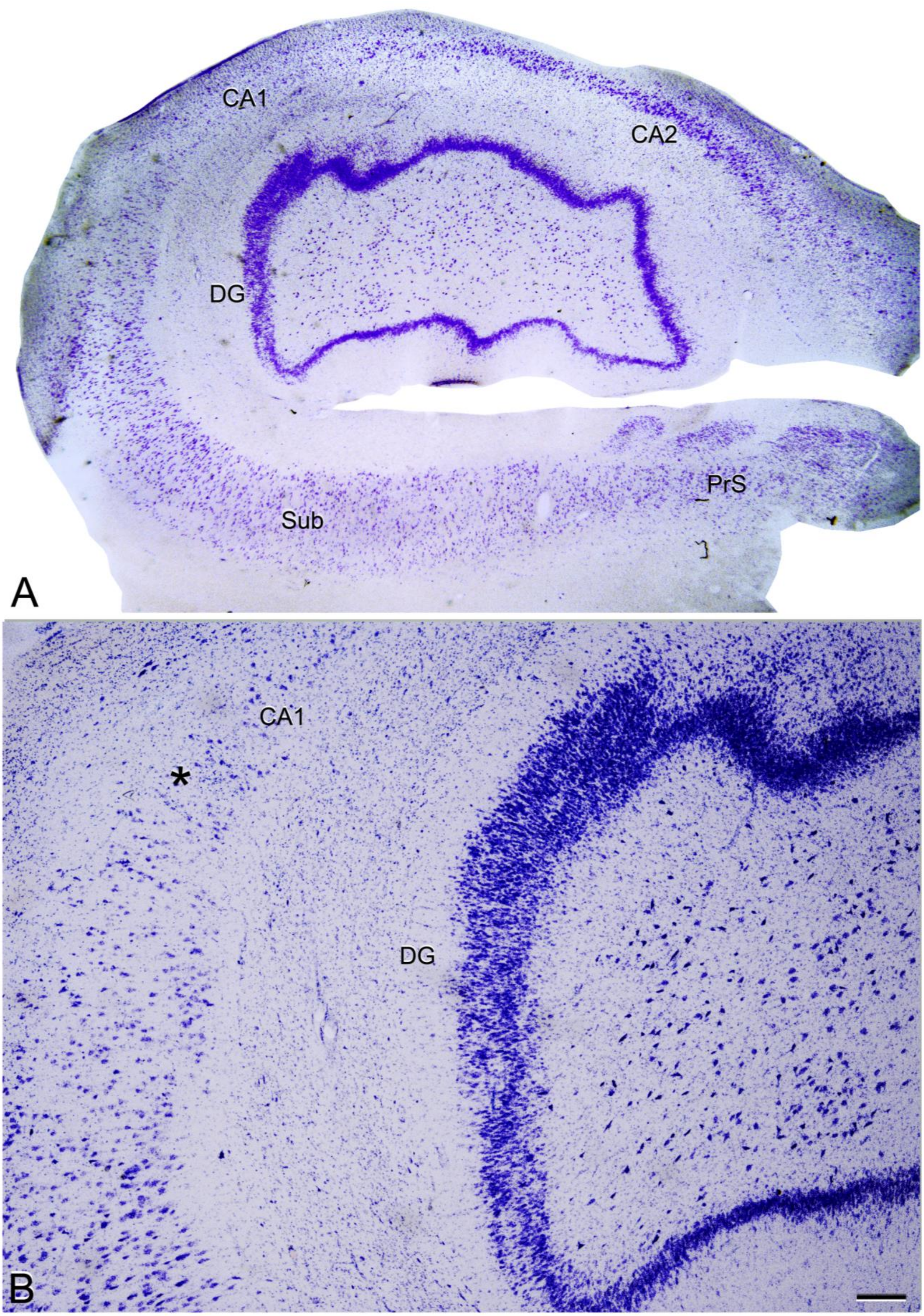

Figure 2-H109-1. **Photomicrographs of a Nissl-stained section**.

(**A**, **B**) Photomicrographs showing at a rostral level the hippocampal formation at low (**A**) and high magnification (**B**). Note the extensive loss of neurons in CA1 (asterisk in **B**). Scale bar shown in (**B**) indicates 675 µm in (**A**) and 235 µm in (**B**). CA1-CA2: Cornu ammonis fields; DG: dentate gyrus; Sub: subiculum. PrS: presubiculum.

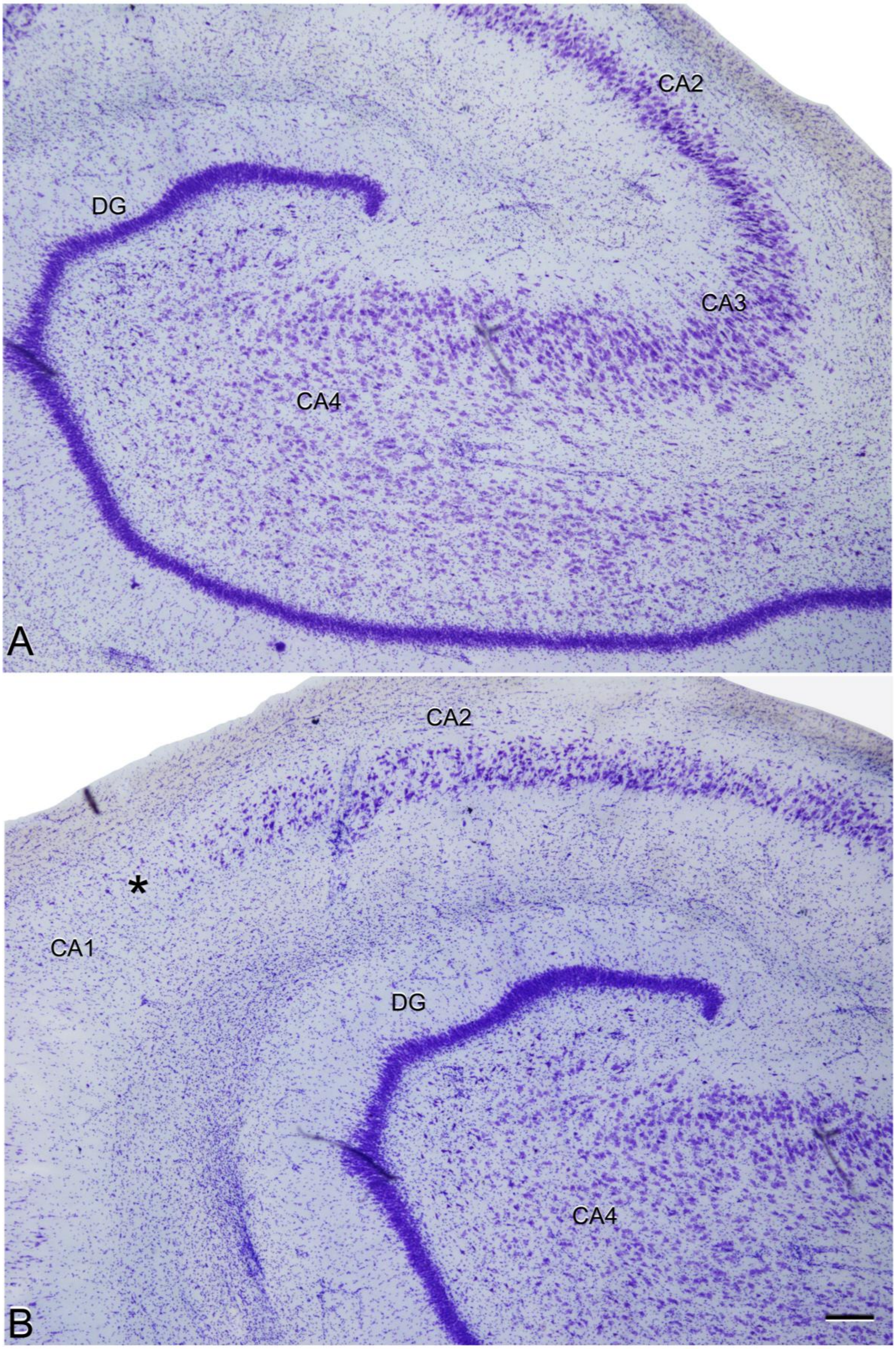

Figure 2-H109-2. **Photomicrographs of a Nissl-stained section**.
(**A**, **B**) Photomicrographs showing the hippocampus at a more posterior level as that shown in Figure 3-H109-1. Neuronal loss is predominately observed in CA1 (asterisk in **B**) (sclerosis type 2). Scale bar shown in (B) indicates 240 μm in (**A**) and (**B**). CA1-CA4: Cornu ammonis fields; DG: dentate gyrus; Sub: subiculum.

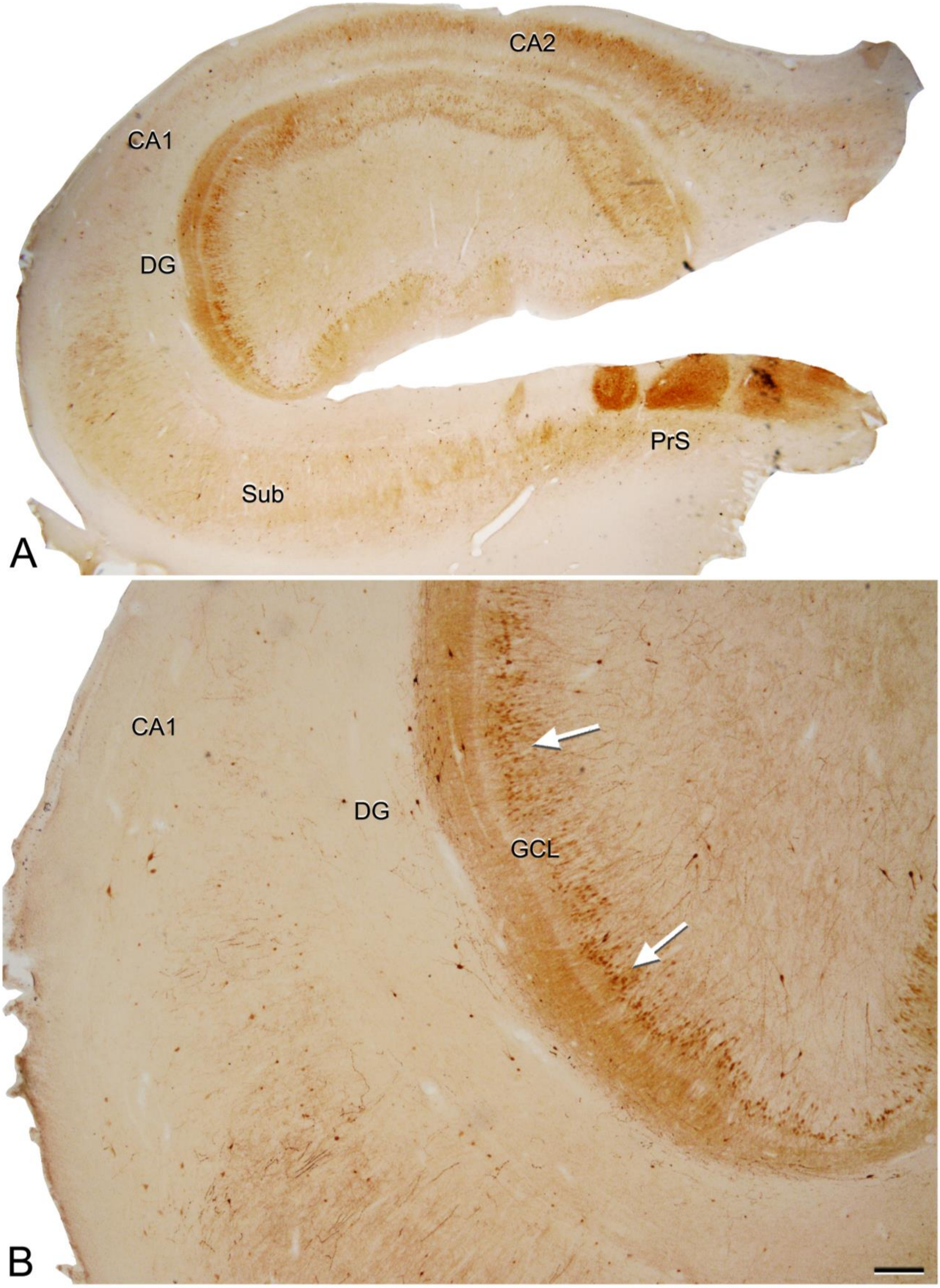

Figure 2-H109-3. **Photomicrographs of a CalB-immunostained section**.
(**A**, **B**) Low- and high-magnification photomicrographs, respectively, of a CalB-immunostained section adjacent to the Nissl-stained section showed in Figure 2-H109-1A. Note the differences in the intensity of labeling for CalB in the different hippocampal fields. In the granule cell layer (GCL) numerous CalB-immunostained neurons are present. Scale bar shown in (**B**) indicates 690 μm in (**A**) and 230 μm in (**B**). CA1, CA2: Cornu ammonis fields; DG: dentate gyrus; Sub: subiculum. PrS: presubiculum.

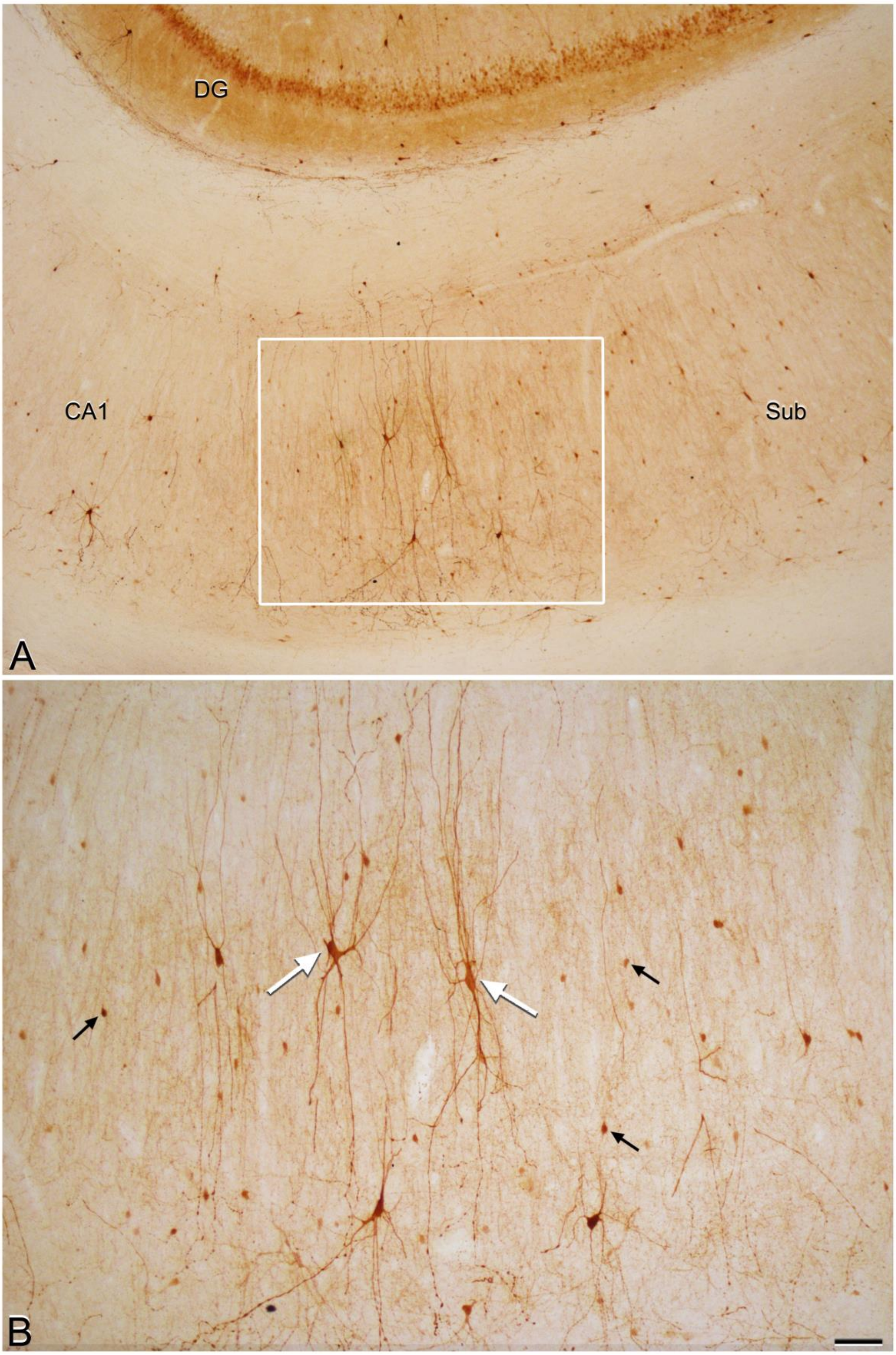

Figure 2-H109-4. **Photomicrographs of a CalB-immunostained section**.

(**A**, **B**) Low- and high-magnification photomicrographs, respectively, of a CalB-immunostained section adjacent to that shown in Figure 2-H109-3A to illustrate the pattern of CalB-immunostaining the border CA1/subiculum. The area indicated by a rectangle in (**A**) is shown at a higher magnification in B. Note the differences in the size of CalB-immunostained interneurons; some are very large (white arrows) whereas others are very small (black arrows). Scale bar shown in (**B**) indicates 230 µm in (**A**) and 90 µm in (**B**). CA1: Cornu ammonis field; DG: dentate gyrus; Sub: subiculum.
