# Supplementary material for: Neuroanatomical and psychological considerations in temporal lobe epilepsy
Source: Front Neuroanat. 2022 Dec 14;16:995286. doi: 10.3389/fnana.2022.995286 (PMC9794593; doi:10.3389/fnana.2022.995286)
Supplement: Supplementary file 1 [file Data_Sheet_1.zip › Supplementary material/Supplementary Figures 2/Supplementary Figures 2-H115.pdf]

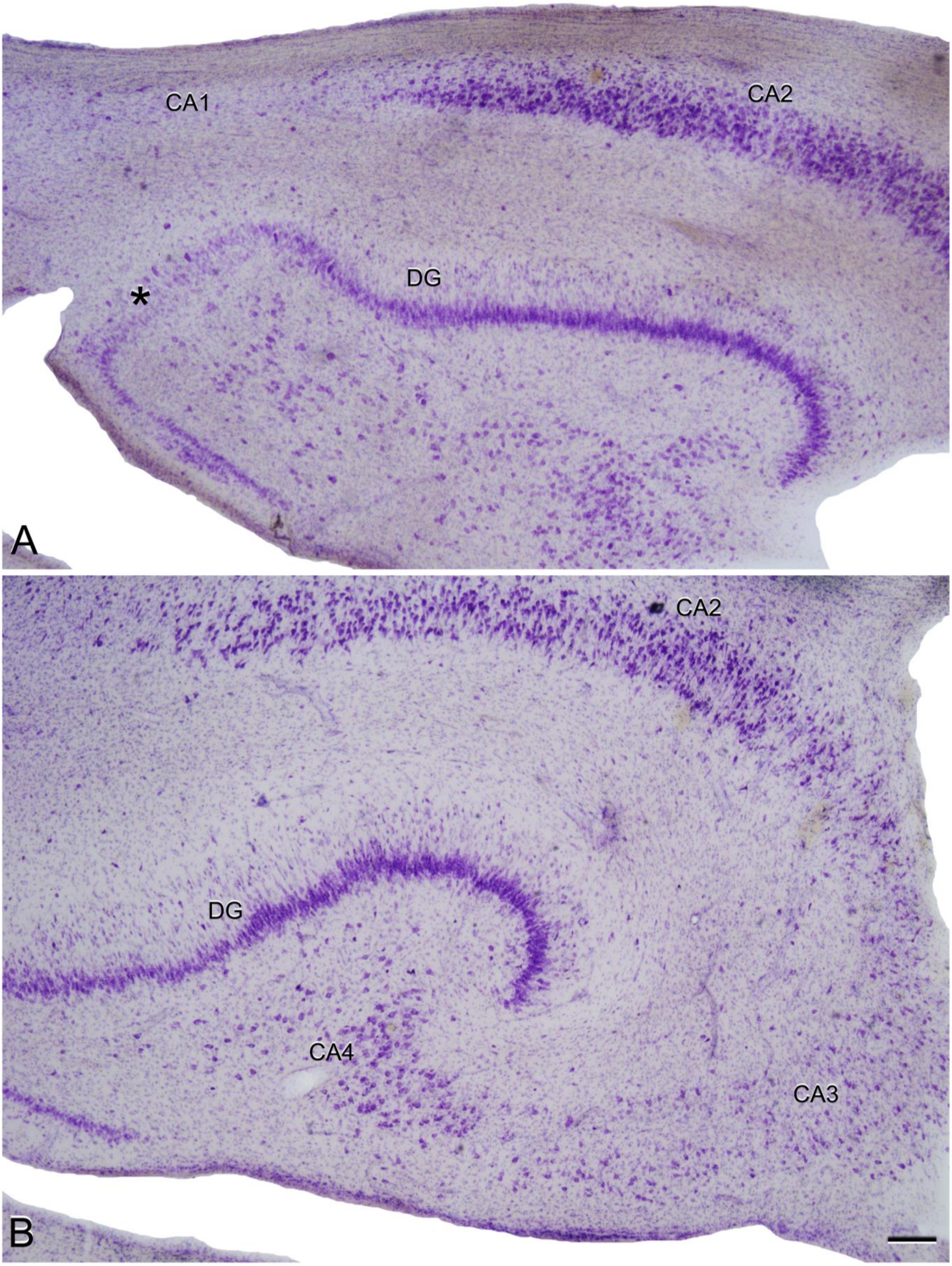

Figure 2-H115-1. **Photomicrographs of Nissl-stained sections**.
(**A**, **B**) Photomicrographs showing the hippocampal formation at a rostral (**A**) and posterior (**B**) levels. Note the loss of neurons in all hippocampal fields except in the CA2. The loss of neurons in the granule cell layer of the dentate gyrus is more noticeable in some regions (asterisk in **A**). Scale bar shown in (**B**) indicates 220 μm in (**A**) and (**B**). CA1-CA4: Cornu ammonis fields; DG: dentate gyrus; Sub: subiculum.
