# Supplementary material for: Neuroanatomical and psychological considerations in temporal lobe epilepsy
Source: Front Neuroanat. 2022 Dec 14;16:995286. doi: 10.3389/fnana.2022.995286 (PMC9794593; doi:10.3389/fnana.2022.995286)
Supplement: Supplementary file 1 [file Data_Sheet_1.zip › Supplementary material/Supplementary Figures 2/Supplementary Figures 2-H123.pdf]

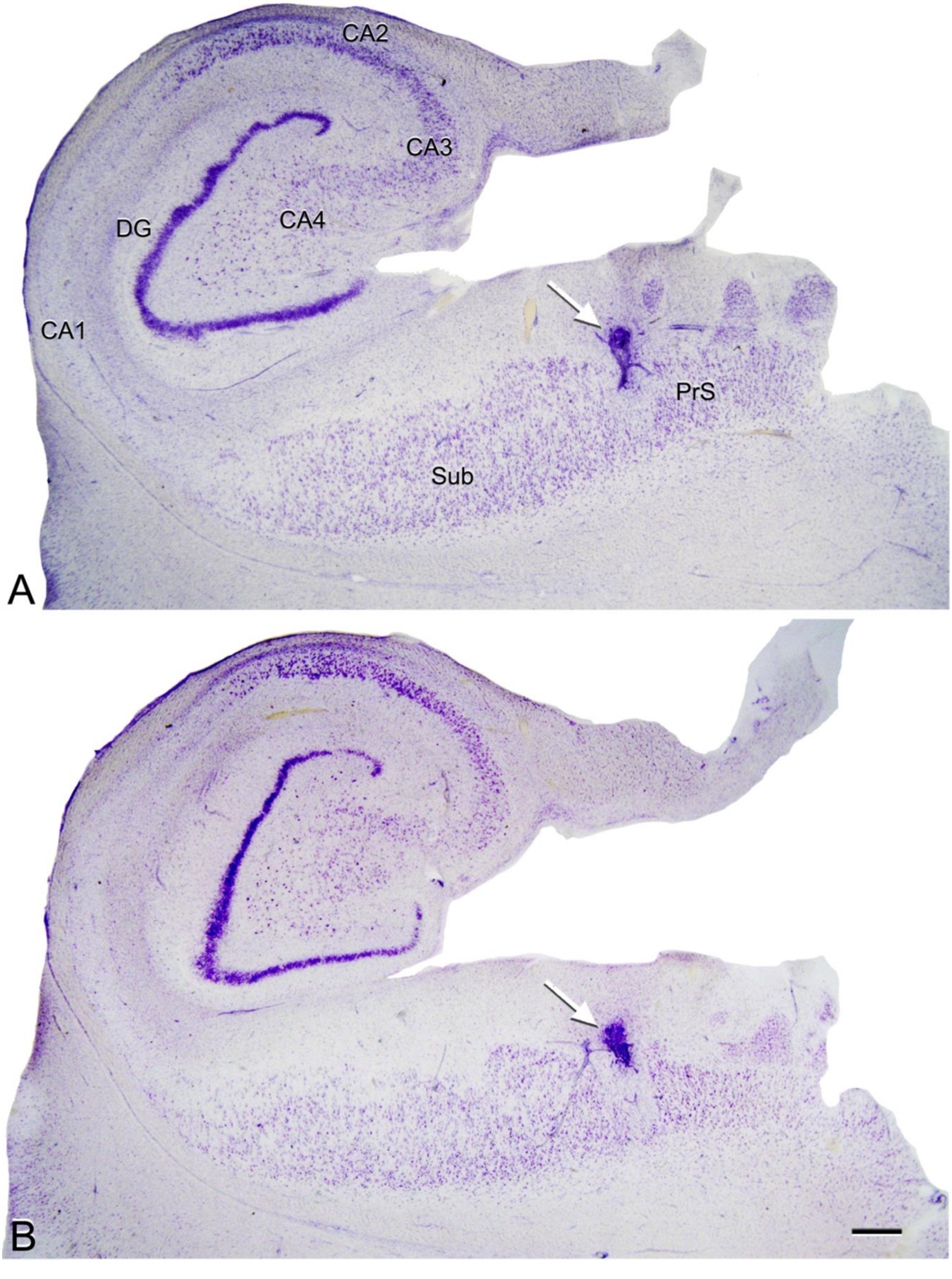

Figure 2-H123-1. **Photomicrographs of Nissl-stained sections**.
(**A**, **B**) Photomicrographs showing the hippocampal formation of two adjacent sections. Note the clear loss of neurons in all hippocampal fields except in the DG and CA2 where the loss is less prominent. Arrow indicate a microvascular alteration at the border between the subiculum and presubiculum which is also shown at higher magnification in Figures 2-H123-3A and 2-H123-3B. Scale bar shown in (**B**) indicates 650 µm in (**A**) and (**B**). CA1-CA4: Cornu ammonis fields; DG: dentate gyrus; Sub: subiculum. PrS: presubiculum.

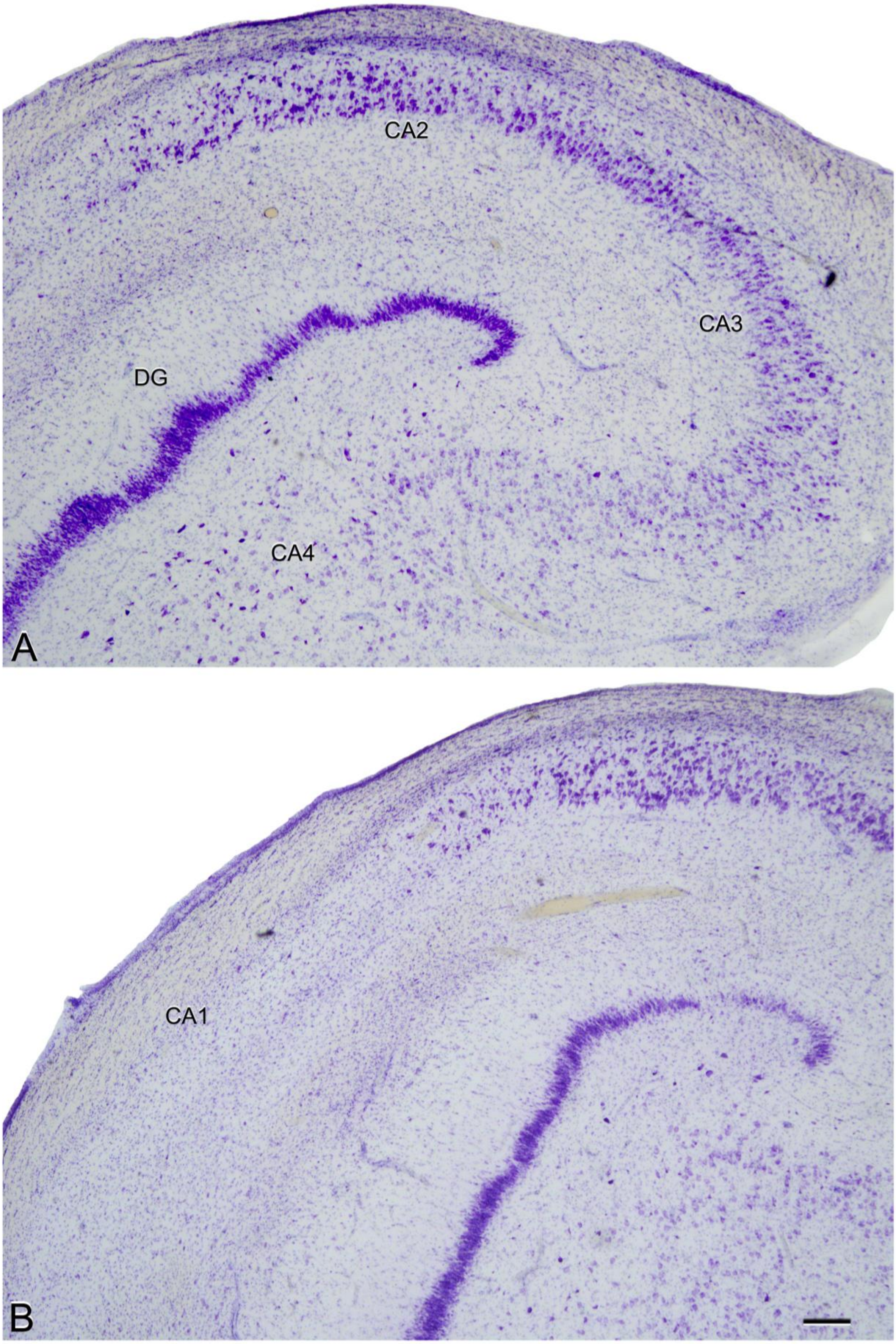

Figure 2-H123-2. **Photomicrographs of a Nissl-stained section**.
(**A**, **B**) Higher magnification of Figure 2-H123-1A to illustrate in greater detail the pathological changes in CA1-CA4 and DG. Scale bar shown in (**B**) indicates 230 μm in (**A**) and (**B**). CA1-CA4: Cornu ammonis fields; DG: dentate gyrus.

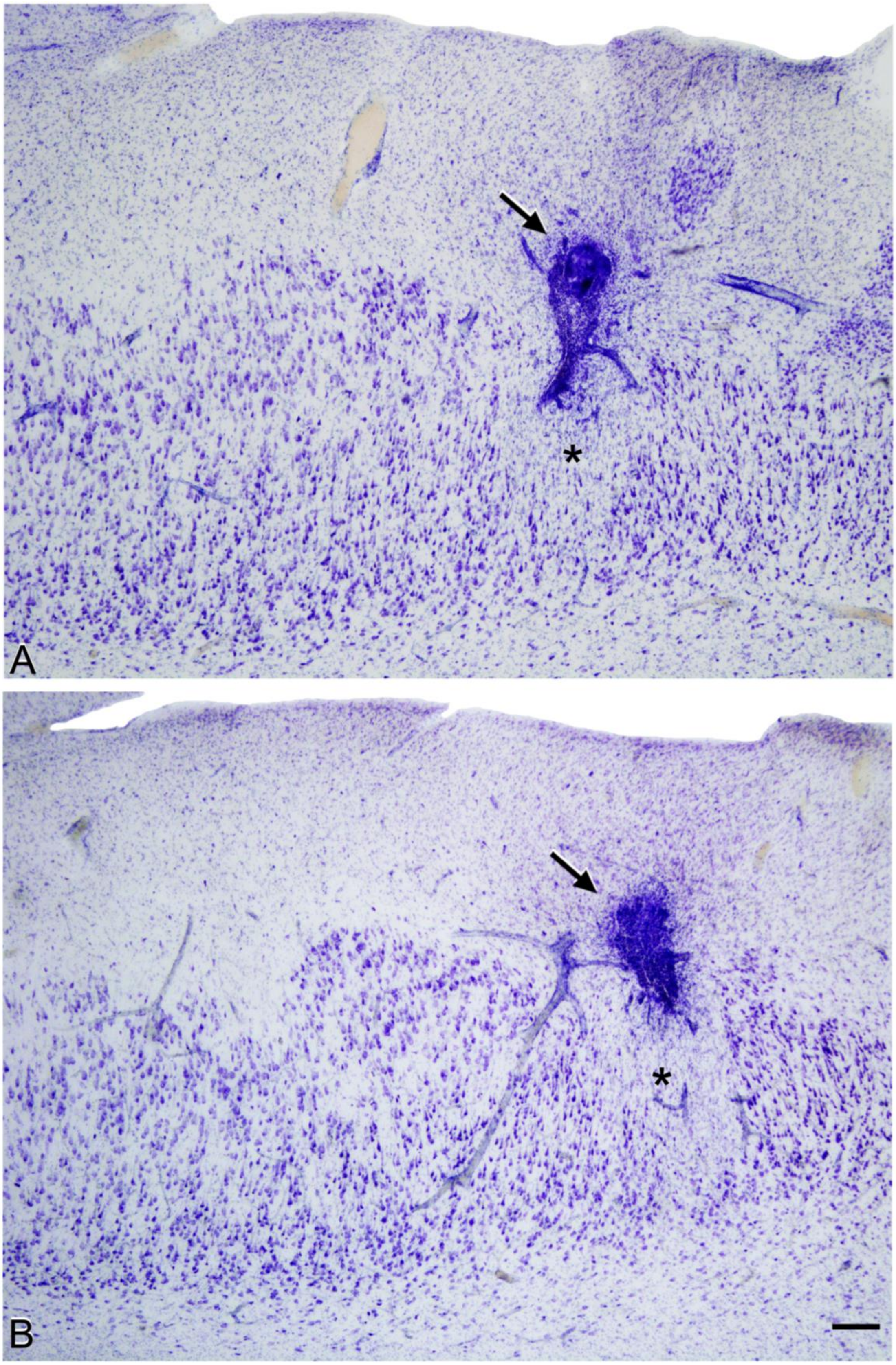

Figure 2-H123-3. **Photomicrographs of Nissl-stained sections**.
(**A**, **B**) Higher magnification of Figures 2-H123-1A and 2-H123-1B, respectively, showing the microvascular alteration (arrows). Note the neuronal loss (asterisks) below the microvascular alteration. Scale bar shown in (**B**) indicates 230 μm in (**A**) and (**B**).

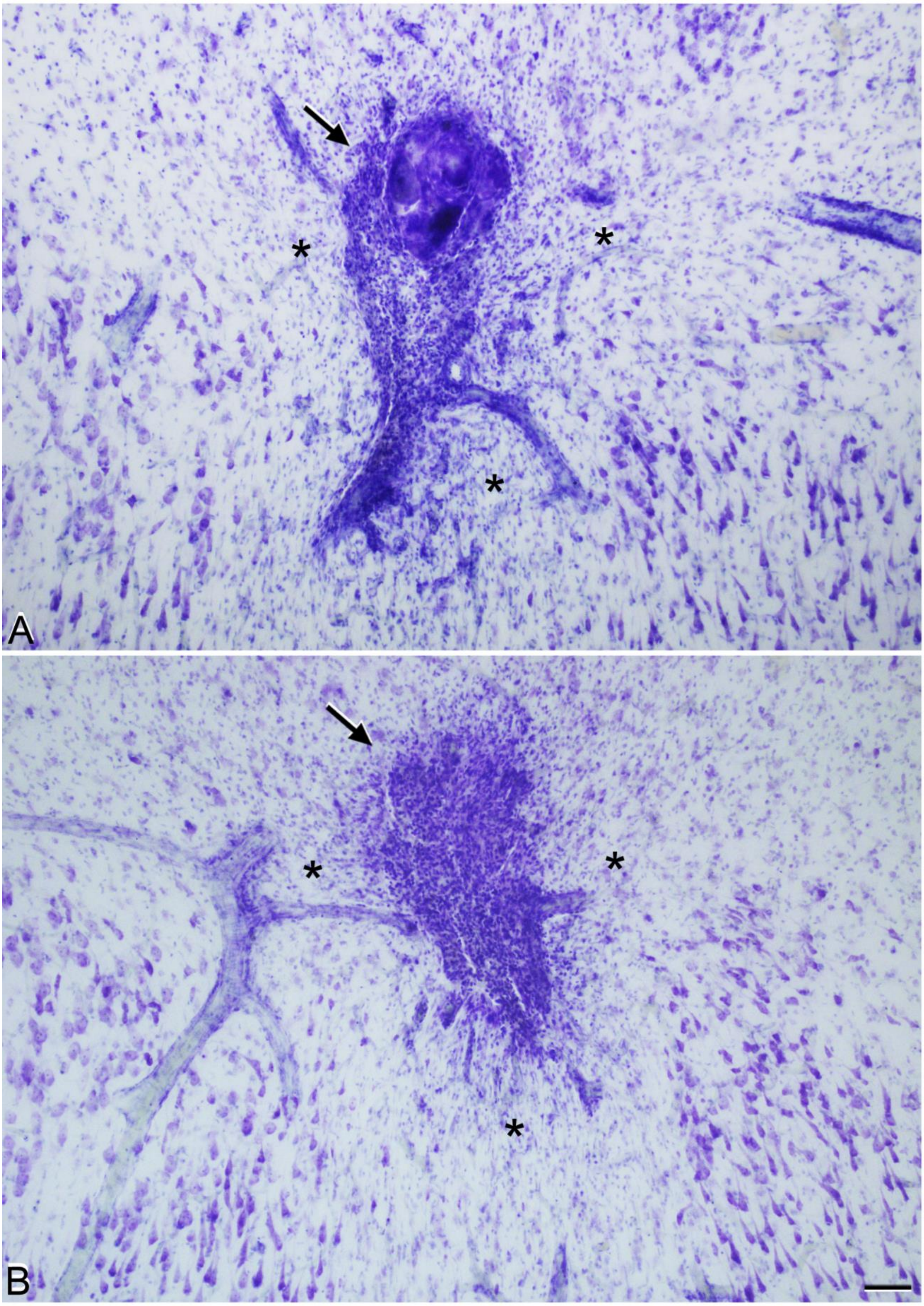

Figure 2-H123-4. **Photomicrographs of Nissl-stained sections**.

(**A**, **B**) Higher magnification of Figures 2-H123-3A and 2-H123-3B, respectively, showing the microvascular alteration (arrows). Note the neuronal loss (asterisks) around the microvascular alteration. Scale bar shown in (**B**) indicates 82 μm in (**A**) and (**B**).

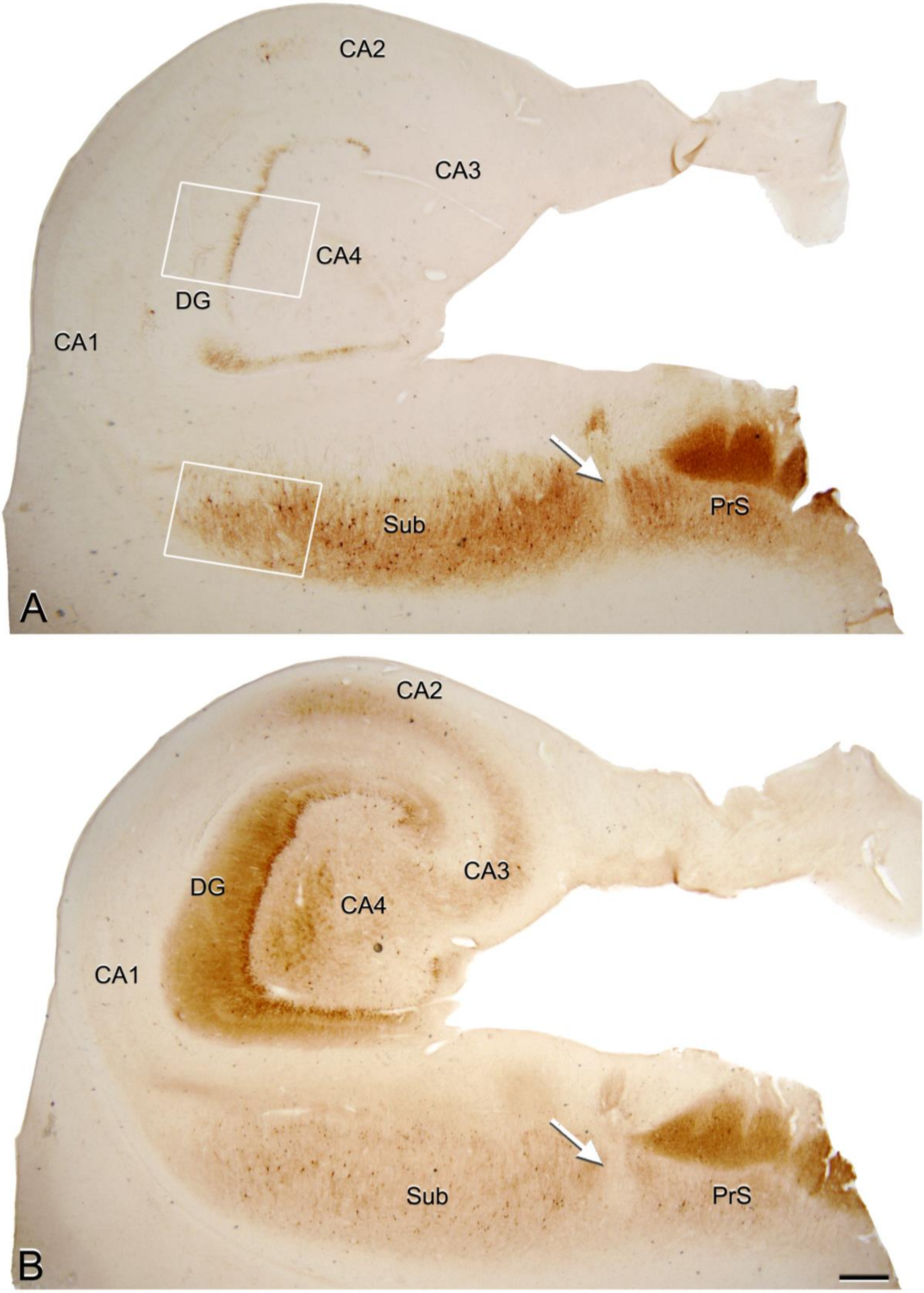

Figure 2-H123-5. **Photomicrographs of PV- and CalB-immunostained sections**.
(**A**, **B**) Photomicrographs of a PV- (**A**) and a CalB-immunostained (**B**) sections adjacent to the Nissl-stained sections showed in. Note the different patterns of staining for PV and CalB and the selective decrease of immunostaining. Arrows indicate a decrease of immunostaining for both PV and CalB in a small zone that corresponds to presence of the microvascular alteration indicates with white arrows in Figure 2-H123-1. Boxed areas in (**A**) indicate regions that are shown at higher magnification in Figure 3-H123-6. Scale bar shown in (**B**) indicates 600 µm in (**A**) and (**B**). CA1-CA4: Cornu ammonis fields; DG: dentate gyrus; Sub: subiculum; PrS: Presubiculum.

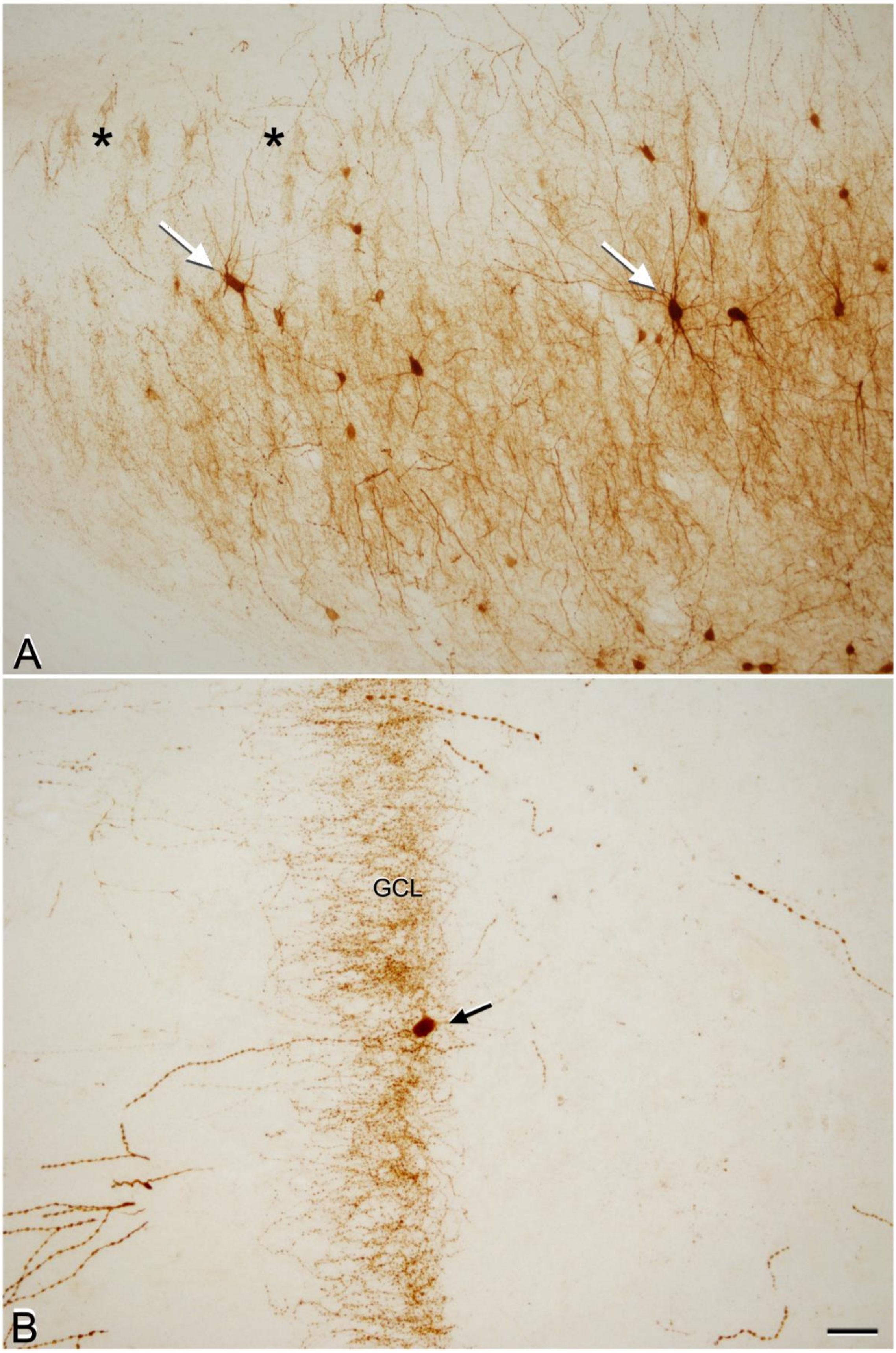

Figure 2-H123-6. **Photomicrographs of a PV-immunostained section**.
(**A**, **B**) Higher magnification of the boxed area of CA1 (**A**) and DG (**B**) in Figure 2-H123-5A, respectively. (**A**) Note the decrease in PV immunostaining at the superficial layer at the border CA1/subiculum (asterisks). White arrows indicate two large PV-immunostained interneurons. (**B**) Detail of the granular cell layer (GCL) of the dentate gyrus to illustrate the labeling in the neuropil. Black arrow indicates a PV-immunostained neuron. Scale bar shown in (**B**) indicates 100 µm in (**A**) and (**B**).

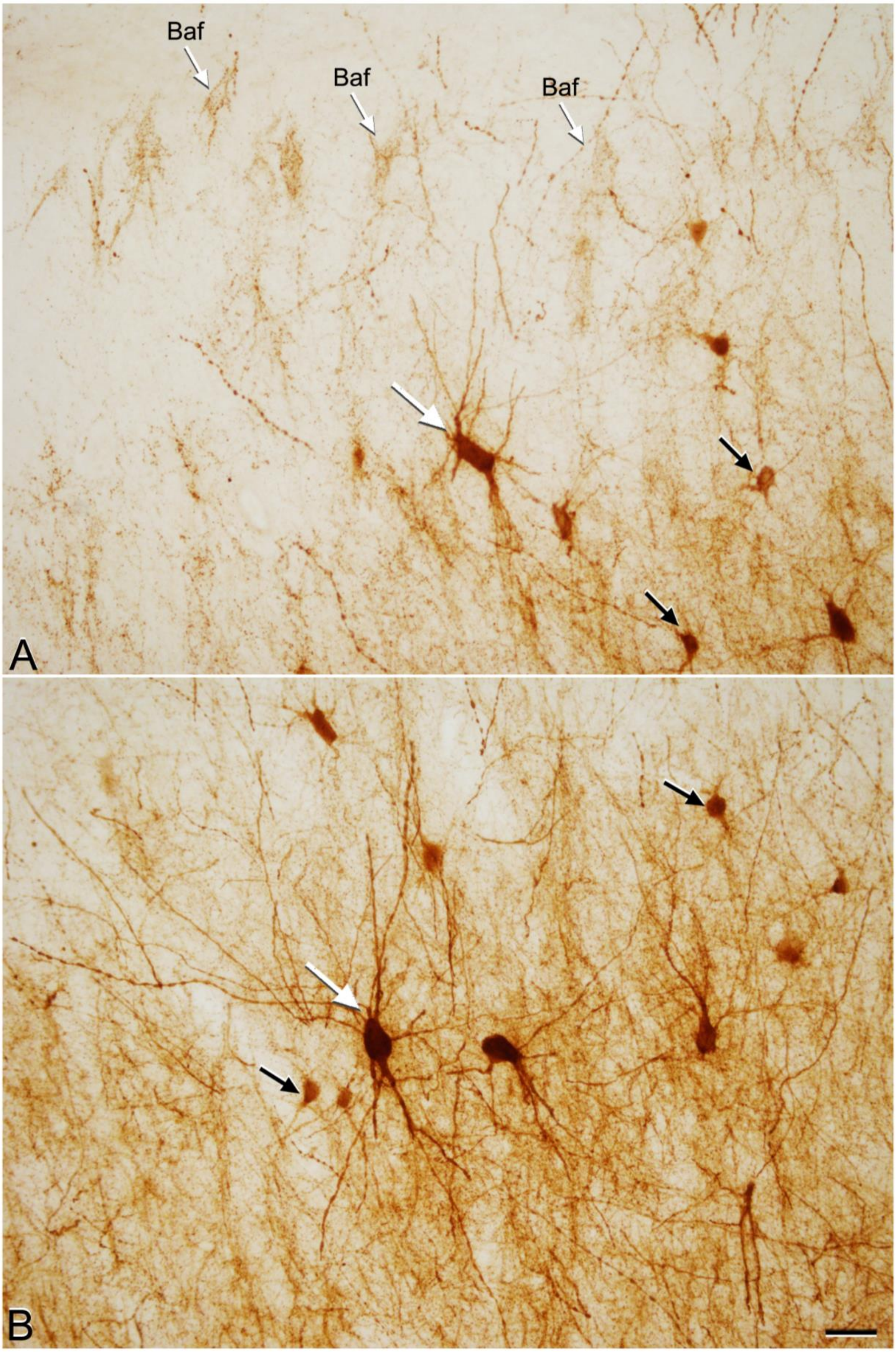

Figure S2-H123-7. **Photomicrographs of a PV-immunostained section**.
(**A**, **B**) Higher magnification of Figure 2-H123-6A. Basket formations (Baf) are selectively stained in the superficial layer of CA1/subiculum (**A**). The pattern of immunostaining in the neuropil is more homogeneous towards the subiculum (**B**). White arrows and black arrows indicate large and small PV-immunostained neurons, respectively. Scale bar shown in (**B**) indicates 50 μm in (**A**) and (**B**).

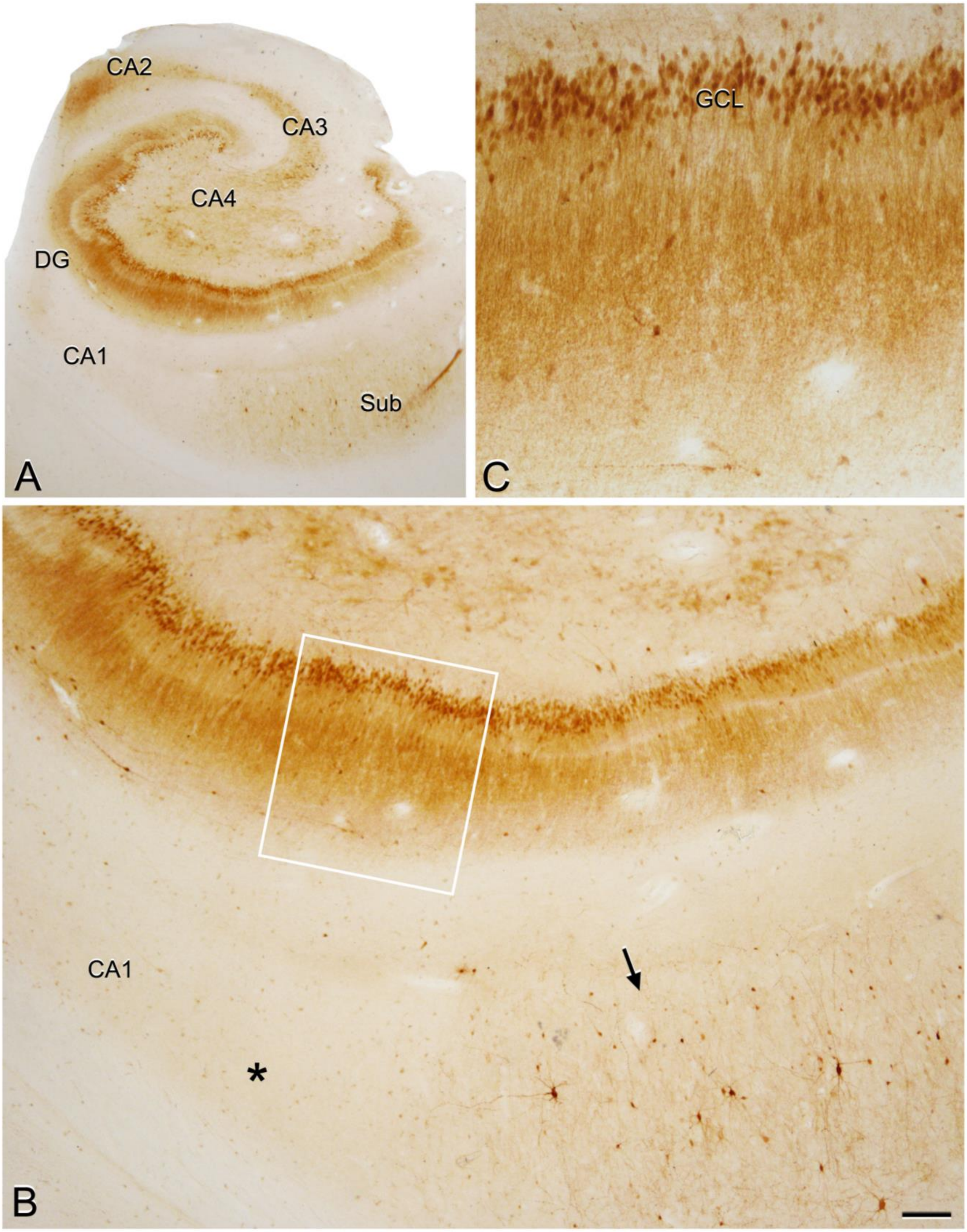

Figure S2-H123-8. **Photomicrographs of a CalB-immunostained section**.
(**A**) Low magnification photomicrograph of the hippocampal formation of an adjacent section to that shown Figure 2-H123-5B, to illustrate the pattern of CalB-immunostaining at a different level of the hippocampus. (**B)** Higher magnification of (**A**) to illustrate the decrease of immunostaining in CA1 (asterisk), whereas at the border between CA1 and subiculum is observed (arrow). (**C**) Higher magnification of the boxed area in (**B**). In the granule cell layer (GCL) numerous CalB-immunostained neurons are present. Scale bar shown in (**B**) indicates 700 µm in (**A**), 220 in (**B**) and 90 in (**C**). CA1-CA4: Cornu ammonis fields; DG: dentate gyrus; Sub: subiculum.
