# Supplementary material for: Neuroanatomical and psychological considerations in temporal lobe epilepsy
Source: Front Neuroanat. 2022 Dec 14;16:995286. doi: 10.3389/fnana.2022.995286 (PMC9794593; doi:10.3389/fnana.2022.995286)
Supplement: Supplementary file 1 [file Data_Sheet_1.zip › Supplementary material/Supplementary Figures 2/Supplementary Figures 2-H136.pdf]

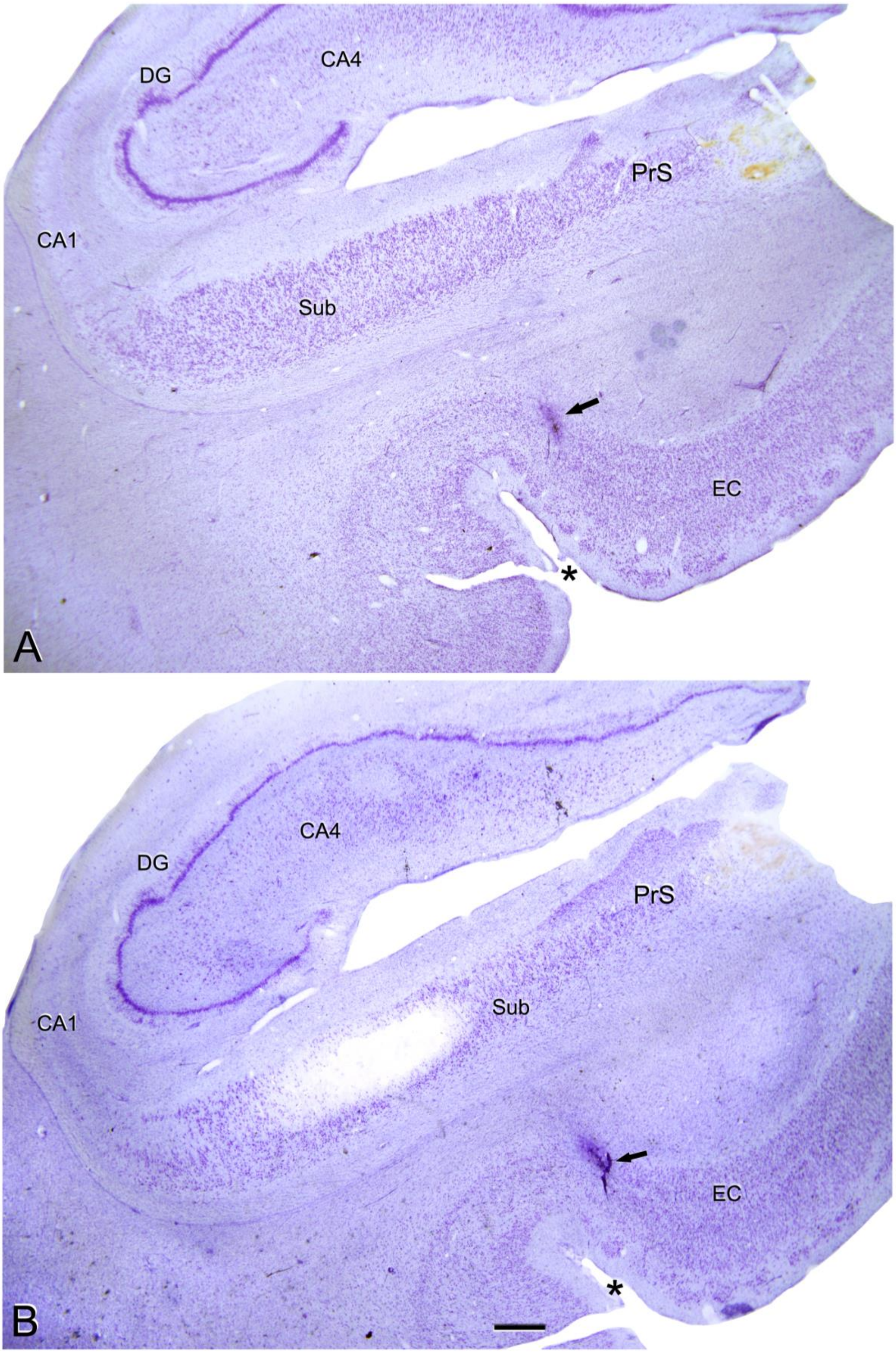

Figure 2-H136-1. **Photomicrographs of Nissl-stained sections**.
(**A**, **B**) Photomicrographs at low magnification showing at a rostral level the hippocampal formation in serial sections. Note the extensive loss of neurons in the CA1 and CA4 and dispersion of the granule cell layer of the DG. (**A**) microvascular alteration is observed in both sections (arrows). The one shown in (**B**) is also illustrated at higher magnification in Figure 2-H136-9. This microvascular alteration was followed in serial sections from the entorhinal cortex to deep to the white matter (see Figure 3-H136-2). Asterisk marks the collateral sulcus. Scale bar shown in (**B**) indicates 800 μm in (**A**) and (**B**). CA1-CA2: Cornu ammonis fields; DG: dentate gyrus; EC: Entorhinal cortex: Sub: subiculum. PrS: presubiculum.

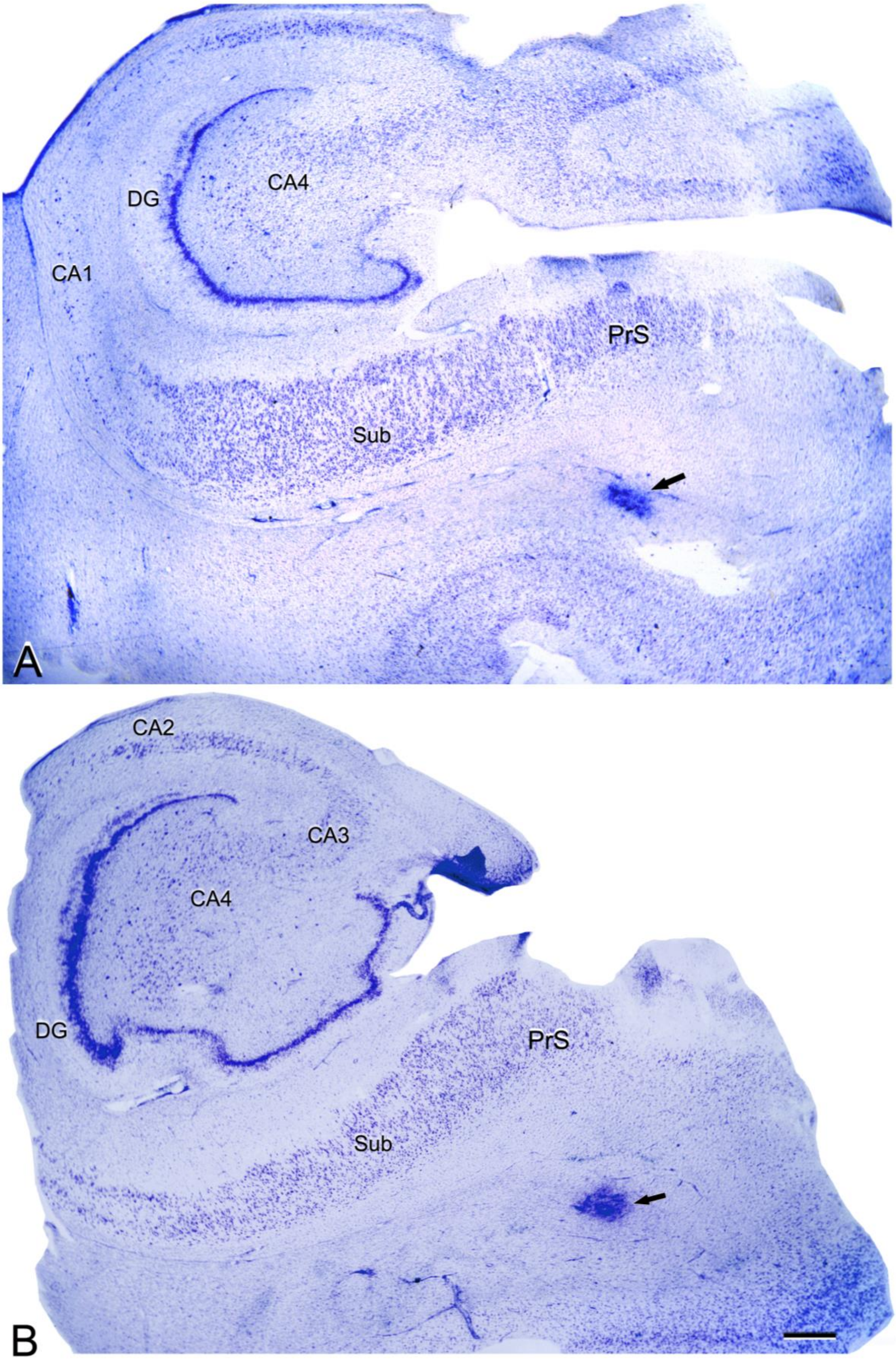

Figure 2-H136-2. **Photomicrographs of Nissl-stained sections**.
(**A**, **B**) Photomicrographs at low magnification showing the hippocampal formation in serial sections. Note the extensive loss of neurons in the CA1 and CA4 and dispersion of the granule cell layer of the DG. Arrows indicate a microvascular alteration in the white matter which was followed up to the entorhinal cortex (Figure 2-H136-1). Scale bar shown in (**B**) indicates 800 µm in (**A**) and (**B**). CA1-CA2: Cornu ammonis fields; DG: dentate gyrus; Sub: subiculum. PrS: presubiculum.

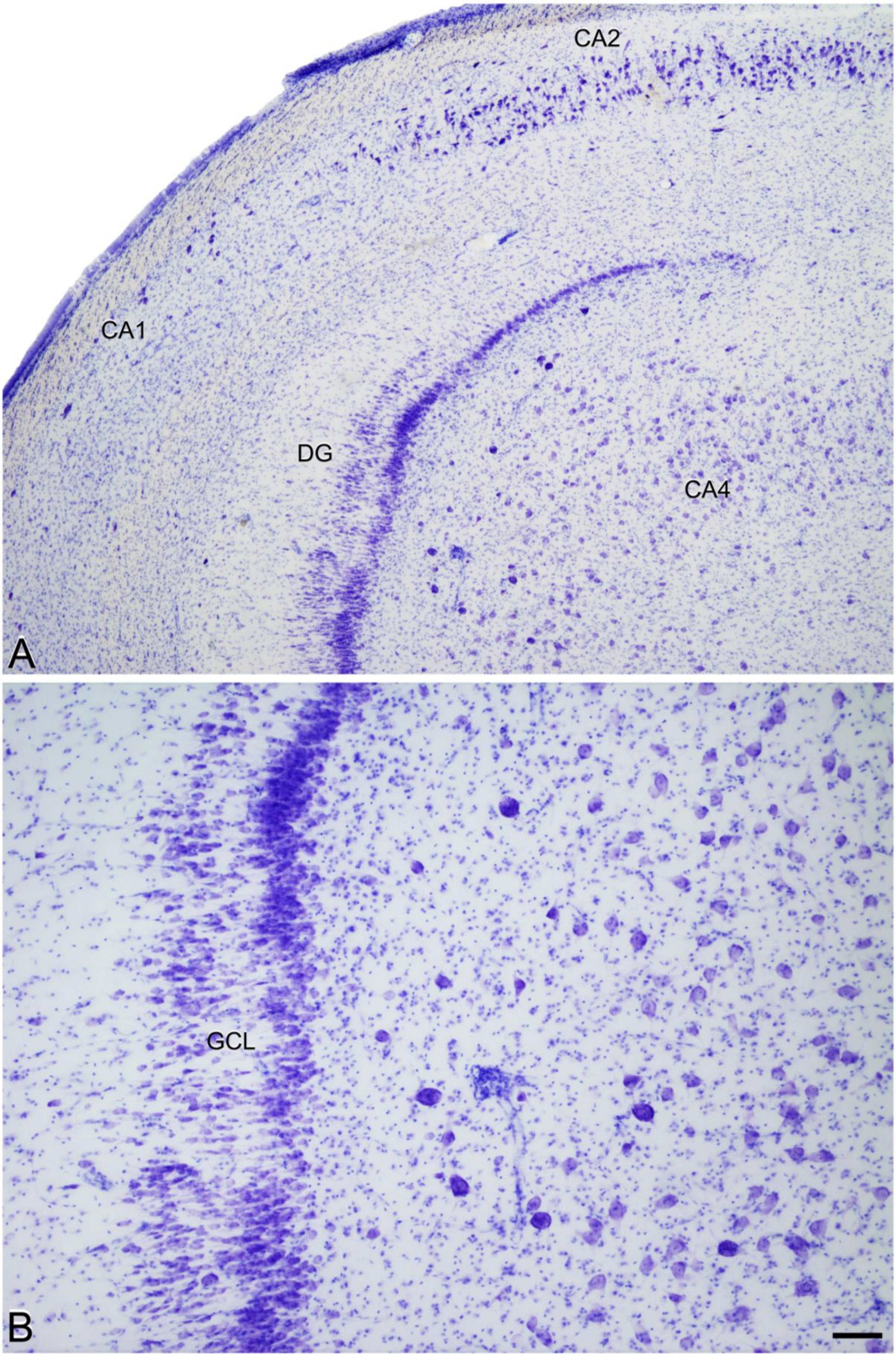

Figure 2-H136-3. **Photomicrographs of a Nissl-stained section**.
(**A**, **B**) Higher magnification Figure 2-H136-2A to illustrate with a greater detail CA4, CA1 and DG fields. Note the bi-laminar pattern of granule cell dispersion in the granule cell layer (GCL) of the DG. (**B)** is a higher magnification of (**A**). Scale bar shown in (**B**) indicates 240 μm in (**A**) and 100 μm in (**B**). CA1-CA4: Cornu ammonis fields; DG: dentate gyrus.

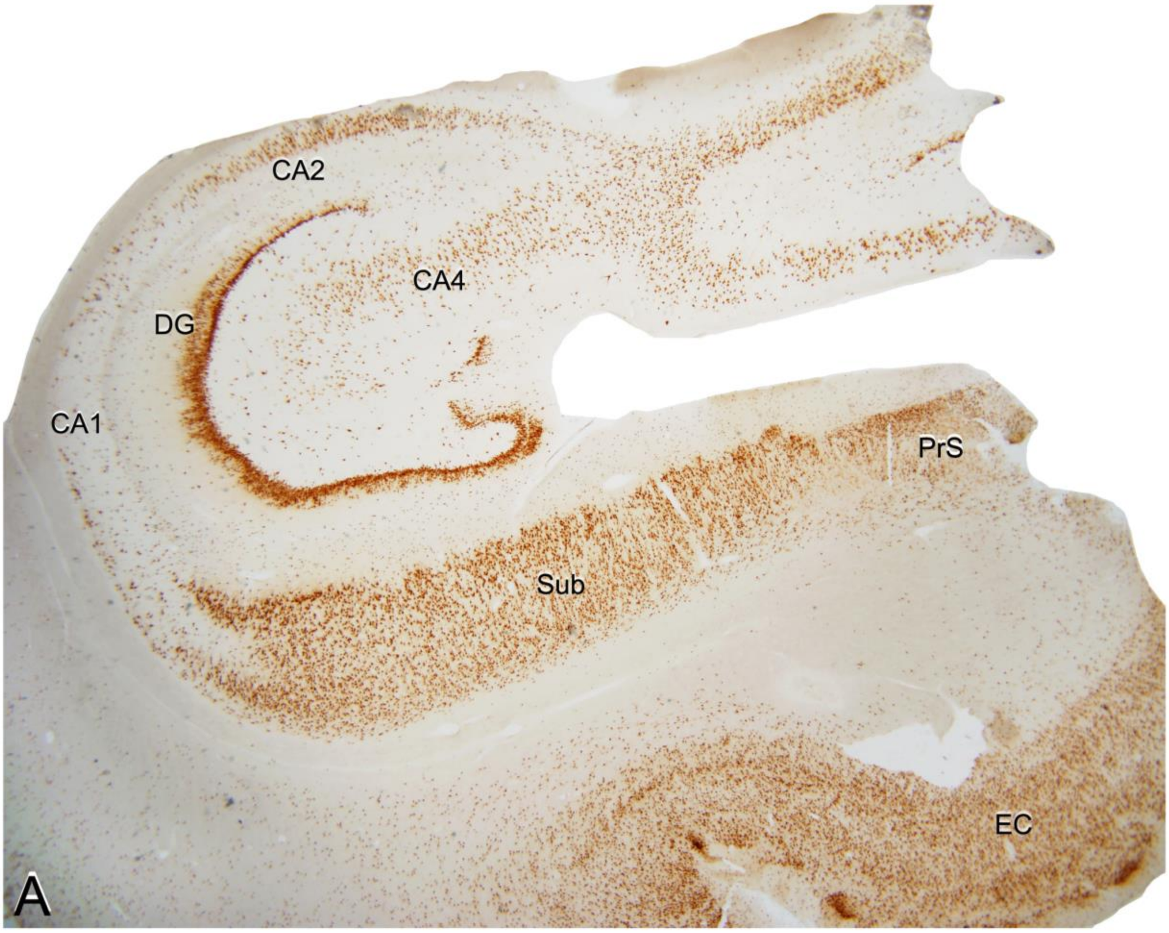

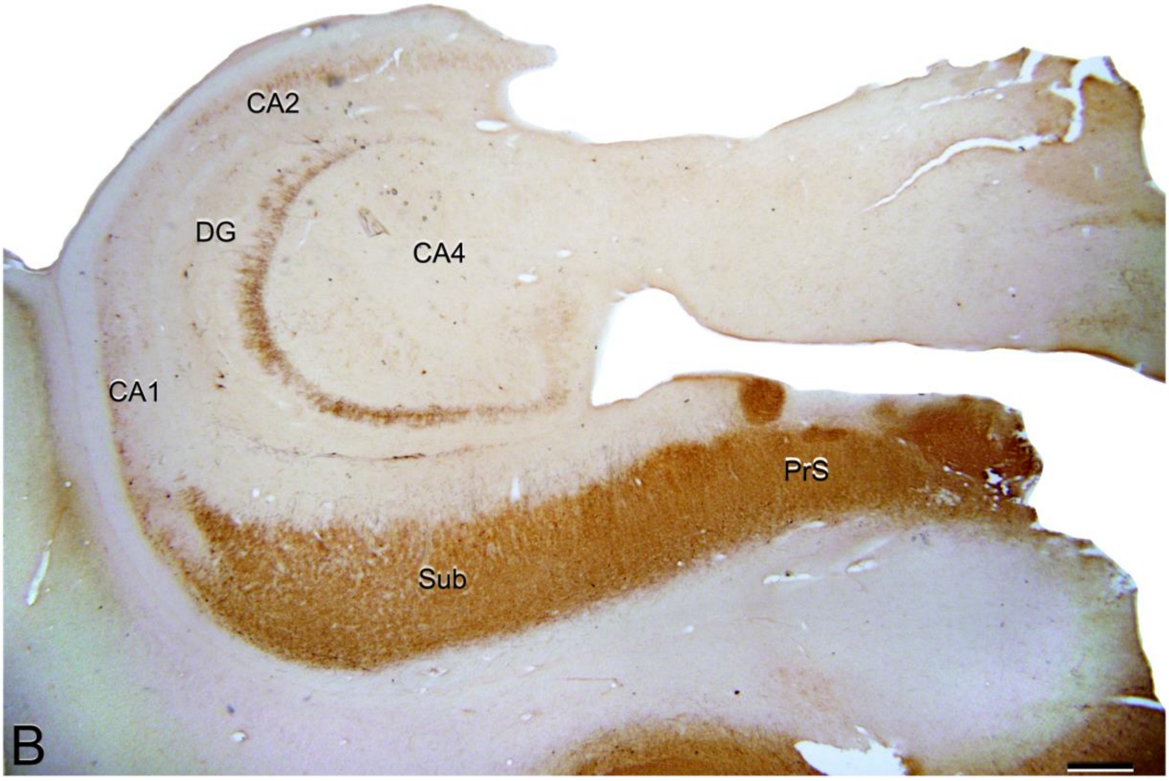

Figure 2-H136-4. **Photomicrographs of NeuN- and PV-immunostained sections**.
(**A**, **B**) Photomicrographs of NeuN- (**A**) and PV- (**B**) immunostained sections adjacent to the Nissl-stained section shown in Figure 2-H136-2A. Note the neuronal loss and the general reduction of PV immunostaining in all hippocampal fields. Scale bar shown in (**B**) indicates 750 µm in (**A**) and (**B**). CA1-CA4: Cornu ammonis fields; DG: dentate gyrus; EC: entorhinal cortex; Sub: subiculum. PrS: presubiculum.

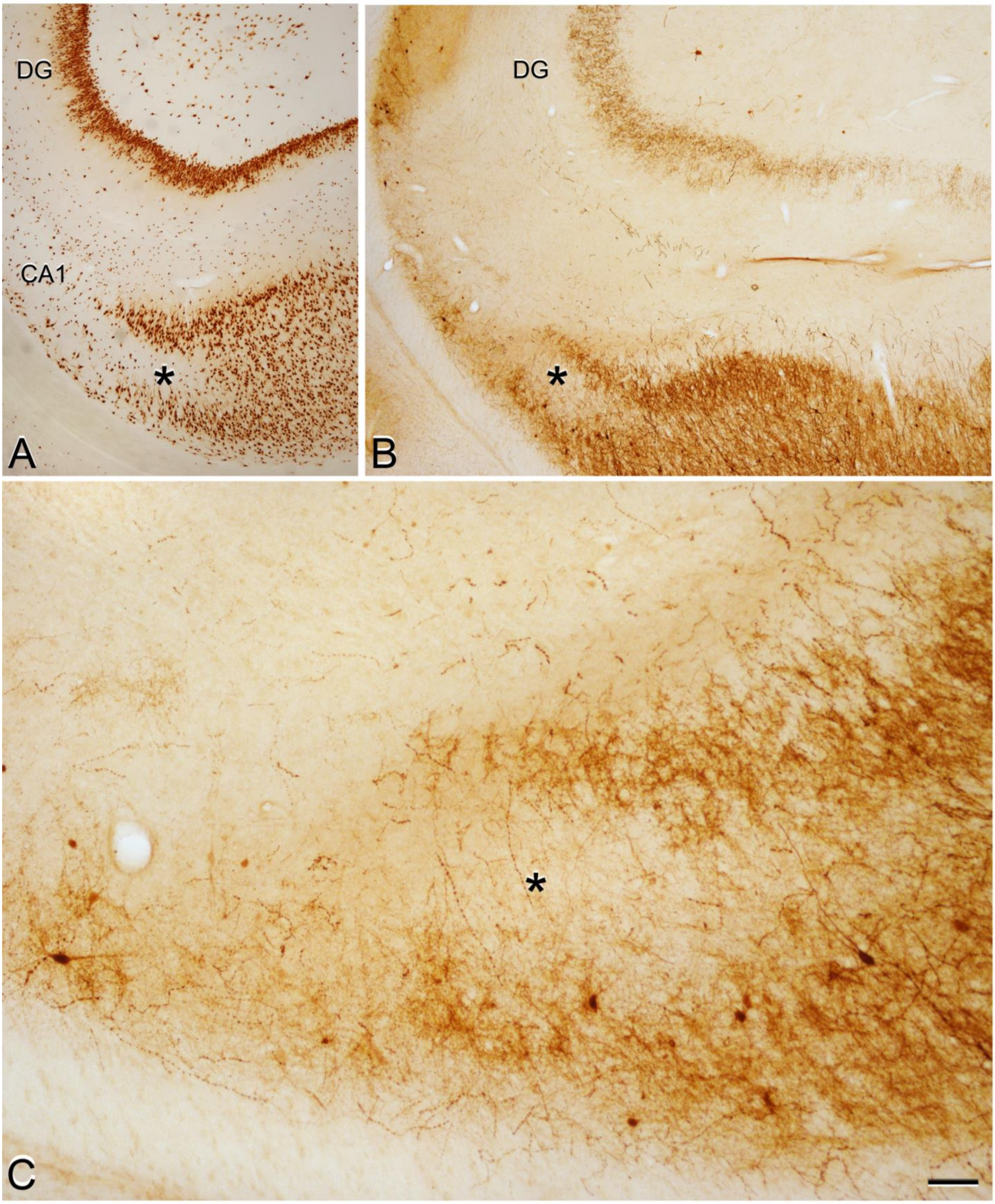

Figure 2-H136-5. Photomicrographs of NeuN- and PV-immunostained sections.
(**A**, **B**) Photomicrographs of NeuN- (**A**) and PV- (**B**) immunostained sections adjacent to the Nissl-stained section shown in Figure 2-H136-2A. Note the neuronal loss and the general reduction of PV immunostaining in all hippocampal fields. Scale bar shown in (**B**) indicates 750 μm in (**A**) and (**B**). CA1-CA4: Cornu ammonis fields; DG: dentate gyrus; Sub: subiculum.

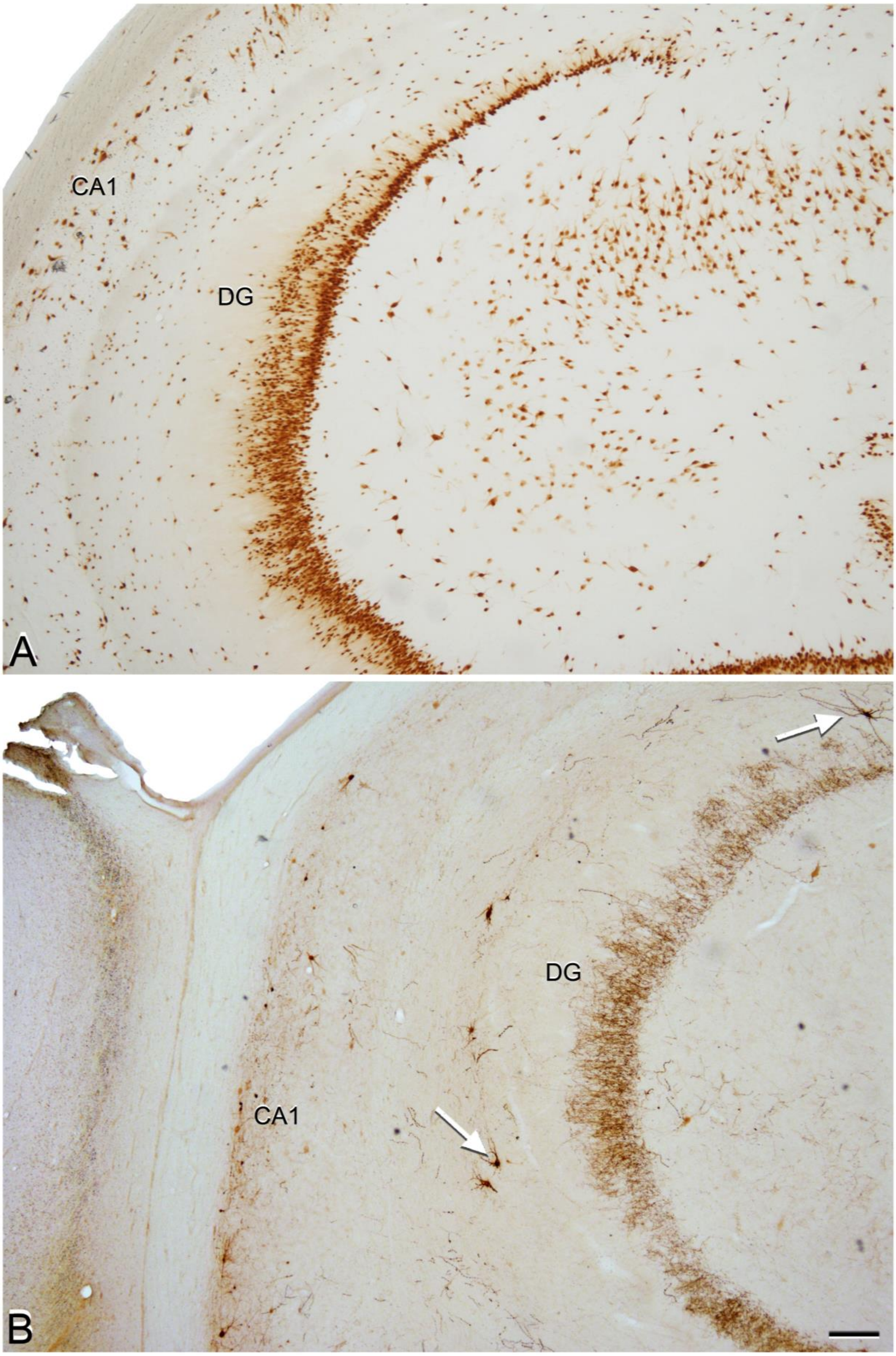

Figure 2-H136-6. **Photomicrographs of NeuN- and PV-immunostained sections**.
(**A**, **B**) Higher magnification of Figures 2-H136-4A and Figure 2-H136-4B to illustrate the pattern of immunostaining of NeuN (A) and PV (**B**) in CA1 and DG. Arrows in (**B**) indicates PV-immunostained neurons also shown at a higher magnification in Figure S3-H136-7. Scale bar shown in (**B**) indicates 250 μm in (**A**) and (**B**).

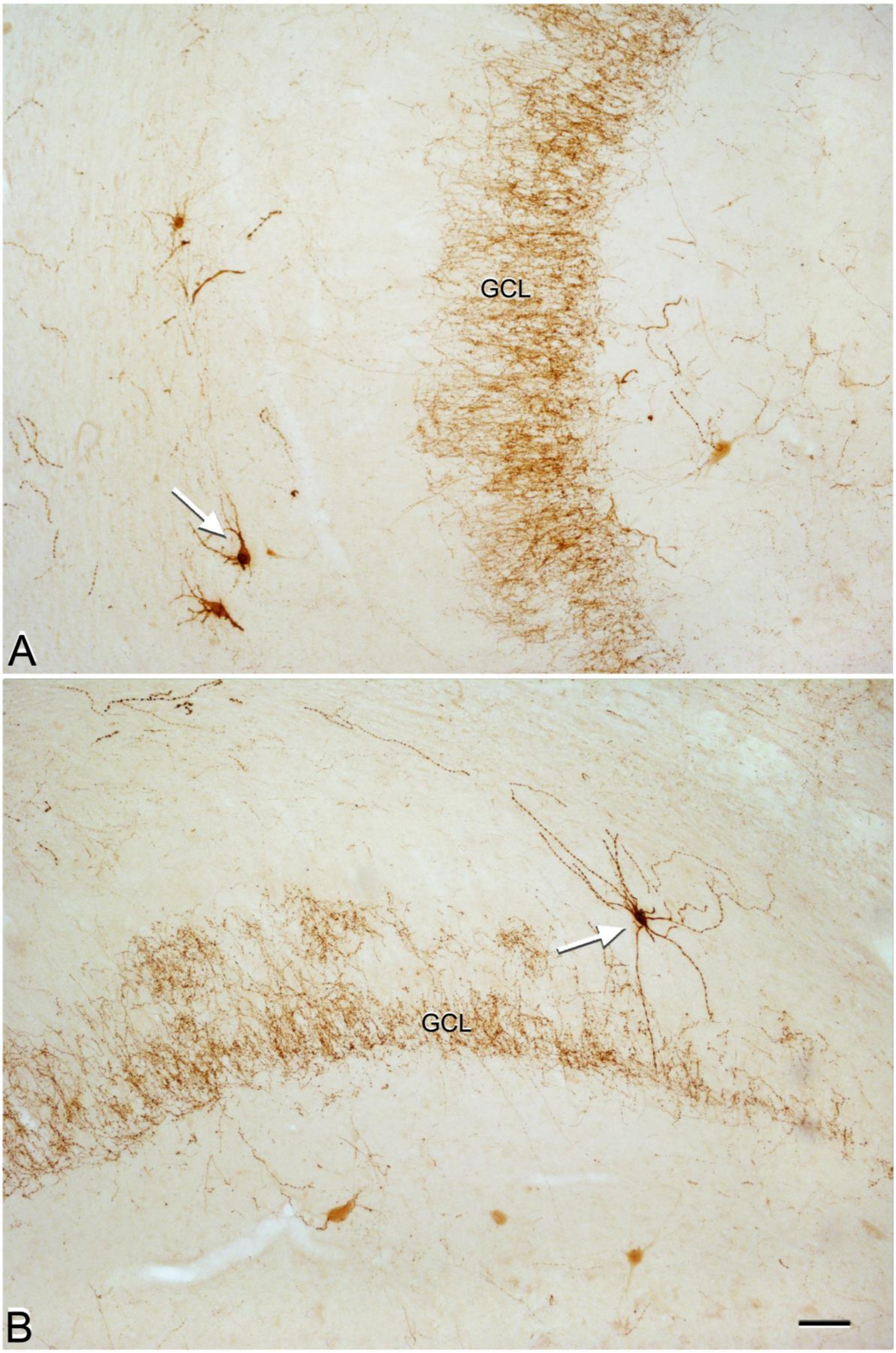

Figure 2-H136-7. **Photomicrographs of PV-immunostained sections**.
(**A**, **B**) Higher magnification of Figure 2-H136-B to illustrate the pattern of immunostaining of PV in the granule cell layer (GLC) of the DG. Arrows indicates two PV-immunostained interneurons also shown in Figure 2-H136-6B. Scale bar shown in (**B**) indicates 100 µm in (**A**) and (**B**).

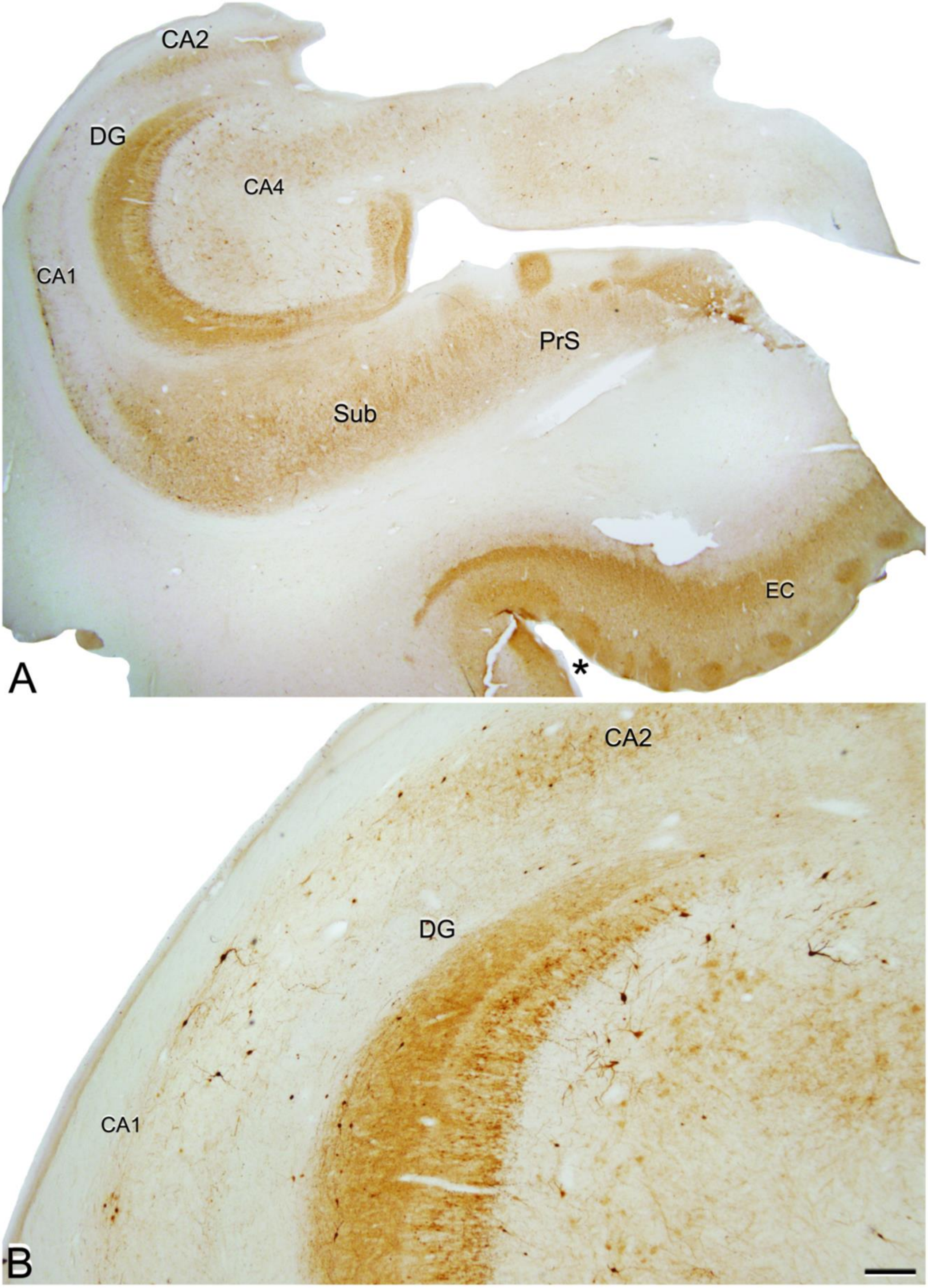

Figure 2-H136-7. **Photomicrographs of a CalB-immunostained section**.
(**A**, **B**) Low- and high-magnification photomicrographs, respectively, of a CalB-immunostained section adjacent to the Nissl-stained section showed in Figure 2-H136-2A. Note the differences in the intensity of labeling for CalB in the different hippocampal fields and the selective decrease in immunostaining. Scale bar shown in (**B**) indicates 750 µm in (**A**) and 240 µm in (**B**). CA1, CA2: Cornu ammonis fields; DG: dentate gyrus; EC: entorhinal cortex; Prs: presubiculum; Sub: subiculum; PrS: presubiculum.

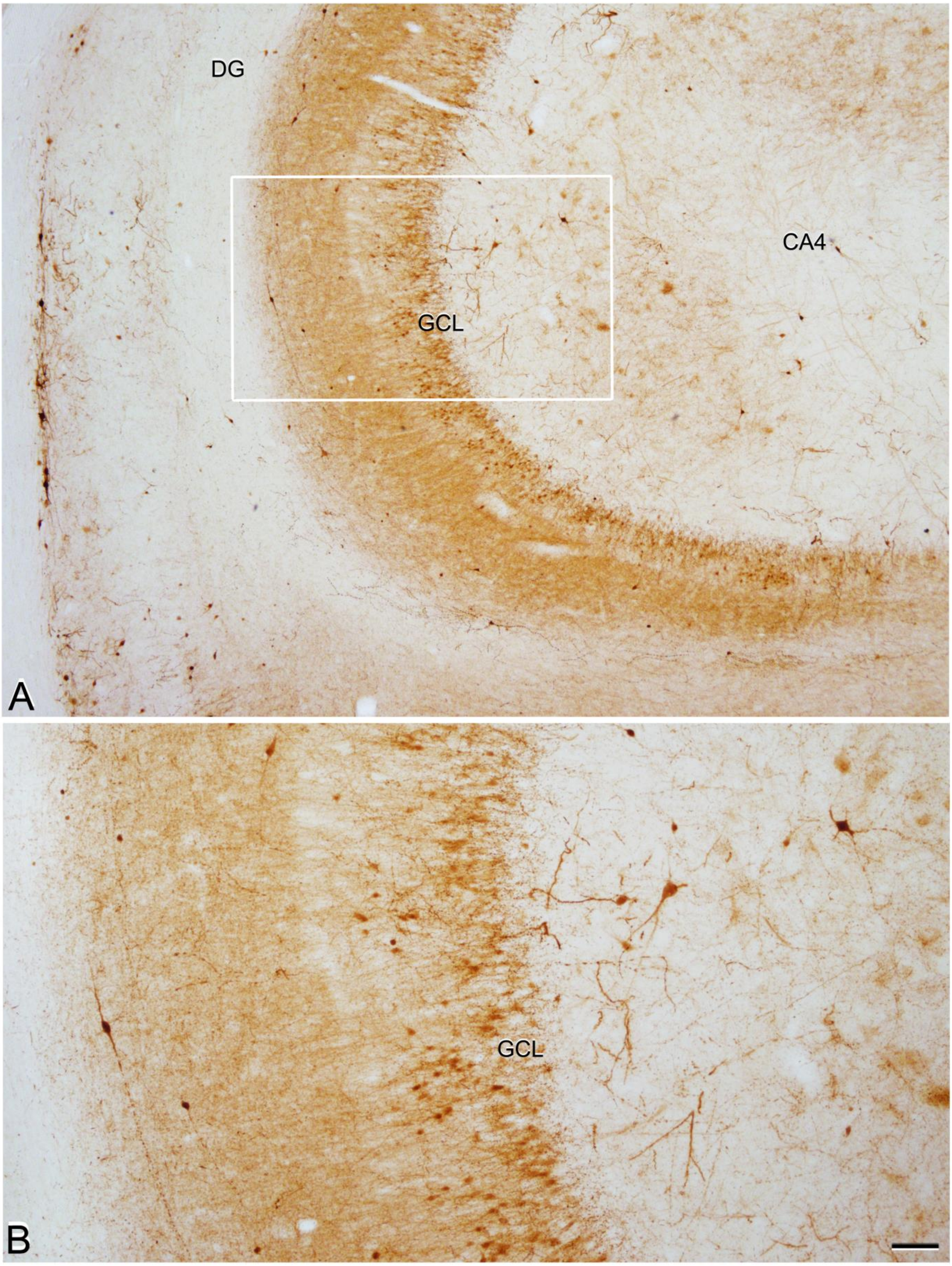

Figure 2-H136-8. **Photomicrographs of a CalB-immunostained section**.
(**A**, **B**) Low- and high-magnification photomicrographs, respectively, of the CalB-immunostained section showed in Figure 2-136-7A at the level of the DG. (**B**) Higher magnification of the boxed area in (**A**). Numerous CalB-immunostained neurons are seen in the granule cell layer (GCL) of the DG. Scale bar shown in (**B**) indicates 220 μm in (**A**) and 85 μm in (**B**).

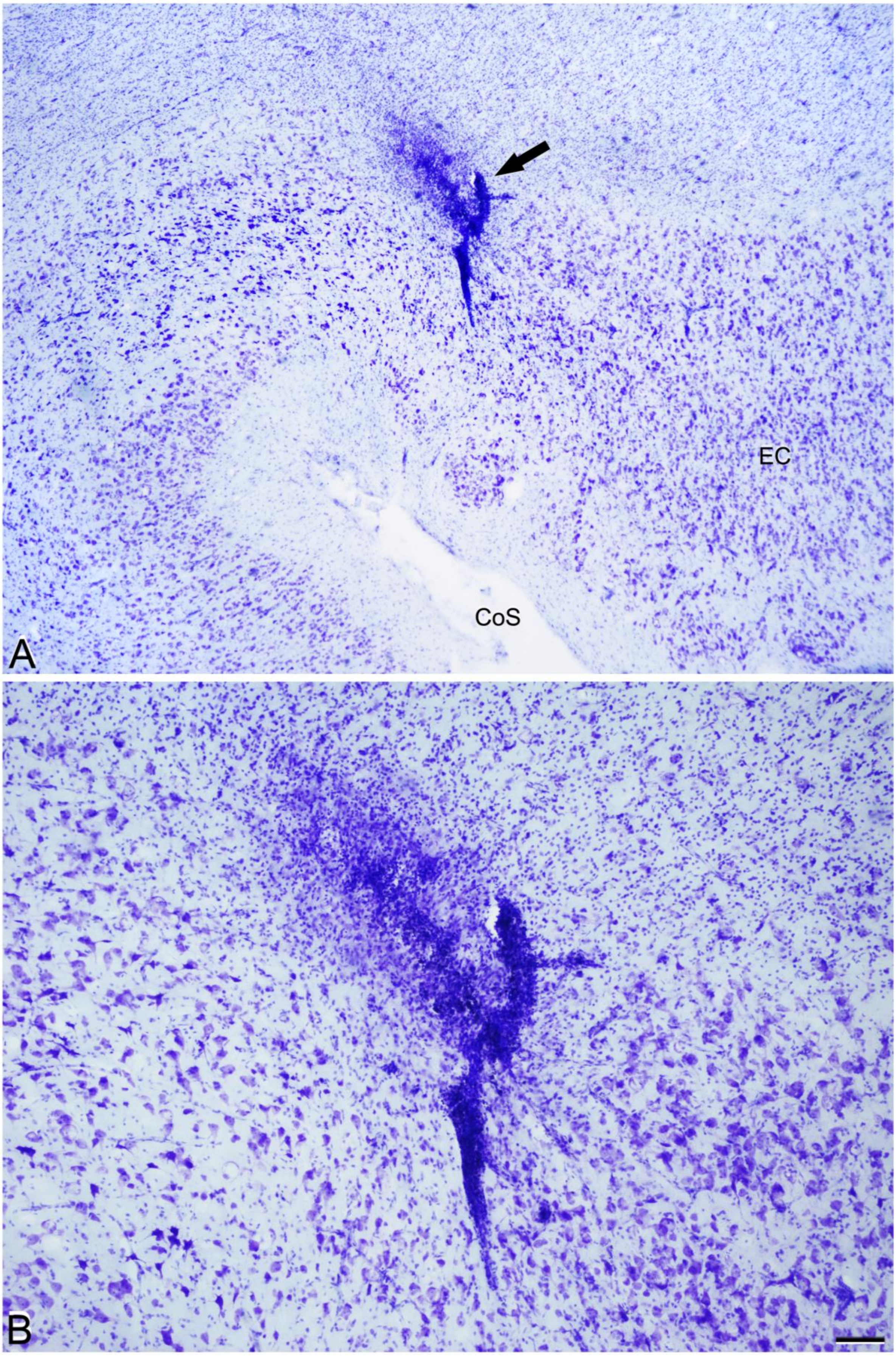

Figure 2-H136-9. **Photomicrographs of a Nissl-stained section.**
(**A**, **B**) Photomicrographs of the microvascular alteration also shown at low magnification in Figure 2-136-1B. The lesion is affecting to a small region of the white matter and deep cortical layers of the entorhinal cortex (EC). Scale bar shown in (**B**) indicates 240 μm in (**A**) and 95 μm in (**B**). Cos: collateral sulcus.

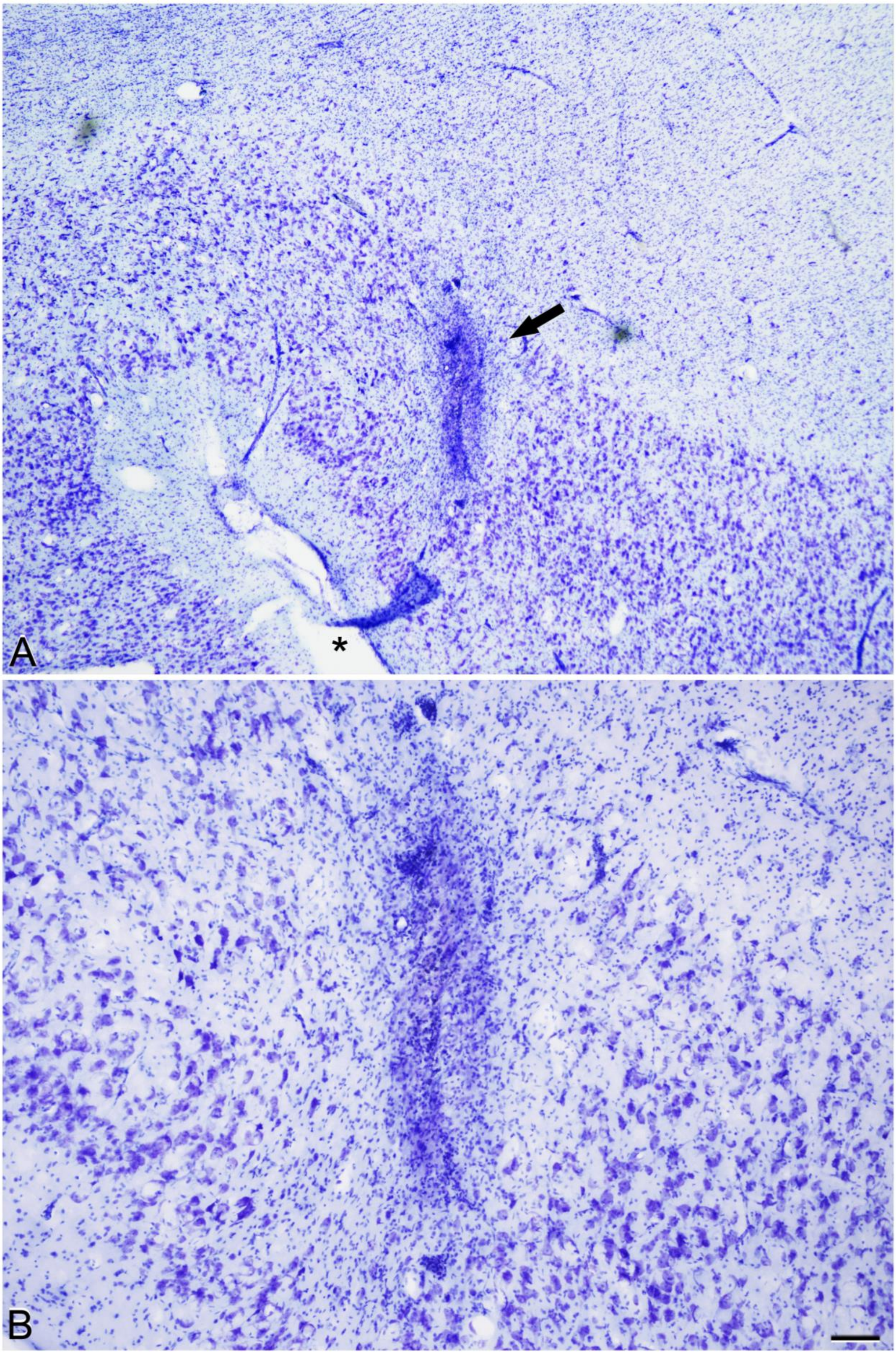

Figure 2-H136-10. **Photomicrographs of a Nissl-stained section.**
(**A, B)** Photomicrographs of the same microvascular alteration (arrow) shown in Figure 2-H136-9 in a more rostral section. The lesion is affecting to all cortical layers of the entorhinal cortex (EC). Note the loss of neurons around the lesion. Asterisk marks the collateral sulcus. Scale bar shown in (**B**) indicates 240 μm in (**A**) and 95 μm in (**B**).

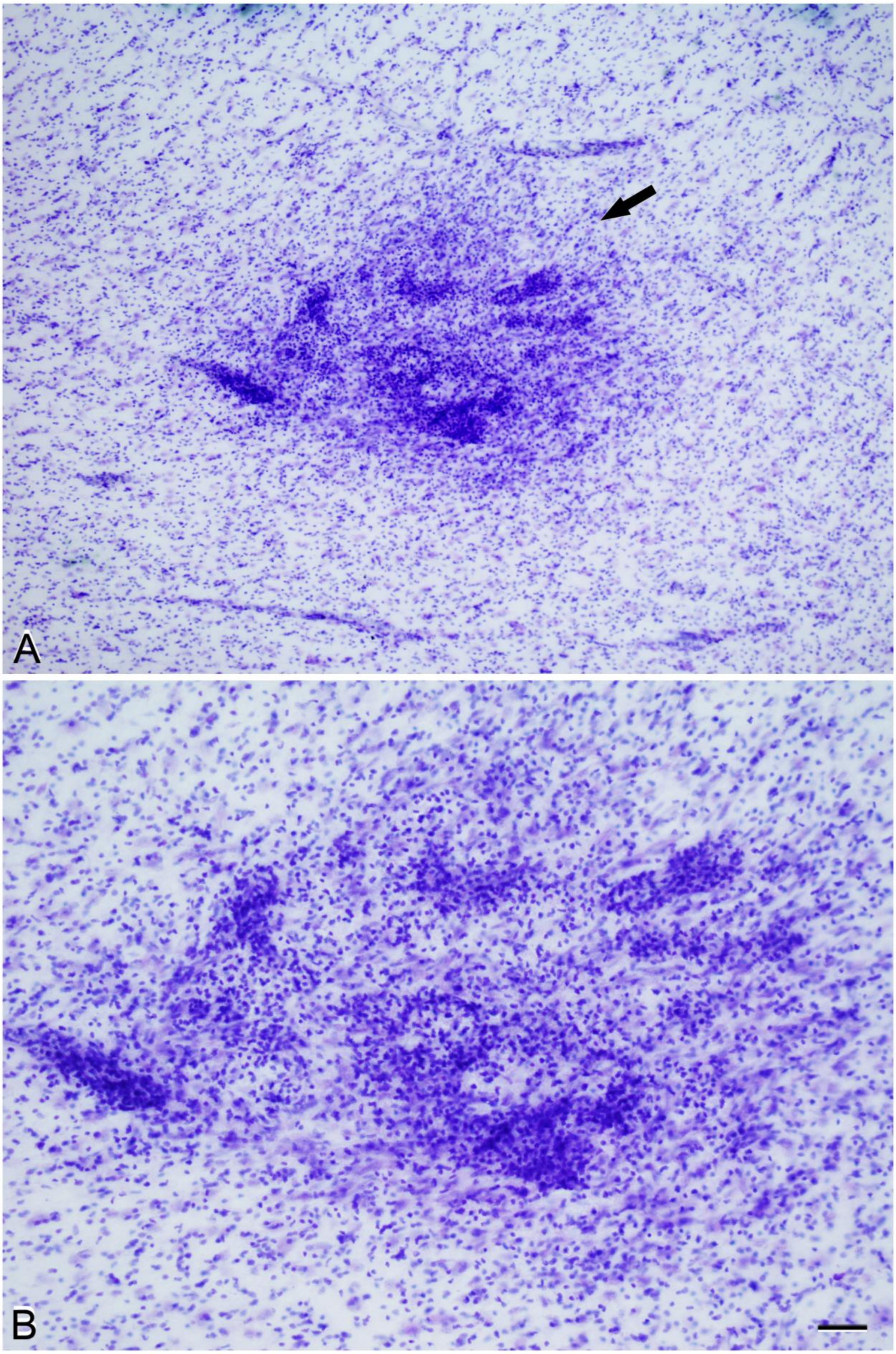

Figure 2-H136-11. **Photomicrographs of a Nissl-stained section.**
(**A**, **B**) Higher magnification photomicrographs of the microvascular alteration shown at low magnification in Figure 2-136-2B. At this level, the lesion is affecting to a small region of the white matter. Scale bar shown in (**B**) indicates 95 µm in (**A**) and 50 µm in (**B**).

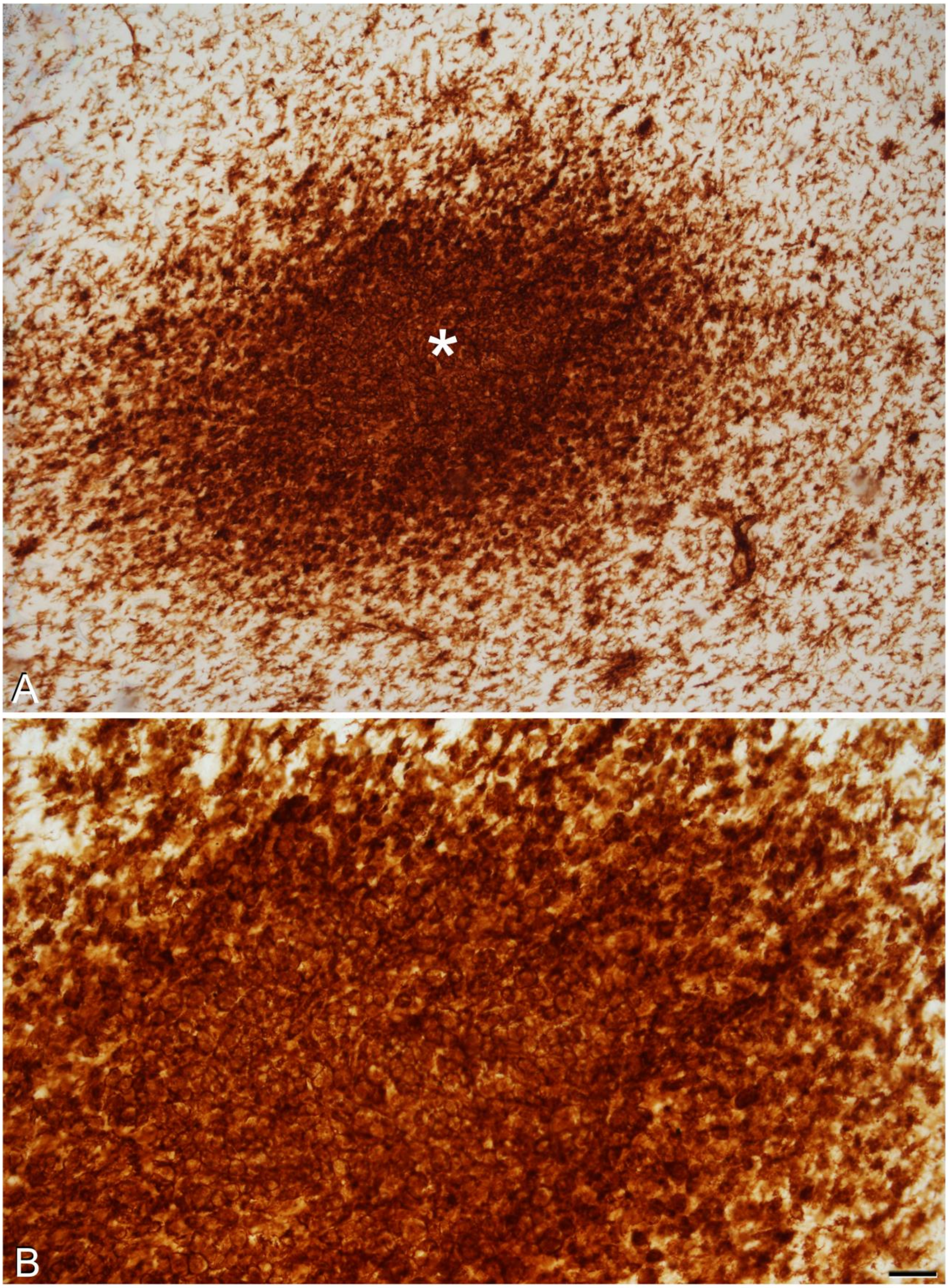

Figure 2-H136-12. **Photomicrographs of a section immunocytochemically stained for HLA-DR**
(**A**, **B**) Low- and high-power photomicrographs of a immunocytochemically stained section for HLA-DR to visualize microglia cells. This section adjacent to the section stained for Nissl shown in Figure 2-H136-11. The microvascular alteration (asterisk) shows perivascular inflammatory cells some of then identified as rounded amoeboid microglia y foamy (lipid laden) macrophages (for a higher magnification and further details see Figure 2-H136-13). Scale bar shown in (**B**) indicates 95 µm in (**A**) and 50 µm in (**B**).

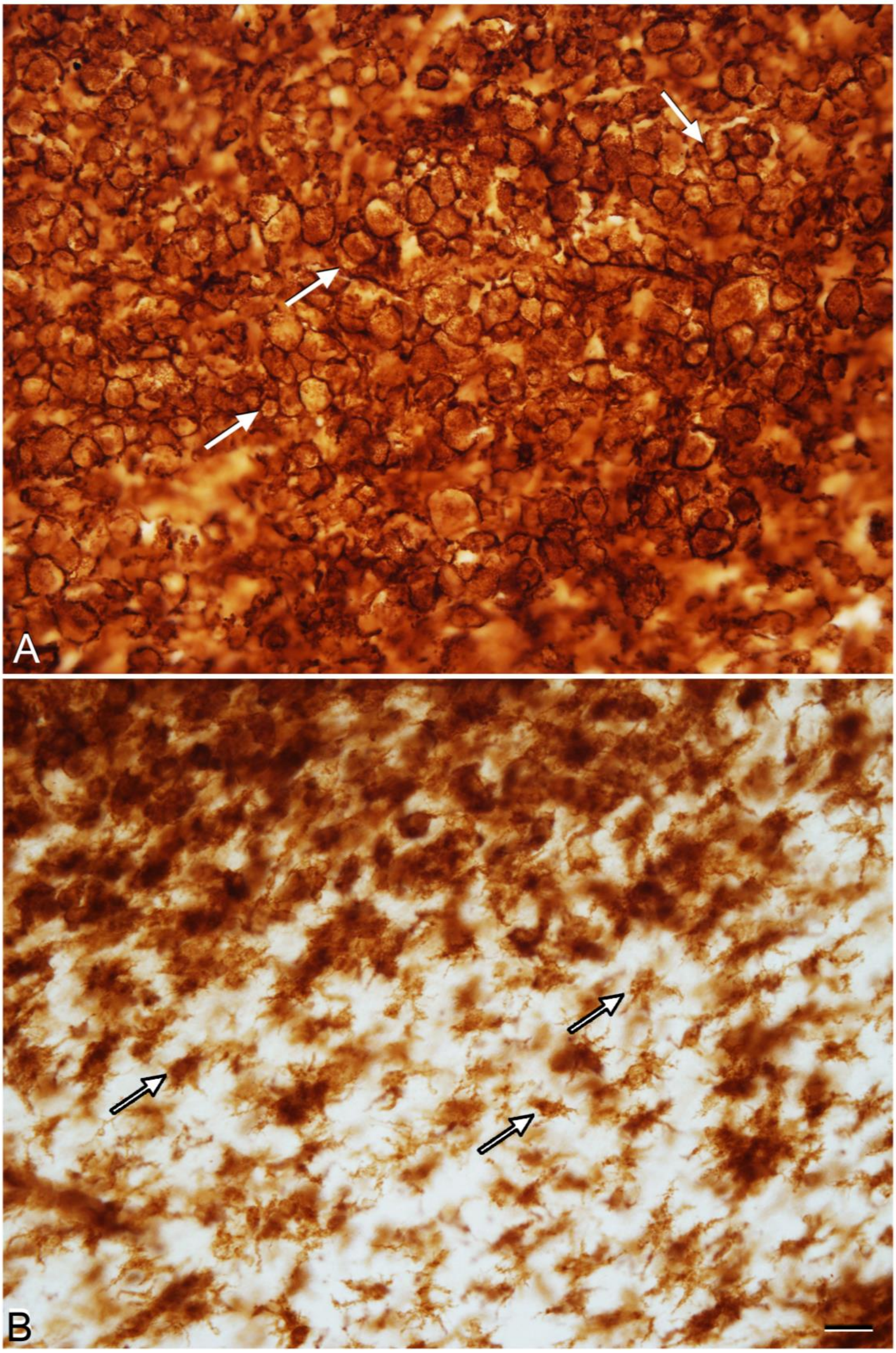

Figure 2-H136-13. **Photomicrographs of a section immunocytochemically stained for HLA-DR**

(**A**, **B**) Higher magnification Figure 2-H136-12, immunocytochemically stained for HLA-DR to visualize microglia cells. Arrows in (**A**) indicate some foamy (lipid laden) macrophages. Arrows in (**B**) indicate some rounded amoeboid microglia cells in the periphery of the lesion. In the normal brain tissue HLA-DR–immunoreactive microglia cells are of small size and possess numerous thin branching processes (resting microglia) (for example, see Hendrickx *et al*., 2017). Scale bar shown in (**B**) indicates 25 μm in (**A**) and (**B**).
