# Supplementary material for: Neuroanatomical and psychological considerations in temporal lobe epilepsy
Source: Front Neuroanat. 2022 Dec 14;16:995286. doi: 10.3389/fnana.2022.995286 (PMC9794593; doi:10.3389/fnana.2022.995286)
Supplement: Supplementary file 1 [file Data_Sheet_1.zip › Supplementary material/Supplementary Figures 2/Supplementary Figures 2-H138.pdf]

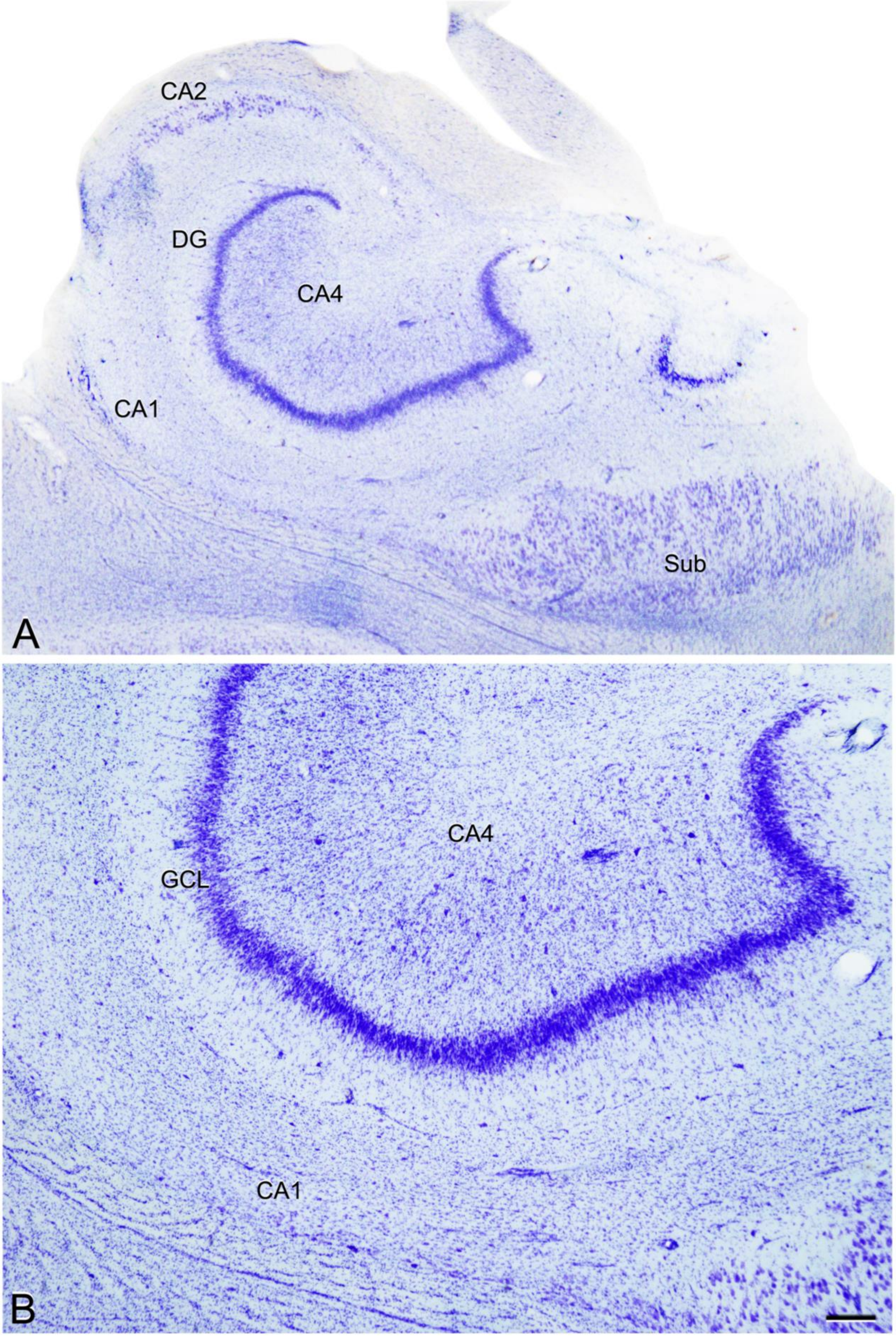

Figure 2-H138-1. **Photomicrographs of a Nissl-stained section**.
(**A**, **B**) Photomicrographs showing the hippocampal formation at low (**A**) and high magnification (**B**). Note the extensive loss of neurons in CA1 and CA4 and the dispersion of the granule cell layer (GCL) of the dentate gyrus. Scale bar shown in (**B**) indicates 500 μm in (**A**) and 240 μm in (**B**). CA1-CA2: Cornu ammonis fields; DG: dentate gyrus; Sub: subiculum.

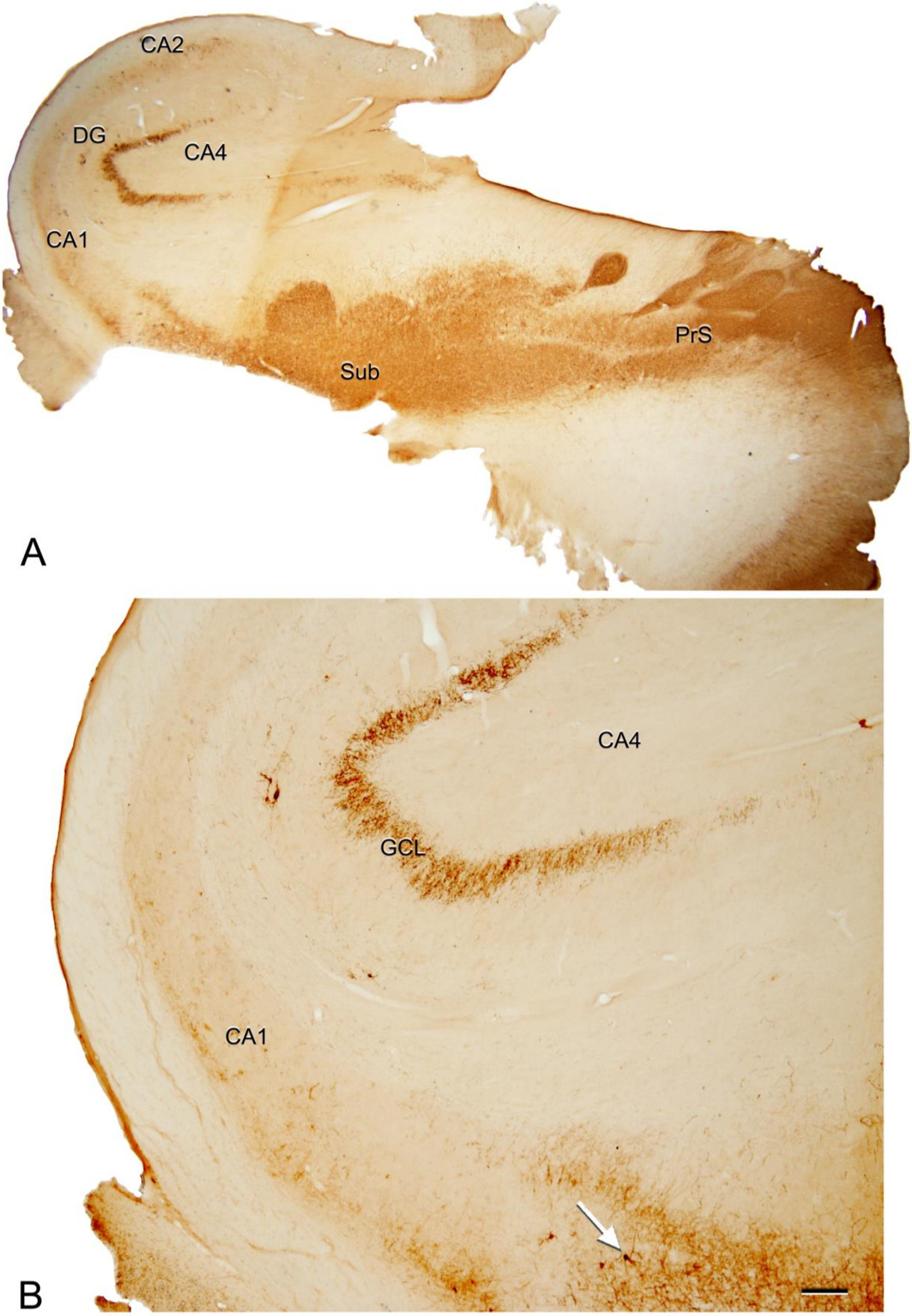

Figure 2-H138-2. **Photomicrographs of a PV-immunostained section**.

(**A**, **B**) Photomicrographs of a PV-immunostained section showing the hippocampal formation at low (**A**) and high magnification (**B**). Note the general reduction of PV immunostaining in all hippocampal fields, except in a small segment of the DG which shows a relatively intense labeling of the neuropil in the granule cell layer (GCL) of the dentate gyrus. Arrow in (**B**) indicates a PV-immunostained neuron also show at a higher magnification in Figure 2-H138-3A. Scale bar shown in (**B**) indicates 670 μm in (**A**) and 210 μm in (**B**). CA1-CA4: Cornu ammonis fields; DG: dentate gyrus; Sub: subiculum; PrS: presubiculum.

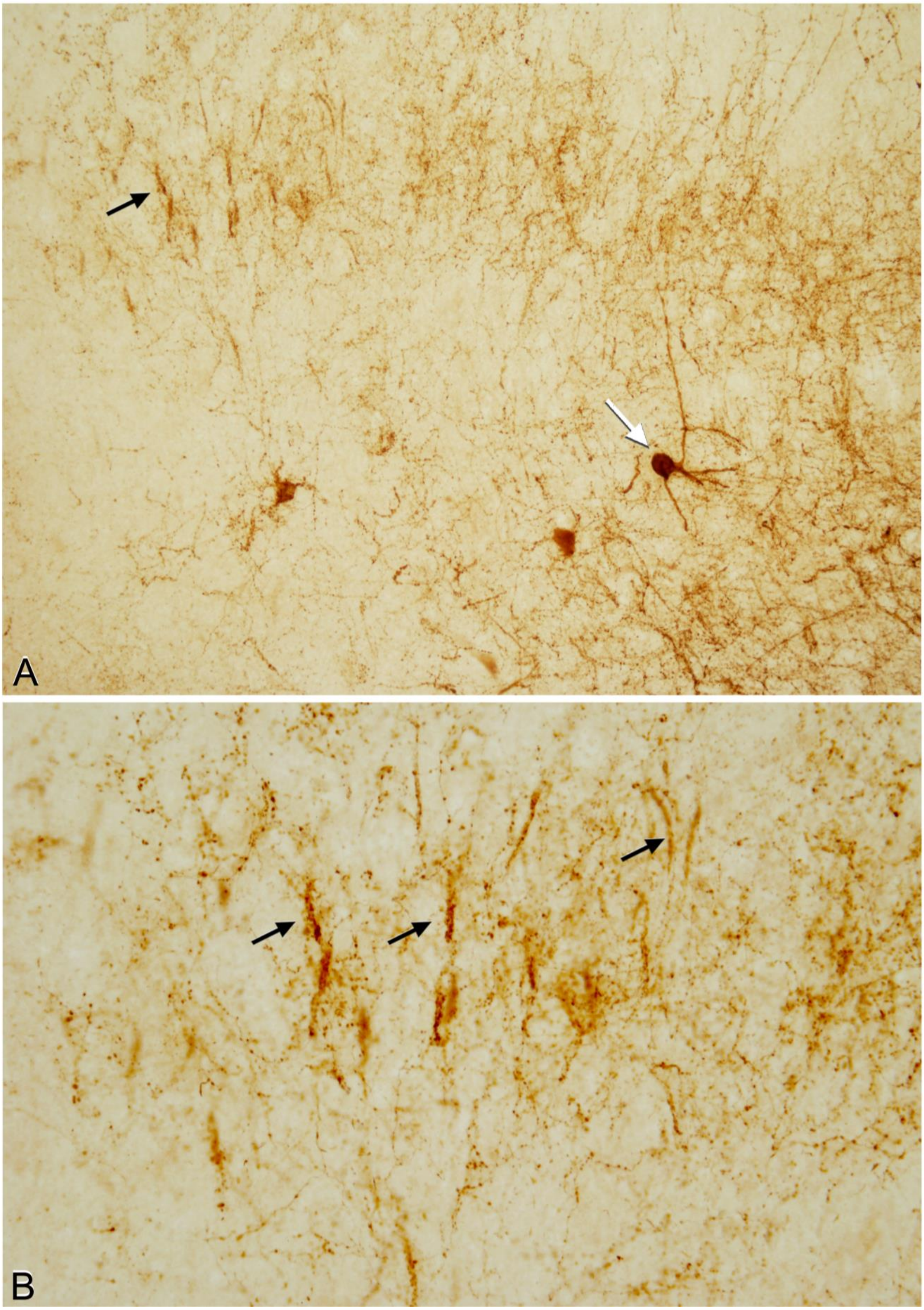

Figure 2-H138-3. **Photomicrographs of a PV-immunostained section**.
(**A**) Higher magnification of Figure 2-H138-2B. White arrow indicates the same PV-immunostained neuron in both pictures. Black arrows indicate chandelier cell axon terminals. (**B**) Higher magnification of (**A**). Note that PV-immunostained chandelier cell axon terminals (arrows) are predominately labeled in this small hippocampal region. Scale bar shown in (**B**) indicates 230 μm in (**A**) and 90 μm in (**B**).
