# Supplementary material for: Neuroanatomical and psychological considerations in temporal lobe epilepsy
Source: Front Neuroanat. 2022 Dec 14;16:995286. doi: 10.3389/fnana.2022.995286 (PMC9794593; doi:10.3389/fnana.2022.995286)
Supplement: Supplementary file 1 [file Data_Sheet_1.zip › Supplementary material/Supplementary Figures 2/Supplementary Figures 2-H164.pdf]

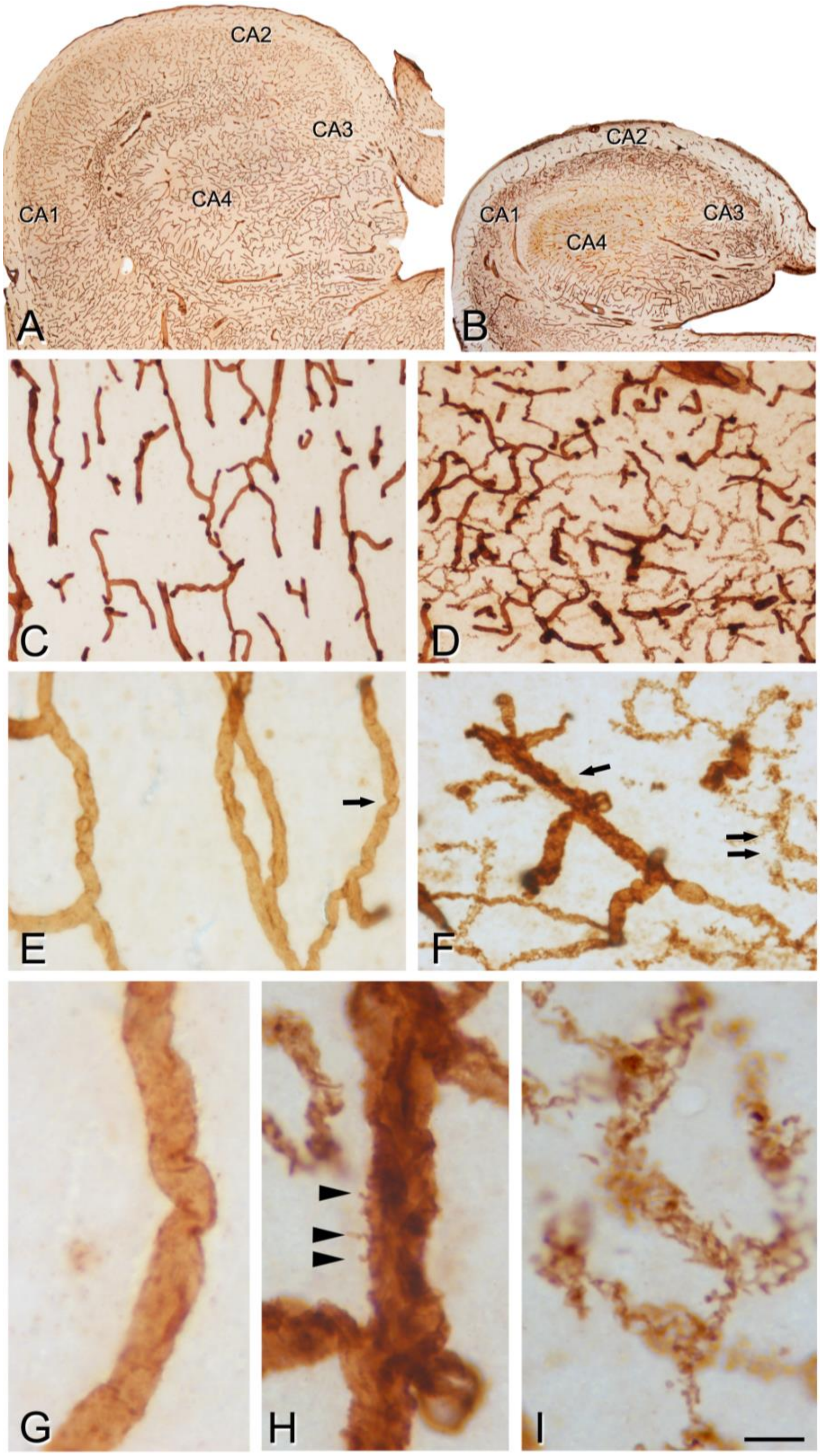

Figure 2-H164. **Photomicrographs of collagen IV-immunostained hippocampal sections**.

(**A**, **B**) Low-power photomicrographs from an autopsy control case (M10: female, 63 years old) (**A**) and from the sclerotic hippocampus of patient H164 (**B**) to show the distribution of collagen IV-immunoreactive (ir) blood vessels. These sections are adjacent to the Nissl-stained sections shown in Figure 1A and 1C, respectively. (**C**, **D**) Photomicrographs of the CA1 from the control (**C**) and sclerotic (**D**) hippocampus illustrating the increase in collagen IV-ir blood vessels in the sclerotic tissue. (**E**, **F**) Higher magnification of the CA1 field from the control (**E**) and sclerotic (**F**) hippocampus illustrating morphological differences in immunostained blood vessels. (**G**) Higher magnification of the blood vessel indicated by an arrow in (**E**) illustrates the smooth surface of the collagen IV-ir blood vessels in the control tissue. (**H**) Higher magnification of the blood vessel marked with a single arrow in (**E**) shows the presence of small spinelike protrusions on the surface of the collagen IV-ir blood vessel (arrowheads). (**I**) Higher magnification of the area indicated with a double arrow in (**F**) to show the abnormal tubular or vascular-like collagen IV-ir structures with a vacuolar or reticulated appearance. Scale bar in (**I**): 920 µm in (**A**, **B**); 75 µm in (**C**, **D**); 30 µm in (**E**, **F**); 10 µm in (**G**-**I**). Adapted from Kastanauskaite et al. (2009).
