# Supplementary material for: Neuroanatomical and psychological considerations in temporal lobe epilepsy
Source: Front Neuroanat. 2022 Dec 14;16:995286. doi: 10.3389/fnana.2022.995286 (PMC9794593; doi:10.3389/fnana.2022.995286)
Supplement: Supplementary file 1 [file Data_Sheet_1.zip › Supplementary material/Supplementary Figures 2/Supplementary Figures 2-H48.pdf]

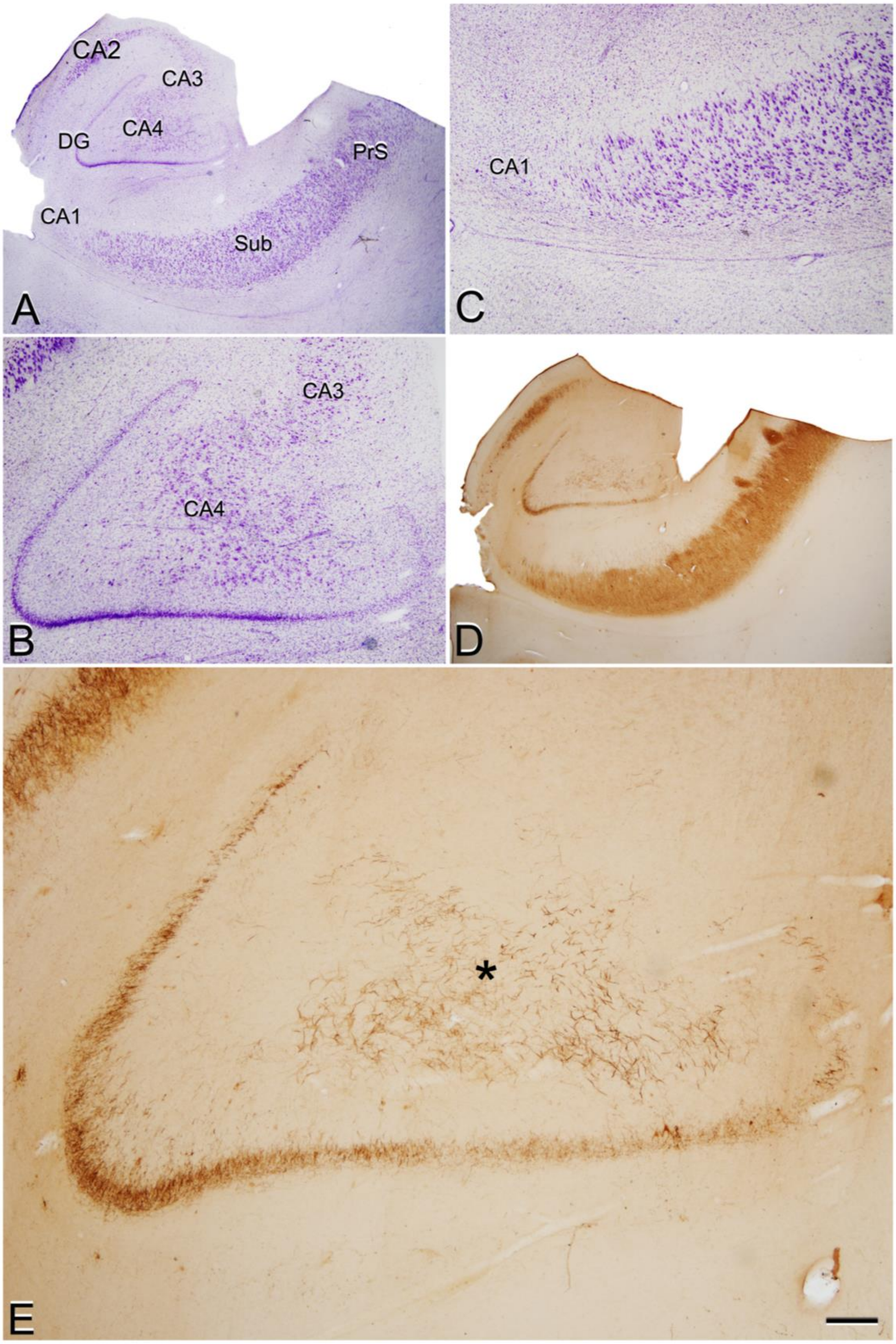

Figure 2-H48-1. **Nissl-stained and PV-immunostained sections**.

(A-C) Photomicrographs of Nissl-stained sections showing the hippocampal formation at low (A) and higher magnification (**B**, **C**). Note the neuronal loss in the DG, CA4, CA3 and CA1 fields. (**D**, **E**) Photomicrographs at low (**D**) and higher (**E**) magnification from a section adjacent to (**A**) immunostained for PV. There is a general reduction of PV immunostaining in DG, CA4, CA3 and CA1 fields. In CA4, PV immunostaining is restricted to a limited area (asterisk) shown at higher magnification in Figures S2-H48-2 and S2-H48-3. Adapted from Arellano et al. (2004). Scale bar shown in (**E**) indicates 1350 μm in (**A**) and (**D**), 500 μm in (**B**) and (**C**) and 250 μm in (**E**). CA1-CA4: Cornu ammonis fields 1-4; DG: dentate gyrus; Sub: subiculum. PrS: presubiculum.

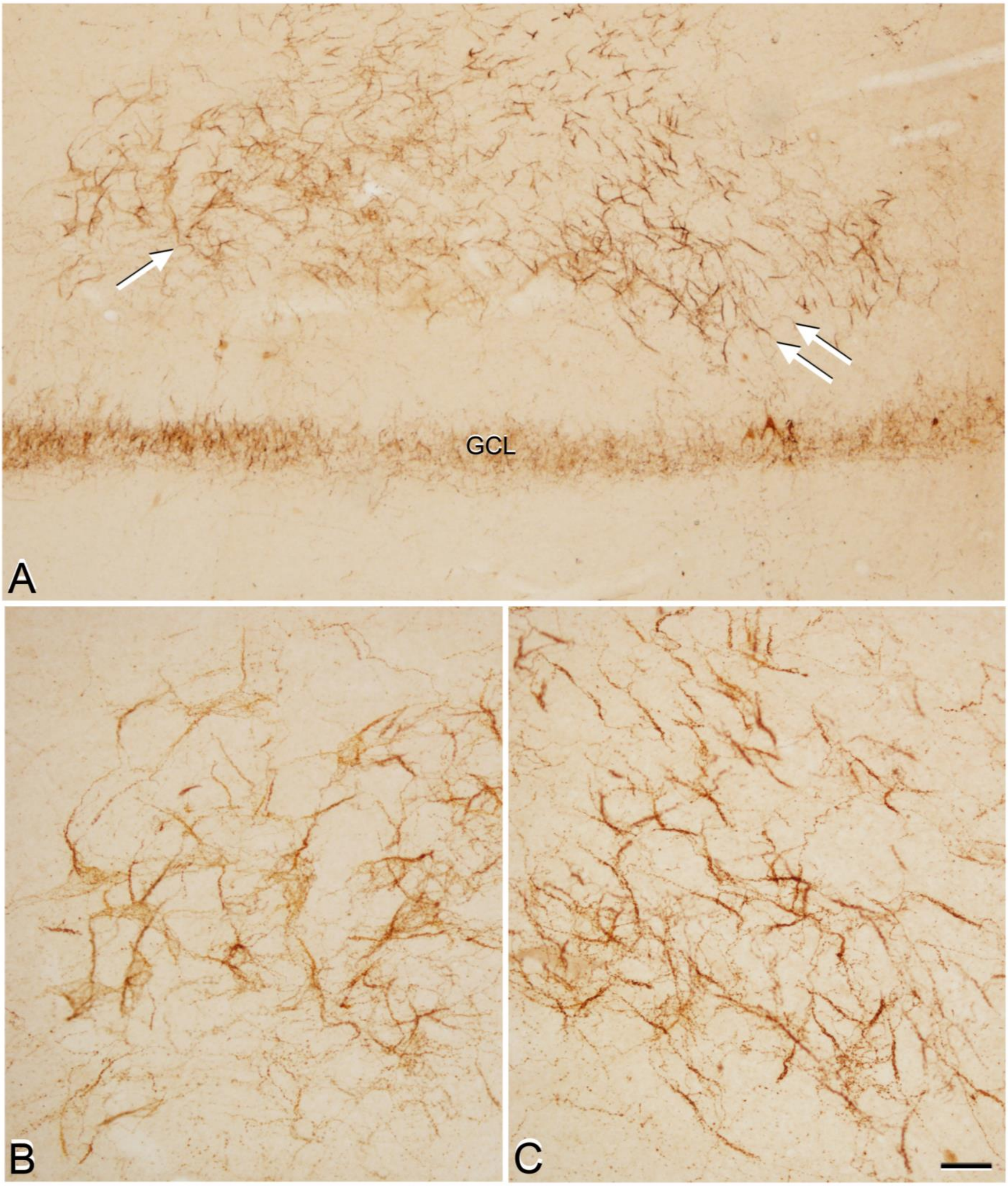

Figure 2-H48-2. **PV-immunoreactive section shows profound alterations in GABAergic perisomatic inhibition**.

(A) Photomicrograph showing PV immunostaining in the granule cell layer (GCL) of the dentate gyrus and CA4 (see Fig. S3-H48-1E). Some surviving neurons are innervated by PV immunostained chandelier-terminals and basket formations (left, arrow) or only by PV immunostained chandelier-terminals (right, double arrow). (**B**, **C**) Higher magnification of (**A**) showing the left and right parts of (**A**), respectively. Adapted from Arellano et al. (2004). Scale bar shown in (**C**) indicates 130 μm in (**A**) and 60 μm in (**B**) and (**C**).

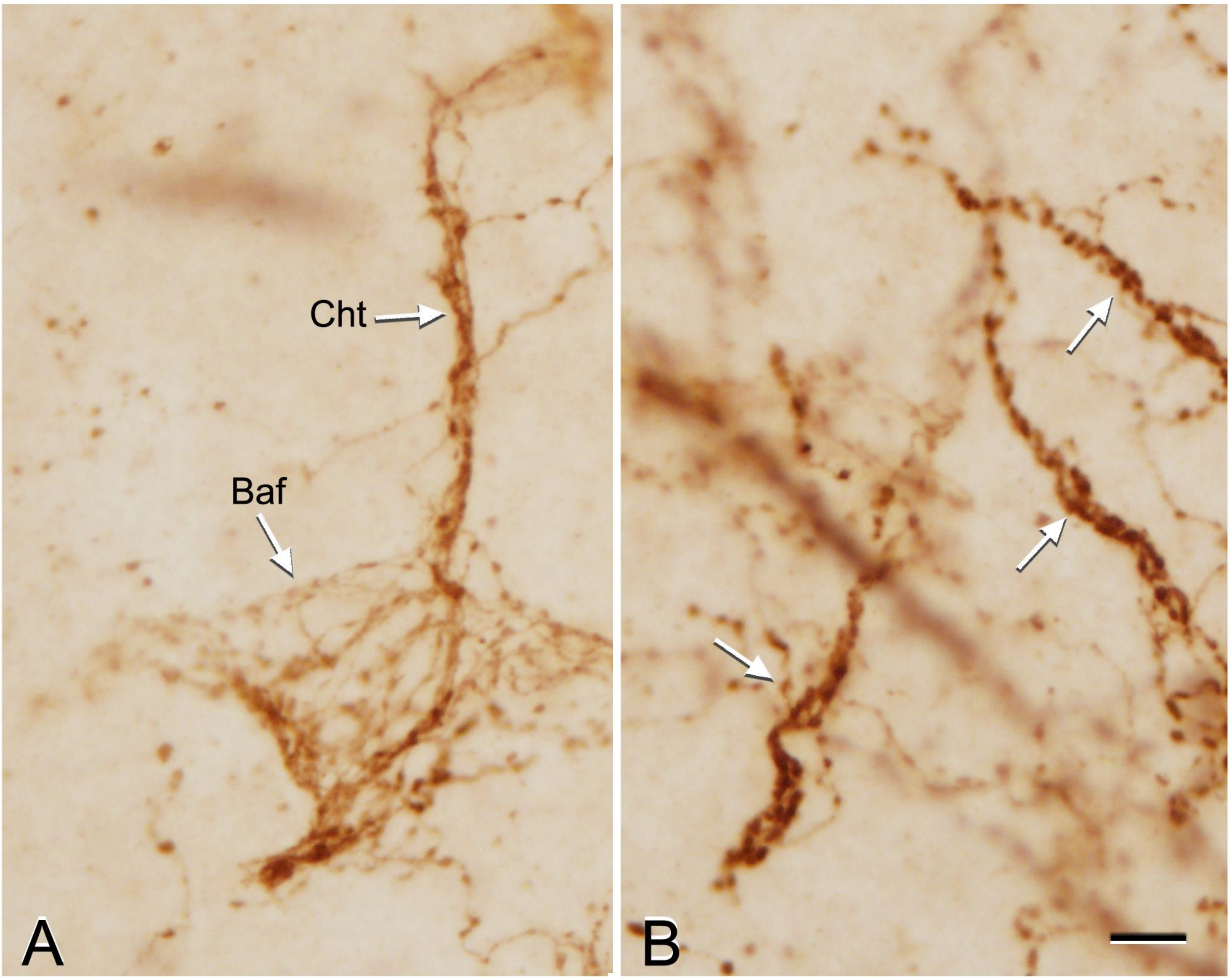

Figure 2-H48-3. **Photomicrographs of a PV-immunoreactive section**.
(**A**) Higher magnification of Figure 3-H48-2B showing a PV immunostaining basket formation (Baf) and a chandelier-terminal (Cht) innervating the soma and the axon initial segment of an unlabeled neuron. (**B**) Higher magnification of Figure 3-H48-2C showing PV immunostaining Cht (arrows). Adapted from Arellano et al. (2004). Scale bar shown in (**B**) indicates 10 μm in (**A**) and (**B**).

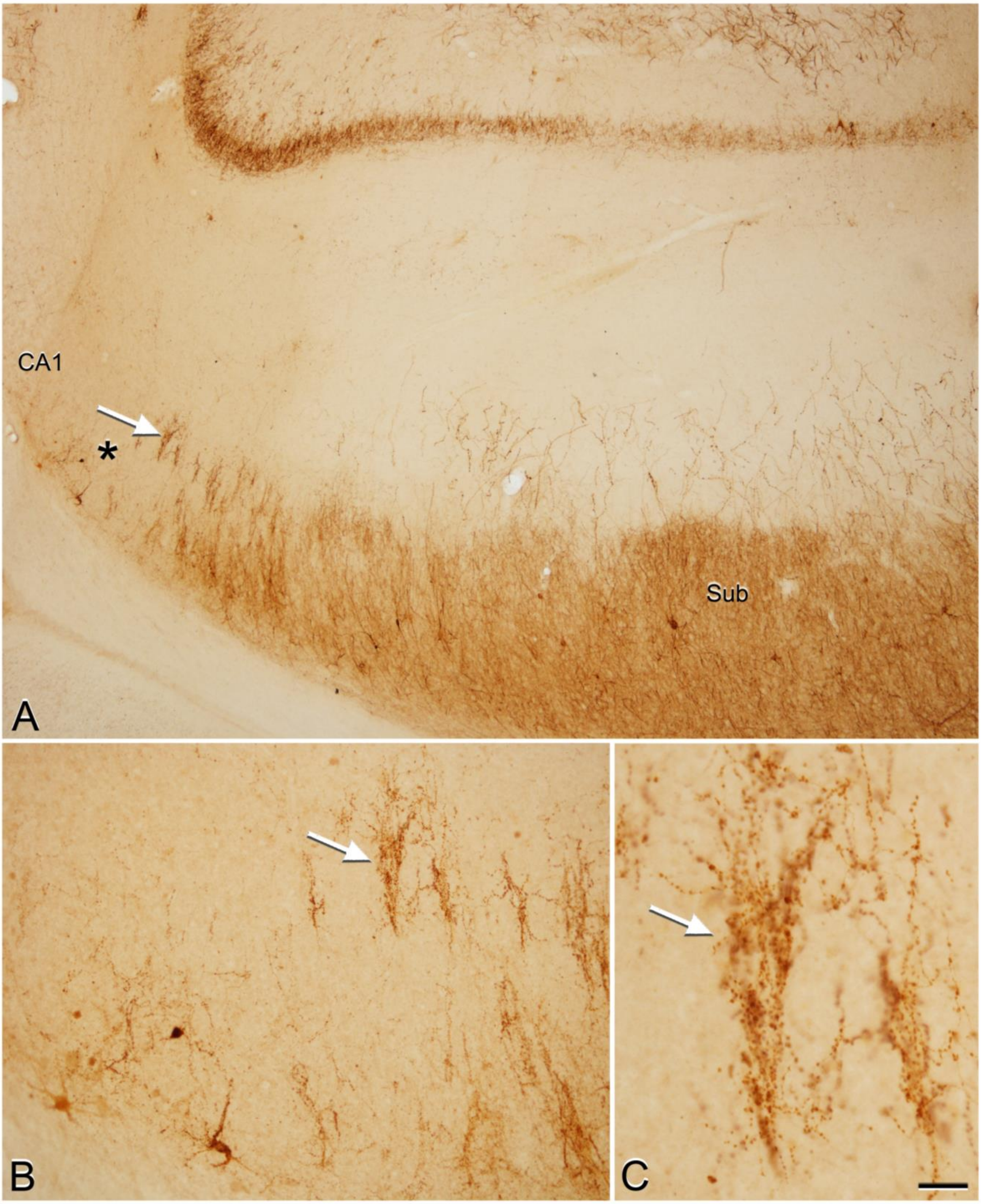

Figure 2-H48-4. **Photomicrographs of a PV-immunoreactive section**.

(A) Higher magnification of Figure 2-H48-1D showing the decrease in the density of PV immunostaining in the neuropil of CA1. Note the presence of abnormally dense basket formations, intensely immunostained for PV around some surviving neurons at the border between the CA1 and the subiculum (asterisk). (**B**, **C)** Higher magnification of (**A**) to illustrate with greater detail the basket formations. Arrows indicate the same basket formation in (**A**-**C**). Adapted from Arellano et al. (2004). Scale bar shown in C indicates 220 µm in (**A**), 70 µm in (**B**) and 25 µm in (**C**).

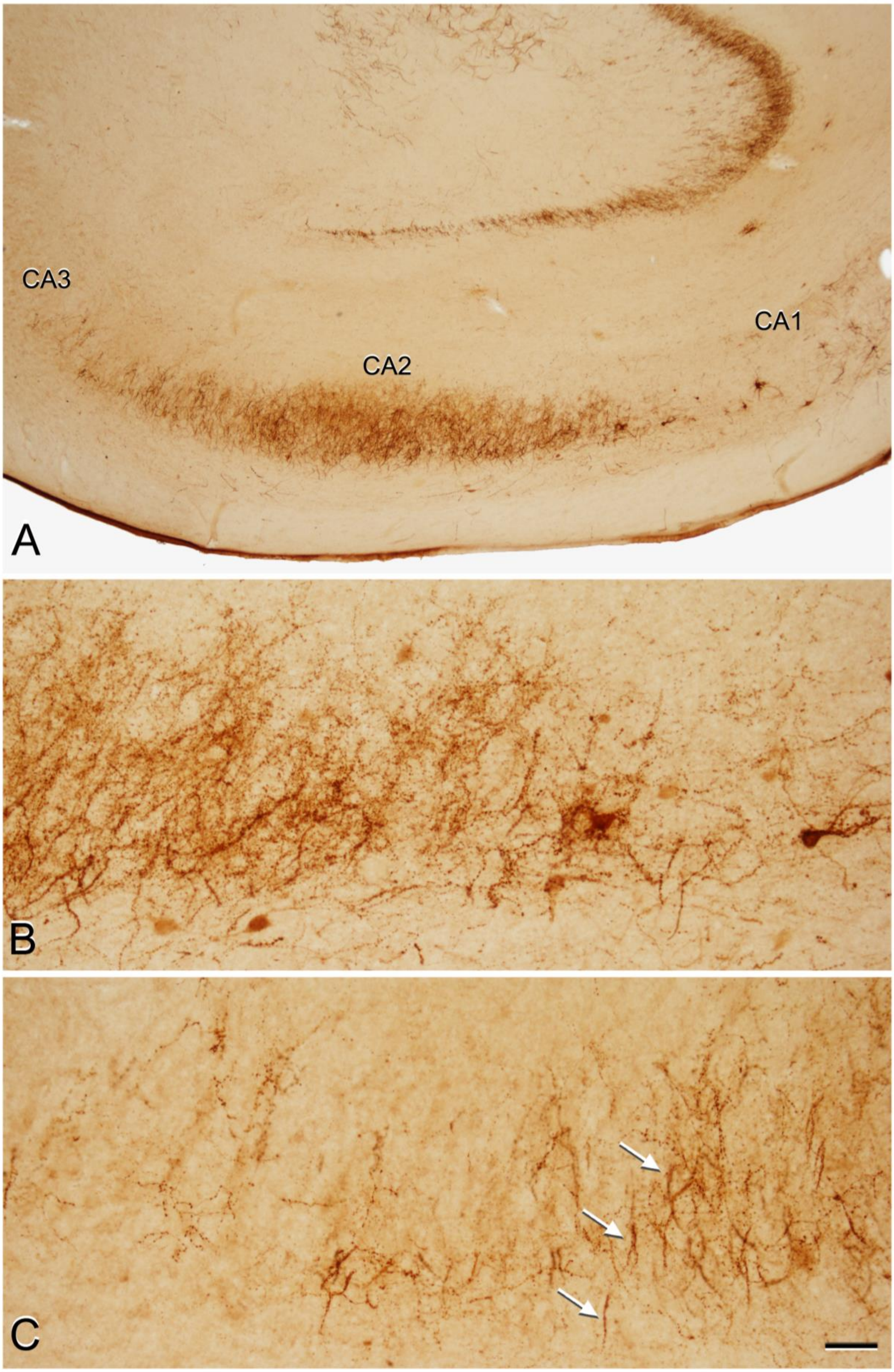

Figure 2-H48-5. **Photomicrographs of a PV-immunoreactive section**.
(**A**) Higher magnification of Figure 3-H48-1D showing PV immunostaining in the neuropil of CA2 and decrease of staining in CA1 and CA3. (**B**, **C**) Higher magnification of (**A**) to illustrate the differences in the pattern of PV immunostaining in the transitional CA2/CA1 (**B**) and CA3/CA2 (**C**) regions. For example, chandelier terminals (arrows in **C**) are predominately labeled in the CA3/CA2 region. Adapted from Arellano et al. (2004). Scale bar shown in (**C**) indicates 260 µm in (**A**), 50 µm in (**B**) and (**C**).
