# Supplementary material for: Neuroanatomical and psychological considerations in temporal lobe epilepsy
Source: Front Neuroanat. 2022 Dec 14;16:995286. doi: 10.3389/fnana.2022.995286 (PMC9794593; doi:10.3389/fnana.2022.995286)
Supplement: Supplementary file 1 [file Data_Sheet_1.zip › Supplementary material/Supplementary Figures 2/Supplementary Figures 2-H61.pdf]

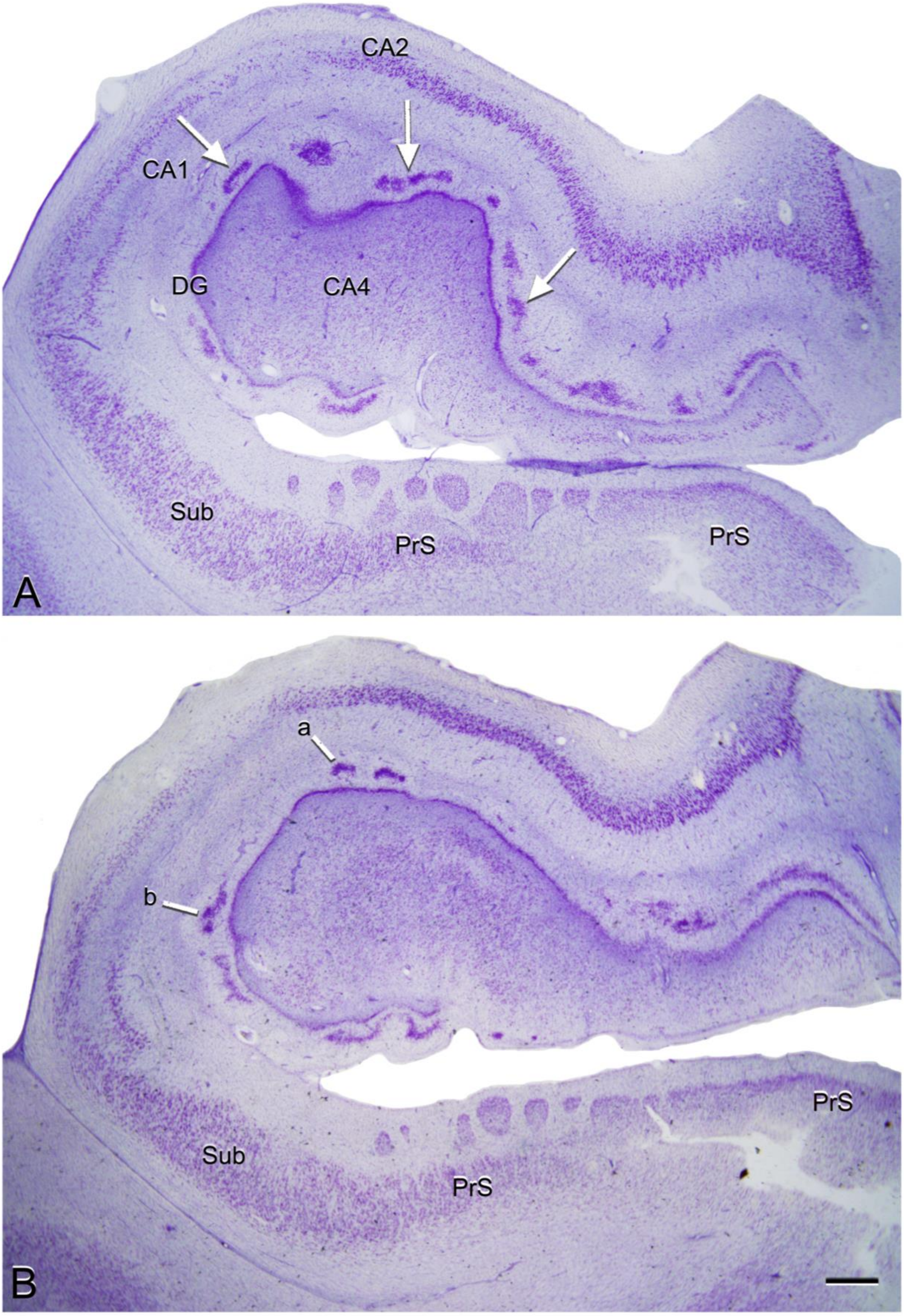

Figure 2-H61-1. **Photomicrographs of Nissl-stained sections**.
(**A**, **B**) Photomicrographs of two serial Nissl-stained sections showing the hippocampal formation at different levels. Note the neuronal loss in the DG, CA4 and CA1 fields. There are multiple clusters of cells (arrows in **A**) with the morphology of granule cells in ectopic locations in the molecular layer of the DG. In (**B**), two clusters of cells (a and b) are also shown in Figure 2-H61-2 at a higher magnification. Scale bar shown in (**B**) indicates 800 μm in (**A**) and (**B**). CA1-CA4: Cornu ammonis fields; DG: dentate gyrus; Sub: subiculum. PrS: presubiculum; PaS: parasubiculum.

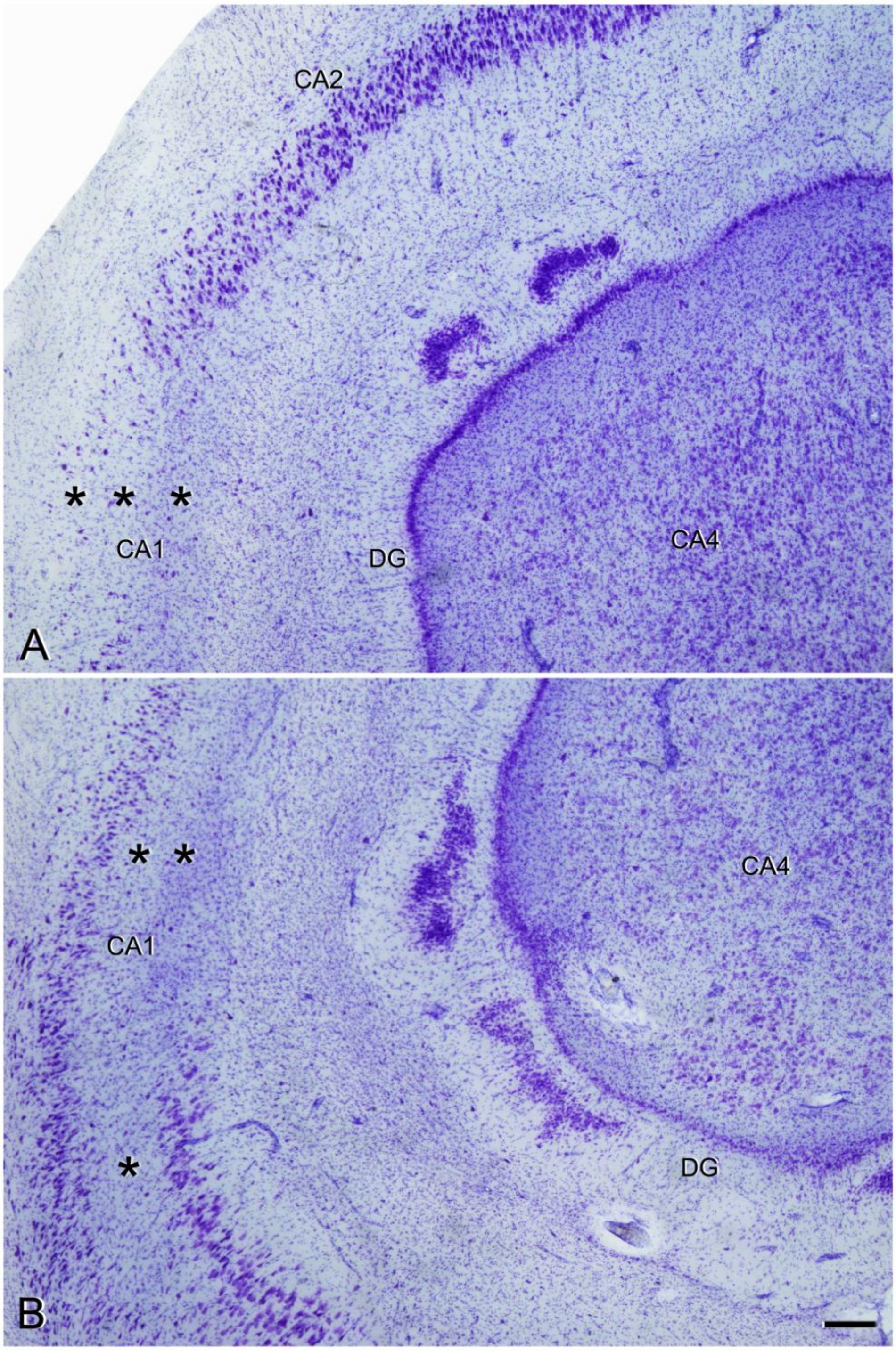

Figure 2-H61-2. **Photomicrographs of a Nissl-stained section**.

(**A**, **B**) Higher-magnification of Figure 2-H61-1B to illustrate the clusters of cells a and b, respectively, and the neuronal loss in the DG, CA4 and CA1 fields. Neuronal loss in CA1 shows regional differences. At the border with CA2, neuronal loss is observed in superficial, middle and deep CA1 layers (tree asterisk in **A**), whereas in the intermediate CA1 region, neuronal loss is observed in the superficial and middle CA1 layers (two asterisks in **B**). At the border with the subiculum, neuronal loss is observed in the middle CA1 layer (one asterisk in **B**). Scale bar shown in (**B**) indicates 250 µm in (**A**) and (**B**). CA1-CA4: Cornu ammonis fields; DG: dentate gyrus.

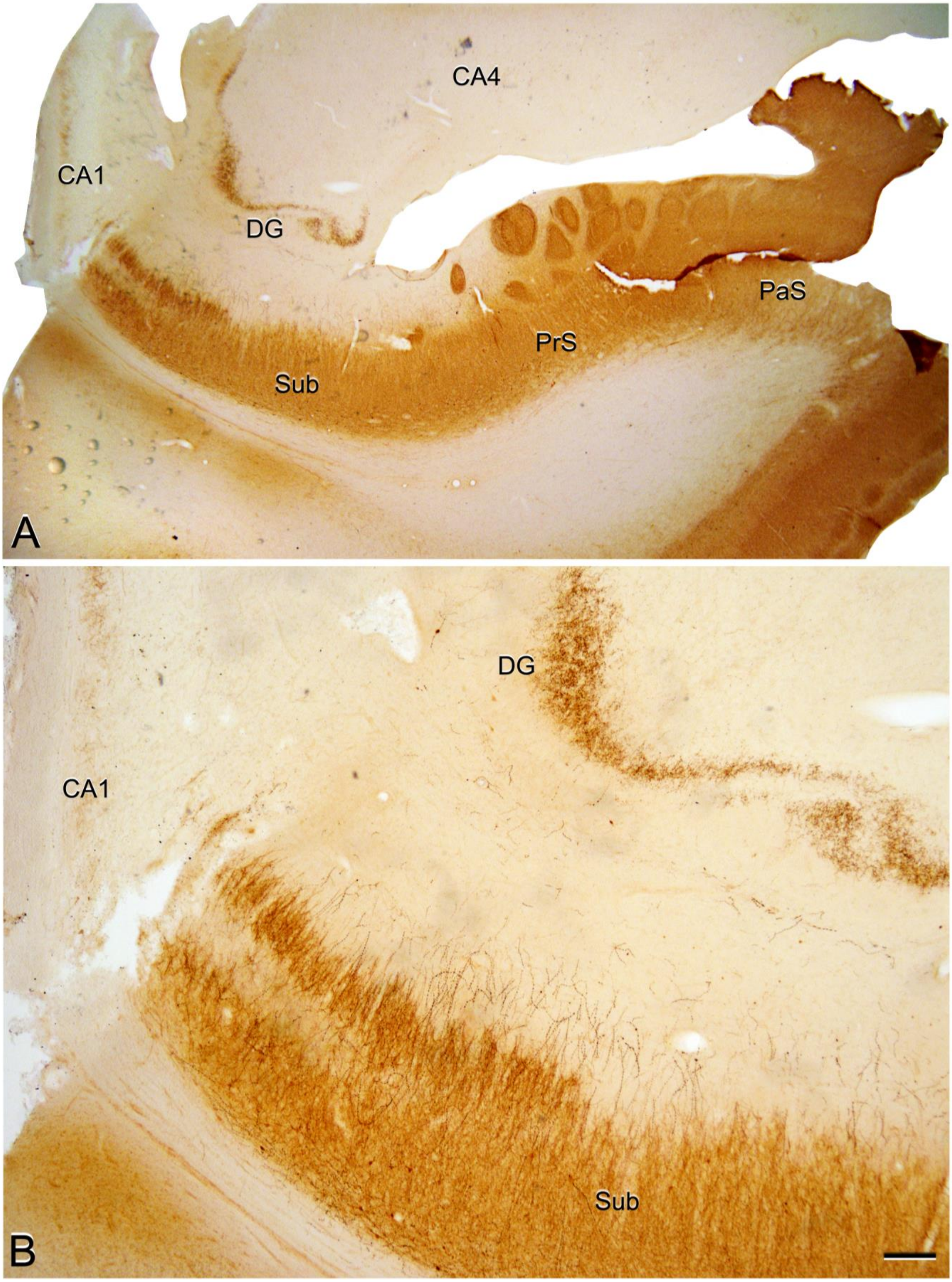

Figure 2-H61-3. Photomicrographs of a PV immunostained section.
(**A**, **B**) Photomicrographs at low (**A**) and high (**B**) magnification from a section adjacent to that shown in Figure H61-S2-1B immunostained for PV. Note the general reduction of PV immunostaining in DG, CA4 and CA1 fields. Scale bar shown in (**B**) indicates 710 µm in (**A**) and 240 µm in (**B**). CA1-CA4: Cornu ammonis fields; DG: dentate gyrus; PrS: presubiculum; PaS: parasubiculum.

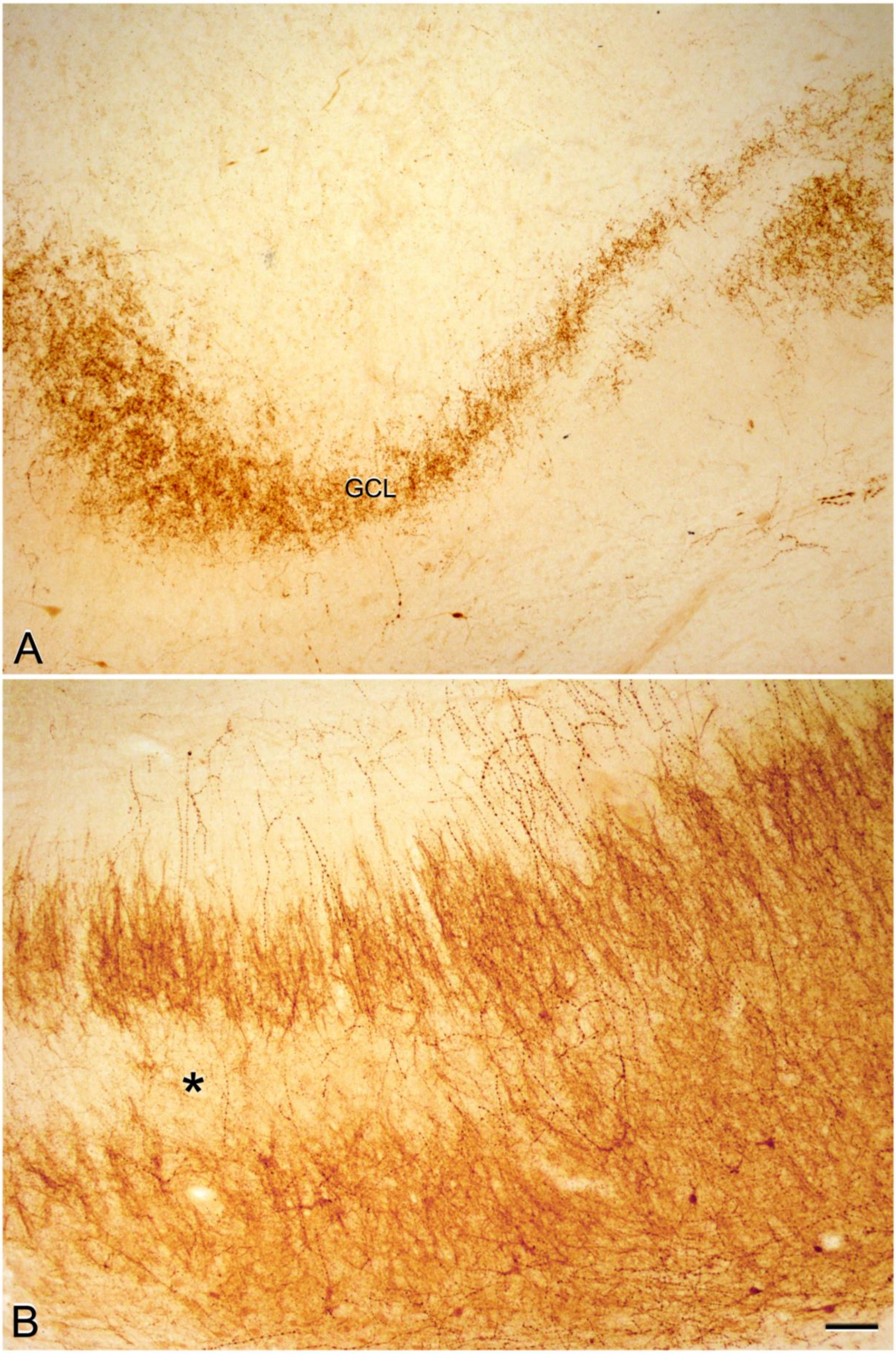

Figure 2-H61-4. Photomicrographs of a PV immunostained section.
(**A**, **B**) Higher-magnification of Figure 2-H61-3 to illustrate the pattern of PV-immunostaining in the granule cell layer (GCL) of the dentate gyrus (**A**) and at the border between CA1 and subiculum (**B**). Note in B the selective laminar decrease of immunostaining (asterisk). Scale bar shown in (**B**) indicates 100 µm in (**A**) and (**B**).
