# Supplementary material for: Neuroanatomical and psychological considerations in temporal lobe epilepsy
Source: Front Neuroanat. 2022 Dec 14;16:995286. doi: 10.3389/fnana.2022.995286 (PMC9794593; doi:10.3389/fnana.2022.995286)
Supplement: Supplementary file 1 [file Data_Sheet_1.zip › Supplementary material/Supplementary Figures 2/Supplementary Figures 2-H67.pdf]

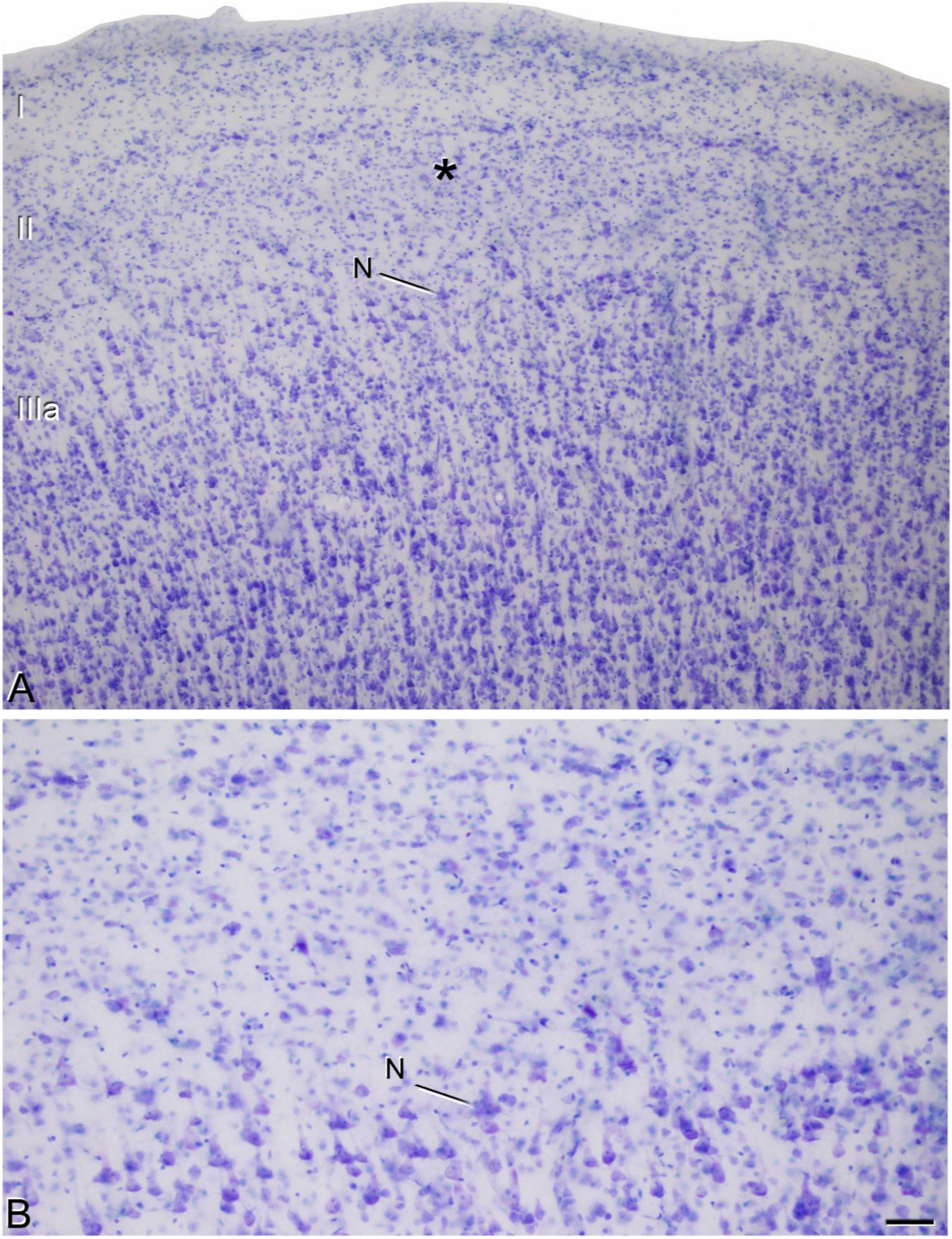

Figure 2-H67-1. **Photomicrographs of a Nissl-stained section**.

(A) Low-power photograph showing neuronal loss (asterisk) mostly in layers II and upper layer IIIa of the lateral temporal cortex (area 21 of Brodmann). (**B**) Higher-magnification of (**A**). N indicates the same neuron in both (**A**) and (**B**). Scale bar shown in (**B**) indicates 90 µm in (**A**) and 40 µm in (**B**).

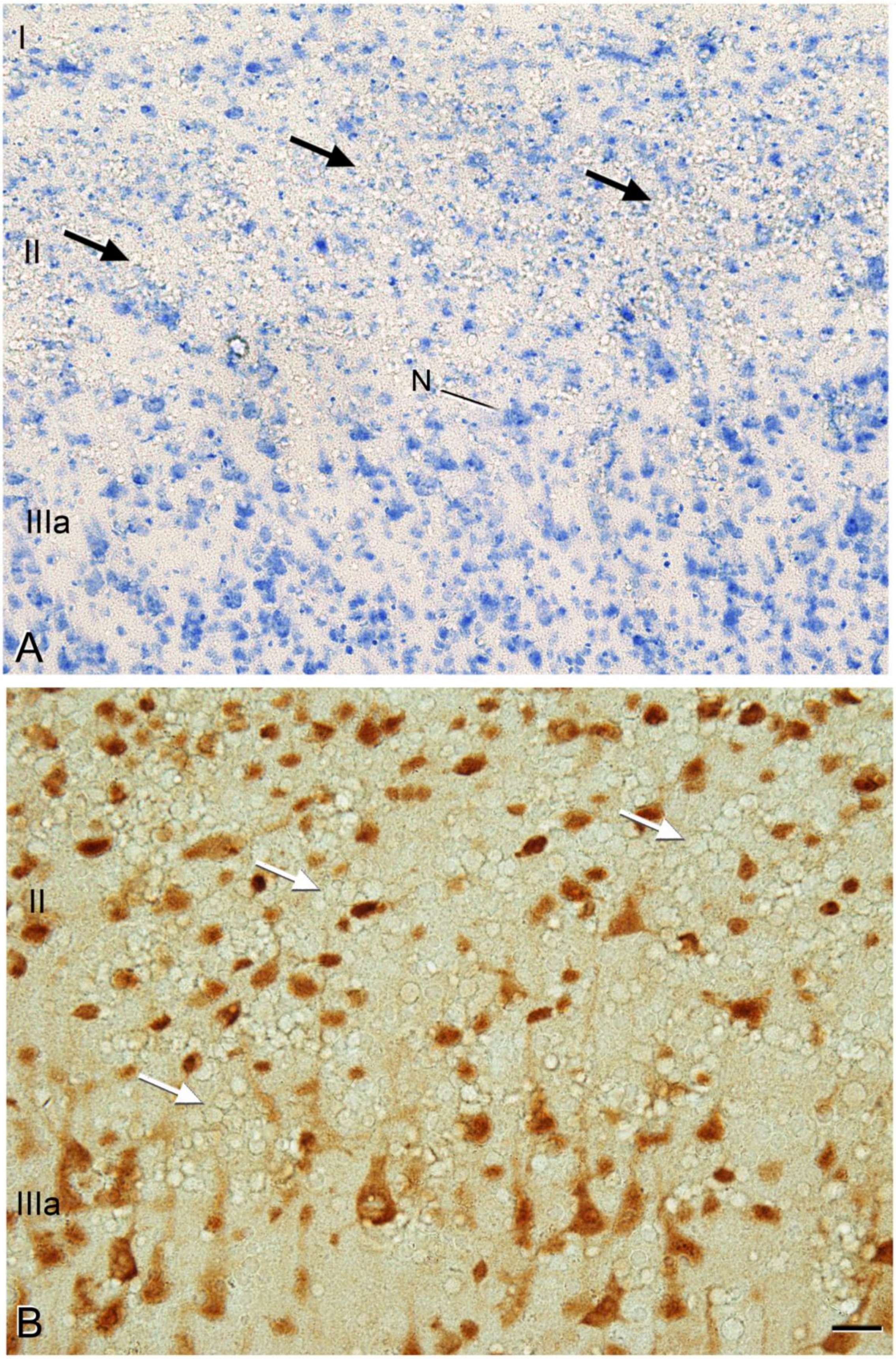

Figure 2-H67-2. **Photomicrographs of Nissl-stained and NeuN-immunostained sections**.
(A) Photomicrograph from the same section and microscopic field as in Figure 2-H67-1 (N indicates the same neuron in both figures) lowering the condenser of the microscope. Black arrows indicate abundant deposits of corpora amylacea (spherical polyglucosan bodies of around 10-15 μm in diameter). (**B**) Adjacent section immunocytochemically stained for NeuN. White arrows indicate some deposits of corpora amylacea. Scale bar shown in (**B**) indicates 40 μm in (**A**) and 25 μm in (**B**).
