# Supplementary material for: Neuroanatomical and psychological considerations in temporal lobe epilepsy
Source: Front Neuroanat. 2022 Dec 14;16:995286. doi: 10.3389/fnana.2022.995286 (PMC9794593; doi:10.3389/fnana.2022.995286)
Supplement: Supplementary file 1 [file Data_Sheet_1.zip › Supplementary material/Supplementary Figures 2/Supplementary Figures 2-H75.pdf]

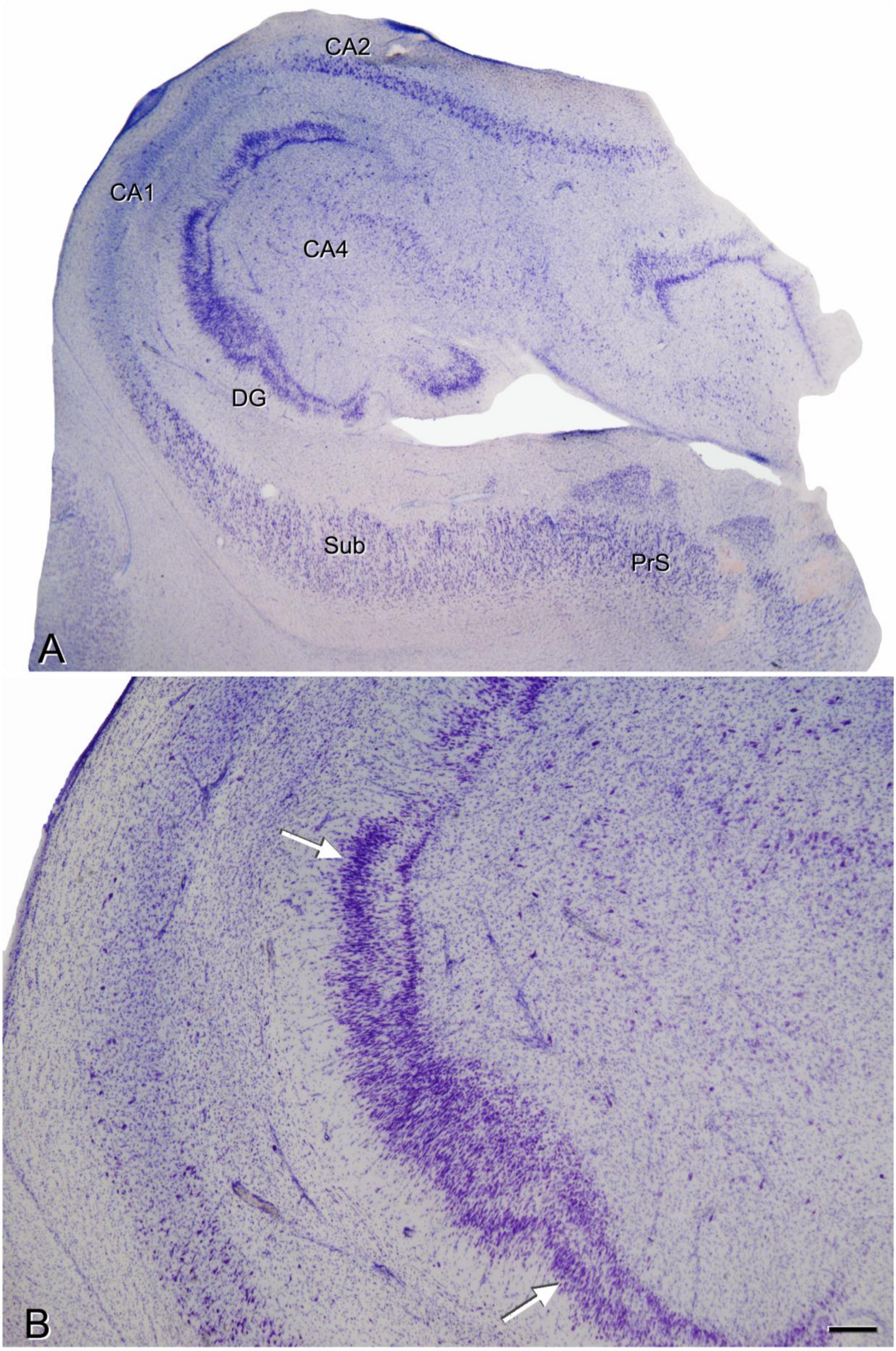

Figure 2-H75-1. **Photomicrographs of a Nissl-stained section**.
(**A**, **B**) Photomicrographs of a Nissl-stained section showing the hippocampal formation. Note the neuronal loss in the CA4, CA3 and CA1 fields. The DG show a bi-laminar pattern of granule cell dispersion (arrows). Scale bar shown in (**B**) indicates 650 μm in (**A**) and 240 μm in (**B**). CA1-CA4: Cornu ammonis fields; DG: dentate gyrus; Sub: subiculum; PrS: presubiculum.
