# Supplementary material for: Neuroanatomical and psychological considerations in temporal lobe epilepsy
Source: Front Neuroanat. 2022 Dec 14;16:995286. doi: 10.3389/fnana.2022.995286 (PMC9794593; doi:10.3389/fnana.2022.995286)
Supplement: Supplementary file 1 [file Data_Sheet_1.zip › Supplementary material/Supplementary Figures 2/Supplementary Figures 2-H80.pdf]

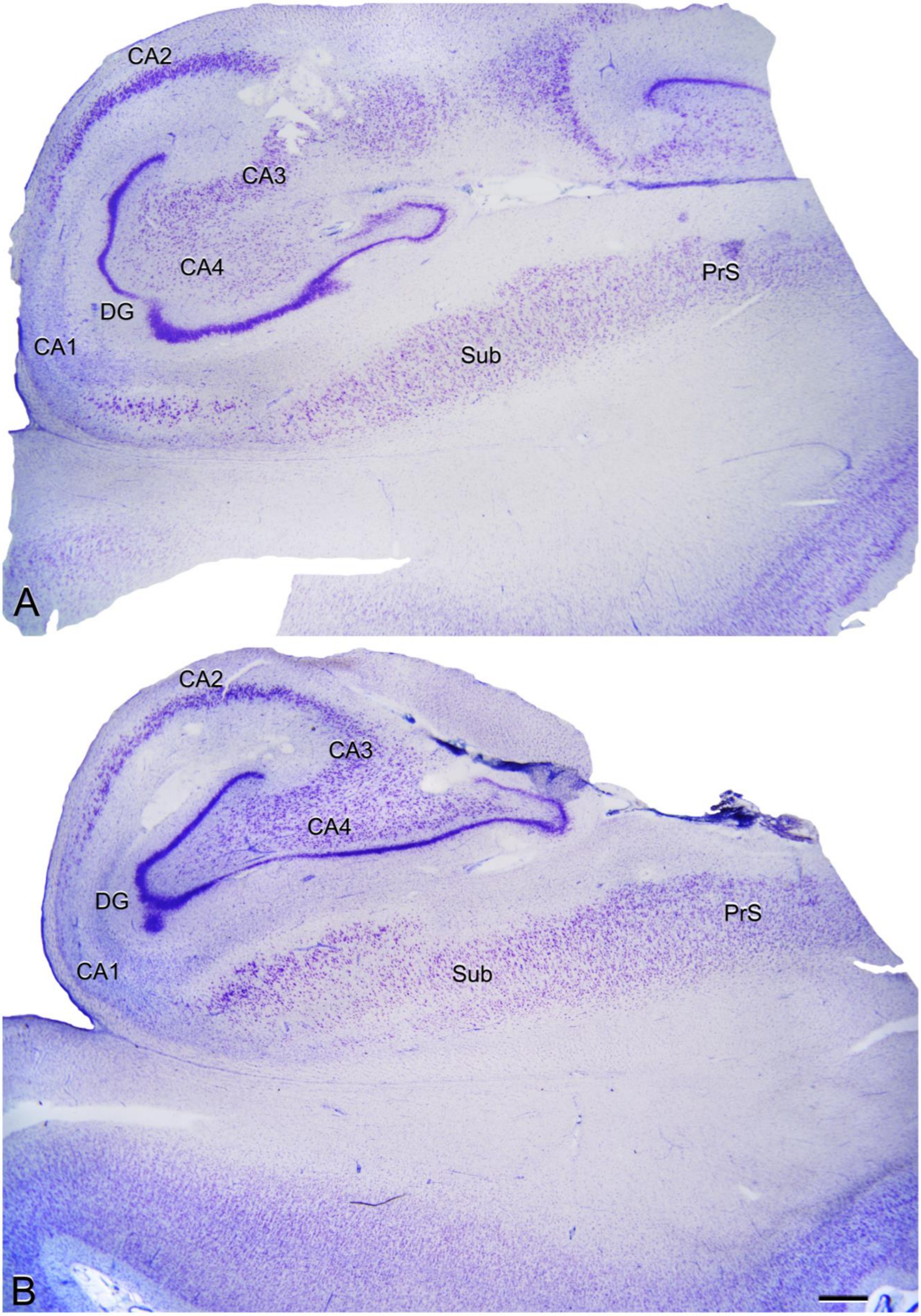

Figure 2-H80-1. **Photomicrographs of Nissl-stained sections**.
(**A**, **B**) Photomicrographs showing the hippocampal formation at a rostral (**A**) and posterior (**B**) levels. Note the extensive loss of neurons in the CA1 field at both levels. Scale bar shown in (**B**) indicates 275 μm in (**A**) and (**B**). CA1-CA2: Cornu ammonis fields; DG: dentate gyrus; Sub: subiculum. PrS: presubiculum.

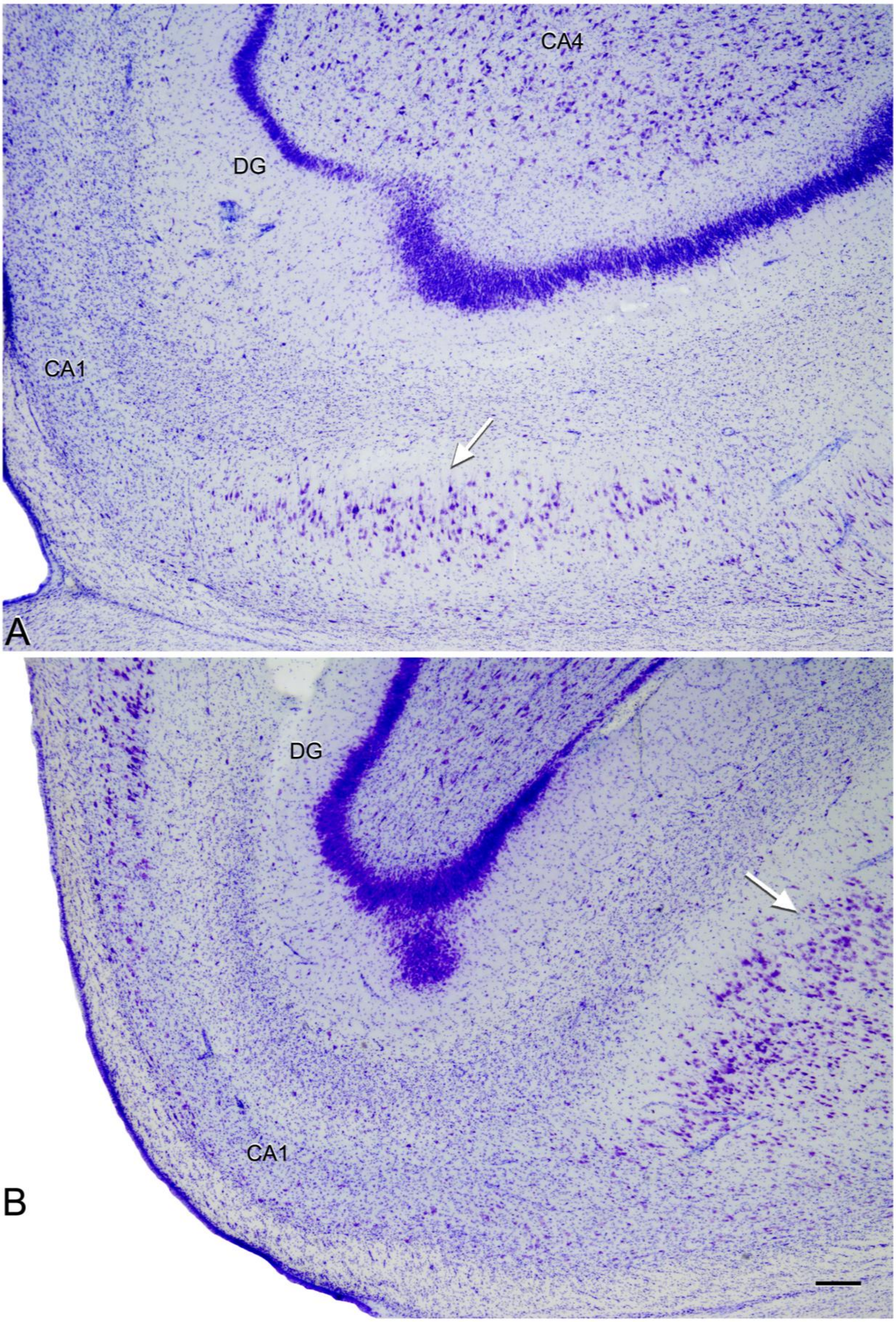

Figure 2-H80-2. **Photomicrographs of Nissl-stained sections**.
(**A**, **B**) Higher magnification Figure 2-H80-1A and 1B, respectively to illustrate with a greater detail CA1 field. Note the numerous surviving cells in the superficial CA1 pyramidal cell layer in a small segment of CA1 (white arrow in **A** and **B**) Scale bar shown in (**B**) indicates 220 µm in (**A**) and in (**B**). CA1-CA4: Cornu ammonis fields; DG: dentate gyrus.

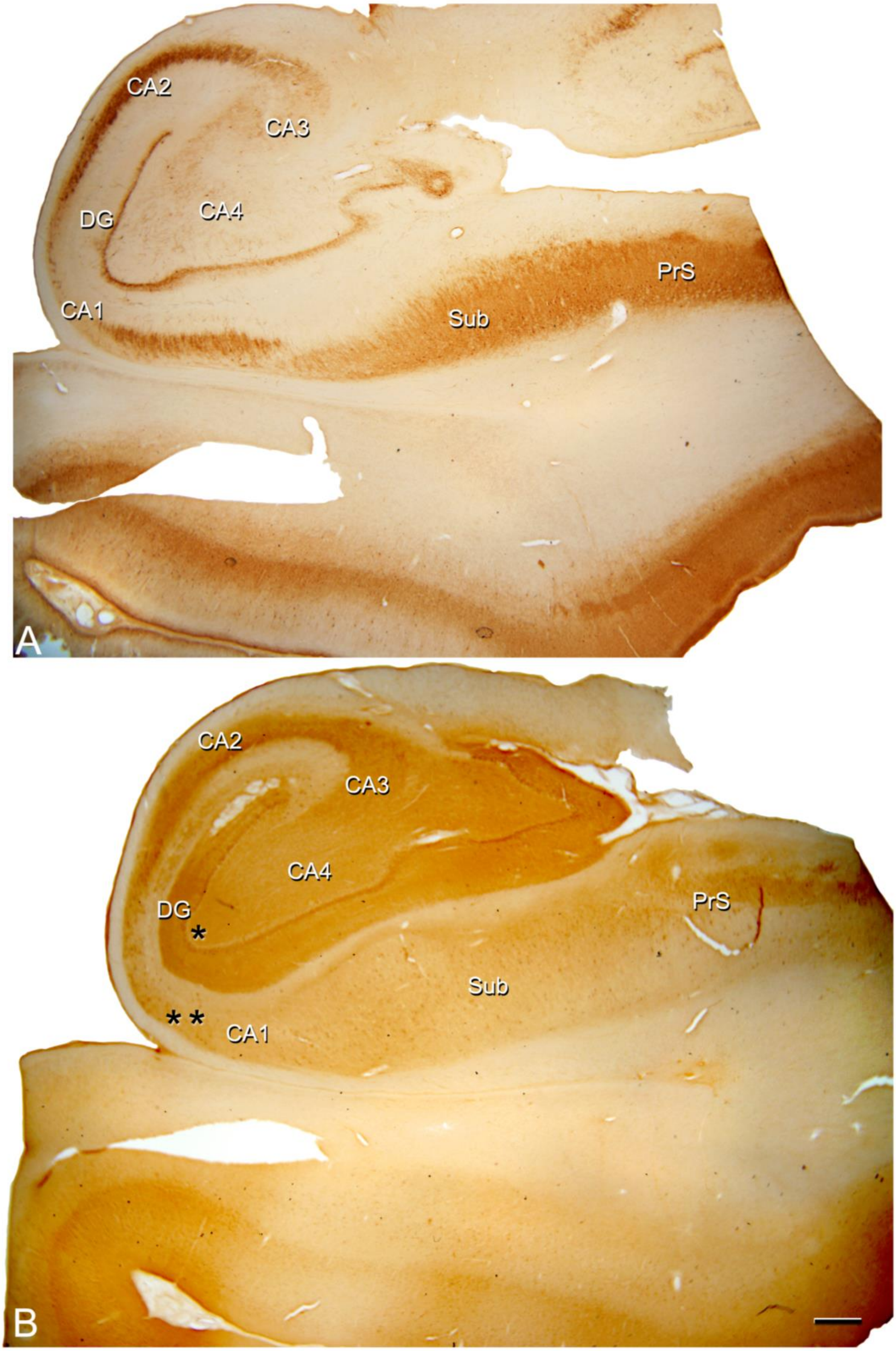

Figure 2-H80-3. **Photomicrographs of PV- and CalB-immunostained sections**.
(**A**, **B**) Photomicrographs of PV- (**A**) and CalB- (**B**) immunostained sections adjacent to the Nissl-stained section shown in Figure S2-H80-1B. Note the general reduction of PV immunostaining in CA4, CA3 and CA1 fields. Asterisks in (**B**) indicate regions that are shown at higher magnification in Figure 3-H80-5. Scale bar shown in (**B**) indicates 700 μm in (**A**) and (**B**). CA1-CA4: Cornu ammonis fields; DG: dentate gyrus; Sub: subiculum. PrS: presubiculum.

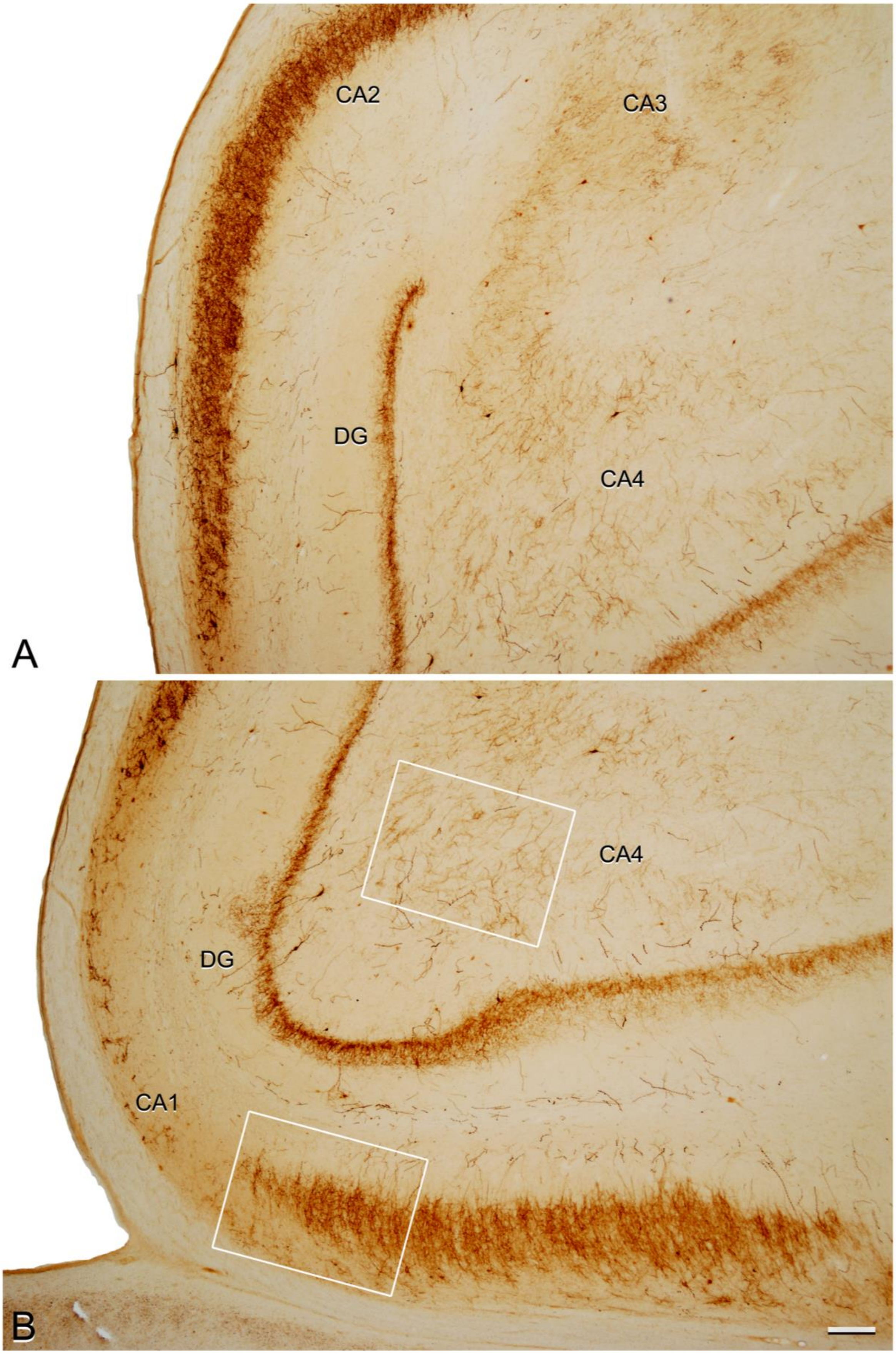

Figure 2-H80-4. **Photomicrographs of a PV-immunostained section**.

(**A**, **B**) Higher magnification of Figure 3-H80-3A to illustrate with a greater detail the pattern of immunostaining of PV in several hippocampal fields. Boxed areas in (**B**) indicate regions that are shown at higher magnification in Figure 3-H80-4. Scale bar shown in (**B**) indicates 230 µm in (**A**) and (**B**). CA1-CA4: Cornu ammonis fields; DG: dentate gyrus.

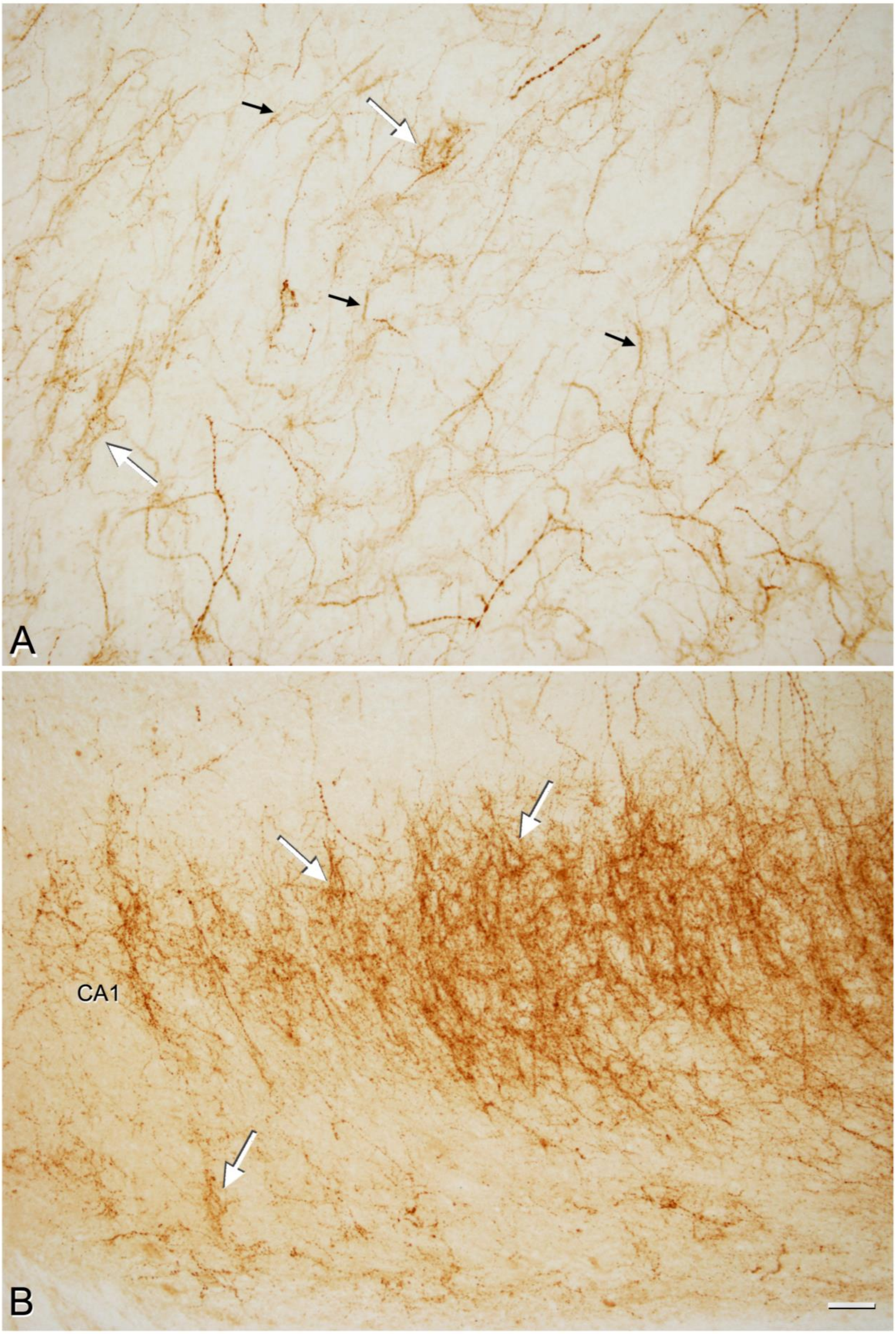

Figure 2-H80-5. **Photomicrographs of a PV-immunostained section**.
(**A**, **B**) Higher magnification of the boxed areas in 2-H80-4B. White arrows black arrows indicate some PV immunostaining basket formations and chandelier-terminals, respectively, in CA4 (**A**) and CA1 (**B**). Scale bar shown in (**B**) indicates 45 µm in (**A**) and in (**B**).

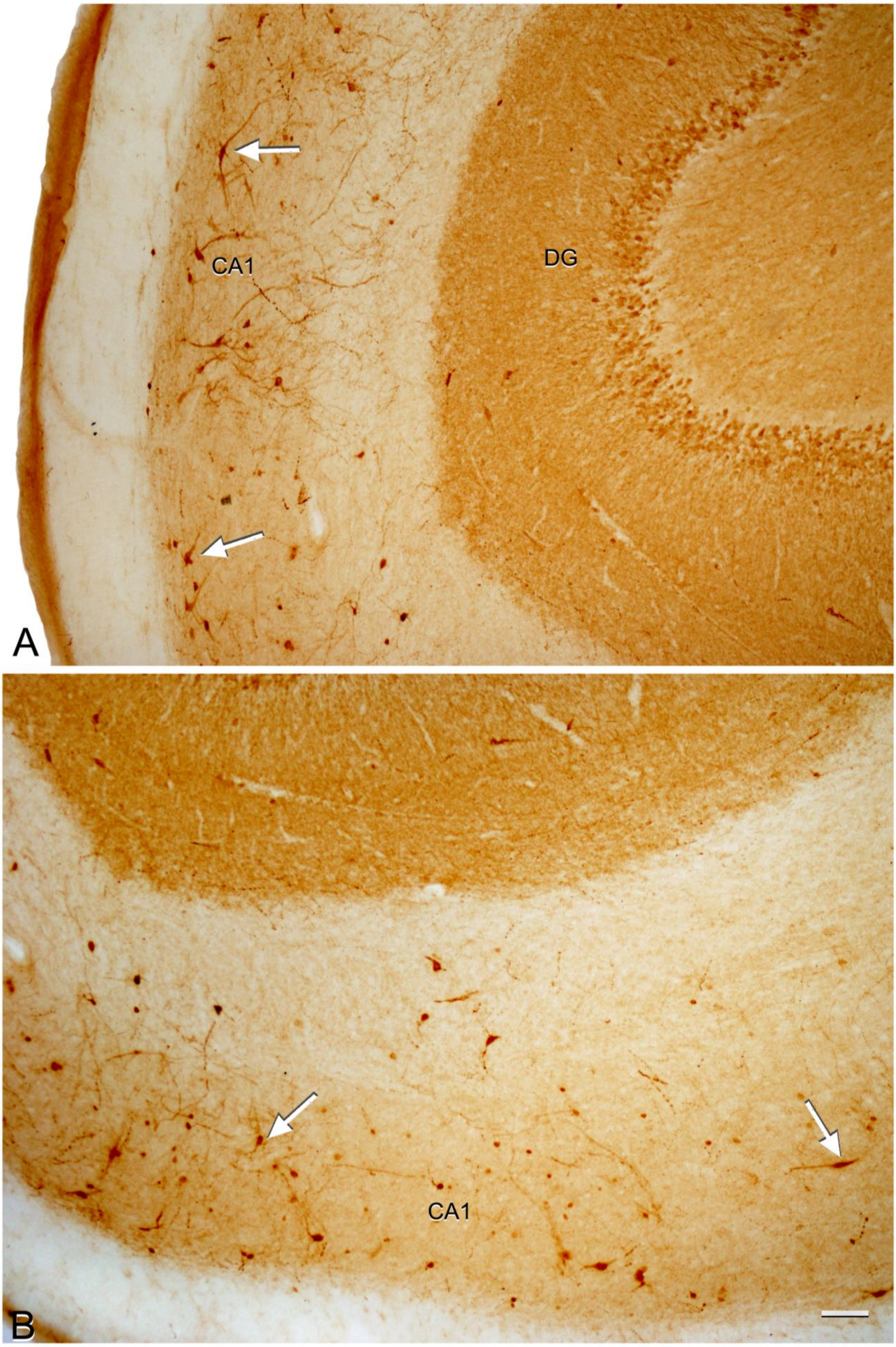

Figure 2-H80-6. **Photomicrographs of a CalB-immunostained section**.
(**A**, **B**) Higher magnification of the regions indicated with one asterisk (**A**) and two asterisks (**B**) in Figure 2-H80-3B, respectively. Numerous CalB-immunostained neurons are seen in the pyramidal cell layer of CA1 field. Scale bar shown in (**B**) indicates 90 μm in (**A**) and in (**B**). DG: dentate gyrus.
