# Supplementary material for: Neuroanatomical and psychological considerations in temporal lobe epilepsy
Source: Front Neuroanat. 2022 Dec 14;16:995286. doi: 10.3389/fnana.2022.995286 (PMC9794593; doi:10.3389/fnana.2022.995286)
Supplement: Supplementary file 1 [file Data_Sheet_1.zip › Supplementary material/Supplementary Figures 2/Supplementary Figures 2-H84.pdf]

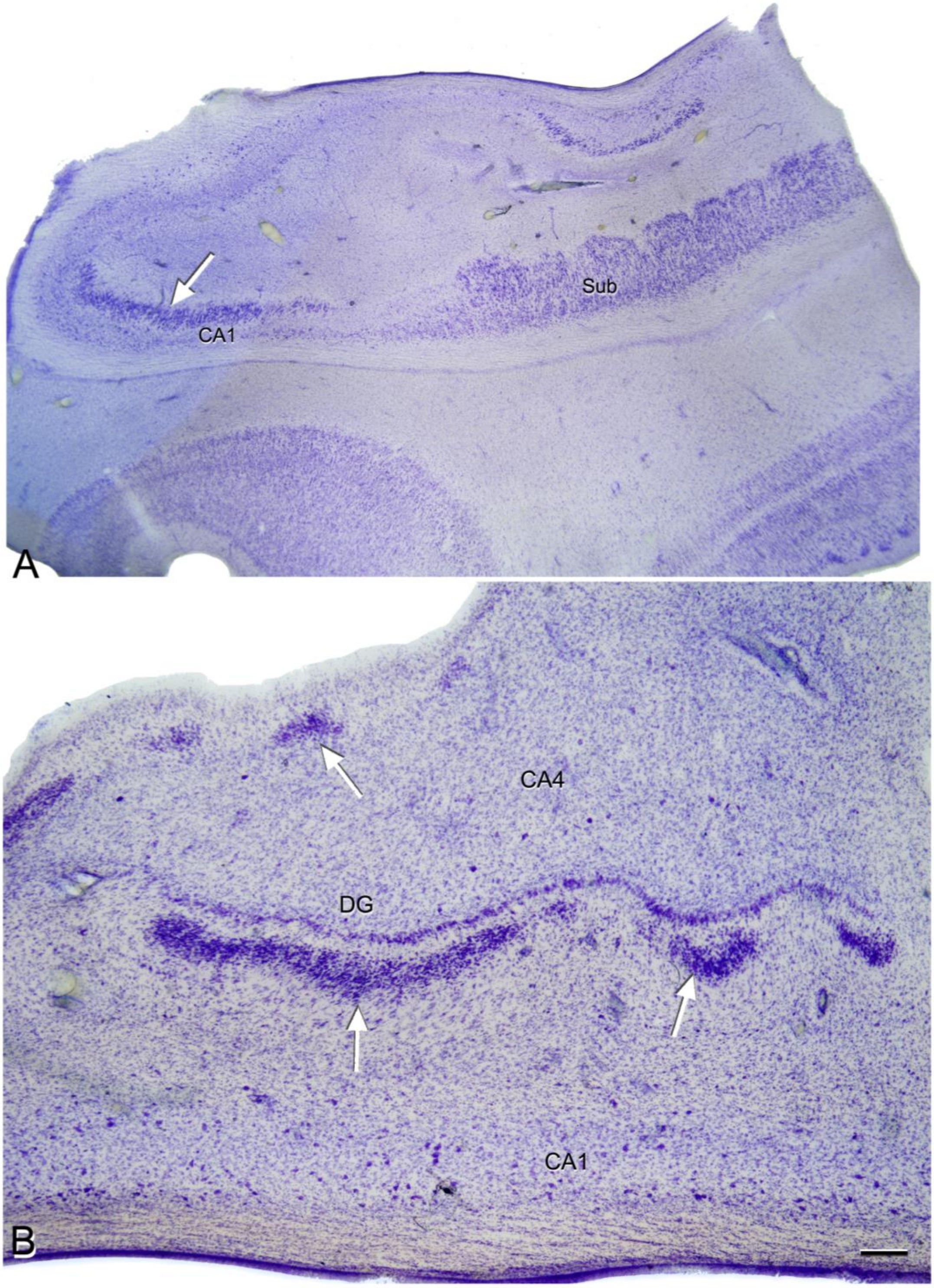

Figure 2-H84-1. **Photomicrographs of Nissl-stained sections**.
(**A**) Photomicrograph showing at a rostral level the hippocampal formation. Note the numerous surviving cells in the superficial CA1 pyramidal cell layer in a small segment of CA1 (white arrow; see also Figure S2-15B) and the extensive loss of neurons in all hippocampal subfields (CA4 and CA3 have practically disappeared). (**B**) Photomicrograph showing the hippocampus at a more posterior level than in (**A**). Note the DG with multiple clusters of cells (arrows) with the morphology of granule cells in ectopic locations in the molecular layer of the DG, and the loss of neurons in CA1 and CA4. At more posterior levels, the hippocampal formation displayed a sclerosis type 1 classic (see Table 3), with less severe neuronal loss. Scale bar shown in (**B**) indicates 730 μm in (**A**) and 200 μm in (**B**). CA1-CA4: Cornu ammonis fields; DG: dentate gyrus; Sub: subiculum.

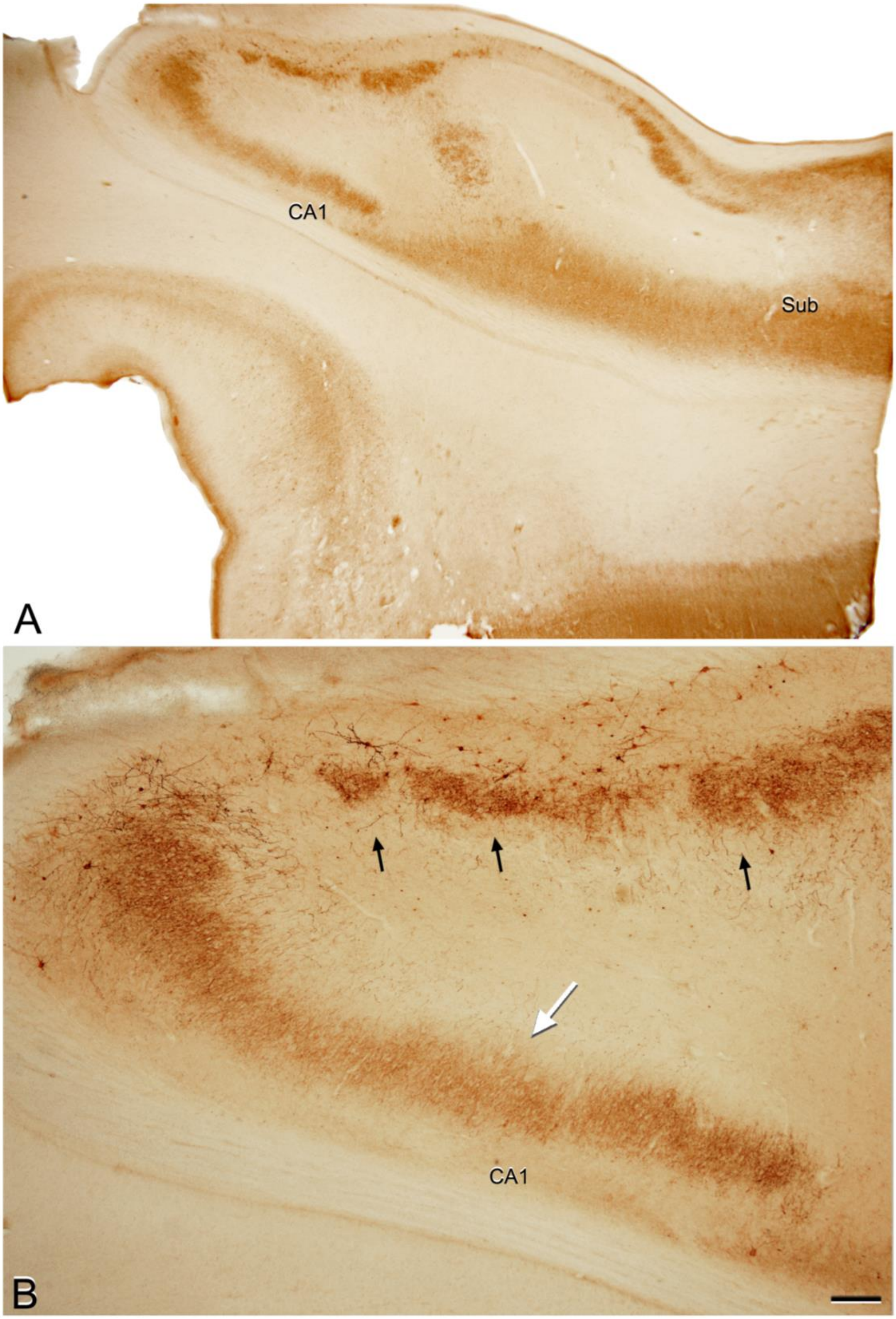

Figure 2-H84-2. Photomicrographs of a PV-immunostained section
(**A**, **B**) Photomicrographs of a PV immunostained section adjacent to the Nissl-stained section showed in Figure S2-H84-1A. Note the intense PV labeling of the neuropil in the superficial layer of CA1 which shows numerous surviving neurons in Nissl stained sections (white arrow) and in the segment of CA1 where only few isolated surviving neurons are present in Nissl stained sections (black arrows). Scale bar shown in (**B**) indicates 730 µm in (**A**) and 240 µm in (**B**). CA1-CA4: Cornu ammonis fields; DG: dentate gyrus; Sub: subiculum.

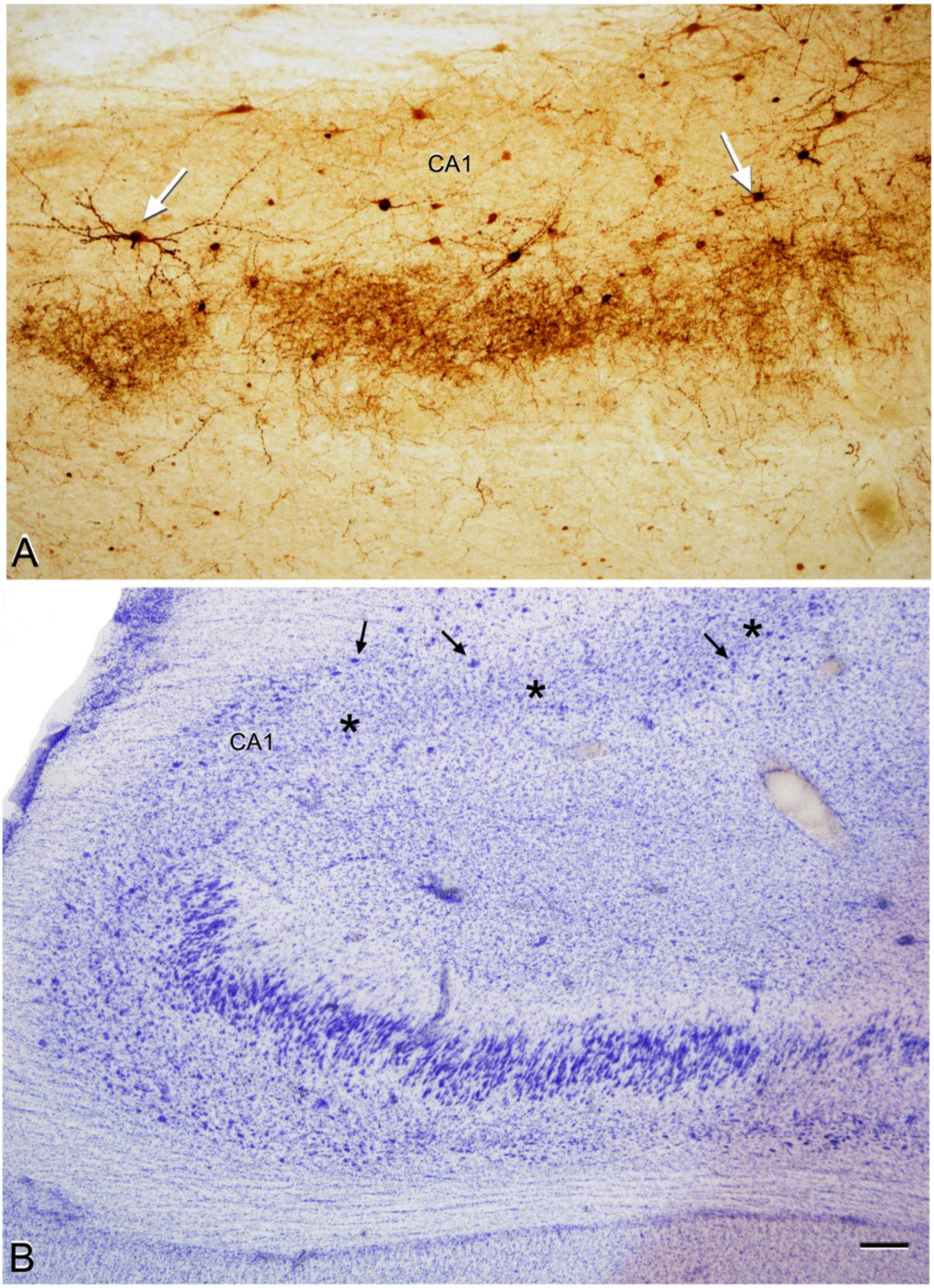

Figure 2-H84-3. Photomicrographs of PV-immunostained and Nissl-stained sections

(**A**) Higher magnification of Figure 3-H84-2B showing the pattern of PV immunostaining corresponding to a small segment of the sclerotic CA1 indicated with asterisks in (**B**). (**B)** Photomicrograph of a Nissl-stained adjacent section to (**A**) which is also shown at a lower magnification in Figure 3-H84-1A. Black arrows indicate some isolated surviving neurons from the pyramidal cell layer of CA1. Note in (**A**) the relatively intense PV-immunostaining in CA1 in spite of the extensive neuronal loss. White arrows indicate some PV-immunostained interneurons. Scale bar shown in (**B**) indicates 90 μm in (**A**) and 230 μm in (**B**).
