# Supplementary material for: Neuroanatomical and psychological considerations in temporal lobe epilepsy
Source: Front Neuroanat. 2022 Dec 14;16:995286. doi: 10.3389/fnana.2022.995286 (PMC9794593; doi:10.3389/fnana.2022.995286)
Supplement: Supplementary file 1 [file Data_Sheet_1.zip › Supplementary material/Supplementary Figures 2/Supplementary Figures 2-H94.pdf]

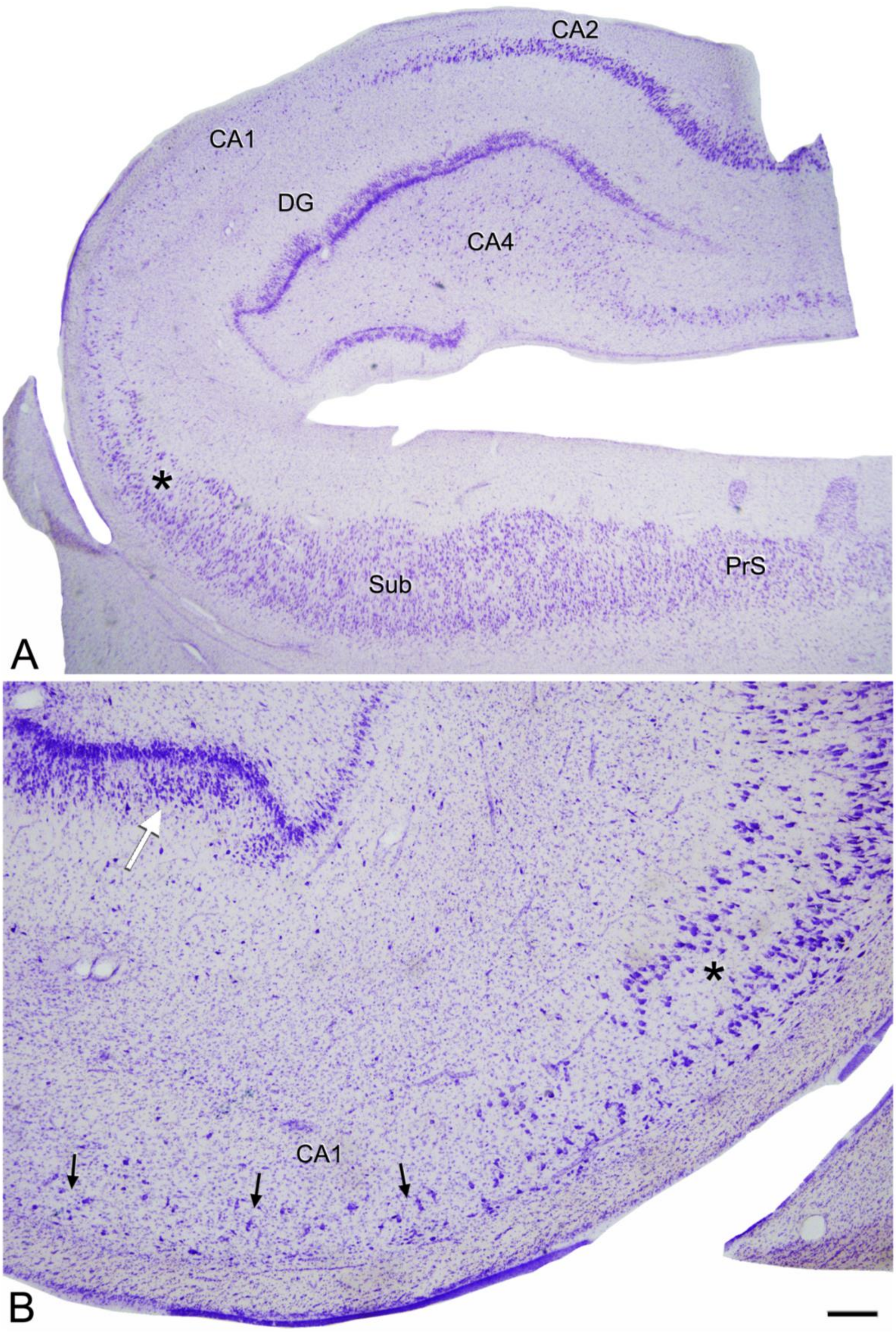

Figure 2-H94-1. **Photomicrographs of a Nissl-stained section**.
(**A**, **B**) Photomicrographs of a Nissl-stained section showing the hippocampal formation. Note the neuronal loss in the CA4, CA3 and CA1 fields. In CA1, at the border with the subiculum, neuronal loss is observed in the middle CA1 layer (one asterisk), whereas more proximal to CA2, most of the surviving cells are located in the deep CA1 layer (black arrows). The DG show a bi-laminar pattern of granule cell dispersion (arrow in **B**). Scale bar shown in (**B**) indicates 700 µm in (**A**) and 250 µm in (**B**). CA1-CA4: Cornu ammonis fields; DG: dentate gyrus; Sub: subiculum. PrS: presubiculum.

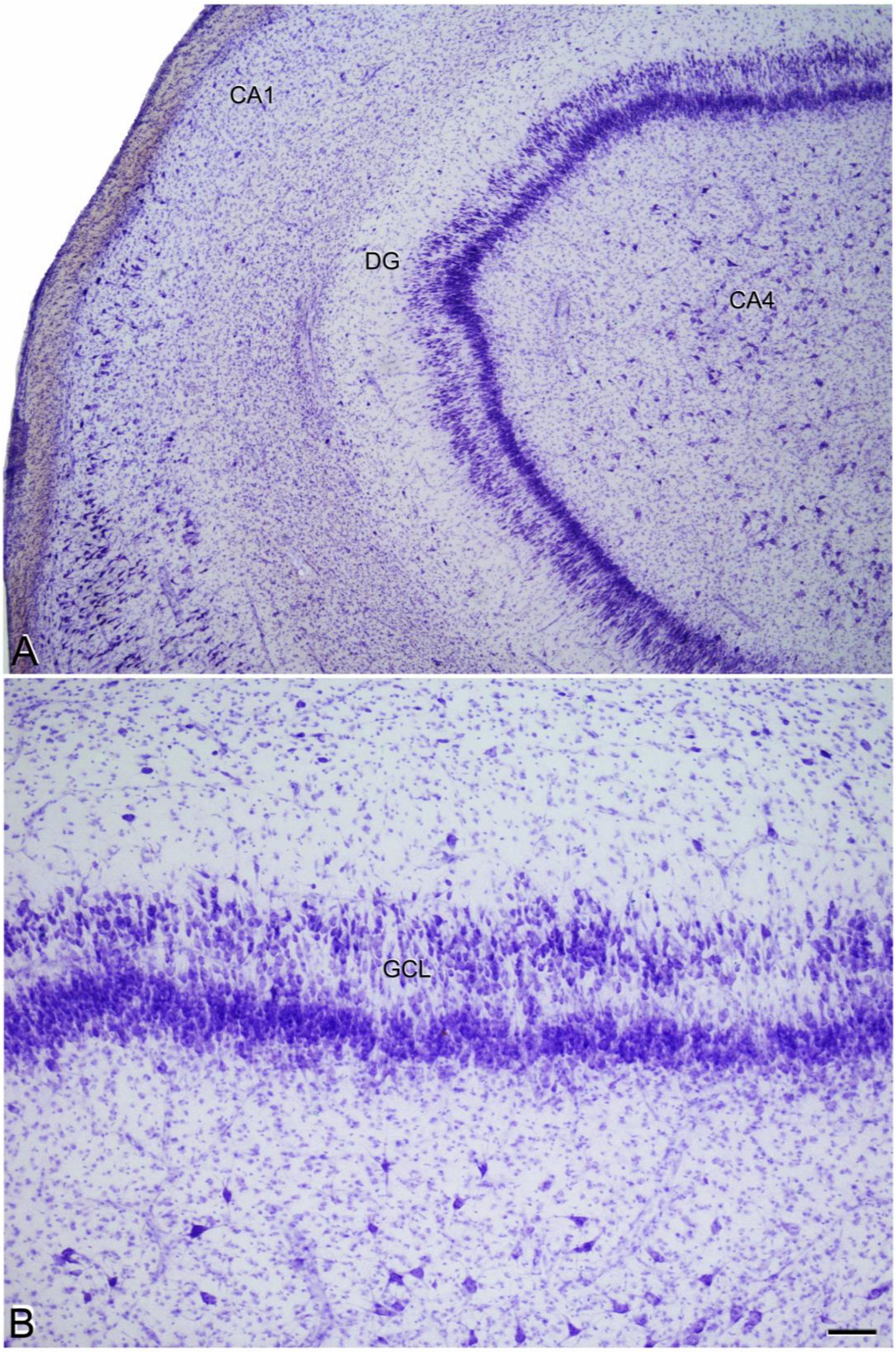

Figure 2-H94-2. Photomicrographs of a Nissl-stained section.

(**A**, **B**) Photomicrographs of a Nissl-stained section adjacent to that shown in Figure 2-H94-1A to illustrate with a greater detail CA4, CA1 and DG fields. Note the bi-laminar pattern of granule cell dispersion in the granule cell layer (GCL) of the DG (**B** is a higher magnification of **A**). Scale bar shown in (**B**) indicates 240 µm in (**A**) and 95 µm in (**B**). CA1-CA4: Cornu ammonis fields; DG: dentate gyrus.

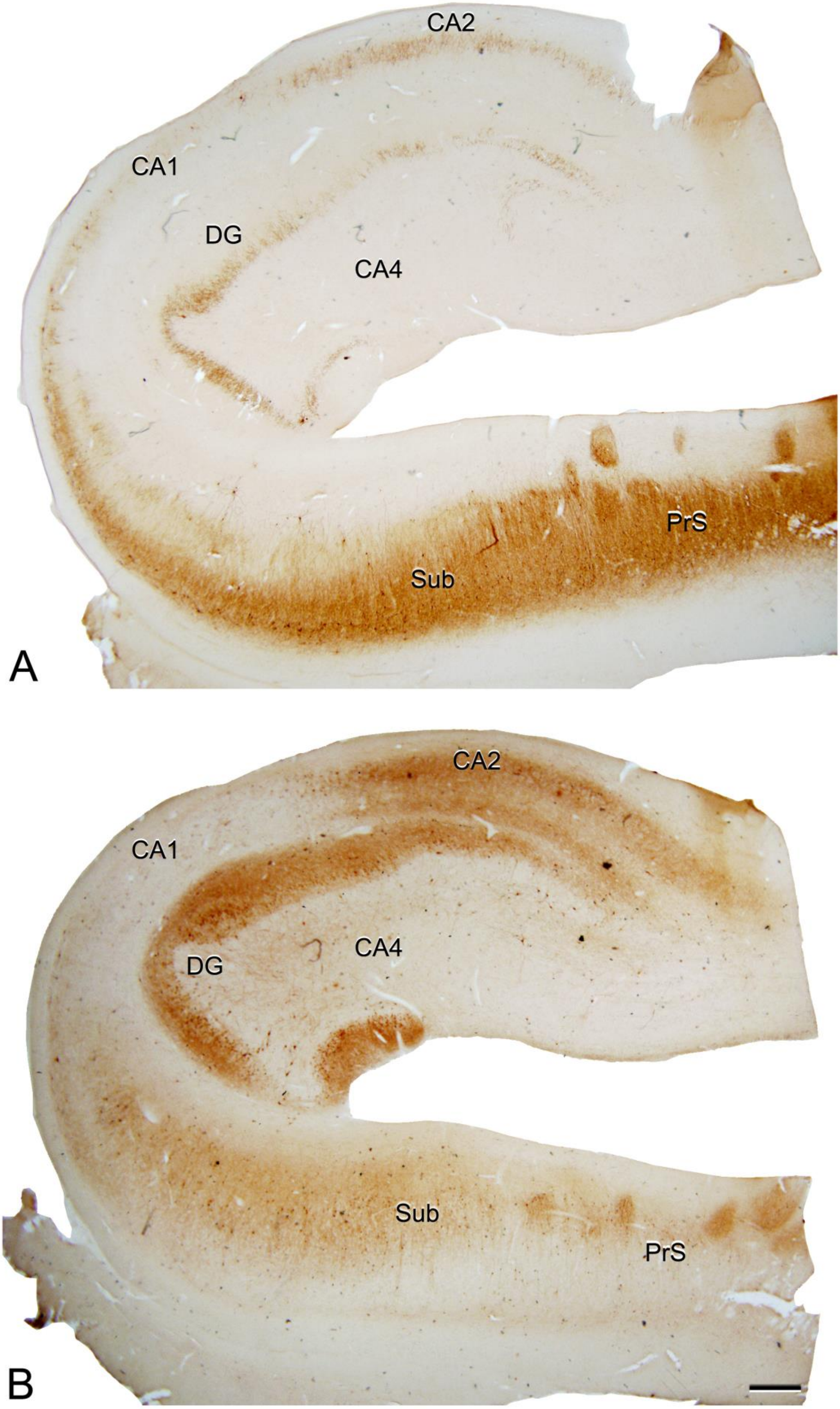

Figure 2-H94-3. Photomicrographs of PV- and CalB-immunostained sections
(**A**, **B**) Photomicrographs of a PV- (**A**) and a CalB-immunostained (**B**) sections adjacent to the Nissl-stained section showed in Figure 3-H94-1A. Note the different patterns of staining for PV and CalB. For example, the intensity of labeling for PV is higher in the Sub and CA1, whereas for CalB the intensity of labeling is higher in the DG and CA2. Scale bar shown in (**B**) indicates 700 µm in (**A)** and (**B**). CA1-CA4: Cornu ammonis fields; DG: dentate gyrus; Sub: subiculum. PrS: presubiculum.

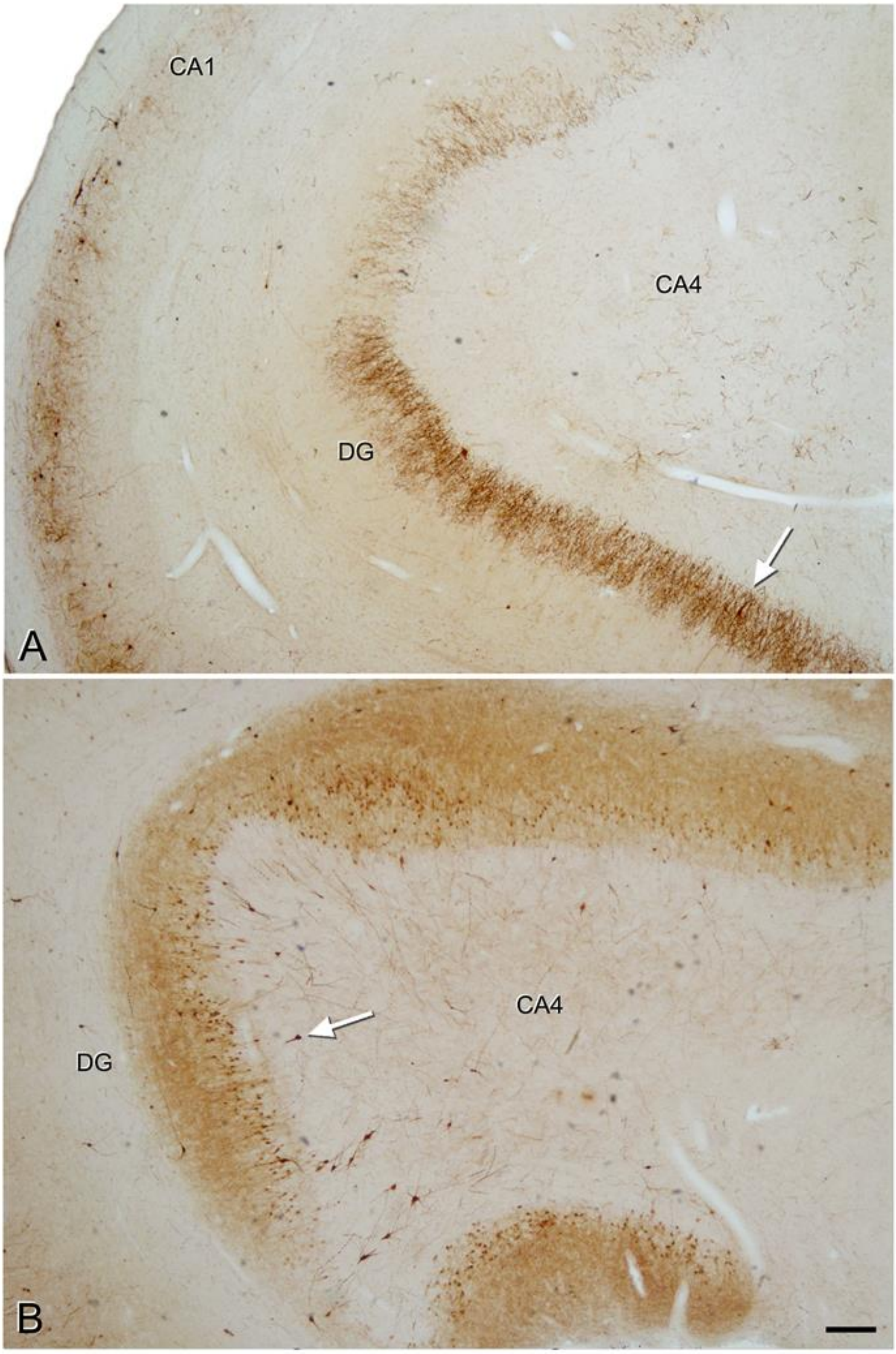

Figure 2-H94-4. Photomicrographs of PV- and CalB-immunostained sections
(**A**) Photomicrograph to illustrate the pattern of PV-immunostaining in the DG, CA4 and CA1 fields in an adjacent section to that shown in Figure 2-H94-3A. (**B**) higher magnification of Figure S2-H94-3B showing the pattern of CalB-immunostaining in the DG, CA4 and CA1 fields. Arrows in (**A)** and (**B**) indicates the same neurons at a higher magnification in (**A)** and (**B**) of Figure 2-H94-5, respectively. Scale bar shown in (**B**) indicates 250 μm in (**A)** and (**B**). CA1-CA4: Cornu ammonis fields; DG: dentate gyrus.

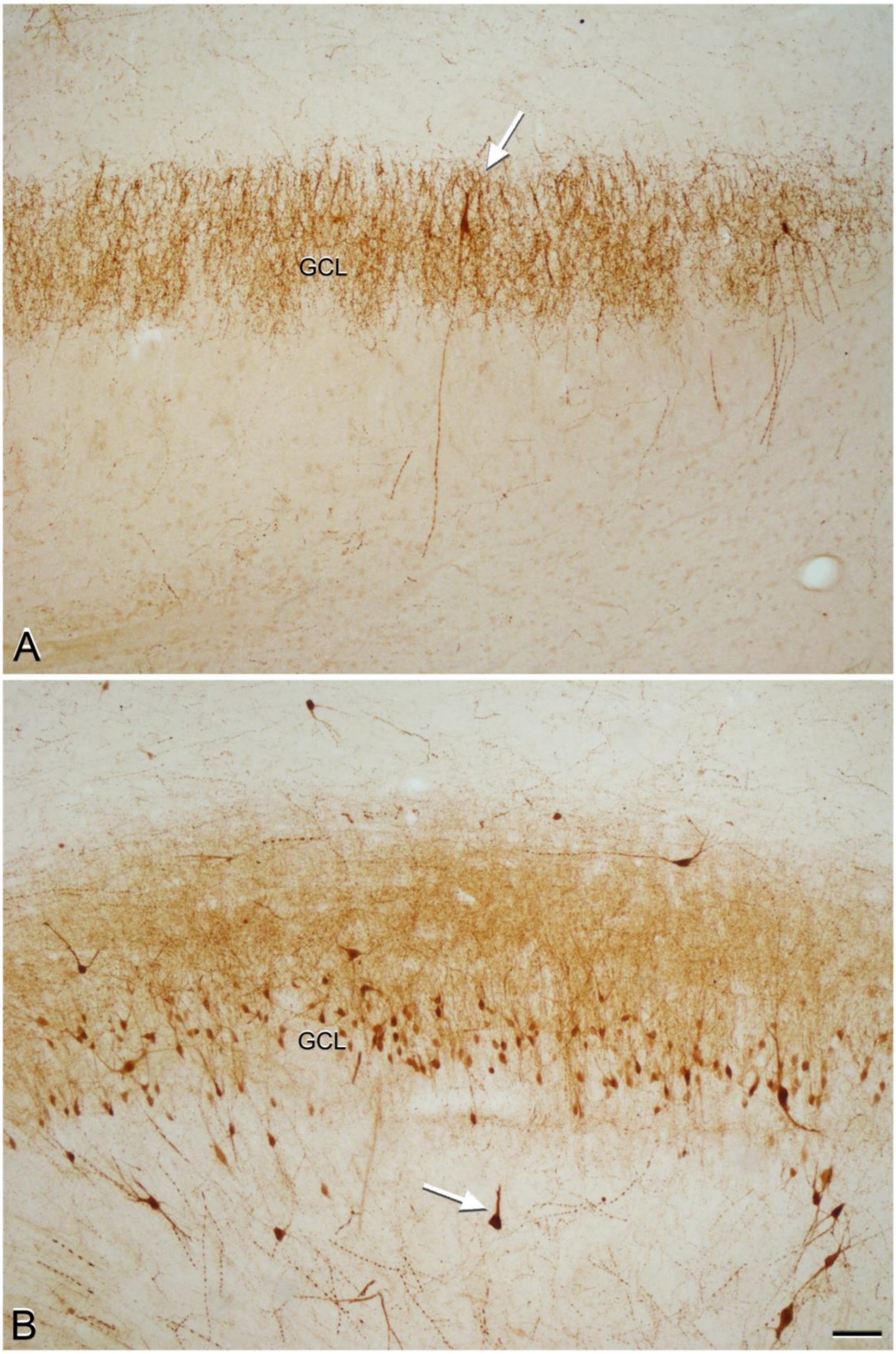

Figure 2-H94-5. Photomicrographs of PV- and CalB-immunostained sections

(**A**, **B**) Higher magnification of panels (**A**) and (**B**) of Figure 2-H94-4 showing the patterns of PV and CB immunostaining in the granule cell layer (GCL) of the dentate gyrus. Note that numerous CalB-immunostained neurons are present in the GCL. Arrows in (**A**) and (**B**) indicates the same neurons in (**A**) and (**B**) of Figure S2-H94-4, respectively. Scale bar shown in (**B)** indicates 95 μm in (**A**) and (**B**)
