# Supplementary material for: Neuroanatomical and psychological considerations in temporal lobe epilepsy
Source: Front Neuroanat. 2022 Dec 14;16:995286. doi: 10.3389/fnana.2022.995286 (PMC9794593; doi:10.3389/fnana.2022.995286)

H33

(left temporal lobe, non-sclerotic hippocampus)

Rey-Osterrieth Complex Figure

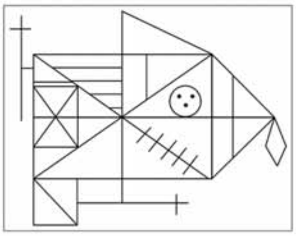

Before

Copy trial

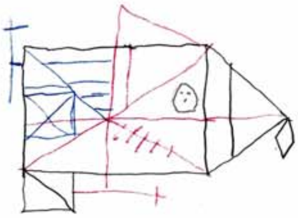

3’ trial

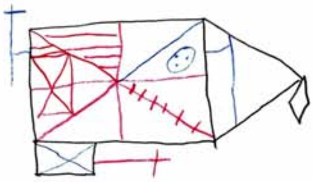

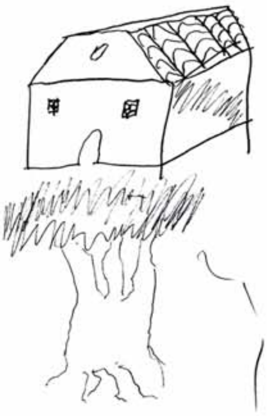

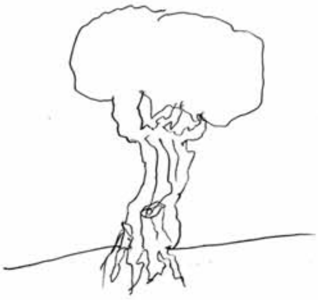

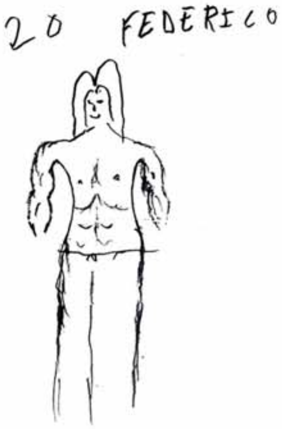

After

Copy trial

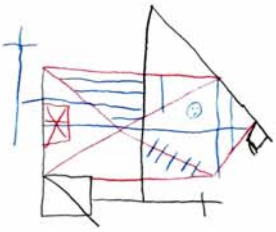

3’ trial

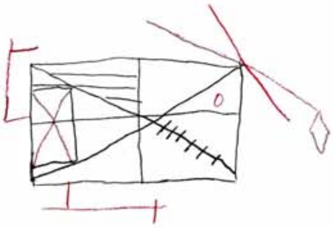

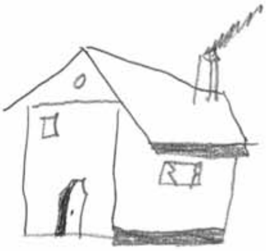

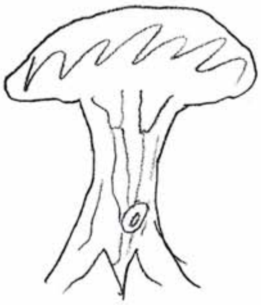

Supplement: Supplementary file 1 [file Data_Sheet_1.zip › Supplementary material/Supplementary Figures 3, patients with normal hippocampus/Patient H33.pdf]
