# Supplementary material for: Neuroanatomical and psychological considerations in temporal lobe epilepsy
Source: Front Neuroanat. 2022 Dec 14;16:995286. doi: 10.3389/fnana.2022.995286 (PMC9794593; doi:10.3389/fnana.2022.995286)

H61

(right temporal lobe, left-handed, sclerotic hippocampus)

Rey-Osterrieth Complex Figure

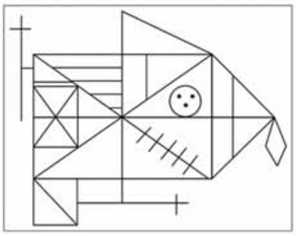

Before

After

Copy trial | 3' trial | Copy trial | 3' trial

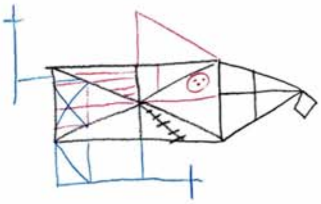

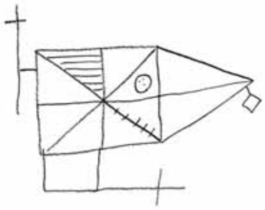

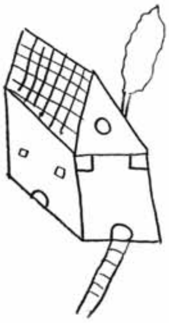

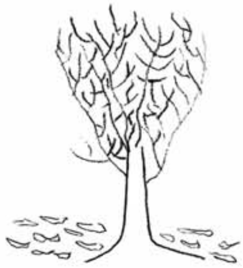

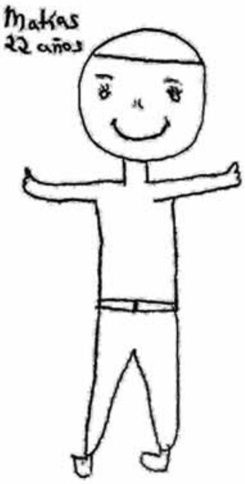

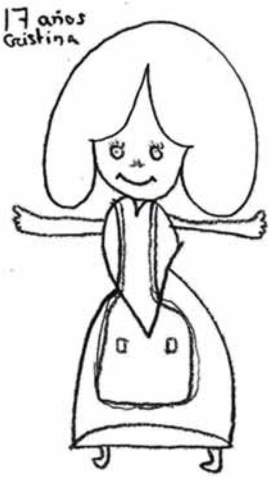

Supplement: Supplementary file 1 [file Data_Sheet_1.zip › Supplementary material/Supplementary Figures 4, patients with hpp. sclerosis/Patient H61.pdf]
