# Supplementary material for: Neuroanatomical and psychological considerations in temporal lobe epilepsy
Source: Front Neuroanat. 2022 Dec 14;16:995286. doi: 10.3389/fnana.2022.995286 (PMC9794593; doi:10.3389/fnana.2022.995286)
Supplement: Supplementary file 1 [file Data_Sheet_1.zip › Supplementary material/Supplementary Table 1.pdf]

Supplementary Table 1: Quantitative analysis of hippocampal histological alterations in the patients included in this review (related to table 4). Number of granule cells per column (100 µm wide; 30 µm thick) and neuronal densities of hippocampal subfields are shown expressed as percentages of controls.

NA indicates data not available. Statistically significant differences with control values were calculated with ANOVA (one-tail) and Dunnet-C post-hoc comparisons: * p<0.05; ** p<0.01; *** p<0.005. See Table 4 for values of GCL neuron numbers and neuronal densities. [♯] CA3 value of H136 and significance level of H44 CA1 value were noticed to be typos in Arellano et al. (2004); corrected values are listed here. Code of patients in bold indicates seizure-free after surgery.

Patient	Neurons/column (%)	Neuronal density (%)					
	GCL	Hilus	CA4	CA3	CA2	CA1	Sub
**H41**	100	69	127	129	89	87	110
**H44**	32***	17***	33***	50***	96	56*♯	111
**H48**	55*	64	69	79	79	39***	128
**H57**	79	13***	17***	35***	55***	35***	115
**H61**	59	114	118	99	103	37***	130
H65	76	92	96	108	122	92	100
H75	99	36***	38***	46***	48***	31***	175***
**H80**	127	103	101	108	97	35***	137***
**H84**	28***	10***	14***	NA	14***	20***	102
H85	99	140	166***	111	NA	96	109
**H94**	90	37***	24***	73	101	28***	104
**H104**	67	29***	32***	NA	55***	33***	111
H108	48***	13***	14***	27***	41***	33***	126
**H109**	87	94	82	105	97	46***	122
H115	45***	22***	27***	43***	66***	23***	93
**H123**	99	42**	80	61	79	37***	110
**H136**	87	20***	54*	30***♯	45***	31***	125
H138	81	47*	27***	40***	72*	38***	153***
