# Supplementary material for: Neuroanatomical and psychological considerations in temporal lobe epilepsy
Source: Front Neuroanat. 2022 Dec 14;16:995286. doi: 10.3389/fnana.2022.995286 (PMC9794593; doi:10.3389/fnana.2022.995286)
Supplement: Supplementary file 1 [file Data_Sheet_1.zip › Supplementary material/Supplementary Table 2.pdf]

Supplementary Table 2: Distribution and the neurochemical characteristics of the axon terminals of chandelier cells (Ch-terminals) in the dentate gyrus and CA fields in epileptic patients. The qualitative data and the description of the methods used to generate this table were taken from Arellano et al (2004). Density of Ch-terminals ranges from 0 (no staining) to +++ (high density of Ch-terminals). C, indicates presence of complex Ch-terminals. NA, indicates data not available. Hilus and CA4 are described together. In addition to shown fields, subsets of Ch-terminals in the upper pyramidal layer of the normal subiculum were found to express CB. GAT-1 (GABA transporter 1). CB (calcium binding protein calbindin D-28k). Code of patients in bold indicates seizure-free after surgery.

**Patient**	**Granular layer**		**Hilus & CA4**		**CA3**		**CA2**		**CA1**	
	GAT-1	CB	GAT-1	CB	GAT-1	CB	GAT-1	CB	GAT-1	CB
**H44**	NA	0	NA	0	NA	0	NA	0	NA	+C
**H48**	+C	0	+	0	+	0	++	0	+++C	+C
**H57**	+	0	0	0	0	0	+C	0	+++C	++
**H61**	0	0	0	0	0	NA	0	NA	+	NA
H75	0	0	0	0	0	0	+	0	+	++
**H84**	+C	+C	0	0	NA	NA	0	+	+++C	+C
**H94**	+C	0	+C	0	+C	+	++C	+C	++C	+
**H104**	++	0	+C	0	NA	NA	+C	+C	++C	++C
H108	++C	+C	+C	0	+C	0	++C	0	++C	+
**H109**	0	+C	+	0	++C	0	+	0	++C	+
H115	+++C	++C	0	+C	NA	NA	+C	++C	+	+
**H123**	+	+C	0	0	0	0	+	0	++C	+
**H136**	0	0	0	0	0	0	+C	0	++C	+
H138	0	0	0	0	0	0	+C	0	++C	+
