# Supplementary material for: Neuroanatomical and psychological considerations in temporal lobe epilepsy
Source: Front Neuroanat. 2022 Dec 14;16:995286. doi: 10.3389/fnana.2022.995286 (PMC9794593; doi:10.3389/fnana.2022.995286)
Supplement: Supplementary file 1 [file Data_Sheet_1.zip › Supplementary material/Supplementary Table 3.pdf]

Supplementary Table 3: Relative abundance of parvalbumin (PV) immunostaining of basket formations (Bas), Ch-terminals (Ch) and other axonal and dendritic processes in the neuropil (Nepil) in the dentate gyrus, hilus, CA4, CA3 and CA2 in epileptic patients. Density of immunostained elements ranges from 0 (no staining) to +++ (high density of labeled elements). C indicates presence of complex Ch- terminals. NA, indicates no data available. See Arellano et al. 2004 for technical details. Code of patients in bold indicates seizure-free after surgery.

**Patient**	**GD**			**Hilus & CA4**			**CA3**			**CA2**		
	**Bas**	**Ch**	**Nepil**	**Bas**	**Ch**	**Nepil**	**Bas**	**Ch**	**Nepil**	**Bas**	**Ch**	**Nepil**
**H44**	+	+	+	NA	NA	NA	NA	NA	NA	NA	NA	NA
**H48**	++	++C	+	++	++C	+	+	+C	-	++	+++C	++
**H57**	+	+C	++	0	0	+	0	0	0	0	+C	0
**H61**	++	+	+	0	+C	+	0	0	+	NA	NA	NA
H75	++	+C	++	+	+C	+	+	+C	+	+	+C	+
**H84**	++	++C	++	+	0	0	+	+C	+	++	++C	+++
**H94**	+	+C	+	+	+C	+	+	+C	+	+	+C	+
**H104**	+	+C	+	+	+C	+	NA	NA	NA	++	++C	+
H108	+	+C	+	0	0	0	0	0	0	0	+	0
**H109**	++	++C	++	+	+C	+	++	0	++	++	+	++
H115	+	++C	+	0	0	+	NA	NA	NA	+	++C	+
**H123**	+	+C	+	0	+C	+	0	0	0	+	+	+
**H136**	++	+	+	+	0	+	+	0	+	+	0	+
H138	+	+C	+	0	0	+	0	+C	0	+	++C	+
