# Supplementary material for: Neuroanatomical and psychological considerations in temporal lobe epilepsy
Source: Front Neuroanat. 2022 Dec 14;16:995286. doi: 10.3389/fnana.2022.995286 (PMC9794593; doi:10.3389/fnana.2022.995286)
Supplement: Supplementary file 1 [file Data_Sheet_1.zip › Supplementary material/Supplementary Table 4.pdf]

Supplementary Table 4: Distribution of complex chandelier cell axon terminal (Ch-terminals) in the granular layer of the dentate gyrus of epileptic patients, as revealed with immunocytochemistry for parvalbumin (PV), GABA transporter 1 (GAT-1) and calbindin D-28k (CB). NA, indicates data not available; 0, indicates no labeling. Focal, indicates that complex Ch-terminals occupy less than 50% of the GCL length; Extensive, indicates complex Ch-terminals occupy 50% or more of the GCL length. Code of patients in bold indicates seizure-free after surgery.

**Patient**	**GAT-1**	**PV**	**CB**
**H44**	NA	0	0
**H48**	Focal	Focal	0
**H57**	0	Focal	0
**H61**	0	0	0
H75	0	Extensive	0
**H84**	Focal	Extensive	Focal
**H94**	Focal	Extensive	0
**H104**	0	Focal	0
H108	Focal	Focal	Focal
**H109**	0	Focal	Focal
H115	Focal	Extensive	Extensive
**H123**	0	Focal	Focal
**H136**	0	0	0
H138	0	Focal	0
