# Supplementary material for: Neuroanatomical and psychological considerations in temporal lobe epilepsy
Source: Front Neuroanat. 2022 Dec 14;16:995286. doi: 10.3389/fnana.2022.995286 (PMC9794593; doi:10.3389/fnana.2022.995286)
Supplement: Supplementary file 1 [file Data_Sheet_1.zip › Supplementary material/Supplementary Table 5.pdf]

Supplementary Table 5. Features, mean coefficient values and percentages of inclusion.

Features included in at least 25% of all the logistic regression models from a multistart pool of 10,000 runs. Column Mean Coefficient Value display the mean value associated to each feature. Percentage column shows the relative frequency each feature was included in the models. This table presents the top 10 features for the machine learning model when predicting recovery from surgery. Due to the low number of samples, we performed a total of 10,000 random multistart runs to detect central tendencies in the detection of key features. The first four features in the table were included in every 3 out of 4 inferred models, indicating a strong relevance as predictors. The mean coefficient illustrates how each of these features weight in the regression: those with a negative coefficient correlates with the negative class (Engel I), whereas features with a positive coefficient directly correlates with the positive class (Engel II or III). A more detailed explanation of said influences for the four first-ranked features by using the SHAP coefficients is presented in Fig. 11.

**Feature**	**Mean Coefficient Value**	**Percentage (%) of inclusion**
HVI_post	-0.796051110801316	99.77
S-CON_pre	0.853269652049734	99.30
Sclerosis	-0.734157593641958	95.58
DEP_pre	-0.504741089835896	83.25
DEPI_pre	0.635987538983057	74.19
DS_pre	0.273744522442318	53.49
PAR_pre	-0.286668727364873	43.02
Sum_Y_post	-0.311775373487352	41.40
SCH_post	0.167521266013375	31.63
ROCF_Copy_pre	-0.146522313049485	26.28

The "_pre" or "_post" suffixes in each feature's identifier correspond to values measured before or after surgery, respectively. HVI: Hypervigilance Index (Rorschach test); S-CON: Suicide constellation score (Rorschach test); Sclerosis: Presence or absence of presence or absence of hippocampal sclerosis in the resected tissue; DEP: Depression (clinical interview); DEPI: Depression index (Rorschach test); DS: digit backward test (WAIS scale); PAR: Paranoia (clinical symptom); Sum_Y: Sum of diffuse shading responses (Rorschach test); SCH: Schizophreniform (clinical symptom); ROCF Copy: Percentile of the copy test (ROCF battery).
